# α-Glucosidase and α-Amylase Inhibitory Potentials of Quinoline–1,3,4-oxadiazole Conjugates Bearing 1,2,3-Triazole with Antioxidant Activity, Kinetic Studies, and Computational Validation

**DOI:** 10.3390/ph15081035

**Published:** 2022-08-22

**Authors:** Nosipho Cele, Paul Awolade, Pule Seboletswe, Kolawole Olofinsan, Md. Shahidul Islam, Parvesh Singh

**Affiliations:** 1 School of Chemistry and Physics, University of KwaZulu-Natal, P/Bag X54001, Westville, Durban 4000, South Africa; 2 Department of Biochemistry, School of Life Sciences, University of Kwazulu-Natal, Westville, Durban 4000, South Africa

**Keywords:** quinoline, 1,3,4-oxadiazole, 1,2,3-triazole, α-glucosidase inhibitor, enzyme kinetics, molecular dynamics

## Abstract

Diabetes mellitus (DM) is a multifaceted metabolic disorder that remains a major threat to global health security. Sadly, the clinical relevance of available drugs is burdened with an upsurge in adverse effects; hence, inhibiting the carbohydrate-hydrolyzing enzymes α-glucosidase and α-amylase while preventing oxidative stress is deemed a practicable strategy for regulating postprandial glucose levels in DM patients. We report herein the α-glucosidase and α-amylase inhibition and antioxidant profile of quinoline hybrids **4a**–**t** and **12a**–**t** bearing 1,3,4-oxadiazole and 1,2,3-triazole cores, respectively. Overall, compound **4i** with a bromopentyl sidechain exhibited the strongest α-glucosidase inhibition (IC_50_ = 15.85 µM) relative to reference drug acarbose (IC_50_ = 17.85 µM) and the best antioxidant profile in FRAP, DPPH, and NO scavenging assays. Compounds **4a** and **12g** also emerged as the most potent NO scavengers (IC_50_ = 2.67 and 3.01 µM, respectively) compared to gallic acid (IC_50_ = 728.68 µM), while notable α-glucosidase inhibition was observed for *p*-fluorobenzyl compound **4k** (IC_50_ = 23.69 µM) and phenyl-1,2,3-triazolyl compound **12k** (IC_50_ = 22.47 µM). Moreover, kinetic studies established the mode of α-glucosidase inhibition as non-competitive, thus classifying the quinoline hybrids as allosteric inhibitors. Molecular docking and molecular dynamics simulations then provided insights into the protein–ligand interaction profile and the stable complexation of promising hybrids at the allosteric site of α-glucosidase. These results showcase these compounds as worthy scaffolds for developing more potent α-glucosidase inhibitors with antioxidant activity for effective DM management.

## 1. Introduction

Diabetes Mellitus (DM) is a chronic systemic disorder characterized by dysfunctional glucose regulation. Typically, patients secrete insufficient insulin (i.e., type 1 diabetes mellitus–T1DM) or develop insulin resistance (i.e., type 2 diabetes mellitus–T2DM), which leads to elevated blood glucose levels (hyperglycemia) [1]. Complications associated with DM include nervous system damage, eye damage, cardiovascular disease, stroke, kidney disease, and gangrene [2]. The disease persists as a heavy burden on public health, having become the ninth leading cause of death with approximately 451 million incidences globally [3]. Recent estimations have shown increased incidences in Africa, with about 15.5 million cases in 2017, and that 69.2% of the adult population are ignorant of their diabetic status [4]. T2DM has a higher incidence rate globally, with about 90% or more of the cases relative to T1DM.

T2DM can be managed by regulating the key molecular targets involved in carbohydrate digestion i.e., α-glucosidase and pancreatic α-amylase [5]. These enzymes break down dietary carbohydrates into simple sugars which are absorbed from the small intestine into the bloodstream. Elevated levels of α-glucosidase and α-amylase are therefore associated with increased postprandial glucose levels seen in T2DM patients, making their inhibition a successful strategy for T2DM management [6]. Nevertheless, the availability of α-glucosidase inhibitors is currently restricted to *N*-heterocyclic carbasugars such as acarbose, voglibose, and miglitol [7]. These molecules present sugar-like moieties that compete with the enzyme’s natural substrates for binding at the active site to inhibit sugar hydrolysis, and consequently decrease postprandial hyperglycemia [8]. Although these drugs have rapid action, they are marred by efficacy problems and adverse side effects such as diarrhea, flatulence, and abdominal discomfort [9], which precipitates the need for new and safer α-glucosidase inhibitors.

Reactive oxygen species (ROS), including free radicals of superoxide anion (O_2_^−^), hydroxyl (OH^−^) radicals, and hydrogen peroxide (H_2_O_2_), play a prominent role in regulating cellular functions as their intermediates control various enzymatic reactions involved in signal transductions [10]. However, their excessive accumulation contributes to toxic cellular oxidative stress which induces the pathogenesis of diseases such as T2DM [11]. Therefore, the development of small molecules with antidiabetic and free radical scavenging potentials is considered an attractive option for T2DM management.

**Figure 1:**
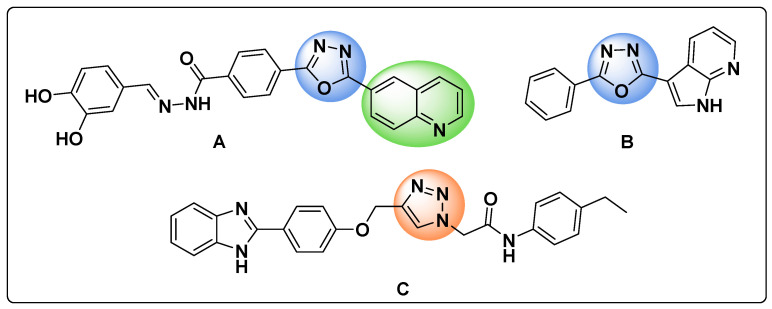
Reported α-glucosidase inhibitors based on quinoline, 1,3,4-oxadiazole, and 1,2,3-triazole cores.

The medicinal chemistry significance of the quinoline scaffold has been reiterated in diverse therapeutic applications including antimicrobial [12], antimalarial [13], anticancer [14], antidiabetic [15], and antioxidant [16] therapies. Among the quinoline congeners, 8-hydroxyquinoline (**8**-**HQ**) has attracted attention due to its metal chelating properties, a characteristic which may be beneficial to attenuating the pathogenesis of several diseases including diabetes [17,18,19]. The 1,3,4-oxadiazole moiety is another relevant pharmacophore, and is a known bioisostere of amide and ester groups. The scaffold’s bioactivity has been linked to its strong hydrogen bond interactions with various targets [20,21]. The electronic effects of the azole stimulate ligand binding and improve lipophilicity for active transport through the cell membrane to various targets to elicit a biological response [20]. For example, Taha et al. [22] showed that a quinoline-1,3,4-oxadiazole hybrid **A** (Figure 1) had 15-fold superior α-glucosidase inhibition (IC_50_ = 2.6 µM) compared with acarbose (IC_50_ = 38.25 µM). Izgi et al. [23] reported the 1,3,4-oxadiazole compound **B** bearing an azaindole core, a potent α-glucosidase inhibitor with IC_50_ = 0.4 mM compared with acarbose IC_50_ = 1.75 mM. Furthermore, the 1,2,3-triazole core is renowned in medicinal chemistry as a five-membered ring with a wide range of biological activity [24,25]. The fame of the pharmacophore’s therapeutic potential has incessantly increased since the dawn of click chemistry, described by Sharpless [26]. As a result, several α-glucosidase inhibitors have been documented with 1,2,3-triazole moieties in their structures [27]. Asemanipoor et al. [28] reported the potent α-glucosidase inhibition (IC_50_ = 35.0 µM) of a 1,2,3-triazole derivative C compared with acarbose standard (IC_50_ = 750.0 µM).

Although quinoline, 1,3,4-oxadiazole, and 1,2,3-triazole moieties have been reported as α-glucosidase inhibitors in many molecular hybrid designs [29], these findings require further investigation to achieve optimal results. Consequently, in continuation of our exploits within the MH approach towards quinoline, 1,3,4-oxadiazole, and 1,2,3-triazole hybrids with therapeutic potentials [30,31], we herein incorporated these pharmacophores in a single molecular hybrid and evaluated the effect on the in vitro α-glucosidase and α-amylase inhibitory potencies and antioxidant activities. We also examined the mode of α-glucosidase inhibition of promising compounds via kinetic studies, then rationalized the results with molecular docking and molecular dynamics simulations.

## 2. Results and Discussion

### 2.1. Chemistry

**Scheme 1:**
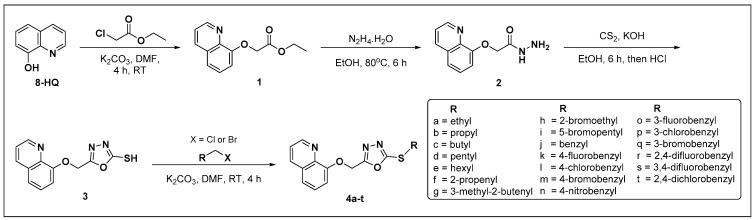
Synthetic route to quinoline-1,3,4-oxadiazole hybrids.

**Scheme 2:**
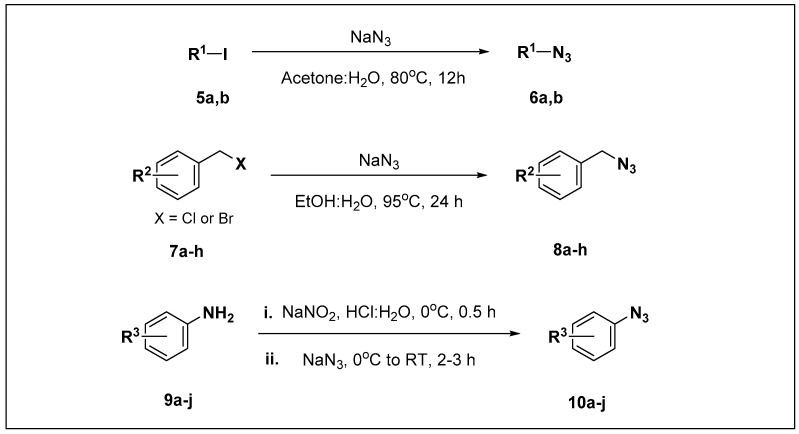
Synthesis of requisite azide substrates for the 1,3-dipolar cycloaddition reaction.

**Scheme 3:**
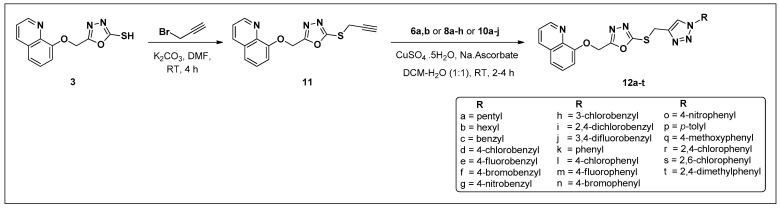
Synthesis of quinoline-1,3,4-oxadiazole-1,2,3-triazole hybrids via Cu(I)-catalyzed azide-alkyne [3 + 2] cycloaddition reaction.

The synthetic route to the targeted quinoline hybrids is depicted in Scheme 1, Scheme 2 and Scheme 3. First, compounds **4a**–**t** were synthesized in four steps to examine the influence of 1,2,3-triazole moiety on the bioactivity of the present molecular design (Scheme 1). The reaction protocol started by *O*-alkylating 8-hydroxyquinoline (**8**-**HQ**) with ethyl chloroacetate in *N*,*N*-dimethylformamide (DMF) at room temperature to give ethyl 2-(quinolin-8-yloxy)acetate **1**. The ester intermediate was then refluxed with hydrazine hydrate in ethanol to give 2-(quinolin-8-yloxy)acetohydrazide **2**. A potassium hydroxide-promoted condensation of **2** with carbon disulfide afforded a potassium carbodithioate salt, which subsequently underwent hydrochloric acid-promoted intramolecular cyclization to yield 5-[(quinolin-8-yloxy)methyl]-1,3,4-oxadiazole-2-thiol **3** in 65% yield over 3 steps. A potassium carbonate-promoted *S*-alkylation of this thiol intermediate with different benzyl and alkyl halides, respectively, afforded the desired quinoline–1,3,4-oxadiazole hybrids **4a**–**t** in moderate to quantitative yields. 

To obtain hybrids **12a**–**t** bearing the 1,2,3-triazole pharmacophore, the azide precursors needed for the Cu(I)-catalyzed azide–alkyne [3 + 2] cycloaddition reaction (click reaction) were synthesized according to their substrate demands (Scheme 2).

With the azides at hand, intermediate **3** was treated with propargyl bromide in DMF in the presence of potassium carbonate to give the corresponding terminal alkyne **11**. This product was subjected to a click reaction with azides **6a**, **b**, or **8a**–**h**, or **10a**–**j** in a DCM:H_2_O (1:1) solvent mixture to afford the target quinoline–1,3,4-oxadiazole–1,2,3-triazole hybrids **12a**–**t** in 43–91% yields (Scheme 3).

### 2.2. Structural Elucidation

**Figure 2:**
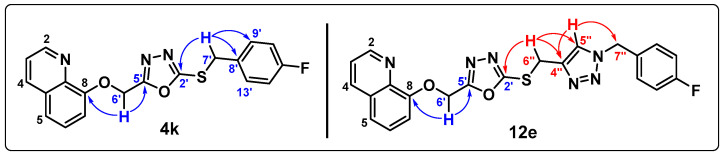
Schematic representation of key HMBC correlations in compounds **4k** and **12e**.

The synthetic protocol’s success was established using spectroscopic experiments, viz., NMR (^1^H, ^13^C and 2D) and HRMS. The emergence of -NH (*δ*_H_ 9.45) and -NH_2_ (*δ*_H_ 4.37) signals in the ^1^H NMR spectrum of compound **2** and the absence of ethoxy group quartet (*δ*_H_ 4.29, *J* = 7.1 Hz) and triplet (*δ*_H_ 1.28, *J* = 7.1 Hz) signals of compound **1**, respectively, confirmed the ester group’s conversion to hydrazide. Likewise, the formation of the 2-mercapto-1,3,4-oxadiazole ring was evidenced by a broad singlet at *δ*_H_ 14.77 corresponding to the thiol group’s deshielded proton. The disappearance of this peak and the appearance of doublet (CH_2_, *δ*_H_ 4.17, *J* = 2.6 Hz) and (CH, *δ*_H_ 3.32, *J* = 2.6 Hz) triplet signals of an allylic alkyne unit confirmed the conversion of **3** to **11**. Furthermore, the structural integrity of quinoline hybrids **4a**–**t** and **12a**–**t** was confirmed by 2D NMR experiments, i.e., heteronuclear single quantum coherence (HSQC) and heteronuclear multiple bond coherence (HMBC) correlations. For instance, in compound **4k**, the correct linkage of quinoline to 1,3,4-oxadiazole was seen in the HMBC correlations of H-6′ (*δ*_H_ 5.61) to C-8 and C-5′quaternary carbons at 153.33 and 164.32 ppm respectively (Figure 2). H-7′ (*δ*_H_ 4.51) correlations to C-2′ (*δ*_C_ 164.82), C-8′ (*δ*_C_ 133.22, d, *J* = 3.0 Hz), and C-9′/C-13′ (*δ*_C_ 131.58, d, *J* = 8.3 Hz, C-9′,13′) showed that alkylation occurred at the sulfur atom of oxadiazole and not the nitrogen of a possible thione tautomer. In the 1,2,3-triazole series, e.g., **12e**, the characteristic triazole H-5″ singlet peak at *δ*_H_ 7.70 showed two HMBC correlations with C-7″ (*δ*_C_ 53.43) and C-4″ (*δ*_C_ 143.25), while H-6″ (*δ*_H_ 4.51) correlated to C-2′ (*δ*_C_ 163.45), C-4″ (*δ*_C_ 143.25), and C-5″ (*δ*_C_ 123.24). Additionally, the molecular ion peaks corresponding to [M + Na] at *m*/*z* 390.0696 and 457.0851 in the HRMS spectra of **4k** and **12e**, respectively, also affirmed the quinoline hybrid’s structural integrity.

### 2.3. Antioxidant Activity Profiling

#### 2.3.1. 2,2-Diphenyl-1-Picrylhydrazyl (DPPH) Radical Scavenging Activity

The DPPH assay is used conventionally to quantify a compound’s ability to trap free radicals. The relative efficacy of compounds **4a**–**t** and **12a**–**t** was evaluated in this regard; the results are shown in Table 1. Except for the hybrids’ precursor, compound **3** (IC_50_ of 25.15 µM), the compounds in both series exhibited moderate free radical scavenging activity relative to the reference scavenger, gallic acid (IC_50_ = 26.63 µM). The 1,3,4-oxadiazole series **4a**–**t** were again more potent than the 1,2,3-triazoles **12a**–**t**. SAR analysis showed that 2-mercapto-1,3,4-oxadiazole core was beneficial to DPPH scavenging activity, as seen in the two-fold improved potency of compound **3** as compared to **8**-**HQ** (IC_50_ = 51.81 µM); however, *S*-alkylation was found generally detrimental to potency.

#### 2.3.2. Ferric Reducing Antioxidant Power (FRAP)

FRAP is a ferric (Fe^3+^) to ferrous (Fe^2+^) ions reduction assay that measures a compound’s electron-donating capacity, i.e., the antioxidant’s reducing power [32]. The results in Table 2 reveal that compound **4i** was the most promising hybrid in this regard; IC_50_ = 22.88 µM. The compound’s FRAP activity was four-fold superior to standard compound ascorbic acid (IC_50_ = 102.62 µM) and comparable to gallic acid (IC_50_ = 17.85 µM).

#### 2.3.3. Nitric Oxide (NO) Activity

The screening of the present compound series for NO radical scavenging activity (Table 2) identified excellent NO scavengers. Compound **4a** from the 1,3,4-oxadiazole series, bearing an ethyl chain, showed the highest NO scavenging activity with IC_50_ = 2.67 µM. Compound **4i**, with IC_50_ = 197.93 µM, displayed a four-fold higher efficacy compared with gallic acid (IC_50_ = 782.68 µM), to emerge as the best antioxidant overall. From the 1,2,3-triazole series, para-nitro benzyl compound **12g** showed excellent NO scavenging ability with IC_50_ = 3.01 µM, contrary to its 1,3,4-oxadiazole congener compound **4n** (IC_50_ > 500 µM).

### 2.4. Enzyme Inhibition and Structure–Activity Relationship (SAR) Analysis

Abounding empirical evidence highlights the significance in the pathogenesis of T2DM of carbohydrate-digesting enzymes α-glucosidase and α-amylase; hence, inhibiting these enzymes is crucial to T2DM management [33]. The inhibitory potencies of quinoline hybrids **4a**–**t** and **12a**–**t** against these enzymes are presented in Table 2. The compounds show low micromolar α-glucosidase inhibition with IC_50_ values ranging between 15.85 to 63.59 µM, in contrast to their moderate α-amylase inhibition. The quinoline–1,3,4-oxadiazole series **4a**–**t** are generally stronger α-glucosidase inhibitors compared with their 1,2,3-triazole congeners **12a**–**t**. This SAR is conceivably due to the increased lipophilicity and polar surface area of the 1,2,3-triazole core. Interestingly, the less lipophilic phenyl compounds **12k**–**t** were also better α-glucosidase inhibitors compared with the more lipophilic benzyl compounds **12a**–**j**. 

**Figure 3:**
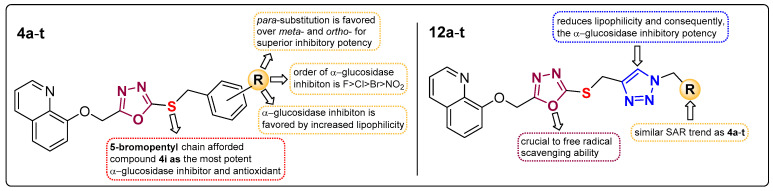
SAR summary of α-glucosidase inhibition and antioxidant activity.

In the 1,3,4-oxadiazole series **4a**–**t**, propyl was identified as the optimal alkane chain, as seen in the α-glucosidase inhibitory potencies of **4a**–**f**. However, replacing these alkyl chains with a bromopentyl unit led to the most potent α-glucosidase inhibitor, compound **4i** (IC_50_ = 15.85 µM) with a superior potency compared to standard drug acarbose (IC_50_ = 17.85 µM). Among the benzyl analogues, para-fluoro substituted compound **4k** (IC_50_ = 23.69 µM) was twice as potent as the meta-fluoro analogue **4o** (IC_50_ = 48.84 µM). The trend was seen in the chloro (**4l**, **4p**) and bromo (**4m**, **4q**) substituted compounds, respectively. This shows that altering the substituent’s position from para to meta is detrimental to α-glucosidase inhibition, and the order of potency in the para-substituted derivatives is F > Cl > Br > NO_2_. It is also inferable that inhibitory potency is dependent on the halogen’s electronegativity. The non-tolerance for meta or ortho positions was again highlighted in disubstituted compounds **4q**–**4t** which had inferior potencies relative to their monosubstituted congeners. Interestingly, those SARs were repeated in the **12a**–**t** series. Compound **12d** (IC_50_ = 38.11 µM) was more potent than **12h** (IC_50_ = 63.59 µM) and **12i** (IC_50_ = 42.79 µM). Conversely, both series exhibited poor α- amylase inhibition. A schematic SAR summary of the antioxidant profiling and α-glucosidase inhibition for the quinoline hybrids is presented in Figure 3.

### 2.5. Mode of α-Glucosidase Inhibition: Enzyme Kinetic Studies

**Figure 4:**
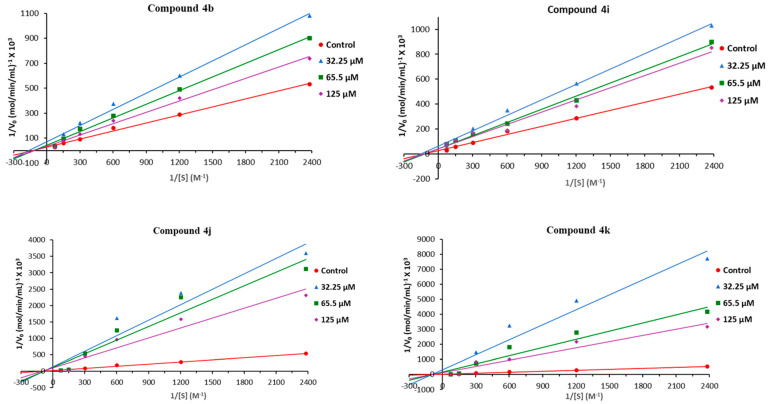

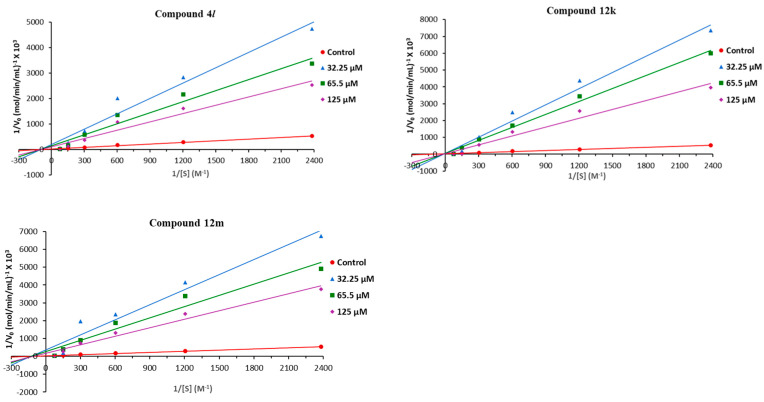
Lineweaver–Burk plots of promising α-glucosidase inhibitors.

Understanding a drug’s mode of inhibiting its target is crucial to improve the drug’s activity profile and target specificity. Although orthosteric inhibition, in which the ligand binds at the target’s active site, dominates among currently available drugs, allosteric inhibitors (i.e., drugs binding at regions close to the active site) are desirable due to their reduced vulnerability to active site mutation or to displacement through native substrate overload at the active site [34]. Accordingly, the mode of α-glucosidase inhibition was investigated for promising compounds 4b, 4i, 4j, 4k, 4l, 12k, and 12m, using a time-dependent para-nitrophenyl-β-D-glucopyranoside (*p*NPG) assay; the results are presented as Lineweaver–Burk plots (Figure 4). Clearly, all the compounds exhibited a non-competitive type of enzyme inhibition as the maximum reaction velocity (Vmax) changed for each inhibitor concentration tested, but the Michaelis–Menten constant (Km) remained unchanged. This indicates that the present compounds bind at the allosteric sites of free α-glucosidase, to inhibit its hydrolytic activity, and of the enzyme-substrate complex, to induce a slower release of the hydrolysis product glucose. Conceivably, this can result in decreased systemic glucose concentration thus alleviating postprandial hyperglycemia and its associated complications.

### 2.6. Homology Modeling and Molecular Docking

**Figure 5:**
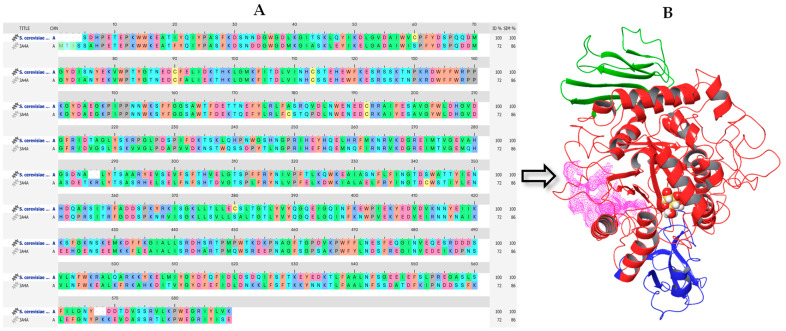
(**A**) Aligned protein sequences of *S. cerevisiae* yeast α-glucosidase and yeast isomaltase (PDB: 3A4A) showing the sequences similarity and identity. (**B**) α-Glucosidase homology model built from the 3A4A structure. The most probable allosteric site obtained from SiteMap calculations is depicted as a mesh surface while glucose bound at the active site is shown in CPK representation.

Having established from enzyme kinetic studies the allosteric mode of α-glucosidase inhibition for promising compounds **4b**, **4i**, **4j**, **4k**, **4l**, **12k**, and **12m**, we sought to gain an in silico insight into the compounds’ binding profiles. However, the 3D-crystal structure of α-glucosidase remained unsolved; thus, we constructed a 3D model of α-glucosidase using the homology modeling module in the Schrödinger molecular modelling suite [35] and the FASTA sequence of *Saccharomyces cerevisiae* yeast α-glucosidase (UniProt entry: P38138) [36]. A BLAST search retuned the oligo-1,6-glucosidase isomaltase structure (PDB: 3A4A) which was selected as a template for model building. Notably, the yeast isomaltase shared a high sequence identity and similarity, 72% and 86%, respectively, with α-glucosidase; hence their 3D structures were similar (Figure 5).

Due to the non-competitive character (i.e., allosteric inhibition) shown by the selected compounds, SiteMap calculations [37] were performed to identify other potential binding sites on α-glucosidase. The calculations produced five binding sites (Table S1) which were evaluated in terms of site score, drugability score (>1), and site volume (>225). Site two was chosen as the most probable allosteric site using these scoring functions (Figure 4). Subsequently, molecular docking calculations were performed at this site using the induced fit docking protocol [38], with Prime and Glide algorithms to account for the protein’s conformational changes due to ligand binding.

**Figure 6:**
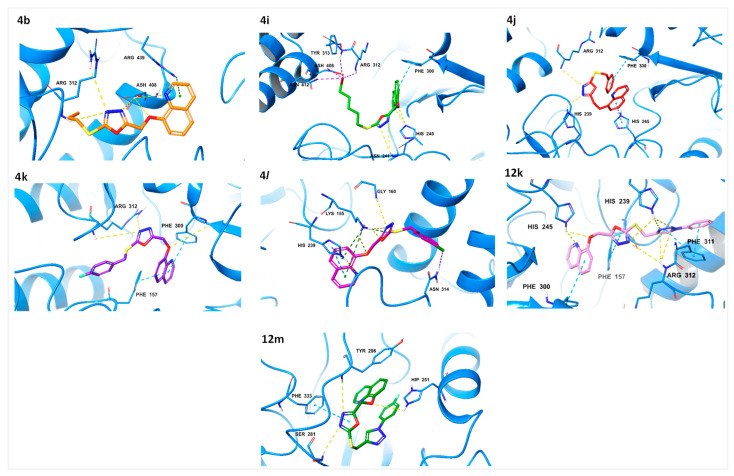
3D representation of the α-glucosidase complexes of promising compounds docked at the allosteric site. (H-bond yellow, π–π stacking blue, halogen purple, and π–cation green).

Analysis of the α-glucosidase complexes of examined compounds (Figure 6) revealed crucial protein–ligand interactions which substantiated the enzyme inhibition data. Foremost, the most potent compound **4i** found an excellent fit at the allosteric site, with the highest docking score of −6.938 in the series reflecting its potency (Table S2). The α-glucosidase complex was characterized by hydrogen bond interactions of the quinoline nitrogen and oxygens atoms with His245, as well as a similar interaction of the oxadiazole ring’s nitrogen with the amino side chain of Asn241. A face-to-face π–π stacking was present between the quinoline ring and Phe300. The ligand’s stability at the allosteric site was fostered by hydrophobic interactions with Lys155, Ser156, Phe157, Phe177, His239, Pro240, His245, Ala278, and Phe311 residues. More importantly, these interactions were anchored by a halogen bond interaction of the bromopentyl unit with Agr312, Tyr313, and Asn412, an interaction which conceivably accounts for the compound’s superior α-glucosidase inhibition.

The biochemical data indicated compound **12k** as the second most potent α-glucosidase inhibitor, and this was reflected in the ligand’s docking score of −6.757. Interestingly, the imidazole ring of His245 flipped 180° to form a hydrogen bond interaction with the ligand’s quinoline nitrogen, while the oxadiazole ring nitrogen and oxygen atoms formed hydrogen bond interactions with Arg439 and amino group of Arg312, respectively. Also, the triazole ring nitrogen formed a hydrogen bond with Agr312 even as the quinoline ring established a π–π stacking interaction with Phe157. Hydrophobic interactions with Phe157, Phe177, Thr215, Leu218, Hie245, Ala278, Phe300, and Arg312 residues stabilized the **12k**-α-glucosidase complex. Furthermore, the compound **4k**-α-glucosidase complex exhibited a lower docking score (−6.498) compared with **4i** & **12k** complexes, aligning with the ligands’ IC_50_ values. Compound **4k**’s interaction profile consisted of hydrogen bond interactions of **8**-**HQ** oxygen with Phe300, oxadiazole ring hydrogen bond donors spanning to the opposite ends of Arg312, one on the side chain amino group, and peptide bond NH. The aromatic rings of Phe157 and Phe300 aligned in plane to establish a π–π stacking interaction with the quinoline ring. 

For compound **4l**-α-glucosidase complex, the hydrogen bond interaction of the quinoline nitrogen was present with His239, while oxygen atoms of the ether linkage and oxadiazole ring interacted in a like manner with Lys155. The oxadiazole nitrogen also formed additional hydrogen bonds with Lys155 and Gly160, respectively. Moreover, the quinoline ring underwent a π–π stacking interaction with the His239 imidazole ring, while the oxadiazole ring experienced a π–cation stacking interaction with the protonated nitrogen of Lys155. The aromatic ring chlorine formed a halogen bond with Asn314, and complex stability was reinforced by hydrophobic interactions with Ly155, Phe157, Phe310, and Glu426. 

The lower inhibitory potencies of compounds **4j** and **12m** (relative to **4b**, **4i**, **4k**, **4l** and **12k**) were highlighted in their docking scores of −5.040 and −4.961, respectively. The interaction profiles of these two compounds were not so impressive as other examined ligands, corroborating the biochemical data. Although the oxadiazole nitrogen formed hydrogen bond interactions with His239 and Arg312 in compound **4j**, and Ser281 and Tyr286 in compound **12m**, the absence of the quinoline nitrogen’s hydrogen bond interaction with allosteric site residues seen in other compounds presumably accounts for the low affinity that was recorded. These results jointly rationalize the compounds’ behavior as non-competitive inhibitors i.e., they may reversibly bind at the allosteric site to trigger altered conformational dynamics at the enzyme’s active site, consequently reducing catalytic activity without competing with the native substrate’s binding. 

### 2.7. Molecular Dynamics Simulations

**Figure 7:**
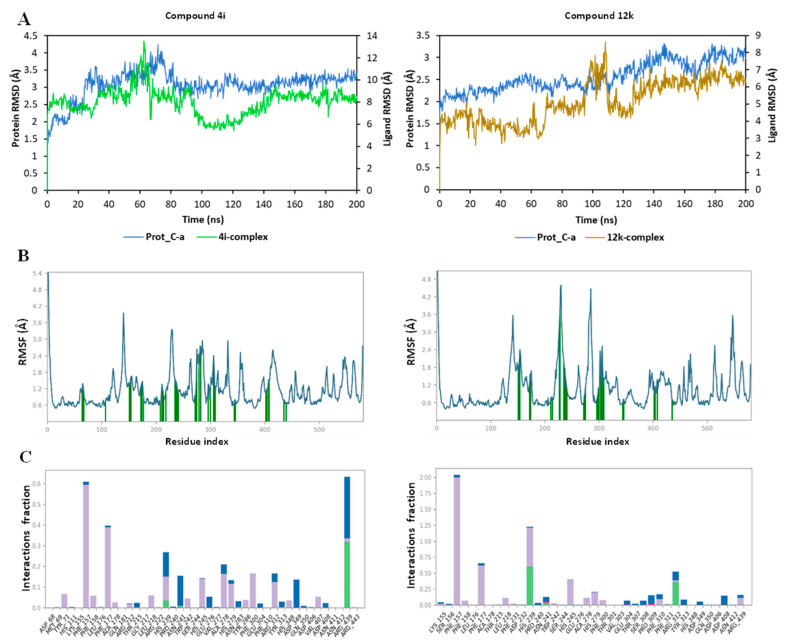
MD simulation trajectory analysis of compounds **4i** and **12k**. (**A**) RMSD and (**B**) RMSF plots. (**C**) Protein–ligand interactions over the 200 ns trajectory.

Molecular dynamics (MD) simulations of the two most promising compounds **4i** and **12k** were performed over 200 ns trajectory, to further evaluate the ligands’ stability at the allosteric site and the ligand-induced perturbation of protein conformation. Conformational stability was measured in terms of the root mean square deviations (RMSD) and root mean square fluctuations (RMSF) (Figure 7). RMSD indicates deviations of the protein–ligand complex from the reference structural conformation over the MD simulation trajectory, thus connoting the complex’s fluctuations. Smaller fluctuations suggest a stable conformation of the protein–ligand complex, and vice versa.

The RMSD of the protein’s C-α in the **4i** complex experienced increased dynamics at the beginning of the MD simulation, highlighting the perturbation effect consequent to **4i** binding at the allosteric site. RMSD for the protein and ligand reached equilibrium after 70 and 120 ns, respectively, with minimal fluctuations from the average values of 3.0 and 8.0 Å, thus indicating a stable complexation over the 200 ns simulation. In contrast, larger fluctuations persisted in the RMSD evolutions of the **12k**–α-glucosidase model system over the 200 ns trajectory. This highlights the system’s reduced stability and, consequently, the weaker α-glucosidase inhibition of **12k** compared to **4i**. Nonetheless, the **12k** model system equilibrated after 100 ns with no significant differences in fluctuation, hence the protein’s C-α and ligand’s RMSD converged at 2.4 and 6.5 Å, respectively. More importantly, the RMSD convergence of the protein’s C-α in both complexes was within the order of ≤3 Å showing no significantly large fluctuations, an indication of its excellent ability to maintain a stable residency, and ligands did not diffuse [39]. 

RMSF is significant for characterizing changes in protein residues’ flexibility within the chain. Herein, the ligands’ effect on the enzyme structure’s flexibility for each residue fluctuation was studied. Figure 7b shows that compound **4i** efficiently induced fluctuations in the protein’s residues, as seen by its highest RMSF value at ~3.8 Å with residues Ala144 and Lys233 fluctuating the most during the 200 ns simulation. Meanwhile, the highest RMSF in the compound **12k** complex occurred at 4.5 Å with residues Lys233 and Ala289. It is also inferable from Figure 7b that compound **4i** perturbed the receptor more than **12k** did. The number of green vertical bars denoting the residues interacting with the ligand were higher in the compound **4i**–α-glucosidase complex compared with **12k**. This further establishes the superior docking score, affinity for the target’s allosteric site, and consequently the α-glucosidase inhibitory potency of compound **4i** relative to **12k**.

The protein–ligand interactions were monitored over the simulation trajectory for contacts such as hydrogen bonds, hydrophobic, and water-mediated hydrogen bonding. Hydrogen bonds and hydrophobic contacts play significant roles at the binding site in ligands’ affinity and stability, respectively, to elicit a biological response; hence their occurrence is vital in MD simulations [40]. The analysis (Figure 7c) showed that hydrophobic interactions with α-glucosidase dominated in compound **4i** compared to **12k**; thus, reaffirming compound **4i**’s improved α-glucosidase inhibition.

## 3. Conclusions

The antidiabetic potential of the quinoline pharmacophore via α-glucosidase inhibition and its antioxidant effects has been detailed in this contribution. Biological evaluations of the synthesized compounds (**4a**–**t** and **12a**–**t**) suggested that the 1,3,4-oxadiazole core was more beneficial to the desired activity compared with 1,2,3-triazole. Compound **4i** bearing a bromopentyl sidechain and compound **12k** with an unsubstituted phenyl-1,2,3-triazole pendant emerged as the most promising α-glucosidase inhibitors overall. Enzyme kinetics further revealed a non-competitive mode of inhibition, thus classifying the present compounds as allosteric α-glucosidase inhibitors. This behavior is valuable to reducing systemic glucose levels while evading the limitations associated with the competitive type of target inhibition. Additionally, molecular docking and molecular dynamics simulations corroborated the compounds’ stability at the allosteric site, as evidenced by their strong interactions with the receptor. The results together illuminate the potential of the present structural template for developing new α-glucosidase inhibitors with improved efficacy for T2DM management.

## 4. Materials and Methods

All chemicals and reagents were purchased from Merck, Johannesburg, South Africa and were used without any purification. The reaction progress was monitored with thin layer chromatography (TLC) plates. Purification of the final products was achieved using column chromatography with silica gel (0.063–0.200 mm) at different gradients of EtOAc-hexane eluents. Melting points were determined with open-end capillary tubes in a Stuart melting point instrument (SMP-3) and were uncorrected. The ^1^H, ^13^C, and 2D-NMR spectra of all synthesized compounds were recorded on 400 and 600 MHz Bruker AvanceIII spectrometers. The chemical shifts were recorded in parts per million (ppm) with deuterated dimethyl sulfoxide-*d*_6_ (δ_H_ 2.50 and δ_C_ 39.50 ppm) and chloroform-*d* (δ_H_ 7.26 and δ_C_ 77.00 ppm), wherein, tetramethylsilane (TMS) at δ_H_ = 0 was used as an internal standard. The splitting pattern is abbreviated as singlet (s), doublet (d), triplet (t), quartet (q), multiplet (m), pentet (p), doublet of triplet (dt), doublet of doublet (dd), triplet of doublet (td) and doublet of quartet (dq), while coupling constants *J* are reported in Hertz (Hz). High-resolution mass spectra were recorded using a Water Micromass LCT Premier TOF-MS spectrometer. The general synthetic procedure for azides is outlined in the supporting information.

### 4.1. Synthesis of 5-[(Quinolin-8-yloxy)methyl]-1,3,4-oxadiazole-2-thiol (***3***)

Anhydrous potassium carbonate 138mmol (2.0 eq) was added to a solution of **8**-**HQ** (68.88 mmol) in DMF 40mL in a round bottom flask and stirred at room temperature (r.t). After a few minutes, ethyl chloroacetate was added with continued stirring for 6 h. The reaction was stopped after TLC showed a consumed **8**-**HQ**, then the mixture was poured into a slurry of crushed ice and the resulting precipitate compound **1** was collected via vacuum filtration as a beige solid at 88% yield. Compound **1** (14.00 g 60 mmol) was dissolved in absolute ethanol 80 mL, and 120 mmol, 2.0 eq of hydrazine hydrate was added slowly. The whole mixture was refluxed at 95 °C overnight. Thereafter, the solvent’s volume was reduced and the reaction flask was cooled in an ice bath to precipitate 2-(quinolin-8-yloxy)acetohydrazide **2**, which was filtered in vacuo while washing with cold diethyl ether to obtain 12.02 g of off-white solid (91% yield). Compound **2** (12.02 g, 55 mmol) was then poured into ethanolic potassium hydroxide and stirred at 80 °C for few a minutes before dropwise addition of carbon disulfide (221 mmol, 5eq). The mixture was refluxed at 80 °C for 12 h. Subsequently, the reaction mixture was evaporated to dryness and the crude product was treated with dilute HCl. The resulting precipitate was then poured into an ice slurry to yield a yellow solid. Recrystallization from ethanol afforded 5-((quinolin-8-yloxy)methyl)-1,3,4-oxadiazole-2-thiol as pale yellow solid at 80% yield.

*Ethyl 2-(Quinolin-8-yloxy)acetate (* **1** *)*

Beige solid; Chemical formula: C_13_H_13_NO_3_; Yield; 88%, Mol wt: 231.25 gmol^−1^.

**^1^H NMR** (400 MHz, Chloroform-*d*) δ 8.95 (dd, J = 4.2, 1.7 Hz, 1H, H-2), 8.15 (dd, J = 8.3, 1.8 Hz, 1H, H-4), 7.49–7.40 (m, 3H, H-3,5,6), 6.98 (dd, J = 6.7, 2.3 Hz, 1H, H-7), 4.96 (s, 2H, H-9), 4.29 (q, J = 7.1 Hz, 2H, H-11), 1.28 (t, J = 7.1 Hz, 3H, H-12).

**^13^C NMR** (101 MHz, Chloroform-*d*) δ 168.82 (C=O), 153.65 (C-8), 149.48 (C-2), 140.14 (C-4), 135.95 (C-8a), 129.58 (C-4a), 126.36 (C-6), 121.80 (C-3), 120.92 (C-5), 109.52 (C-7), 66.22 (O-CH_2_), 61.43 (C(O)O-CH_2_), 14.16 (CH_3_).

*2-(Quinolin-8-yloxy)acetohydrazide (* **2** *)*

Off-white solid; Chemical formula: C_11_H_11_N_3_O_2_, Yield; 91%, Mol wt: 217.22 gmol^−1^.

**^1^H NMR** (400 MHz, DMSO-*d*_6_) δ 9.45 (s, 1H, N*H*-1), 8.90 (dd, *J* = 4.1, 1.7 Hz, 1H, H-2), 8.35 (dd, *J* = 8.4, 1.7 Hz, 1H, H-4), 7.64–7.46 (m, 3H, H-3,5,6), 7.25 (dd, *J* = 7.6, 1.3 Hz, 1H, H-7), 4.74 (s, 2H, H-9), 4.37 (s, 2H, N*H_2_*).

**^13^C NMR** (101 MHz, DMSO) δ 166.80, 153.84, 149.29, 139.75, 136.02, 129.08, 126.72, 121.97, 120.90, 111.62, 68.24.

*5-[(Quinolin-8-yloxy)methyl]-1,3,4-oxadiazole-2-thiol (* **3** *)*

Dark yellow solid; Chemical formula: C_12_H_9_N_3_O_2_S, Yield; 80%, Mol wt: 259.28 g/mol

**^1^H NMR** (600 MHz, DMSO-*d_6_*) δ 14.77 (s, 1H, H-7′), 9.11 (dd, J = 4.9, 1.5 Hz, 1H, H-2), 8.97 (d, J = 8.4 Hz, 1H, H4), 7.98 (dd, J = 8.4, 5.0 Hz, 1H, H-3), 7.90 (d, J = 8.2 Hz, 1H, H-5), 7.81 (t, J = 8.1 Hz, 1H, H-6), 7.71 (d, J = 7.9 Hz, 1H, H-7), 5.61 (s, 2H, H-6′).

**^13^C NMR** (151 MHz, DMSO-*d_6_*) δ 178.65 (C-2′), 159.37 (C-5′), 149.81 (C-8), 147.28 (C-2), 143.50 (C-4), 133.23 (C-8a), 129.94 (C-4a), 129.26 (C-6), 123.19 (C-3), 122.13 (C-5), 114.22 (C-7), 61.27 (C-6′).

**HRMS**: (ESI^+^-MS, *m*/*z*) calcd for C_12_H_8_N_3_O_2_S (M − H)^+^: 258.0337; found: 258.0337.

### 4.2. General Synthetic Procedure for Quinoline-1,3,4-Oxadiazole Conjugates (***4a**–**t***)

To a solution of compound **3** (103.7 mg, 0.4 mmol) in DMF (3 mL), potassium carbonate (82.92 mg, 1.5 eq) was added and stirred until the reaction formed a paste in the round bottom flask. Then, 1.05 eq of appropriate benzyl bromides and aliphatic alkyl halides were added and stirring continued at r.t. for 1–2 h. Thereafter, the flask’s contents were poured in a slurry of ice and extracted with ethyl acetate. The crude product was purified with column chromatography using EtOAc-hexane eluents to obtain quinoline-1,3,4-oxadiazole conjugates in moderate to quantitative yields.

*2-(Ethylthio)-5-[(quinolin-8-yloxy)methyl]-1,3,4-oxadiazole (* **4a** *)*

Brown oil; Chemical formula: C_14_H_13_N_3_O_2_S, Yield; 53%, Mol wt: 287.34 gmol^−1^, M.p: 80.7–83.1 °C.

**^1^H NMR** (600 MHz, DMSO-*d_6_*) δ 8.88 (dd, J = 4.1, 1.8 Hz, 1H, H-2), 8.35 (dd, J = 8.3, 1.8 Hz, 1H, H-4), 7.62 (dd, J = 8.2, 1.2 Hz, 1H, H-5), 7.57 (dd, J = 8.3, 4.1 Hz, 1H, H-3), 7.54 (t, J = 7.9 Hz, 1H, H-6), 7.40 (dd, J = 7.7, 1.2 Hz, 1H, H-7), 5.63 (s, 2H H-6′), 3.26 (q, J = 7.3 Hz, 2H, H-7′), 1.37 (t, J = 7.3 Hz, 3H, H-8′).

**^13^C NMR** (151 MHz, DMSO-*d_6_*) δ 164.90, 163.58, 152.93, 149.39, 139.73, 135.93, 129.15, 126.53, 121.99, 121.49, 111.62, 60.56, 26.61, 14.70.

**HRMS**: (ESI^+^-MS, *m*/*z*) calcd for C_14_H_13_N_3_O_2_S (M + Na)^+^: 310.0626; found: 310.0631.

*2-(Propylthio)-5-[(quinolin-8-yloxy)methyl]-1,3,4-oxadiazole (* **4b** *)*

Brown oil; Chemical formula: C_15_H_15_N_3_O_2_S, Yield; 100%, Mol wt: 301.36 gmol^−1^.

**^1^H NMR** (600 MHz, DMSO-*d_6_*) δ 8.88 (dd, J = 4.1, 1.7 Hz, 1H, H-2), 8.35 (dd, J = 8.3, 1.7 Hz, 1H, H-4), 7.62 (dd, J = 8.3, 1.2 Hz, 1H, H-5), 7.57 (dd, J = 8.3, 4.1 Hz, 1H, H-3), 7.54 (t, J = 8.0 Hz, 1H, H-6), 7.39 (dd, J = 7.7, 1.2 Hz, 1H, H-7), 5.61 (s, 2H, H-6′), 3.23 (t, J = 7.1 Hz, 2H, H-7′), 1.73 (m, J = 7.3 Hz, 2H, H-8′), 0.96 (t, J = 7.4 Hz, 3H, H-9′).

**^13^C NMR** (151 MHz, DMSO-d6) δ 165.49 (C-2′), 164.08 (C-5′), 153.42 (C-8), 149.88 (C-2), 140.24 (C-8a), 136.43 (C-4), 129.65 (C-4a), 127.02 (C-6), 122.49 (C-3), 122.01 (C-5), 112.20 (C-7), 61.08 (C-6′), 34.38 (C-7′), 22.79 (C-8′), 13.19 (C-9′).

**HRMS**: (ESI^+^-MS, *m*/*z*) calcd for C_15_H_15_N_3_O_2_S (M + Na)^+^: 324.0783; found: 324.0786.

*2-(Butylthio)-5-[(quinolin-8-yloxy)methyl]-1,3,4-oxadiazole* (**4c**)

Yellow solid; Chemical formula: C_16_H_17_N_3_O_2_S, Yield; 53%, Mol wt: 315.39 gmol^−1^, M.p: 88.3–93.5 °C. 

**^1^H NMR** (600 MHz, DMSO-*d_6_*) δ 8.88 (dd, J = 4.2, 1.8 Hz, 1H, H-2), 8.36 (dd, J = 8.3, 1.8 Hz, 1H, H-4), 7.63 (d, J = 8.4 Hz, 1H, H-5), 7.58 (dd, J = 8.3, 4.1 Hz, 1H, H-3), 7.54 (t, J = 8.0 Hz, 1H, H-6), 7.39 (dd, J = 7.8, 1.2 Hz, 1H, H-7), 5.62 (s, 2H H-6′), 3.25 (t, J = 7.3 Hz, 2H, H-7′), 1.74–1.65 (m, 2H, H-8′), 1.39 (hept, J = 7.7 Hz, 2H, H-9′), 0.87 (t, J = 7.4 Hz, 3H, H-10′). 

**^13^C NMR** (151 MHz, DMSO-*d_6_*) δ 165.47 (C-2′), 164.10 (C-5′), 153.44 (C-8), 149.88 (C-2), 140.28 (C-8a), 136.43 (C-4), 129.66 (C-4a), 127.02 (C-6), 122.49 (C-3), 122.03 (C-5), 112.28 (C-7), 61.13 (C-6′), 32.23 (C-7′), 31.42 (C-8′), 21.44 (C-9′), 13.72 (C-10′).

**HRMS**: (ESI^+^-MS, *m*/*z*) calcd for C_16_H_17_N_3_O_2_S (M + Na)^+^: 338.0939; found: 338.0943.

*2-(Pentylthio)-5-[(quinolin-8-yloxy)methyl]-1,3,4-oxadiazole* (**4d**)

Brown solid; Chemical formula: C_17_H_19_N_3_O_2_S, Yield; 77%, Mol wt: 329.42 gmol^−1^, M.p: 84.6–90.1 °C.

**^1^H NMR** (600 MHz, DMSO-*d_6_*) δ 8.88 (dd, J = 4.1, 1.7 Hz, 1H, H-2′), 8.36 (dd, J = 8.3, 1.7 Hz, 1H, H-4′), 7.63 (dd, J = 8.2, 1.2 Hz, 1H, H-5), 7.58 (dd, J = 8.3, 4.1 Hz, 1H, H-3), 7.54 (t, J = 7.9 Hz, 1H, H-6), 7.38 (dd, J = 7.7, 1.2 Hz, 1H, H-7), 5.62 (s, 2H, H-6′), 3.24 (t, J = 7.3 Hz, 2H, H-7′), 1.71 (p, J = 7.3 Hz, 2H, H-8′), 1.38–1.30 (m, 2H, H-9′), 1.27 (m, J = 14.6, 7.0 Hz, 2H, H-10′), 0.85 (t, J = 7.2 Hz, 3H, H-11′).

**^13^C NMR** (151 MHz, DMSO-*d_6_*) δ 165.49 (C-2′), 164.09 (C-5′), 153.42 (C-8), 149.88 (C-2), 140.25 (C-8a), 136.43 (C-4), 129.66 (C-4a), 127.02 (C-6), 122.49 (C-3), 122.01 (C-5), 112.22 (C-7), 61.07 (C-6′), 32.49 (C-7′), 30.40 (C-8′), 29.04 (C-9′), 21.94 (C-10′),14.18 (C-11′).

**HRMS**: (ESI^+^-MS, *m*/*z*) calcd for C_17_H_19_N_3_O_2_S (M + Na)^+^: 352.1096; found: 352.1101.

*2-(Hexylthio)-5-[(quinolin-8-yloxy)methyl]-1,3,4-oxadiazole* (**4e**)

Brown oil; Chemical formula: C_18_H_21_N_3_O_2_S, Yield; 60%, Mol wt: 343.44 gmol^−1^.

**^1^H NMR** (600 MHz, DMSO-*d_6_*) δ 8.87 (dd, J = 4.1, 1.7 Hz, 1H, H-2), 8.36 (dd, J = 8.3, 1.7 Hz, 1H, H-4), 7.62 (dd, J = 8.3, 1.2 Hz, 1H, H-5), 7.57 (dd, J = 8.3, 4.1 Hz, 1H, H-3), 7.54 (t, J = 7.9 Hz, 1H, H-6), 7.38 (dd, J = 7.7, 1.2 Hz, 1H, H-7), 5.61 (s, 2H, H-6′), 3.24 (t, J = 7.3 Hz, 2H, H-7′), 1.70 (p, J = 7.5 Hz, 2H, H-9′), 1.36 (q, J = 7.1, 6.6 Hz, 2H, H-11′), 1.25 (m, J = 7.5, 4.5 Hz, 4H, H-8′,10′), 0.84 (t, *J* = 7.0 Hz, 3H, H-12′).

**^13^C NMR** (151 MHz, DMSO-*d_6_*) δ 165.49 (C-2′), 164.08 (C-5′), 153.41 (C-8), 149.87 (C-2), 140.23 (C-8a), 136.43 (C-4), 129.65 (C-4a), 127.02 (C-6), 122.49 (C-3), 121.99 (C-5), 112.14 (C-7), 61.05 (C-6′), 32.51 (C-7′), 31.01 (C-8′), 29.32 (C-9′), 27.90 (C-11′), 22.35 (C-10′), 14.26 (C-12′).

**HRMS**: (ESI^+^-MS, *m*/*z*) calcd for C_18_H_21_N_3_O_2_S (M + Na)^+^: 366.1252; found: 366.1252.

*2-(Allylthio)-5-[(quinolin-8-yloxy)methyl]-1,3,4-oxadiazole* (**4f**)

Yellow oil; Chemical formula: C_15_H_13_N_3_O_2_S, Yield; 52%, Mol wt: 299.35 gmol^−1^.

**^1^H NMR** (400 MHz, DMSO-*d_6_*) δ 8.88 (dd, J = 4.1, 1.8 Hz, 1H, H-2), 8.36 (dd, J = 8.3, 1.7 Hz, 1H, H-4), 7.63 (dd, J = 8.3, 1.2 Hz, 1H, H-5), 7.58 (dd, J = 8.3, 4.2 Hz, 1H, H-3), 7.54 (t, J = 7.9 Hz, 1H, H-6), 7.38 (dd, J = 7.7, 1.3 Hz, 1H, H-7), 5.95 (m, J = 17.0, 10.0, 7.0 Hz, 1H, H-8′), 5.63 (s, 2H, H-6′), 5.30 (m, J = 17.0, 1.4 Hz, 2H, H-9b), 5.12 (m, J = 10.0, 1.7, 0.9 Hz, 2H, H-9a), 3.92 (dd, J = 6.9, 1.1 Hz, 2H, H-7′).

**^13^C NMR** (101 MHz, DMSO-*d_6_*_)_ δ 164.75 (C-2′), 164.31 (C-5′), 153.37 (C-8), 149.90 (C-2), 140.16 (C-8a), 136.45 (C-4), 132.87 (C-8′), 129.63 (C-4a), 127.05 (C-6), 122.53 (C-3), 121.95 (C-5), 119.93 (C-9), 111.99 (C-7), 60.94 (C-6′), 35.04 (C-7′).

**HRMS**: (ESI^+^-MS, *m*/*z*) calcd for C_15_H_13_N_3_O_2_S (M + Na)^+^: 322.0626; found: 322.0636. 

*2-[(3-Methylbut-2-en-1-yl)thio]-5-[(quinolin-8-yloxy)methyl]-1,3,4-oxadiazole* (**4g**)

Yellow oil; Chemical formula: C_17_H_17_N_3_O_2_S, Yield; 30%, Mol wt: 327.40 gmol^−1^.

**^1^H NMR** (400 MHz, DMSO-*d_6_*) δ 8.88 (dd, J = 4.2, 1.8 Hz, 1H, H-2), 8.36 (dd, J = 8.3, 1.7 Hz, 1H, H-4), 7.63 (dd, J = 8.2, 1.2 Hz, 1H, H-5), 7.60–7.51 (m, 2H, H-3,6), 7.38 (dd, J = 7.7, 1.3 Hz, 1H, H-7), 5.61 (s, 2H, H-6′), 5.35 (dd, J = 7.8, 1.5 Hz, 1H, H-8′), 3.91 (d, J = 7.8 Hz, 2H, H-7′), 1.66 (s, 6H, H-10′,11′).

**^13^C NMR** (101 MHz, DMSO-*d_6_*) δ 164.60 (C-2′), 164.11 (C-5′), 153.29 (C-8), 149.87 (C-2), 140.04 (C-8a), 136.60 (C-4), 129.64 (C-4a), 127.11 (C-6), 122.54 (C-3), 122.03 (C-5), 112.21 (C-7), 79.26 (C-8′), 75.51 (C-9′), 61.05 (C-6′), 21.19 (C-7′).

**HRMS**: (ESI^+^-MS, *m*/*z*) calcd for C_17_H_17_N_3_O_2_S (M + Na)^+^: 350.0939; found: 350.0950.

*2-[(2-Bromoethyl)thio]-5-[(quinolin-8-yloxy)methyl]-1,3,4-oxadiazole* (**4h**)

Yellow oil; Chemical formula: C_14_H_12_BrN_3_O_2_S, Yield; 54%, Mol wt: 326.33 gmol^−1^.

**^1^H NMR** (600 MHz, DMSO-*d_6_*) δ 8.88 (dd, J = 4.2, 1.7 Hz, 1H, H-2), 8.36 (dd, J = 8.3, 1.7 Hz, 1H, H-4), 7.63 (dd, J = 8.3, 1.2 Hz, 1H, H-5), 7.58 (dd, J = 8.3, 4.1 Hz, 1H, H-3), 7.55 (t, J = 7.9 Hz, 1H, H-6), 7.40 (dd, J = 7.7, 1.2 Hz, 1H, H-7), 5.62 (s, 2H, H-6′), 3.83 (t, J = 7.3 Hz, 2H, H-7′), 3.72 (t, J = 7.1 Hz, 2H, H-8′).

**^13^C NMR** (151 MHz, DMSO-*d_6_*) δ 164.59 (C-2′), 164.38 (C-5′), 153.43 (C-8), 149.90 (C-2), 140.25 (C-8a), 136.45 (C-4), 129.65 (C-4a), 127.04 (C-6), 122.50 (C-3), 122.05 (C-5), 112.30 (C-7), 61.13 (C-6′), 34.36 (C-7′), 31.19 (C-8′).

**HRMS**: (ESI^+^-MS, *m*/*z*) calcd for C_14_H_12_BrN_3_O_2_S (M + Na)^+^: 387.9731; found: 387.9747.

*2-[(5-Bromopentyl)thio]-5-[(quinolin-8-yloxy)methyl]-1,3,4-oxadiazole* (**4i**)

Yellow oil; Chemical formula: C_17_H_18_BrN_3_O_2_S, Yield; 61%, Mol wt: 408.31 gmol^−1^.

**^1^H NMR** (600 MHz, DMSO- *d_6_*) δ 8.89 (dd, *J* = 4.2, 1.6 Hz, 1H, H-2), 8.42 (dd, *J* = 8.3, 1.7 Hz, 1H, H-4), 7.65 (d, *J* = 8.2 Hz, 1H, H-5), 7.62 (dd, *J* = 8.3, 4.2 Hz, 1H, H-3), 7.57 (t, *J* = 8.0 Hz, 1H, H-6), 7.42 (d, *J* = 7.8 Hz, 1H, H-7), 5.63 (s, 2H, H-6′), 3.51 (t, *J* = 6.7 Hz, 2H, H-11′), 3.26 (t, *J* = 7.3 Hz, 2H, H-7′), 1.81 (p, *J* = 6.9 Hz, 2H, H-9′), 1.74 (p, *J* = 7.4 Hz, 2H, H-8′), 1.49 (p, *J* = 7.7 Hz, 2H, H-10′).

**^13^C NMR** (151 MHz, DMSO-*d*_6_) δ 165.48 (C-2′), 164.02 (C-5′), 152.99 (C-8), 149.58 (C-2), 139.88 (C-8a), 137.27 (C-4), 129.68 (C-4a), 127.30 (C-6), 122.59 (C-3), 122.01 (C-5), 112.40 (C-7), 61.07 (C-6′), 35.26 (C-11′), 32.30 (C-7′), 31.97 (C-9′), 28.46 (C-8′), 26.82 (C-10′).

**HRMS**: (ESI^+^-MS, *m*/*z*) calcd for C_17_H_18_BrN_3_O_2_S (M + Na)^+^: 430.0201; found: 430.0212.

*2-(Benzylthio)-5-[(quinolin-8-yloxy)methyl]-1,3,4-oxadiazole* (**4j**)

Light brown solid; Chemical formula: C_19_H_15_N_3_O_2_S, Yield; 50%, Mol wt: 349.41 gmol^−1^, M.p: 75.3–79.8 °C.

**^1^H NMR** (600 MHz, DMSO-*d_6_*) δ 8.87 (dd, J = 4.1, 1.8 Hz, 1H, H-2), 8.36 (dd, J = 8.3, 1.8 Hz, 1H, H-4), 7.63 (dd, J = 8.2, 1.2 Hz, 1H, H-5), 7.58 (dd, J = 8.3, 4.1 Hz, 1H, H-3), 7.54 (t, J = 8.0 Hz, 1H, H-6), 7.42 (dd, J = 7.6, 1.7 Hz, 2H, H-9′, 13′), 7.37 (dd, J = 7.8, 1.2 Hz, 1H, H-7), 7.33–7.24 (m, 3H, H-10′, 11′,12′), 5.62 (s, 2H, H-6′), 4.52 (s, 2H, H-7′).

**^13^C NMR** (151 MHz, DMSO-*d_6_*) δ 164.87 (C-2′), 164.31 (C-5′), 153.38 (C-8), 149.90 (C-2), 140.21 (C-8a), 136.82 (C-8′), 136.44 (C-4), 129.65 (C-4a), 129.43 (C-9′,13′), 129.01 (C-10′,12′), 128.23 (C-11′), 127.04 (C-6), 122.52 (C-3), 121.98 (C-5), 112.05 (C-7), 60.98 (C-6′), 36.34 (C-7′).

**HRMS**: (ESI^+^-MS, *m*/*z*) calcd for C_19_H_15_N_3_O_2_S (M + Na)^+^: 372.0783; found: 372.0790.

*2-[(4-Fluorobenzyl)thio]-5-[(quinolin-8-yloxy)methyl]-1,3,4-oxadiazole* (**4k**)

Yellow crystals; Chemical formula: C_19_H_16_FN_3_O_2_S, Yield; 32%, Mol wt: 367.40 gmol^−1^, M.p: 106.9–109.4 °C.

**^1^H NMR** (600 MHz, DMSO-*d_6_*) δ 8.87 (dd, J = 4.1, 1.7 Hz, 1H, H-2), 8.36 (dd, J = 8.3, 1.7 Hz, 1H, H-4), 7.63 (dd, J = 8.3, 1.2 Hz, 1H, H-5), 7.58 (dd, J = 8.3, 4.1 Hz, 1H, H-3), 7.54 (t, J = 7.9 Hz, 1H, H-6), 7.47 (dd, J = 8.5, 5.6 Hz, 2H, H-9′,13′), 7.36 (dd, J = 7.7, 1.2 Hz, 1H, H-7), 7.09 (d, J = 8.9 Hz, 2H, H-10′,12′), 5.61 (s, 2H, H-6′), 4.51 (s, 2H, H-7′).

**^13^C NMR** (151 MHz, DMSO-*d_6_*) δ 164.82 (C-2′), 164.32 (C-5′), 162.05 (d, J = 244.2 Hz, C-11′), 153.33 (C-8), 149.90 (C-2), 140.12 (C-8a), 136.46 (C-4), 133.22 (d, J = 3.0 Hz, C-8′), 131.58 (d, J = 8.3 Hz, C-9′,13′), 129.63 (4a), 127.05 (C-6), 122.55 (C-3), 121.92 (C-5), 115.80 (d, J = 21.5 Hz, C-10′,12′), 111.82 (C-7), 60.85 (C-6′), 35.46 (C-7′).

**HRMS**: (ESI^+^-MS, *m*/*z*) calcd for C_19_H_14_FN_3_O_2_S (M + Na)^+^: 390.0688; found: 390.0696.

*2-[(4-Chlorobenzyl)thio]-5-[(quinolin-8-yloxy)methyl]-1,3,4-oxadiazole* (**4l**)

Cream crystals; Chemical formula: C_19_H_14_ClN_3_O_2_S, Yield; 85%, Mol wt: 383.85 gmol^−1^, M.p: 101.4–104.2 °C.

**^1^H NMR** (600 MHz, DMSO-*d_6_*) δ 8.87 (dd, J = 4.1, 1.7 Hz, 1H, H-2), 8.36 (dd, J = 8.3, 1.8 Hz, 1H, H-4), 7.63 (dd, J = 8.3, 1.2 Hz, 1H, H-5), 7.58 (dd, J = 8.3, 4.1 Hz, 1H, H-3), 7.53 (t, J = 8.0 Hz, 1H, H-6), 7.45 (d, J = 8.4 Hz, 2H, H-9′,13′), 7.36 (dd, J = 7.8, 1.2 Hz, 1H, H-7), 7.32 (d, J = 8.4 Hz, 2H, H-10′,12′), 5.61 (s, 2H, H-6′), 4.51 (s, 2H, H-7′).

**^13^C NMR** (151 MHz, DMSO-*d6*) δ 164.71 (C-2′), 164.37 (C-5′), 153.37 (C-8), 149.89 (C-2), 140.19 (C-8a), 136.45 (C-4), 136.13 (C-8′), 132.86 (C-11′), 131.32 (C-9′,13′), 129.65 (C-4a), 128.94 (C-10′,12′), 127.04 (C-6), 122.53 (C-3), 121.98 (C-5), 112.00 (C-7), 60.96 (C-6′), 35.52 (C-7′).

**HRMS**: (ESI^+^-MS, *m*/*z*) calcd for C_19_H_14_ClN_3_O_2_S (M + Na)^+^: 406.0393; found: 406.0407.

*2-[(4-Bromobenzyl)thio]-5-[(quinolin-8-yloxy)methyl]-1,3,4-oxadiazole* (**4m**)

Yellow solid; Chemical formula: C_19_H_14_BrN_3_O_2_S, Yield; 27%, Mol wt: 428.30 gmol^−1^, M.p: 120.9–124.1 °C.

**^1^H NMR** (600 MHz, DMSO-d6) δ 8.87 (dd, J = 4.1, 1.7 Hz, 1H, H-2), 8.36 (dd, J = 8.3, 1.7 Hz, 1H, H-4), 7.63 (dd, J = 8.3, 1.2 Hz, 1H, H-5), 7.58 (dd, J = 8.3, 4.1 Hz, 1H, H-3), 7.53 (t, J = 8.0 Hz, 1H, H-6), 7.46 (d, J = 8.4 Hz, 2H, H-10′,12′), 7.38 (d, J = 8.4 Hz, 2H, H-9′,13′), 7.36 (dd, J = 7.8, 1.2 Hz, 1H, H-7), 5.61 (s, 2H, H-6′), 4.49 (s, 2H, H-7′).

**^13^C NMR** (151 MHz, DMSO-*d_6_*) δ 164.69 (C-2′), 164.38 (C-5′), 153.38 (C-8), 149.89 (C-2), 140.20 (C-8a), 136.57 (C-8′), 136.45 (C-4), 131.87 (C-10′,12′), 131.64 (C-9′,13′), 129.65 (C-4a), 127.04 (C-6), 122.52 (C-3), 121.98 (C-5), 121.40 (C-11′), 112.02 (C-7), 60.97 (C-6′), 35.57 (C-7′).

**HRMS**: (ESI^+^-MS, *m*/*z*) calcd for C_19_H_14_BrN_3_O_2_S (M + Na)^+^: 449.9888; found: 449.9895.

*2-[(4-Nitrobenzyl)thio]-5-[(quinolin-8-yloxy)methyl]-1,3,4-oxadiazole* (**4n**)

Yellow crystals; Chemical formula: C_19_H_14_N_4_O_4_S, Yield; 104%, Mol wt: 394.40 gmol^−1^, M.p: 156.6–160.1 °C.

**^1^H NMR** (600 MHz, DMSO-*d_6_*) δ 8.88 (dd, J = 4.2, 1.8 Hz, 1H, H-2), 8.35 (dd, J = 8.3, 1.8 Hz, 1H, H-4), 8.13 (dd, J = 8.7, 6.8 Hz, 2H, H-10′,12′), 7.71 (dd, J = 8.7, 6.7 Hz, 2H, H-9′,13′), 7.63 (dd, J = 8.3, 1.2 Hz, 1H, H-5), 7.57 (dd, J = 8.3, 4.1 Hz, 1H, H-3), 7.53 (t, J = 8.0 Hz, 1H, H-6), 7.37 (dd, J = 7.8, 1.2 Hz, 1H, H-7), 5.62 (s, 2H, H-6′), 4.65 (s, 2H, H-7′).

**^13^C NMR** (151 MHz, DMSO-*d_6_*) δ 163.98 (C-2′), 163.97 (C-5′), 152.88 (C-8), 149.38 (C-2), 146.85 (C-11′), 144.68 (C-8′), 139.67 (C-8a), 135.95 (C-4), 130.25 (C-9′,13′), 129.14 (4a), 126.52 (C-6), 123.53 (C-10′,12′), 122.02 (C-3), 121.45 (C-5), 111.41 (C-7), 60.43 (C-6′), 34.86 (C-7′).

**HRMS**: (ESI^+^-MS, *m*/*z*) calcd for C_19_H_14_N_4_O_4_S (M + Na)^+^: 417.0633; found: 417.0645. 

*2-[(3-Fluorobenzyl)thio]-5-[(quinolin-8-yloxy)methyl]-1,3,4-oxadiazole* (**4o**)

Yellow oil; Chemical formula: C_19_H_14_FN_3_O_2_S, Yield; 63%, Mol wt: 367.40 gmol^−1^.

**^1^H NMR** (600 MHz, DMSO-*d_6_*) δ 8.91 (d, J = 4.2 Hz, 1H, H-2), 8.48 (d, J = 8.2 Hz, 1H, H-4), 7.68 (d, J = 8.2 Hz, 1H, H-5), 7.65 (dd, J = 8.3, 4.3 Hz, 1H, H-3), 7.59 (t, J = 8.0 Hz, 1H, H-6), 7.43 (d, J = 7.7 Hz, 1H, H-7), 7.33 (q, J = 7.5 Hz, 1H, H-10′), 7.31–7.24 (m, 2H, H-9′,13′), 7.10 (td, J = 8.6, 2.6 Hz, 1H, H-11′), 5.64 (s, 2H, H-6′), 4.54 (s, 2H, H-7′).

**^13^C NMR** (151 MHz, DMSO-*d_6_*) δ 164.79 (d, J = 3.9 Hz, C-2′), 164.26 (d, J = 3.4 Hz, C-5′), 162.42 (d, J = 244.1 Hz, C-12′), 152.59 (C-8), 149.30 (C-2), 139.79 (d, J = 7.9 Hz, C-8a), 137.98 (C-4), 130.94 (d, J = 8.4 Hz, C-10′), 129.71 (d, J = 2.4 Hz, C-4a), 127.51 (d, J = 3.9 Hz, C-6), 125.57 (t, J = 3.2 Hz, C-9′), 122.65 (d, *J* = 3.5 Hz, C-3), 122.04 (d, J = 7.7 Hz, C-5), 116.21 (dd, J = 21.9, 2.9 Hz, C-13′), 115.09 (dd, J = 21.0, 1.4 Hz, C-11′), 122.65 (d, *J* = 3.5 Hz, C-7), 61.13 (C-6′), 35.62 (C-7′).

**HRMS**: (ESI^+^-MS, *m*/*z*) calcd for C_19_H_14_FN_3_O_2_S (M + Na)^+^: 390.0688; found: 390.0697. 

*2-[(3-Chlorobenzyl)thio]-5-[(quinolin-8-yloxy)methyl]-1,3,4-oxadiazole* (**4p**)

Yellow oil; Chemical formula: C_19_H_14_ClN_3_O_2_S, Yield; 27%, Mol wt: 383.85 gmol^−1^.

**^1^H NMR** (600 MHz, DMSO-*d_6_*) δ 8.87 (dd, J = 4.2, 1.8 Hz, 1H, H-2), 8.36 (dd, J = 8.3, 1.8 Hz, 1H, H-4), 7.62 (d, J = 8.2 Hz, 1H, H-5), 7.57 (dd, J = 8.3, 4.1 Hz, 1H, H-3), 7.54 (s, 1H, H-13′), 7.52 (d, J = 8.0 Hz, 1H, H-6), 7.39 (dd, J = 7.1, 1.7 Hz, 1H, H-9′), 7.36 (dd, J = 7.7, 1.2 Hz, 1H, H-7), 7.34–7.26 (m, 2H, H-10′,11′), 5.60 (s, 2H, H-6′), 4.53 (s, 2H, H-7′).

**^13^C NMR** (151 MHz, DMSO-*d_6_*) δ 164.72 (C-2′), 164.40 (C-5′), 153.38 (C-8), 149.90 (C-2), 140.20 (C-8a), 139.59 (C-8′), 136.45 (C-4), 133.47 (C-12′), 130.82 (C-10′), 129.64 (C-4a), 129.29 (C-13′), 128.19 (C-9′), 128.14 (C-11′), 127.03 (C-6), 122.51 (C-3), 122.02 (C-5), 112.11 (C-7), 61.04 (C-6′), 35.51 (C-7′).

**HRMS**: (ESI^+^-MS, *m*/*z*) calcd for C_19_H_14_ClN_3_O_2_S (M + Na)^+^: 406.0393; found: 406.0405. 

*2-[(3-Bromobenzyl)thio]-5-[(quinolin-8-yloxy)methyl]-1,3,4-oxadiazole* (**4q**)

Yellow oil; Chemical formula: C_19_H_14_BrN_3_O_2_S, Yield; 51%, Mol wt: 428.30 gmol^−1^.

**^1^H NMR** (600 MHz, DMSO-*d_6_*) δ 8.90 (d, J = 4.0 Hz, 1H, H-2), 8.45 (d, J = 8.2 Hz, 1H, H-4), 7.69 (s, 1H, H-13′), 7.67 (d, J = 8.2 Hz, 1H, H-5), 7.64 (dd, J = 8.3, 4.2 Hz, 1H, H-3), 7.58 (t, J = 8.0 Hz, 1H, H-6), 7.47 (dd, J = 8.0, 1.9 Hz, 1H, H-11′), 7.44 (d, J = 7.7 Hz, 1H, H-9′), 7.42 (d, J = 7.7 Hz, 1H, H-7), 7.25 (t, J = 7.8 Hz, 1H, H-10′), 5.63 (s, 2H, H-6′), 4.52 (s, 2H, H-7′).

**^13^C NMR** (151 MHz, DMSO-*d_6_*) δ 164.76 (C-2′), 164.37 (C-5′), 153.30 (C-8), 149.47 (C-2), 139.86 (C-8a), 139.82 (C-8′), 137.58 (C-4), 137.47 (C-10′), 132.15 (C-13′), 131.08 (C-11′), 129.73 (C-4a), 128.50 (C-9′), 127.36 (C-6), 122.58 (C-3), 122.07 (C-5), 122.02 (C-12′), 112.95 (C-7), 61.38 (C-6′), 35.61 (C-7′).

**HRMS**: (ESI^+^-MS, *m*/*z*) calcd for C_19_H_14_BrN_3_O_2_S (M + Na)^+^: 449.9888; found: 449.9903.

*2-[(2,4-Difluorobenzyl)thio]-5-[(quinolin-8-yloxy)methyl]-1,3,4-oxadiazole* (**4r**)

Cream solid; Chemical formula: C_19_H_13_F_2_N_3_O_2_S, Yield; 29%, Mol wt: 385.39 gmol^−1^, M.p: 98.7–105.2 °C.

**^1^H NMR** (600 MHz, DMSO-*d_6_*) δ 8.87 (dd, J = 4.1, 1.7 Hz, 1H, H-2), 8.36 (dd, J = 8.3, 1.8 Hz, 1H, H-4), 7.63 (dd, J = 8.2, 1.2 Hz, 1H, H-5), 7.61–7.51 (m, 3H, H-3.9′,6), 7.37 (dd, J = 7.7, 1.2 Hz, 1H, H-7), 7.24 (ddd, J = 10.6, 9.2, 2.6 Hz, 1H, H-12′), 6.99 (td, J = 8.5, 2.4 Hz, 1H, H-10′), 5.61 (s, 2H, H-6′), 4.53 (s, 2H, H-7′).

**^13^C NMR** (151 MHz, DMSO-*d_6_*) δ 164.58 (d, J = 7.5 Hz, C-5′), 164.31 (C-2′), 161.97 (d, J = 7.8 Hz, C-11′), 161.10 (d, J = 26.8 Hz, C-8′), 153.45 (d, J = 13.5 Hz, C-8), 149.89 (C-2), 140.32 (d, J = 25.4 Hz, C-8a), 136.43 (d, J = 3.8 Hz, C-4), 132.96 (dd, J = 9.9, 5.2 Hz, C-9′), 129.68 (d, J = 7.7 Hz, C-4a), 127.01 (d, J = 5.3 Hz, C-6), 122.48 (d, J = 8.6 Hz, C-3), 122.09 (d, J = 20.1 Hz, C-5), 120.44–120.17 (m, C-13′), 112.60 (C-7), 112.05 (dd, J = 21.5, 3.6 Hz, C-10′), 104.57 (dd, J = 26.1, 25.4 Hz, C-12′), 61.20 (d, J = 40.4 Hz, C-6′), 29.97–29.67 (m, C-7′).

**HRMS**: (ESI^+^-MS, *m*/*z*) calcd for C_19_H_13_F_2_N_3_O_2_S (M + Na)^+^: 408.0594; found: 408.0604. 

*2-[(3,4-Difluorobenzyl)thio]-5-[(quinolin-8-yloxy)methyl]-1,3,4-oxadiazole* (**4s**)

Cream solid; Chemical formula: C_19_H_13_F_2_N_3_O_2_S, Yield; 62%, Mol wt: 385.39 gmol^−1^, M.p: 98.8–101.2 °C.

**^1^H NMR** (600 MHz, DMSO-*d_6_*) δ 8.88 (dd, J = 4.1, 1.8 Hz, 1H, H-2), 8.35 (dd, J = 8.3, 1.7 Hz, 1H, H-4), 7.63 (dd, J = 8.3, 1.2 Hz, 1H, H-5), 7.61–7.50 (m, 3H, H-3,13′,6), 7.37 (dd, J = 7.7, 1.2 Hz, 1H, H-7), 7.20 (td, J = 9.9, 2.5 Hz, 1H, H-12′), 6.98 (td, J = 8.5, 2.6 Hz, 1H, H-9′), 5.62 (s, 2H, H-6′), 4.52 (s, 2H, H-7′).

**^13^C NMR** (151 MHz, DMSO-*d_6_*) δ 164.56 (C-5′), 164.31 (C-2′), 162.55 (dd, J = 236.3, 11.0 Hz, C-10′), 160.90 (dd, J = 241.0, 12.0 Hz, C-11′), 153.42 (C-8), 149.88 (C-2), 140.25 (C-8a), 136.43 (C-4), 132.98 (dd, J = 10.0, 4.9 Hz, C-13′), 129.66 (C-4a), 127.01 (C-6), 122.49 (C-3), 122.03 (C-5), 120.29 (dd, J = 14.6, 3.6 Hz, C-8′), 112.42–112.16 (m, C-9), 112.04 (dd, J = 21.4, 3.6 Hz, C-7), 104.57 (t, J = 25.8 Hz, C-12′), 61.10 (C-6′), 29.81 (C-7′).

**HRMS**: (ESI^+^-MS, *m*/*z*) calcd for C_19_H_13_F_2_N_3_O_2_S (M + Na)^+^: 408.0594; found: 408.0606. 

*2-[(2,4-Dichlorobenzyl)thio]-5-[(quinolin-8-yloxy)methyl]-1,3,4-oxadiazole* (**4t**)

Cream solid; Chemical formula: C_19_H_13_Cl_2_N_3_O_2_S, Yield; 40%, Mol wt: 418.30 gmol^−1^, M.p: 116.7–119.7 °C.

**^1^H NMR** (400 MHz, DMSO-*d_6_*) δ 8.86 (dd, J = 4.2, 1.7 Hz, 1H, H-2), 8.36 (dd, J = 8.3, 1.7 Hz, 1H, H-4), 7.64 (d, J = 2.2 Hz, 1H, H-12′), 7.62 (dd, J = 8.3, 1.2 Hz, 1H, H-5), 7.60–7.55 (m, 2H, H-9′,3), 7.53 (t, J = 8.0 Hz, 1H, H-6), 7.36 (dd, J = 7.7, 1.2 Hz, 1H, H-6), 7.32 (dd, *J* = 8.3, 2.2 Hz, 1H, H-10′), 5.61 (s, 2H, H-6′), 4.57 (s, 2H, H-7′).

**^13^C NMR** (101 MHz, DMSO-*d_6_*) δ 169.37 (C-5′), 168.94 (C-2′), 158.14 (C-8), 154.71 (C-2), 145.02 (C-8a), 141.19 (C-4), 139.51 (C-11′), 138.81 (C-13′), 138.15 (C-8′), 137.94 (C-9′), 134.40 (C-4a), 134.30 (C-12′), 132.75 (C-10′), 131.78 (C-6), 127.26 (C-3), 126.74 (C-5), 116.77 (C-7), 65.77 (C-6′), 38.86 (C-7′).

**HRMS**: (ESI^+^-MS, *m*/*z*) calcd for C_19_H_13_Cl_2_N_3_O_2_S (M + Na)^+^: 440.0003; found: 440.0016.

### 4.3. Synthesis of 2-(Prop-2-yn-1-ylthio)-5-[(quinolin-8-yloxy)methyl]-1,3,4-oxadiazole (***11***)

A measure of 0.722 g (3.4 mmol 1 eq) of compound **3** was dissolved in a stirring mixture of DMF (5 mL) and pre-activated K_2_CO_3_ (0.769 g, 2.0 eq). A sticky mass was formed after few minutes, then 0.36 mL of propargyl bromide (80% in toluene) in DMF (2 mL) was added and stirring continued at r.t for 4 h. On completion of the reaction as evidenced by TLC, the mixture was poured into a slurry of ice and stirred vigorously for 30 min. The resulting brown precipitate was filtered in vacuo to obtain 0.66 g compound 11 (79% yield). 

Brown solid; Chemical formula: C_15_H_11_N_3_O_2_S, Yield; 79%, Mol wt: 297.33 gmol^−1^, M.p: 79.1–82.7 °C.

**^1^H NMR** (400 MHz, DMSO-*d_6_*) δ 8.89 (dd, J = 4.2, 1.7 Hz, 1H, H-2), 8.38 (dd, J = 8.3, 1.8 Hz, 1H, H-4), 7.64 (dd, J = 8.3, 1.2 Hz, 1H, H-5), 7.59 (dd, J = 8.3, 4.1 Hz, 1H, N-3), 7.55 (t, J = 7.9 Hz, 1H, H-6), 7.40 (dd, J = 7.7, 1.2 Hz, 1H, H-7), 5.64 (s, 2H, H-6′), 4.17 (d, J = 2.6 Hz, 2H, H-7′), 3.32 (t, J = 2.6 Hz, 1H, H-8′).

**^13^C NMR** (101 MHz, DMSO-*d_6_*) δ 164.60 (C-5′), 164.11 (C-2′), 153.29 (C-8), 149.87 (C-2), 140.04 (C-8a), 136.60 (C-4), 129.64 (C-4a), 127.11 (C-6), 122.54 (C-3), 122.03 (C-5), 112.21 (C-7), 79.26 (C-8′), 75.51 (C-9′), 61.05 (C-6′), 21.19 (C-7′).

**HRMS**: (ESI^+^-MS, *m*/*z*) calcd for C_15_H_11_N_3_O_2_S (M + Na)^+^: 320.0470; found: 320.0476.

### 4.4. General Synthetic Procedure for Quinoline-1,3,4-Oxadiazole-1,2,3-Triazole Hybrids (***12a**–**t***)

To a solution of alkyne intermediate **11** (0.15 g) in DCM (10 mL) in a round bottom flask, sodium ascorbate (22 mol%) and copper(II) sulphate pentahydrate (10 mol%) in water (10 mL) were added and stirred at r.t for few minutes. Then, the appropriate azides (1.1 eq) in DCM were added and the mixture was stirred for 2–4 h. After this time, TLC analysis showed the alkyne was consumed, then the reaction was diluted with water and filtered to remove residual salts. The resulting filtrate was extracted with DCM, washed with brine and dried over anhydrous sodium sulphate, filtered, and concentrated under reduced pressure. The crude product was purified with column chromatography in EtOAc-hexane eluents to obtain pure quinoline-1,3,4-oxadiazole-1,2,3-triazole hybrids **12a**–**t** in excellent yields (43–91%). 

*2-{[(1-Pentyl-1H-1,2,3-triazol-4-yl)methyl]thio-5-[(quinolin-8-yloxy)methyl]-1,3,4-oxadiazole* (**12a**)

Yellow solid; Chemical Formula: C_20_H_22_N_6_O_2_S, Yield; 59%, Mol wt: 410.49 gmol^−1^, M.p: 90–94 °C. 

**^1^H NMR** (400 MHz, DMSO-*d_6_*) δ 8.88 (dd, J = 4.2, 1.8 Hz, 1H), 8.37 (dd, J = 8.3, 1.8 Hz, 1H), 8.13 (s, 1H), 7.63 (dd, J = 8.3, 1.3 Hz, 1H), 7.60–7.57 (m, 1H), 7.54 (d, J = 8.1 Hz, 1H), 7.40 (dd, J = 7.7, 1.3 Hz, 1H), 5.62 (s, 2H), 4.60 (s, 2H), 4.28 (t, J = 7.1 Hz, 2H), 1.73 (p, J = 7.2 Hz, 2H), 1.31–1.05 (m, 4H), 0.80 (t, J = 7.2 Hz, 3H).

**^13^C NMR** (101 MHz, DMSO-*d_6_*) δ 164.70, 164.35, 153.42, 149.92, 142.24, 140.15, 136.49, 129.65, 127.08, 124.24, 122.55, 121.97, 111.98, 61.02, 49.82, 29.78, 28.37, 27.30, 21.91, 14.18.

**HRMS**: (ESI^+^-MS, *m*/*z*) calcd for C_20_H_22_N_6_O_2_S (M + Na)^+^: 433.1423; found: 433.1413.

*2-{[(1-Hexyl-1H-1,2,3-triazol-4-yl)methyl]thio}-5-[(quinolin-8-yloxy)methyl]-1,3,4-oxadiazole* (**12b**)

Yellow solid; Chemical Formula: C_21_H_24_N_6_O_2_S, Yield; 75%, Mol wt: 424.52 gmol^−1^, M.p: 72–78 °C.

**^1^H NMR** (400 MHz, DMSO-*d_6_*) δ 8.88 (dd, *J* = 4.1, 1.7 Hz, 1H), 8.37 (dd, *J* = 8.3, 1.7 Hz, 1H), 8.13 (s, 1H), 7.67–7.51 (m, 3H), 7.40 (dd, *J* = 7.7, 1.3 Hz, 1H), 5.62 (s, 2H), 4.60 (s, 2H), 4.28 (t, *J* = 7.1 Hz, 2H), 1.72 (p, *J* = 7.2 Hz, 2H), 1.41–1.04 (m, 6H), 0.84–0.76 (m, 3H).

**^13^C NMR** (101 MHz, DMSO-*d_6_*) δ 164.70, 164.34, 153.41, 149.92, 142.25, 140.14, 136.49, 129.64, 127.09, 124.25, 122.56, 121.96, 111.96, 61.01, 49.83, 30.96, 30.04, 27.29, 25.85, 22.32, 14.25.

**HRMS**: (ESI^+^-MS, *m*/*z*) calcd for C_21_H_24_N_6_O_2_S (M + Na)^+^: 447.1579; found: 447.1568.

*2-{[(1-Benzyl-1H-1,2,3-triazol-4-yl)methyl]thio}-5-[(quinolin-8-yloxy)methyl]-1,3,4-oxadiazole* (**12c**)

Yellow solid; Chemical Formula: C_22_H_18_N_6_O_2_S, Yield; 82%, Mol wt: 430.48 gmol^−1^, M.p: 68–71 °C.

**^1^H NMR** (400 MHz, Chloroform-*d*) δ 8.86 (dd, *J* = 4.2, 1.7 Hz, 1H), 8.08 (dd, *J* = 8.3, 1.7 Hz, 1H), 7.59 (s, 1H), 7.46–7.34 (m, 3H), 7.25 (dd, *J* = 5.1, 2.0 Hz, 3H), 7.23–7.16 (m, 1H), 7.20–7.10 (m, 2H), 5.45 (s, 2H), 5.37 (s, 2H), 4.44 (s, 2H).

**^13^C NMR** (101 MHz, Chloroform-*d*) δ 165.77, 163.48, 153.07, 149.59, 143.05, 139.95, 136.32, 134.43, 129.65, 129.11, 128.76, 128.01, 126.58, 123.27, 121.95, 121.73, 110.75, 60.75, 54.19, 26.91.

**HRMS**: (ESI^+^-MS, *m*/*z*) calcd for C_20_H_22_N_6_O_2_S (M + Na)^+^: 453.1110; found: 453.1098.

*2-{[(1-(4-Chlorobenzyl)-1H-1,2,3-triazol-4-yl)methyl]thio}-5-[(quinolin-8-yloxy)methyl]-1,3,4-oxadiazole* (**12d**)

Cream white solid; Chemical Formula: C_22_H_17_ClN_6_O_2_S, Yield; 63%, Mol wt: 464.93 gmol^−1^, M.p: 92–96 °C.

**^1^H NMR** (400 MHz, Chloroform-*d*) δ 8.87–8.81 (m, 1H), 8.08 (dd, *J* = 8.3, 1.7 Hz, 1H), 7.64 (s, 1H), 7.45–7.32 (m, 3H), 7.26–7.14 (m, 3H), 7.11–7.01 (m, 2H), 5.44 (s, 2H), 5.33 (s, 2H), 4.43 (s, 2H).

**^13^C NMR** (101 MHz, Chloroform-*d*) δ 165.60, 163.58, 153.19, 149.57, 143.27, 140.19, 136.15, 134.76, 133.00, 129.67, 129.27, 129.26, 126.51, 123.25, 121.87, 121.83, 111.10, 60.92, 53.37, 26.90.

**HRMS**: (ESI^+^-MS, *m*/*z*) calcd for C_22_H_17_ClN_6_O_2_S (M + Na)^+^: 487.0720; found: 487.0706.

*2-{[(1-(4-Fluorobenzyl)-1H-1,2,3-triazol-4-yl)methyl]thio}-5-[(quinolin-8-yloxy)methyl]-1,3,4-oxadiazole* (**12e**)

Yellow solid; Chemical Formula: C_22_H_17_FN_6_O_2_S, Yield; 62%, Mol wt: 448.47 gmol^−1^, M.p: 96–101 °C.

**^1^H NMR** (400 MHz, Chloroform-*d*) δ 8.95 (d, *J* = 3.7 Hz, 1H, H-2), 8.20 (dd, *J* = 8.2, 1.7 Hz, 1H, H-4), 7.70 (s, 1H, H-5″), 7.56–7.42 (m, 3H, H-3,H-5, H-6), 7.28 (dd, *J* = 7.3, 1.6 Hz, 1H, H-7), 7.21 (dd, *J* = 8.5, 5.3 Hz, 2H, H-2′″&H-6′″), 7.07–6.96 (m, 2H, H-3′″&H-5′″), 5.54 (s, 2H, H-6′), 5.42 (s, 2H, H-7″), 4.51 (s, 2H, H-6″).

**^13^C NMR** (101 MHz, Chloroform-*d*) δ 165.81, C-5′, 164.08, C-4′″, 163.45, C-2′, 161.61, C-4′″, 152.92, C-8, 149.42, C-2, 143.25,C-4″, 139.60, C-8a, 136.66, C-4, 130.34 (d, *J* = 3.4 Hz, C-1′″), 129.92 (d, *J* = 8.4 Hz, C-2′″&6′″), 129.68, C-4a, 126.72, C-6, 123.24. C-5″, 121.96, C-3, 121.73, C-5, 116.11 (d, *J* = 21.9 Hz, C-3′″&5′″), 110.84, C-7, 60.74, C-6′, 53.43, C-7″, 26.87, C-6″.

**HRMS**: (ESI^+^-MS, *m*/*z*) calcd for C_22_H_17_ClN_6_O_2_S (M + Na)^+^: 471.1004; found: 471.0999.

*2-{[(1-(4-Bromobenzyl)-1H-1,2,3-triazol-4-yl)methyl]thio}-5-[(quinolin-8-yloxy)methyl]-1,3,4-oxadiazole* (**12f**)

Cream solid; Chemical Formula: C_22_H_17_BrN_6_O_2_S, Yield; 53%, Mol wt: 509.38 gmol^−1^, M.p: 78–82 °C.

**^1^H NMR** (400 MHz, Chloroform-*d*) δ 8.89–8.83 (m, 1H), 8.10 (dd, *J* = 8.3, 1.6 Hz, 1H), 7.65 (s, 1H), 7.47–7.35 (m, 5H), 7.20 (dd, *J* = 7.4, 1.5 Hz, 1H), 7.05–6.97 (m, 2H), 5.46 (s, 2H), 5.33 (s, 2H), 4.45 (s, 2H).

**^13^C NMR** (101 MHz, Chloroform-*d*) δ 165.75, 163.50, 153.07, 149.59, 143.33, 139.94, 136.34, 133.48, 132.26, 129.66, 129.60, 126.59, 123.36, 122.91, 121.96, 121.75, 110.69, 60.73, 53.46, 26.85.

**HRMS**: (ESI^+^-MS, *m*/*z*) calcd for C_22_H_17_BrN_6_O_2_S (M + Na)^+^: 517.0058; found: 517.0046.

*2-{[(1-(4-Nitrobenzyl)-1H-1,2,3-triazol-4-yl)methyl]thio}-5-[(quinolin-8-yloxy)methyl]-1,3,4-oxadiazole* (**12g**)

Bright yellow solid; Chemical Formula: C_22_H_17_N7O_4_S, Yield; 74%, Mol wt: 475.48 gmol^−1^, M.p: 100–106 °C.

**^1^H NMR** (400 MHz, Chloroform-*d*) δ 8.83 (dd, *J* = 4.2, 1.7 Hz, 1H), 8.12–8.04 (m, 3H), 7.77 (s, 1H), 7.46–7.34 (m, 3H), 7.28–7.14 (m, 2H), 5.49 (s, 2H), 5.44 (s, 2H), 4.45 (s, 2H).

**^13^C NMR** (101 MHz, Chloroform-*d*) δ 165.55, 163.66, 149.53, 136.23, 129.72, 128.54, 126.52, 124.21, 123.70, 121.89, 111.17, 60.98, 53.06, 26.87.

**HRMS**: (ESI^+^-MS, *m*/*z*) calcd for C_22_H_17_N7O_4_S (M + Na)^+^: 498.0960; found: 498.0951.

*2-{[(1-(3-Chlorobenzyl)-1H-1,2,3-triazol-4-yl)methyl]thio}-5-[(quinolin-8-yloxy)methyl]-1,3,4-oxadiazole* (**12h**)

Cream white solid; Chemical Formula: C_22_H_17_ClN_6_O_2_S, Yield; 76%, Mol wt: 464.93 gmol^−1^, M.p: 78–84 °C.

**^1^H NMR** (600 MHz, Chloroform-*d*) δ 8.87 (dd, *J* = 4.3, 1.7 Hz, 1H), 8.11 (dd, *J* = 8.2, 1.7 Hz, 1H), 7.67 (s, 1H), 7.46–7.37 (m, 3H), 7.25–7.16 (m, 3H), 7.13 (d, *J* = 1.9 Hz, 1H), 7.01 (dt, *J* = 7.4, 1.5 Hz, 1H), 5.47 (s, 2H), 5.35 (s, 2H), 4.46 (s, 2H).

**^13^C NMR** (101 MHz, Chloroform-*d*) δ 165.69, 163.56, 153.17, 149.70, 143.33, 140.19, 136.38, 136.11, 134.95, 130.41, 129.65, 128.97, 128.03, 126.48, 126.02, 123.44, 121.95, 121.77, 110.63, 60.75, 53.43, 26.85.

**HRMS**: (ESI^+^-MS, *m*/*z*) calcd for C_22_H_17_ClN_6_O_2_S (M + Na)^+^: 487.0720; found: 487.0730.

*2-{[(1-(2,4-Dichlorobenzyl)-1H-1,2,3-triazol-4-yl)methyl]thio}-5-[(quinolin-8-yloxy)methyl]-1,3,4-oxadiazole* (**12i**)

Cream white solid; Chemical Formula: C_22_H_16_C_l2_N_6_O_2_S, Yield; 57%, Mol wt: 499.37 gmol^−1^, M.p: 124–130 °C.

**^1^H NMR** (400 MHz, Chloroform-*d*) δ 8.97 (dd, *J* = 4.3, 1.7 Hz, 1H), 8.22 (dd, *J* = 8.4, 1.7 Hz, 1H), 7.82 (s, 1H), 7.59–7.46 (m, 3H), 7.42 (d, *J* = 2.1 Hz, 1H), 7.31 (dd, *J* = 7.4, 1.6 Hz, 1H), 7.22 (dd, *J* = 8.3, 2.1 Hz, 1H), 7.04 (d, *J* = 8.3 Hz, 1H), 5.57 (d, *J* = 3.4 Hz, 4H), 4.56 (s, 2H).

**^13^C NMR** (101 MHz, Chloroform-*d*) δ 165.73, 163.47, 152.89, 149.41, 143.17, 139.57, 136.67, 134.04, 130.95, 130.91, 129.74, 129.67, 127.93, 126.72, 123.76, 121.96, 121.72, 110.83, 60.73, 50.84.

**HRMS**: (ESI^+^-MS, *m*/*z*) calcd for C_22_H_16_C_l2_N_6_O_2_S (M + Na)^+^: 521.0411; found: 521.0421.

*2-{[(1-(3,4-Difluorobenzyl)-1H-1,2,3-triazol-4-yl)methyl]thio}-5-[(quinolin-8-yloxy)methyl]-1,3,4-oxadiazole* (**12j**)

Cream white solid; Chemical Formula: C_22_H_16_F_2_N_6_O_2_S, Yield; 61%, Mol wt: 466.46 gmol^−1^, M.p: 70–75 °C.

**^1^H NMR** (400 MHz, Chloroform-*d*) δ 8.97 (dd, *J* = 4.2, 1.7 Hz, 1H), 8.20 (dd, *J* = 8.4, 1.7 Hz, 1H), 7.78 (s, 1H), 7.56–7.45 (m, 3H), 7.34–7.19 (m, 2H), 6.92–6.80 (m, 2H), 5.56 (s, 2H), 5.49 (s, 2H), 4.54 (s, 2H).

**^13^C NMR** (101 MHz, Chloroform-*d*) δ 165.76, 164.55 (dd, *J* = 250.5, 12.1 Hz), 163.49, 160.65 (dd, *J* = 251.3, 27.0 Hz), 153.00, 149.52, 143.18, 139.79, 136.47, 131.51 (dd, *J* = 9.9, 4.7 Hz), 129.67, 126.64, 123.41, 121.95, 121.73, 117.78 (dt, *J* = 14.6, 3.7 Hz), 112.15 (dd, *J* = 21.5, 3.8 Hz), 110.77, 104.40 (t, *J* = 25.4 Hz), 60.74, 47.17 (d, *J* = 3.8 Hz), 26.84.

**HRMS**: (ESI^+^-MS, *m*/*z*) calcd for C_22_H_16_C_l2_N_6_O_2_S (M + Na)^+^: 521.0411; found: 521.0421.

*2-{[(1-Phenyl-1H-1,2,3-triazol-4-yl)methyl]thio}-5-[(quinolin-8-yloxy)methyl]-1,3,4-oxadiazole* (**12k**)

Light brown solid; Chemical Formula: C_21_H_16_N_6_O_2_S, Yield; 91%, Mol wt: 416.46 gmol^−1^, M.p: 122–128 °C.

**^1^H NMR** (400 MHz, Chloroform-*d*) δ 8.83 (dd, *J* = 4.3, 1.7 Hz, 1H), 8.11 (s, 1H), 8.05 (dd, *J* = 8.3, 1.8 Hz, 1H), 7.60 (dd, *J* = 7.6, 1.8 Hz, 2H), 7.45–7.28 (m, 6H), 7.18 (dd, *J* = 7.3, 1.7 Hz, 1H), 5.47 (s, 2H), 4.54 (s, 2H).

**^13^C NMR** (101 MHz, Chloroform-*d*) δ 165.75, 163.64, 153.18, 149.71, 143.46, 140.23, 136.82, 136.06, 129.70, 129.63, 128.85, 126.44, 121.91, 121.79, 121.59, 120.60, 110.77, 60.83, 26.83.

**HRMS**: (ESI^+^-MS, *m*/*z*) calcd for C_21_H_16_N_6_O_2_S (M + Na)^+^: 439.0953; found: 439.0959.

*2-{[(1-(4-Chlorophenyl)-1H-1,2,3-triazol-4-yl)methyl]thio}-5-[(quinolin-8-yloxy)methyl]-1,3,4-oxadiazole* (**12l**)

Yellow solid; Chemical Formula: C_21_H_15_ClN_6_O_2_S, Yield; 79%, Mol wt: 450.90 gmol^−1^, M.p: 132–136 °C.

**^1^H NMR** (400 MHz, Chloroform-*d*) δ 8.85 (dd, *J* = 4.3, 1.7 Hz, 1H), 8.15–8.05 (m, 2H), 7.61–7.52 (m, 2H), 7.48–7.33 (m, 5H), 7.20 (dd, *J* = 7.3, 1.6 Hz, 1H), 5.48 (s, 2H), 4.54 (s, 2H).

**^13^C NMR** (101 MHz, Chloroform-*d*) δ 165.73, 163.64, 153.06, 149.59, 143.79, 139.94, 136.34, 135.30, 134.62, 129.87, 129.66, 126.56, 121.96, 121.79, 121.76, 121.57, 110.77, 60.80, 26.74.

**HRMS**: (ESI^+^-MS, *m*/*z*) calcd for C_21_H_16_N_6_O_2_S (M + Na)^+^: 473.0563; found: 473.0560.

*2-{[(1-(4-Fluorophenyl)-1H-1,2,3-triazol-4-yl)methyl]thio}-5-[(quinolin-8-yloxy)methyl]-1,3,4-oxadiazole* (**12m**)

Brown crystalline; Chemical Formula: C_21_H_15_FN_6_O_2_S, Yield; 84%, Mol wt: 434.45 gmol^−1^, M.p: 134–140 °C.

**^1^H NMR** (400 MHz, Chloroform-*d*) δ 8.83 (dd, *J* = 4.3, 1.8 Hz, 1H), 8.10–8.02 (m, 2H), 7.64–7.53 (m, 2H), 7.44–7.31 (m, 3H), 7.19 (d, *J* = 7.4 Hz, 1H), 7.13–7.03 (m, 2H), 5.47 (s, 2H), 4.53 (s, 2H).

**^13^C NMR** (101 MHz, Chloroform-*d*) δ 165.72, 161.21, 153.20, 149.72, 143.64, 140.23, 136.05, 133.09, 129.64, 126.44, 122.65, 122.56, 121.93, 121.80, 116.76, 116.53, 110.71, 60.81, 26.76.

**HRMS**: (ESI^+^-MS, *m*/*z*) calcd for C_21_H_16_N_6_O_2_S (M + Na)^+^: 457.0859; found: 457.0851.

*2-{[(1-(4-Bromophenyl)-1H-1,2,3-triazol-4-yl)methyl]thio}-5-[(quinolin-8-yloxy)methyl]-1,3,4-oxadiazole* (**12n**)

Brown solid; Chemical Formula: C_21_H_15_BrN_6_O_2_S, Yield; 77%, Mol wt: 495.35 gmol^−1^, M.p: 126–132 °C.

**^1^H NMR** (400 MHz, Chloroform-*d*) δ 8.84 (dd, *J* = 4.2, 1.7 Hz, 1H), 8.13–8.03 (m, 2H), 7.56–7.46 (m, 4H), 7.45–7.32 (m, 3H), 7.19 (dd, *J* = 6.8, 2.2 Hz, 1H), 5.48 (s, 2H), 4.54 (s, 2H).

**^13^C NMR** (101 MHz, Chloroform-*d*) δ 165.71, 163.68, 153.20, 149.73, 143.83, 140.24, 136.07, 135.79, 132.84, 129.65, 126.45, 122.51, 121.98, 121.94, 121.82, 121.47, 110.73, 60.82, 26.74.

**HRMS**: (ESI^+^-MS, *m*/*z*) calcd for C_21_H_15_BrN_6_O_2_S (M + Na)^+^: 517.0058; found: 517.0046.

*2-{[(1-(4-Nitrophenyl)-1H-1,2,3-triazol-4-yl)methyl]thio}-5-[(quinolin-8-yloxy)methyl]-1,3,4-oxadiazole* (**12o**)

Bright yellow crystals; Chemical Formula: C_21_H_15_N_7_O_4_S, Yield; 83%, Mol wt: 461.45 gmol^−1^, M.p: 150–156 °C.

**^1^H NMR** (400 MHz, Chloroform-*d*) δ 8.94 (dd, *J* = 4.3, 1.7 Hz, 1H), 8.42 (s, 1H), 8.40–8.32 (m, 2H), 8.20 (dd, *J* = 8.3, 1.7 Hz, 1H), 8.00–7.92 (m, 2H), 7.56–7.44 (m, 3H), 7.30 (dd, *J* = 7.3, 1.6 Hz, 1H), 5.57 (s, 2H), 4.65 (s, 2H).

**^13^C NMR** (101 MHz, Chloroform-*d*) δ 165.62, 163.67, 152.99, 149.52, 147.24, 144.57, 140.96, 139.75, 136.53, 129.68, 126.64, 125.44, 122.00, 121.83, 121.67, 120.61, 110.76, 60.78, 26.59.

**HRMS**: (ESI^+^-MS, *m*/*z*) calcd for C_21_H_15_N_7_O_4_S (M + Na)^+^: 484.0804; found: 484.0799.

*2-[(Quinolin-8-yloxy)methyl]-5-{[(1-(p-tolyl)-1h-1,2,3-triazol-4-yl)methyl]thio}-1,3,4-oxadiazole* (**12p**)

Light brown solid; Chemical Formula: C_22_H_18_N_6_O_2_S, Yield; 73%, Mol wt: 430.48 gmol^−1^, M.p: 78–80 °C.

**^1^H NMR** (400 MHz, Chloroform-*d*) δ 8.87 (dd, *J* = 4.2, 1.8 Hz, 1H), 8.10 (dd, *J* = 8.3, 1.7 Hz, 1H), 8.07 (s, 1H), 7.52–7.45 (m, 2H), 7.40 (dddd, *J* = 9.7, 8.3, 5.9, 1.5 Hz, 3H), 7.21 (dd, *J* = 8.7, 7.3 Hz, 3H), 5.50 (d, *J* = 1.8 Hz, 2H), 4.55 (s, 2H), 2.33 (s, 3H).

**^13^C NMR** (101 MHz, Chloroform-*d*) δ 165.83, 163.58, 152.96, 149.43, 143.29, 139.71, 138.99, 136.58, 134.58, 130.19, 129.68, 126.68, 121.92, 121.77, 121.55, 111.09, 60.88, 26.89, 21.08.

**HRMS**: (ESI^+^-MS, *m*/*z*) calcd for C_21_H_15_N_7_O_4_S (M + Na)^+^: 453.1110; found: 453.1097.

*2-{[(1-(4-Methoxyphenyl)-1H-1,2,3-triazol-4-yl)methyl]thio}-5-[(quinolin-8-yloxy)methyl]-1,3,4-oxadiazole* (**12q**)

Black solid; Chemical Formula: C_22_H_18_N_6_O_3_S, Yield; 76%, Mol wt: 446.48 gmol^−1^, M.p: 122–128 °C.

**^1^H NMR** (400 MHz, Chloroform-*d*) δ 8.84 (dd, *J* = 4.3, 1.7 Hz, 1H), 8.07 (dd, *J* = 8.3, 1.7 Hz, 1H), 8.02 (s, 1H), 7.53–7.45 (m, 2H), 7.44–7.32 (m, 3H), 7.20 (dd, *J* = 7.3, 1.6 Hz, 1H), 6.93–6.84 (m, 2H), 5.48 (s, 2H), 4.53 (s, 2H), 3.76 (s, 3H).

**^13^C NMR** (101 MHz, Chloroform-*d*) δ 165.80, 163.60, 159.87, 153.12, 149.62, 143.21, 140.08, 136.20, 130.28, 129.64, 126.52, 122.23, 121.91, 121.77, 121.71, 114.71, 110.84, 60.83, 55.62, 26.88.

**HRMS**: (ESI^+^-MS, *m*/*z*) calcd for C_22_H_18_N_6_O_3_S (M + Na)^+^: 469.1059; found: 469.1049.

*2-{[(1-(2,4-Dichlorophenyl)-1H-1,2,3-triazol-4-yl)methyl]thio}-5-[(quinolin-8-yloxy)methyl]-1,3,4-oxadiazole* (**12r**)

Light brown solid; Chemical Formula: C_21_H_14_C_l2_N_6_O_2_S, Yield; 73%, Mol wt: 485.35 gmol^−1^, M.p: 112–116 °C.

**^1^H NMR** (400 MHz, Chloroform-*d*) δ 8.85 (dd, *J* = 4.3, 1.7 Hz, 1H), 8.14 (s, 1H), 8.08 (dd, *J* = 8.3, 1.7 Hz, 1H), 7.55 (d, *J* = 2.4 Hz, 1H), 7.45–7.30 (m, 5H), 7.20 (dd, *J* = 7.2, 1.7 Hz, 1H), 5.48 (s, 2H), 4.56 (s, 2H).

**^13^C NMR** (101 MHz, Chloroform-*d*) δ 165.59, 163.61, 153.04, 149.57, 142.75, 139.91, 136.31, 135.29, 133.67, 131.59, 130.81, 129.64, 127.79, 126.74, 126.55, 125.39, 121.93, 121.74, 110.69, 60.73, 26.68.

**HRMS**: (ESI^+^-MS, *m*/*z*) calcd for C_21_H_14_C_l2_N_6_O_2_S (M + Na)^+^: 521.0411; found: 521.0421.

*2-{[(1-(2,6-Dichlorophenyl)-1H-1,2,3-triazol-4-yl)methyl]thio}-5-[(quinolin-8-yloxy)methyl]-1,3,4-oxadiazole* (**12s**)

Light brown solid; Chemical Formula: C_21_H_14_C_l2_N_6_O_2_S, Yield; 77 %, Mol wt: 485.35 gmol^−1^, M.p: 113–118 °C.

**^1^H NMR** (400 MHz, Chloroform-*d*) δ 8.84 (dd, *J* = 4.3, 1.7 Hz, 1H), 8.07 (dd, *J* = 8.3, 1.7 Hz, 1H), 7.89 (s, 1H), 7.44–7.26 (m, 6H), 7.23–7.16 (m, 1H), 5.49 (s, 2H), 4.58 (s, 2H).

**^13^C NMR** (101 MHz, Chloroform-*d*) δ 163.63, 153.09, 149.53, 142.67, 140.02, 136.23, 131.72, 129.63, 128.76, 126.54, 125.80, 121.89, 121.74, 110.92, 60.88, 26.91.

**HRMS**: (ESI^+^-MS, *m*/*z*) calcd for C_21_H_14_C_l2_N_6_O_2_S (M + Na)^+^: 508.01; found: 508.35.

*2-{[(1-(2,6-Dimethylphenyl)-1H-1,2,3-triazol-4-yl)methyl]thio}-5-[(quinolin-8-yloxy)methyl]-1,3,4-oxadiazole* (**12t**)

Brown solid; Chemical Formula: C_23_H_20_N_6_O_2_S, Yield; 43%, Mol wt: 444.51 gmol^−1^, M.p: 110–116 °C.

**^1^H NMR** (400 MHz, Chloroform-*d*) δ 8.82 (dd, *J* = 4.2, 1.7 Hz, 1H), 8.05 (dd, *J* = 8.4, 1.7 Hz, 1H), 7.71 (s, 1H), 7.43–7.31 (m, 2H), 7.25–7.15 (m, 1H), 7.05 (d, *J* = 7.6 Hz, 2H), 5.49 (s, 2H), 4.57 (s, 2H), 1.83 (s, 6H).

**^13^C NMR** (101 MHz, Chloroform-*d*) δ 165.71, 163.61, 153.09, 149.58, 142.63, 140.05, 136.19, 135.69, 135.34, 130.03, 129.63, 128.41, 126.48, 125.09, 121.90, 121.77, 110.82, 60.81, 27.03, 17.31.

**HRMS**: (ESI^+^-MS, *m*/*z*) calcd for C_23_H_20_N_6_O_2_S, (M + Na)^+^: 467.1266; found: 467.1267.

### 4.5. α-Glucosidase Inhibitory Activity

The α-glucosidase inhibitory activity was evaluated using a previous method [41] with slight modifications. Briefly, 100 µL aliquot of each compound or acarbose (62.5–500 µM) was added to α-glucosidase (1.0 U/mL) solution in 100 mM sodium phosphate buffer (pH 6.8). The reaction was incubated at 37 °C for 15 min before adding 50 µL of 5 mM pNPG solution. After further incubation at 37 °C for another 30 min, the absorbance of the resulting solution was measured at 405 nm. Each compound’s inhibitory activity was calculated as a percentage of the control sample using the expression below:

$$\%\;\mathrm{Inhibition}=\left(1-\frac{\mathrm{Absorbance}\;\mathrm{of}\;\mathrm{compound}}{\mathrm{Absorbance}\;\mathrm{of}\;\mathrm{control}}\right)\times 100$$

### 4.6. α-Amylase Inhibitory Activity

α-Amylase inhibitory activity was determined according to Ibitoye et al. [42] with slight modifications. Briefly, 200 μL of each compound or acarbose (62.5–500 μM) was incubated at 25 °C for 10 min, with 200 μL solution of porcine pancreatic amylase (0.5 mg/mL) prepared in 6 μM NaCl and 20 mM sodium phosphate buffer (pH 6.9). Then, 500 μL of 1% starch solution was added before further incubation at 25 °C for 15 min. The reaction in the mixture was terminated with a 1 mL dinitrosalicylate reagent before boiling for 10 min. The cooled mixture was diluted with 5 mL distilled water, and the absorbance was read at 540 nm. The inhibitory activity was calculated using the formula below:

$$\%\;\mathrm{Inhibition}=\left(1-\frac{\mathrm{Absorbance}\;\mathrm{of}\;\mathrm{compound}}{\mathrm{Absorbance}\;\mathrm{of}\;\mathrm{control}}\right)\times 100$$

### 4.7. Antioxidant Activity

#### 4.7.1. Nitric Oxide Scavenging Activity 

The antioxidant capacity of the compounds to scavenge nitric oxide radicals was determined using a modified version of the protocol by Kurian et al. [43]. Briefly, 250 μL of sodium nitroprusside (10 mM) prepared in sodium phosphate buffer (pH 7.4) was added to 500 μL of the compound solution (62.5–500 μM) or distilled water (control). The resulting solution was incubated at 37 °C for 2 h. Then, 250 μL of Griess reagent was added to the reaction mixture before the absorbance was measured at 546 nm. The nitric oxide scavenging activity of the compounds was calculated with the formula:

$$\mathrm{Nitric}\;\mathrm{oxide}\;\mathrm{scavenging}\;(\%)=\left(1-\frac{\mathrm{Absorbance}\;\mathrm{of}\;\mathrm{compound}}{\mathrm{Absorbance}\;\mathrm{of}\;\mathrm{control}}\right)\times 100$$

#### 4.7.2. 2,2′-Diphenyl-1-Picrylhydrazyl (DPPH) Scavenging Activity

The compounds were evaluated for their ability to scavenge stable DPPH radicals, using a modification of the method by Turkoglu et al. [44]. 2 mL of varied concentrations (62.5–500 μM) of compound or gallic acid was added to 2 mL 0.3 mM DPPH prepared in methanol. After thorough mixing, the solution was kept in a dark chamber at room temperature (25 °C) for 30 min. Then, the absorbance was measured at 517 nm, and the DPPH radical scavenging activity was calculated as follows:

$$\mathrm{DPPH}\;\mathrm{scavenging}\;\mathrm{activity}\;(\%)=\left(1-\frac{\mathrm{Absorbance}\;\mathrm{of}\;\mathrm{compound}}{\mathrm{Absorbance}\;\mathrm{of}\;\mathrm{control}}\right)\times 100$$

#### 4.7.3. Ferric Reducing Antioxidant Power 

500 μL of varying concentrations of the compounds or gallic acid (62.5–500 μM) was added to 250 μL distilled water, 100 μL of 200 mM phosphate buffer (pH = 7.3), and 100 μL of 1% potassium ferricyanide [K_3_Fe(CN)_6_]. The mixture was incubated at 50 °C for 20 min, followed by acidification with 100 μL trichloroacetic acid (10%). After centrifugation at 3500 rpm for 10 min, 200 μL of the supernatant was transferred into another test tube containing 200 μL of distilled water and 0.8 mL of FeCl_3_ (0.1%) [45]. Finally, the absorbance was read at 700 nm, and the total reductive antioxidant power was calculated thus:

$$\mathrm{Ferric}\;\mathrm{reducing}\;\mathrm{antioxidant}\;\mathrm{power}\;(\%)=\frac{\mathrm{Absorbance}\;\mathrm{of}\;\mathrm{compound}}{\mathrm{Absorbance}\;\mathrm{of}\;500\text{µ}M\;\mathrm{gallic}\;\mathrm{acid}}\times 100$$

#### 4.7.4. Mode of α-Glucosidase Inhibition

The mode of inhibition of α-glucosidase of the **4b**, **4i**, **4j**, **4k**, **4l**, **12k**, and **12m** compounds was determined using the method described by Kazeem et al. 2013, with modifications [46]. Briefly, 50 µL of the **4b**, **4i**, **4j**, **4k**, **4l**, **12k**, and **12m** compounds at (0 µM, 75 µM, 150 µM and 300 µM) concentrations were preincubated with 100 µL of α-glucosidase solution for 10 min at 25 °C in one set of tubes. In another set of tubes, α-glucosidase was preincubated with 50 µL of phosphate buffer (pH 6.9). 25 µL of pNPG at increasing concentrations (0.125–4.0 mg/mL) was added to both sets of solution to start the reaction. Immediately, the absorbance of the resulting solution was monitored for 2 min in a spectrophotometer at 405 nm. The amount of 4-nitrophenol released was determined from a 4-nitrophenol standard curve and then converted to reaction velocities. 

### 4.8. Molecular Modeling

All in silico calculations were performed with the Schrodinger molecular modeling suite (version 2021-2) using the OPLS4 force field.

#### 4.8.1. Homology Modeling of α-Glucosidase and SiteMap

Using the homology modeling module in Schrödinger, the FASTA format sequence of *Saccharomyces cerevisiae* (UniProt entry: P38138) was downloaded and a Protein BLAST was run to obtain a structure template for the homology model building. From the retuned templates, the native structure of oligo-1,6-glucosidase isomaltase yeast (PDB: 3A4A) was selected. The sequences of the target and template were aligned, and the model was built and its energy minimized with Prime. The structural precision of the model was evaluated with a Ramachandran plot and structure reliability report. Thereafter, SiteMap calculations were performed using the default settings to identify potential allosteric sites.

#### 4.8.2. Molecular Docking

The lowest-energy 3D conformations of the identified inhibitors were generated with LigPrep [47], assigning their protonation and ionization states with Epik [48]. The minimized 3D coordinates of the built α-glucosidase model were generated with Protein Preparation Wizard [49] by adding hydrogens, filling in missing loops, and generating heteroatoms and tautomers with Epik at pH 7.2 ± 0.2. This was followed by hydrogen bond network optimization and restrained minimization to an RMSD of 0.30 Å. The prepared ligands were then docked at the allosteric site deduced from SiteMap analysis using the induced-fit docking module (IFD) [50]. In brief, the employed standard protocol included defining the ligand grid box as the centroid of the SiteMap points, setting the ligand box size to ≤20 Å and conformational sampling at an energy window of 2.5 kcalmol^−1^. The initial glide docking stage produced poses which were processed for Prime side chain refinement. Finally, complexes with energy of 30 kcalmol^−1^ were redocked using the extra precision mode to avoid false positives and the 10 best poses were selected for analysis. The protein–ligand interactions were characterized by hydrogen bonds, hydrophobic interactions, and π–π stacking. 

#### 4.8.3. Molecular Dynamics Simulations 

All MD simulations were performed with the Desmond [51] module. The required model systems for compounds **4i** and **12k** were generated with the system builder in an orthorhombic box in the SPC water model, and periodic boundary conditions of 10 Å for all dimensions. Before model relaxation with NVT and NPT ensembles, 0.15 M of Na^+^ and Cl^-^ ions were added to neutralize the system, and minimization with 1000 steps of steepest descent was performed using the NPT ensemble. The MD simulation was thereafter conducted for 200 ns at a constant temperature (300.15 K) and pressure (1.01325 bar), while recording the trajectory 200 ps intervals. Trajectory analysis was achieved with the simulation interaction diagram tool.

## Figures and Tables

**Table 1:** IC_50_ (µM) for antioxidant profiling of compounds **4a**–**t** and **12a**–**t**.

Entry	R	DPPH	FRAP	NO	Entry	R	DPPH	FRAP	NO
**8-HQ**	n/a	51.81	–	183.90	**3**	H	25.15	–	463.85
**4a**	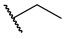	70.94	–	**2.67**	**12a**	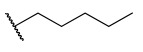	89.98	–	–
**4b**	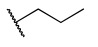	78.20	–	–	**12b**	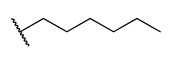	80.00	–	–
**4c**	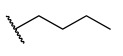	79.03	–	–	**12c**	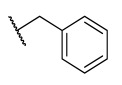	80.17	–	–
**4d**	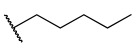	82.90	–	457.38	**12d**	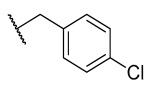	80.40	–	–
**4e**	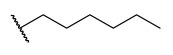	75.74	–	–	**12e**	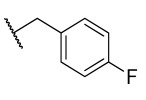	92.82	–	–
**4f**	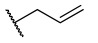	72.11	–	–	**12f**	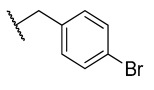	91.78	–	481.76
**4g**	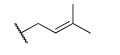	86.39	–	–	**12g**	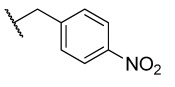	81.91	–	3.01
**4h**	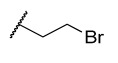	75.35	–	–	**12h**	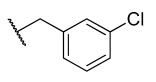	90.69	–	–
**4i**	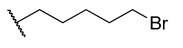	54.86	**22.88**	197.93	**12i**	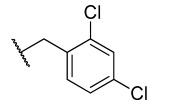	95.38	–	–
**4j**	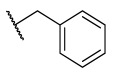	54.86	–	399.32	**12j**	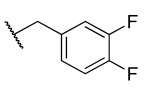	101.73	–	–
**4k**	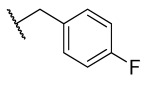	81.13	–	–	**12k**	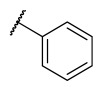	85.72	–	–
**4l**	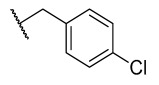	77.71	–	–	**12l**	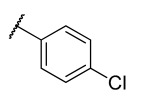	81.74	–	–
**4m**	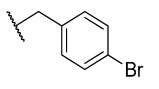	85.15	–	–	**12m**	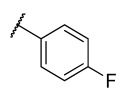	88.69	–	–
**4n**	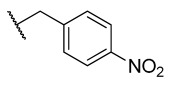	77.23	–	–	**12n**	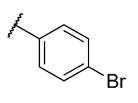	92.19	–	–
**4o**	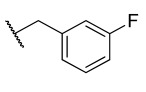	76.85	–	–	**12o**	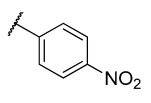	88.92	–	–
**4p**	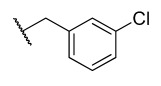	67.99	–	–	**12p**	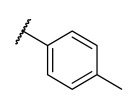	96.04	–	–
**4q**	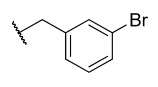	78.59	–	–	**12q**	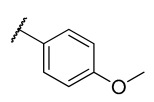	98.50	–	–
**4r**	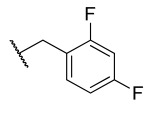	64.52	–	–	**12r**	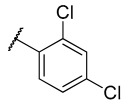	100.96	–	–
**4s**	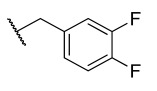	87.22	–	–	**12s**	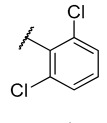	84.48	–	–
**4t**	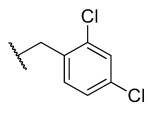	93.97	–	–	**12t**	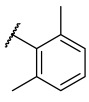	90.02	–	–
Gallic acid		26.63	17.85	728.68	Ascorbic acid			102.62	

“–” = greater than 500 μM.

**Table 2:** IC_50_ (µM) for α-glucosidase and α-amylase inhibition by compounds **4a**–**t** and **12a**–**t**.

Entry	R	α-Glucosidase	α-Amylase	Entry	R	α-Glucosidase	α-Amylase
**8-HQ**	n/a	29.28	–	**3**	H	34.70	–
**4a**	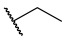	41.60	–	**12a**	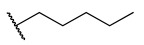	35.19	–
**4b**	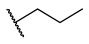	24.87	–	**12b**	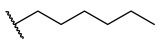	36.74	–
**4c**	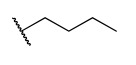	41.35	–	**12c**	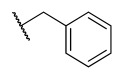	46.86	–
**4d**	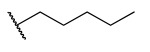	46.66	–	**12d**	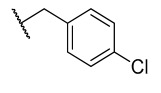	38.11	–
**4e**	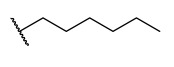	48.08	210.26	**12e**	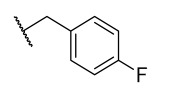	39.34	–
**4f**	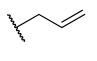	34.50	–	**12f**	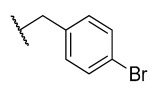	56.36	–
**4g**	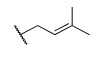	34.09	–	**12g**	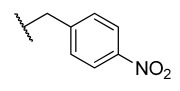	46.51	–
**4h**	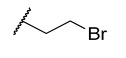	46.07	–	**12h**	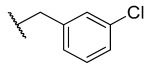	63.59	–
**4i**	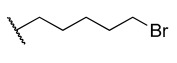	15.85	–	**12i**	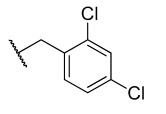	42.79	–
**4j**	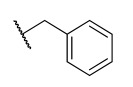	26.67	–	**12j**	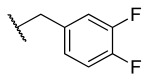	59.02	–
**4k**	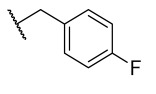	23.69	–	**12k**	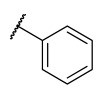	22.47	–
**4l**	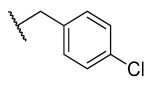	25.23	–	**12l**	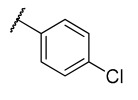	38.02	–
**4m**	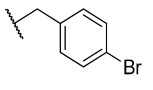	28.31	–	**12m**	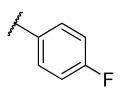	26.81	–
**4n**	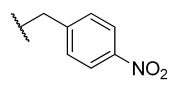	32.76	–	**12n**	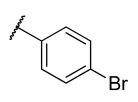	35.22	–
**4o**	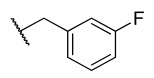	48.84	–	**12o**	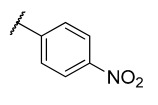	38.30	–
**4p**	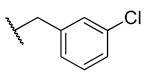	29.56	–	**12p**	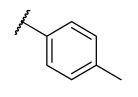	44.07	494.76
**4q**	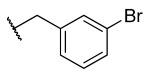	29.64	426.77	**12q**	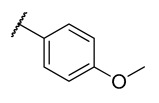	62.05	–
**4r**	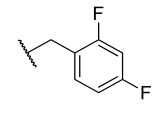	28.22	–	**12r**	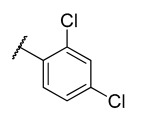	48.54	–
**4s**	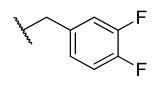	35.37	–	**12s**	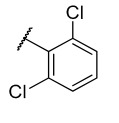	38.79	–
**4t**	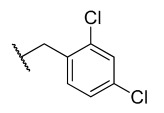	27.67	–	**12t**	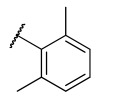	44.12	–
Acarbose		17.85	84.26				

“–” = greater than 500 μM.

## Data Availability

All the research data are available in the supporting information section.

## Supplementary Materials

The following supporting information can be downloaded at: https://www.mdpi.com/article/10.3390/ph15081035/s1, Table S1: Details of SiteMap analysis to select the putative allosteric site; Table S2: Induced docking results at the selected allosteric site.
