# Supplementary material for: α-Glucosidase and α-Amylase Inhibitory Potentials of Quinoline–1,3,4-oxadiazole Conjugates Bearing 1,2,3-Triazole with Antioxidant Activity, Kinetic Studies, and Computational Validation
Source: Pharmaceuticals (Basel). 2022 Aug 22;15(8):1035. doi: 10.3390/ph15081035 (PMC9414972; doi:10.3390/ph15081035)
Supplement: Supplementary file 1 [file pharmaceuticals-15-01035-s001.zip › pharmaceuticals-1852562-supplementary.pdf]

SUPPORTING INFORMATION

α-Glucosidase and α-amylase inhibitory potentials of quinoline–1,3,4-oxadiazole conjugates bearing 1,2,3-triazole with antioxidant activity, kinetic studies and computational validation

Nosipho Cele [a], Paul Awolade [a], Pule Seboletswe[a], Kolawole Olofinsan [b], Md. Shahidul Islam[b], Parvesh Singh[a*]

[a] *School of Chemistry and Physics, University of KwaZulu-Natal, P/Bag X54001, Westville, Durban, South Africa*
[b] *Department of Biochemistry, School of Life Sciences, University of Kwazulu-Natal, Westville, Durban, South Africa*

*Email: singhp4@ukzn.ac.za, parveshdurban@gmail.com

Contents

Table S1: Details of SiteMap analysis to select the putative allosteric site 2

Table S2: Induced docking results at the selected allosteric site 2

S.3 Synthetic procedures 3

S.3.1 General procedure for the synthesis of alkyl azides 6a,b 3

S.3.2 General procedure for the synthesis of benzyl azides 8a-h 3

S.3.3 General procedure for the synthesis of phenyl azides 10a-j 3

S.4 Spectral data 4

Table S1: Details of SiteMap analysis to select the putative allosteric site

Site	SiteScore	DScore	Volume	Balance	Residues
1	1.064	730.247	730.247	0.569	155,156,157,159,160,176,177,215,216,217,218,228,229,231,232,234,235,236,237,239,240,241,242,245,276,277,278,279,280,300,301,304,307,308,309,310,311,312,313,314,349,350,408,412,415,416,419,420,425,426,439
2	1.156	358.435	358.435	1.887	216,217,241,245,246,250,251,254,277,279,280,281,284,285,286,292,293,294,295,296,297,299,329,330,333,339,340
3	0.779	138.915	138.915	0.301	354,365,366,367,370,391,392,474,475,476,480,481,484,486,487,488
4	0.798	116.277	116.277	0.750	15,331,332,334,335,336,337,338,339,340,380,508,509,510,511,512,527
5	0.673	92.267	92.267	0.341	399,403,452,453,454,460,462,463,464,467,468,470,472,473

Table S2: Induced docking results at the selected allosteric site

Compound	IC_{50} (µM)	Docking Score	Glide energy	Glide Emodel	H-Bond residues
4b	24.87	-5.489	-30.792	-54.445	Arg312 and Glu408
4i	15.85	-6.938	-51.445	-80.251	His245, Asn241, Agr312, Tyr313 and Asn412
4j	26.67	-5.040	-42.085	-59.442	His239 and Arg312
4k	23.69	-6.498	-51.569	-83.536	Phe300 and Arg312
4l	25.23	-5.806	-48.401	-64.485	Lys155 and Gly160 and His239
12k	22.47	-6.757	-51.159	-92.087	His245, Arg312 and Arg439
12m	26.81	-4.961	-51.007	-80.735	His251, Ser281, Tyr286 and Phe333

S.3 Synthetic procedures

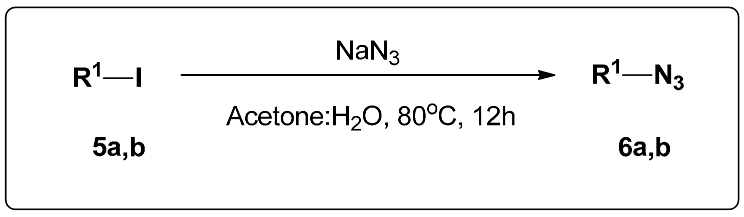

S.3.1 General procedure for the synthesis of alkyl azides 6a,b

Appropriate aliphatic halide **5a,b** (20mmol) was dissolved in a mixture of acetone:water (3:1) in a round bottom flask (RBF) and allowed to reflux for 15mins at 80 ºC. Subsequently, sodium azide (1.5eq) was added and the mixture refluxed for further 12h. Thereafter, the suspension was poured in water and extracted with ethyl acetate. Dried with anhydrous sodium sulphate and concentrated under reduced pressure to yield yellow oil.

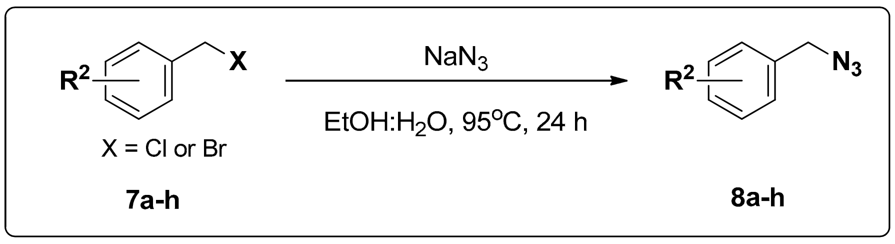

S.3.2 General procedure for the synthesis of benzyl azides 8a-h

A solution of relative benzyl halides **7a-h** (8mmol) and sodium azide 0.772g (1.5eq) in a mixture of ethanol:water (8:1) were allowed to stir at 95 ºC for 24h. At the end of the reaction after monitoring with TLC, water was added to the suspension and extracted with ethyl acetate. The combined organic layer was dried with anhydrous magnesium sulphate and dried under with a rotavapor at low temperature to obtain a yellow liquid.

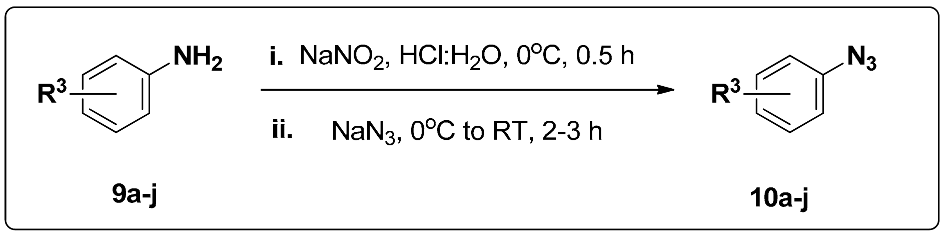

S.3.3 General procedure for the synthesis of phenyl azides 10a-j

Appropriate aniline 10mmol (1eq) was dissolved to a solution of concentrated HCl:water (1:1) in an RBF and stirred at 0-5 ºC. Thereafter, 15.4mmol (1.5eq) of sodium nitrite dissolved in water was added in small portion for 5mins and stirred for a further 30mins. Subsequent amount of sodium azide 19mmol (1.5eq) was added and stirred to rt for further 2-3h. The reaction was diluted with water and extracted with diethyl ether. The combined organic extracts were dried with anhydrous sodium sulphate and concentrated under reduced pressure to obtain crude azides at good yeilds.

S.4 Spectral data

ethyl 2-(quinolin-8-yloxy)acetate (**1**)

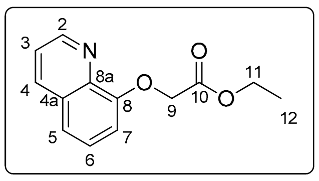

^{1}H NMR

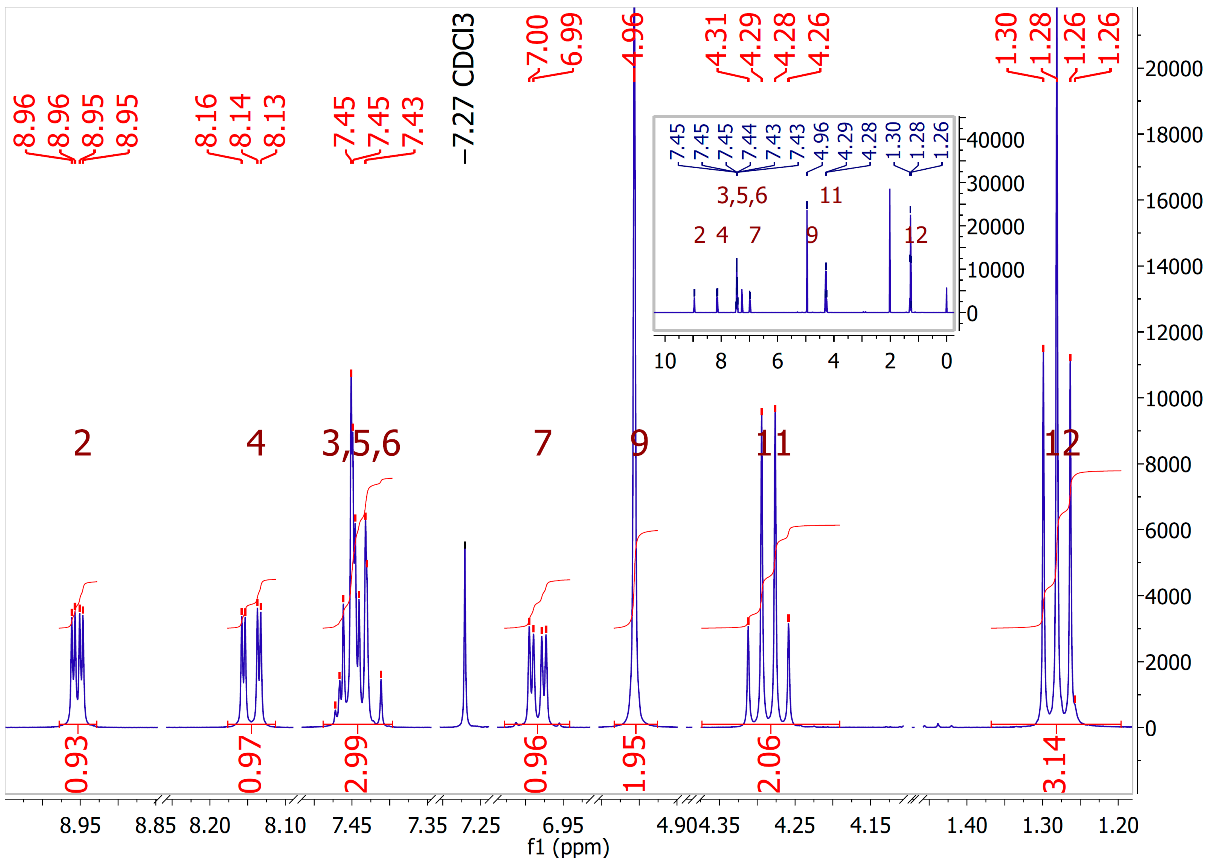

^{13}C NMR

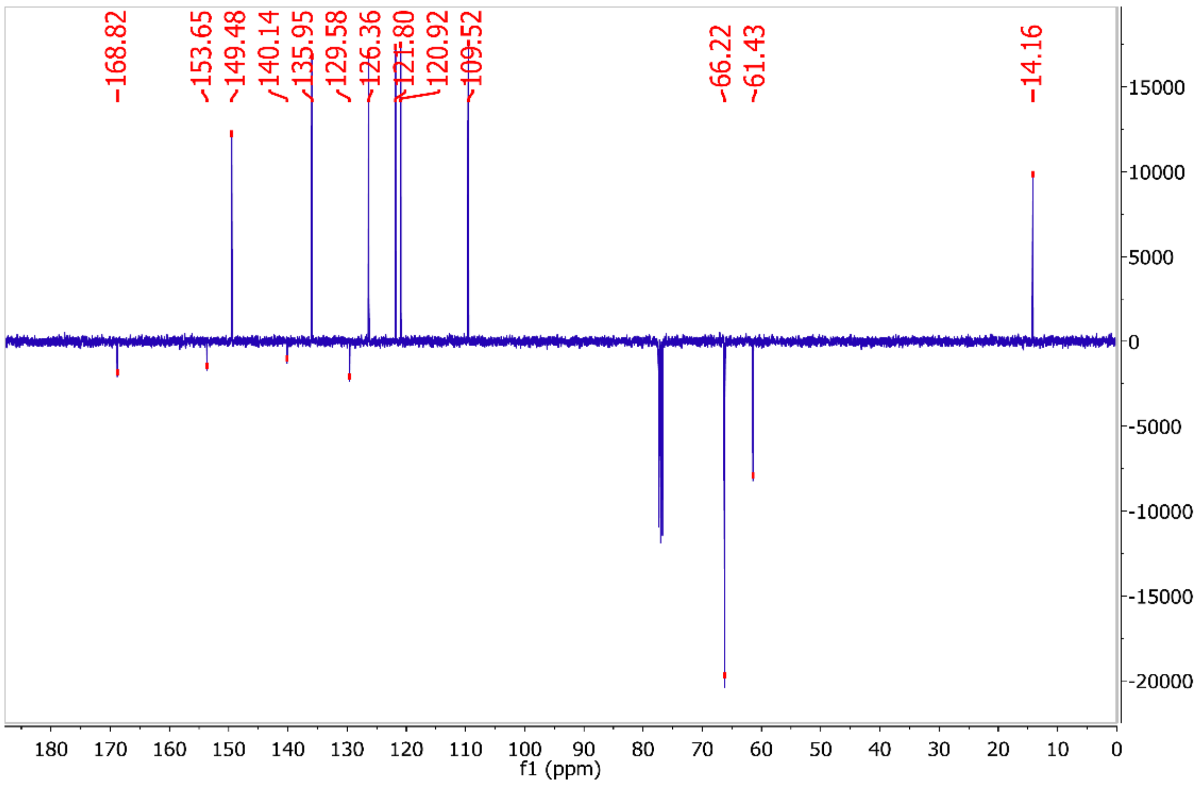

2-(quinolin-8-yloxy)acetohydrazide (**2**)

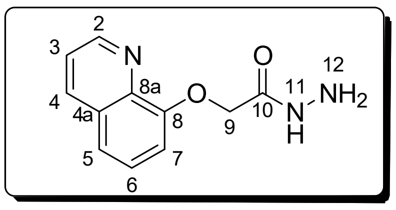

^{1}H NMR

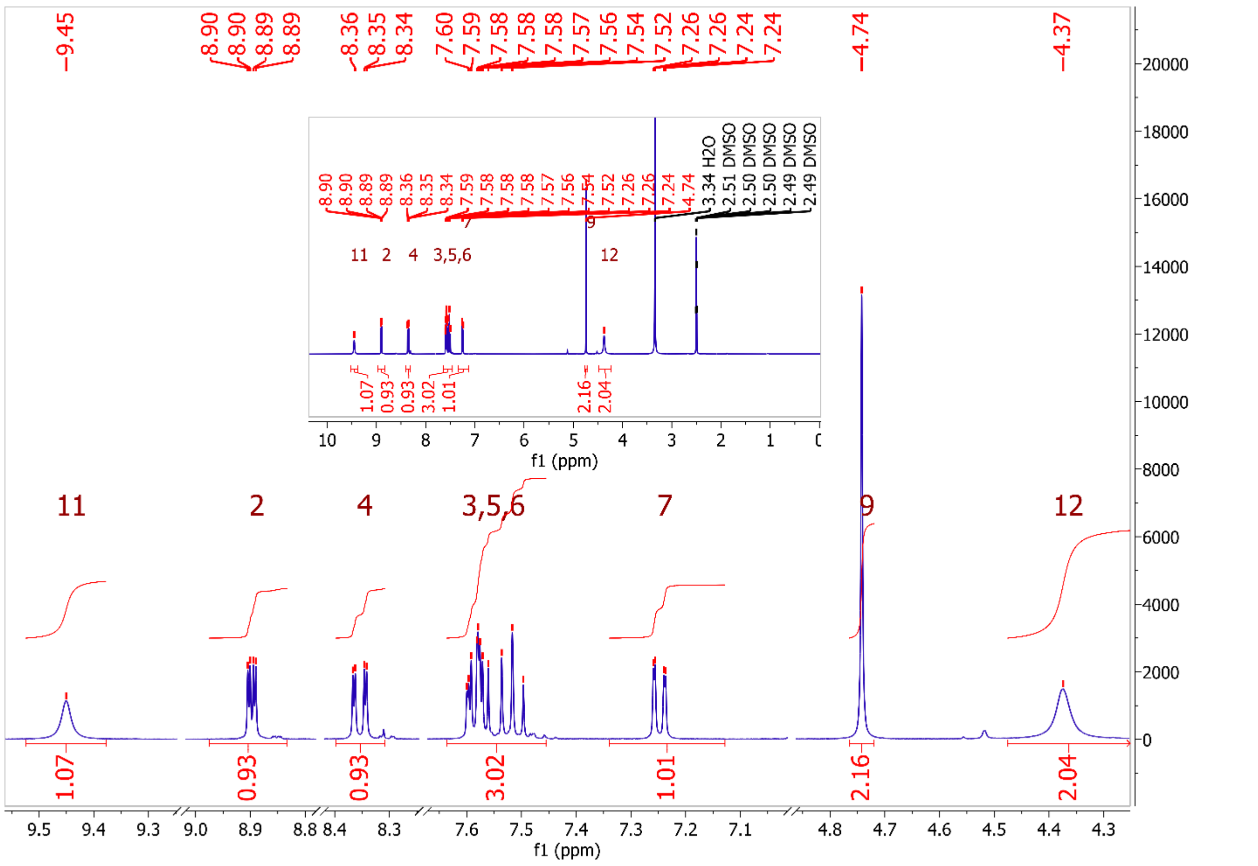

^{13}C NMR

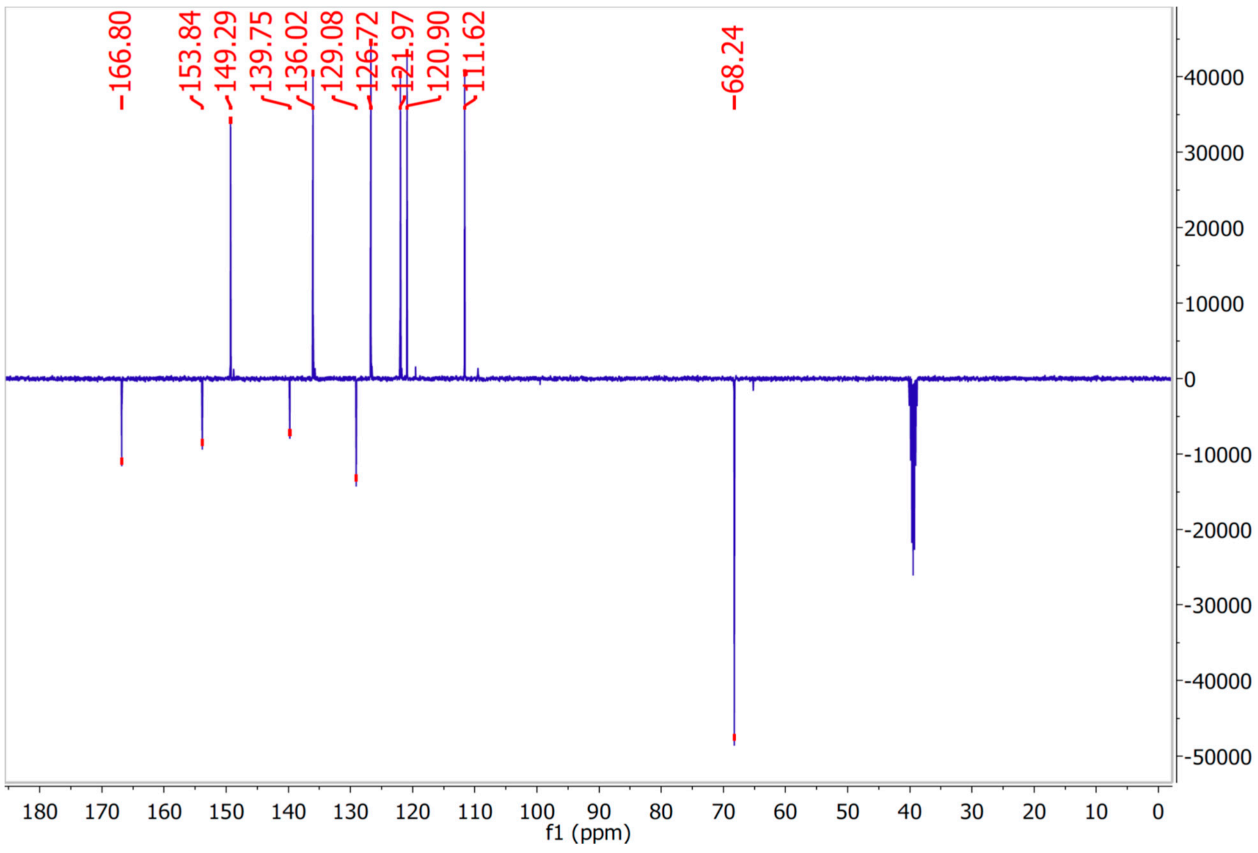

5-[(quinolin-8-yloxy)methyl]-1,3,4-oxadiazole-2-thiol (**3**)

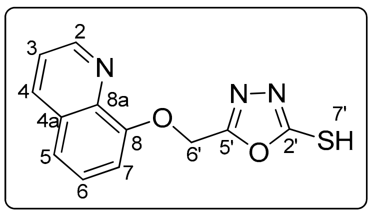

^{1}H NMR

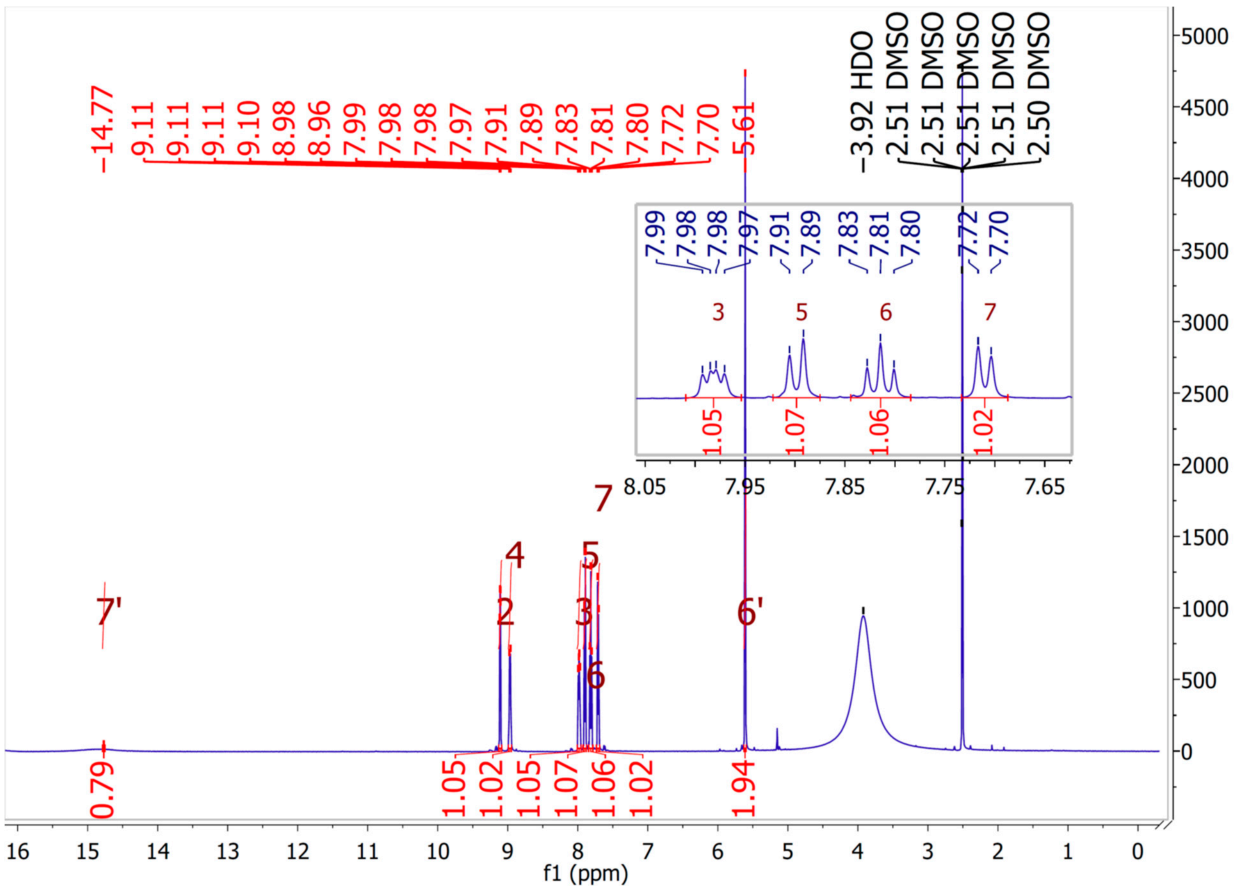

^{13}C NMR

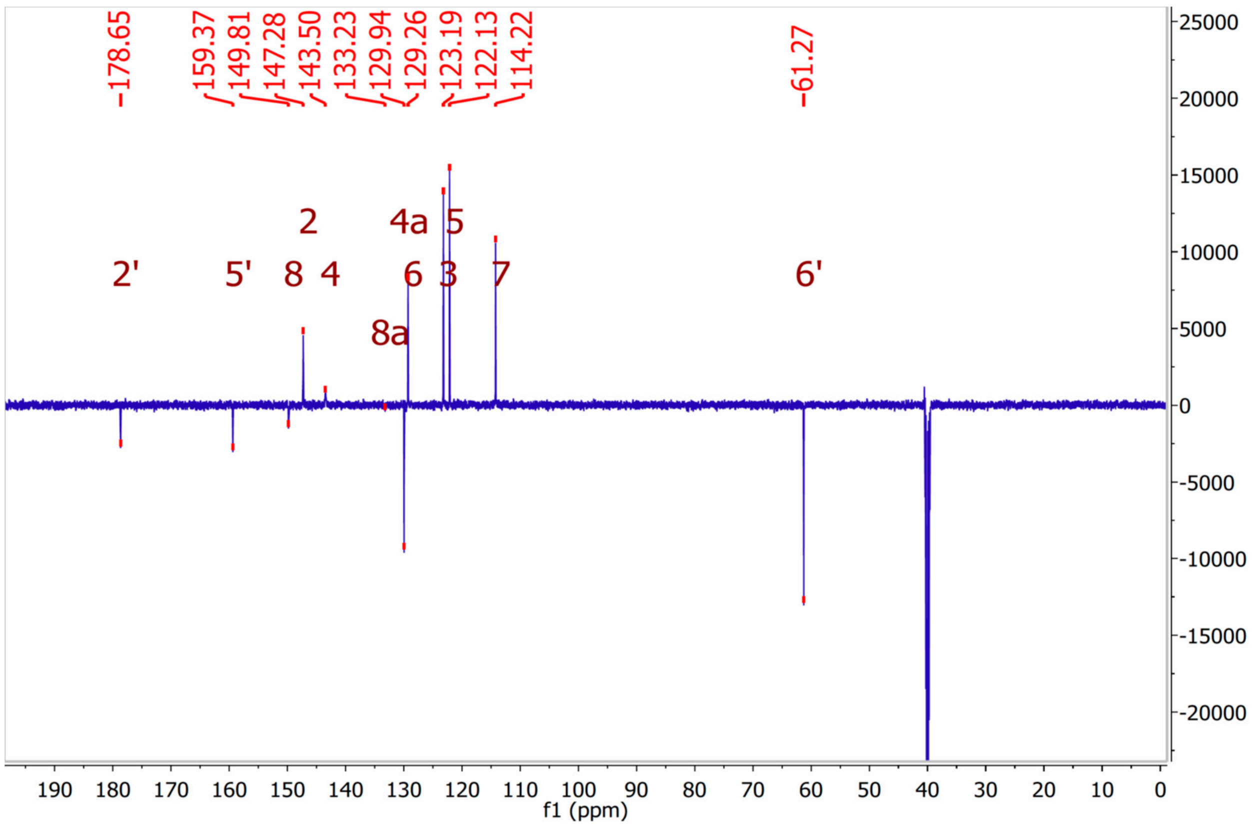

HRMS

Elemental Composition Report _Compound 3

Single Mass Analysis
Tolerance = 5.0 PPM / DBE: min = -1.5, max = 500.0
Element prediction: Off
Number of isotope peaks used for i-FIT = 3

Monoisotopic Mass, Even Electron Ions
33 formula(e) evaluated with 1 results within limits (all results (up to 1000) for each mass) Elements Used:
C: 10-15 H: 5-10 N: 0-5 O: 0-5 S: 0-1

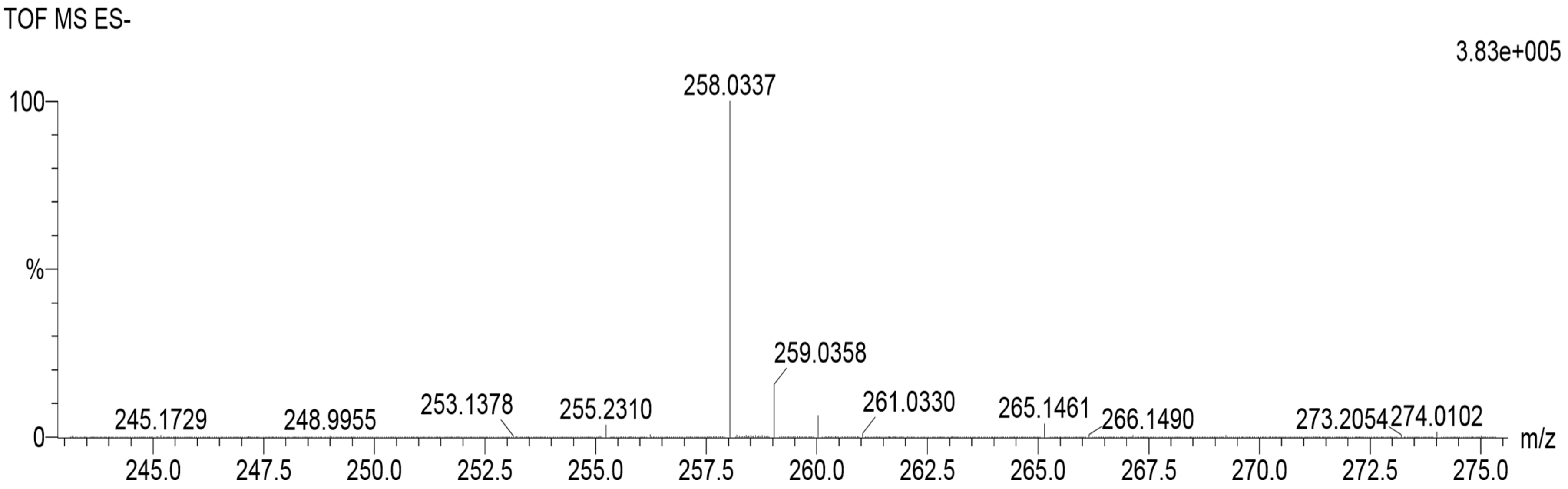

Minimum:				-1.5			
Maximum:		5.0	5.0	500.0			
Mass	Calc. Mass	mDa	PPM	DBE	i-FIT	i-FIT (Norm)	Formula
258.0337	258.0337	0.0	0.0	10.5	647.6	0.0	C12 H8 N3 O2 S

2-(ethylthio)-5-[(quinolin-8-yloxy)methyl]-1,3,4-oxadiazole (**4a**)

^{1}H NMR

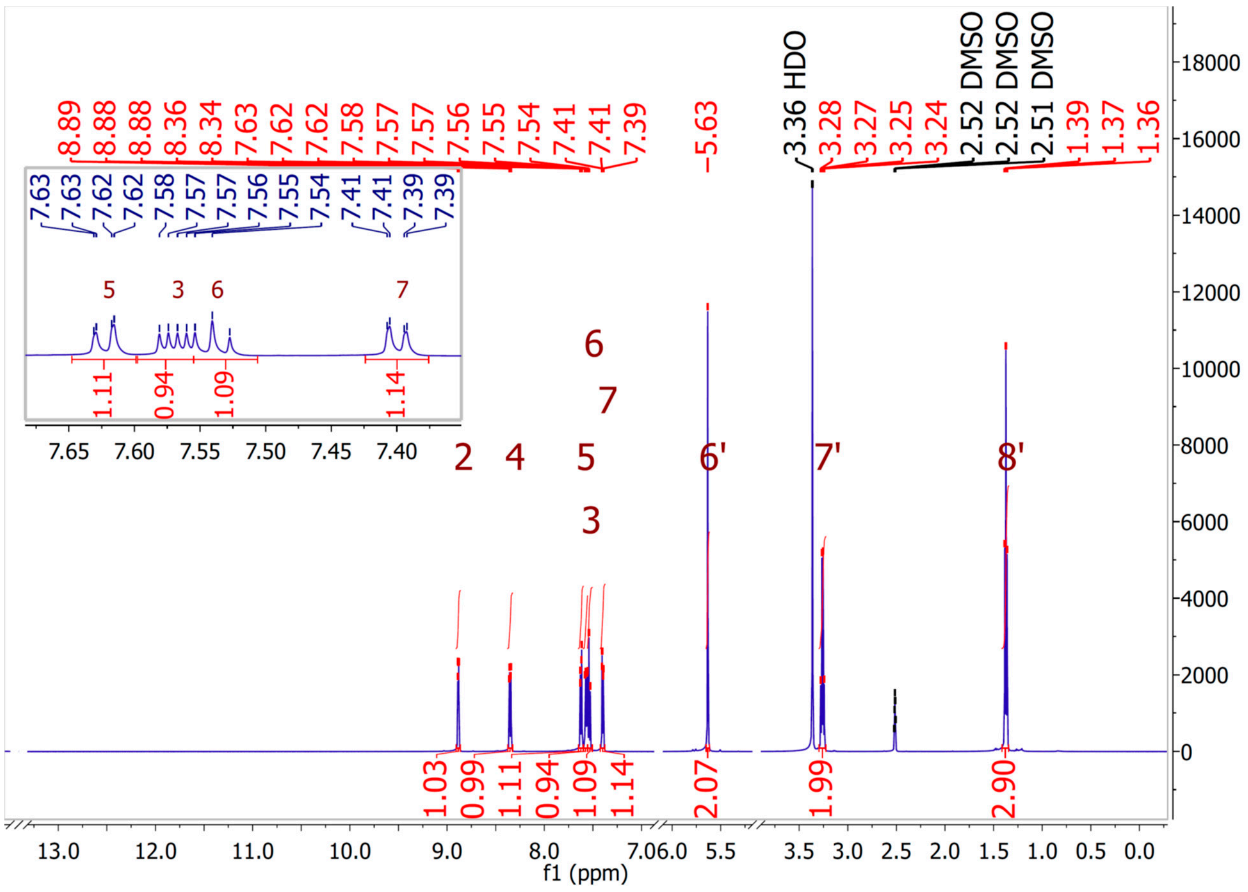

^{13}C NMR

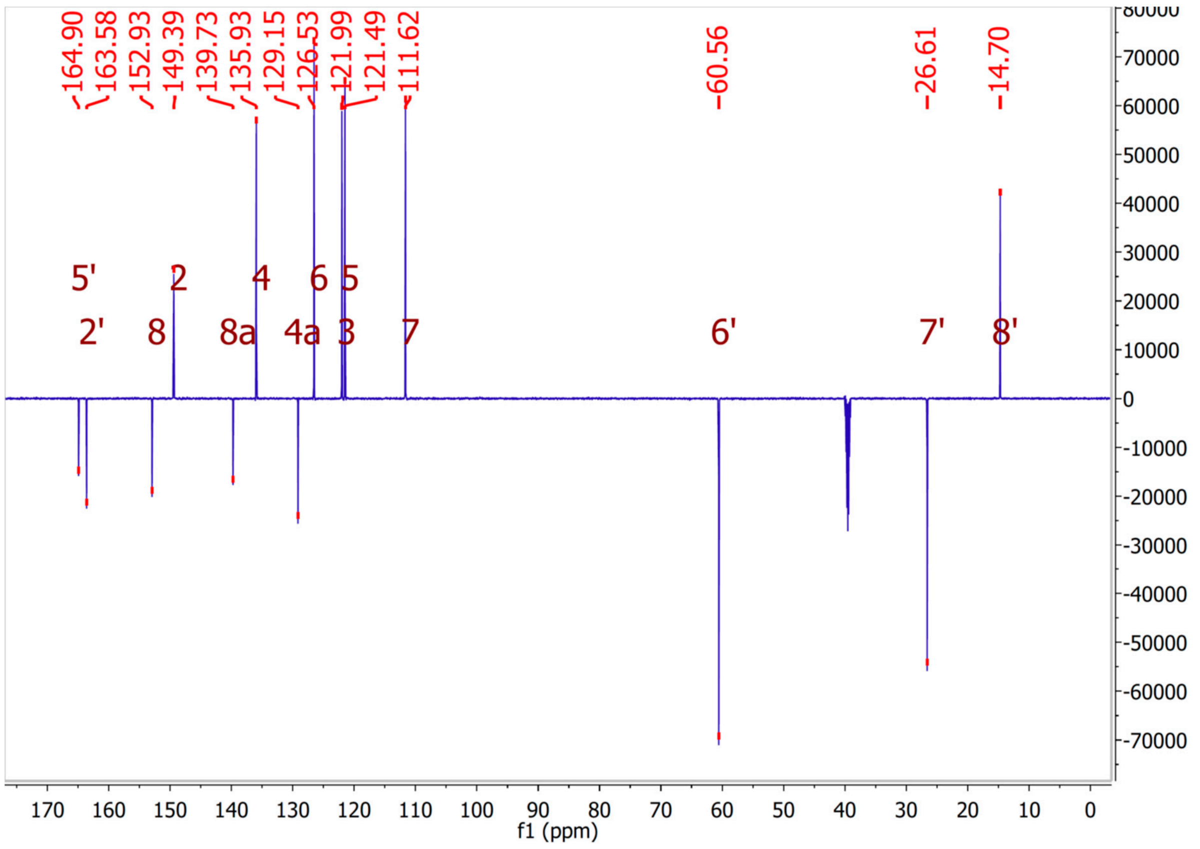

HRMS

Elemental Composition Report_ Compound 4a

Single Mass Analysis

Tolerance = 5.0 PPM / DBE: min = -1.5, max = 500.0
Element prediction: Off
Number of isotope peaks used for i-FIT = 3

Monoisotopic Mass, Even Electron Ions
74 formula(e) evaluated with 1 results within limits (all results (up to 1000) for each mass) Elements Used:
C: 10-15 H: 10-15 N: 0-5 O: 0-5 S: 0-1 Na: 0-1

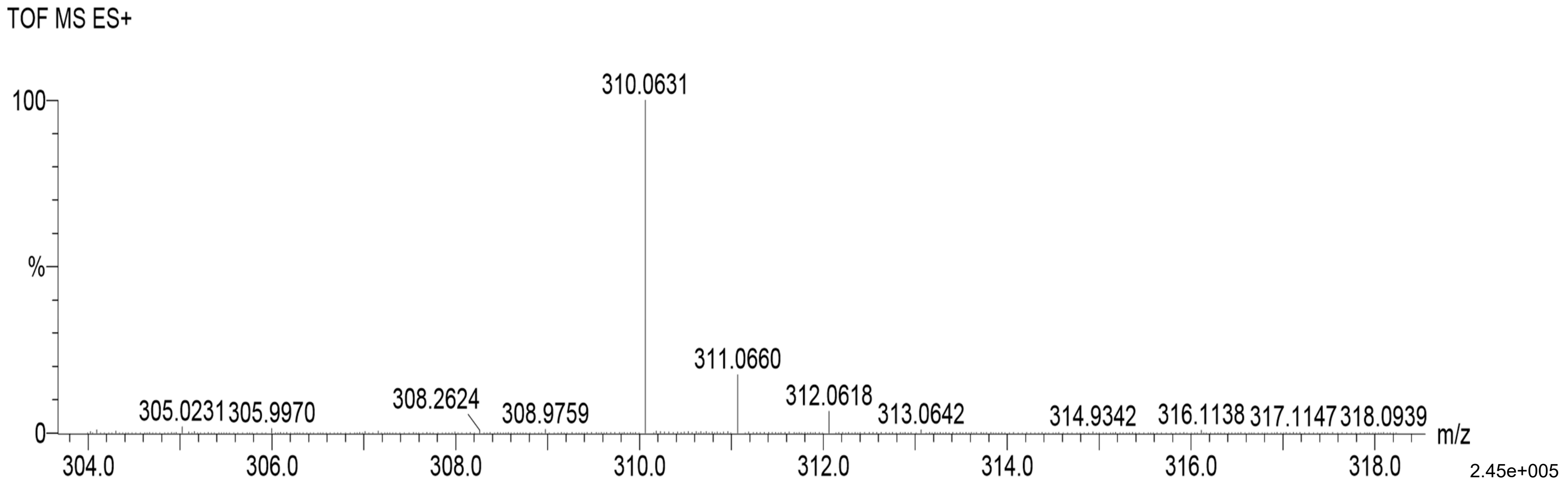

Minimum:				-1.5			
Maximum:		5.0	5.0	500.0			
Mass	Calc. Mass	mDa	PPM	DBE	i-FIT	i-FIT (Norm)	Formula
310.0631	310.0626	0.5	1.6	9.5	653.9	0.0	C14 H13 N3 O2 S Na

2-(propylthio)-5-[(quinolin-8-yloxy)methyl]-1,3,4-oxadiazole (**4b**)

^{1}H NMR

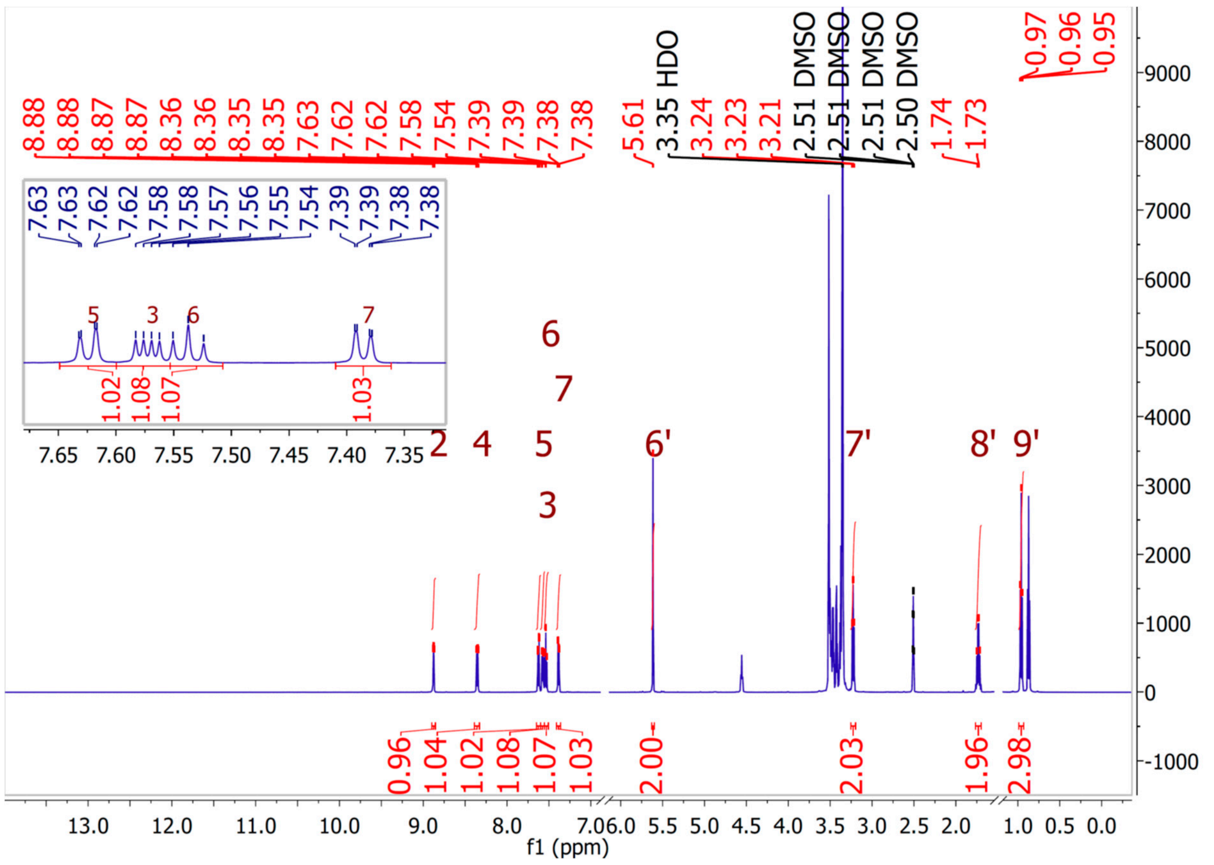

^{13}C NMR

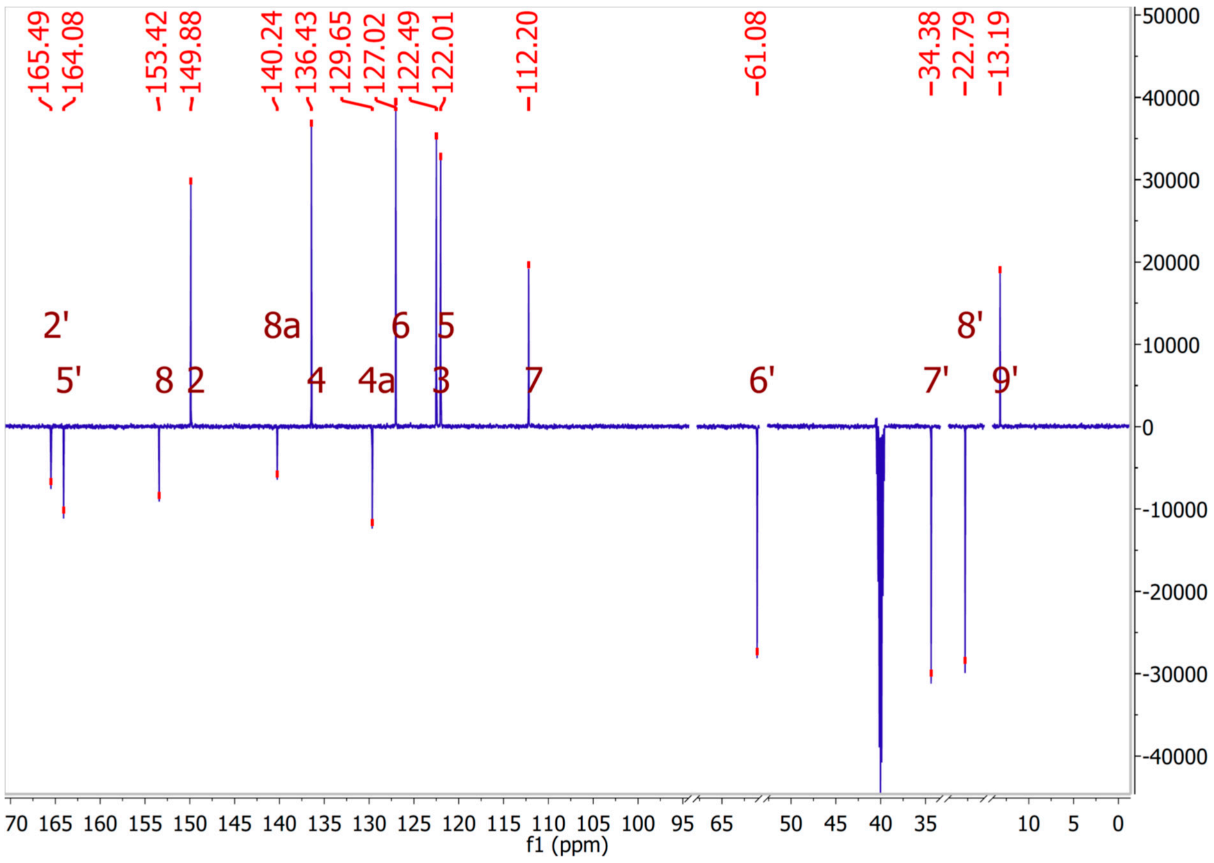

HRMS

Elemental Composition Report_Compound 4b

Single Mass Analysis

Tolerance = 5.0 PPM / DBE: min = -1.5, max = 500.0
Element prediction: Off
Number of isotope peaks used for i-FIT = 3

Monoisotopic Mass, Even Electron Ions
86 formula(e) evaluated with 1 results within limits (all results (up to 1000) for each mass) Elements Used:
C: 10-15 H: 10-15 N: 0-5 O: 0-5 Na: 0-1 S: 0-1

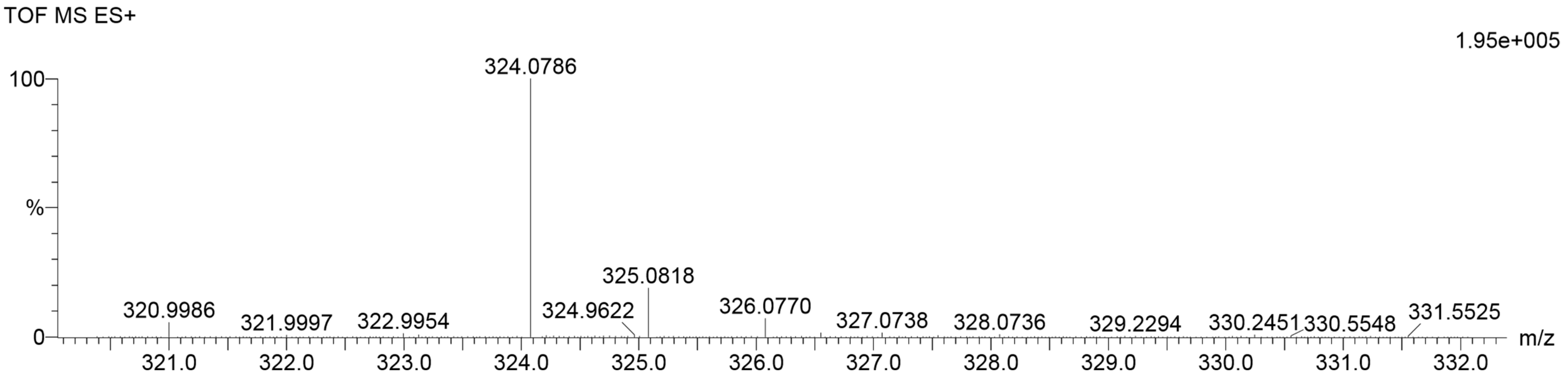

	Mass	Calc. Mass	mDa	PPM	DBE	i-FIT	i-FIT (Norm)	Formula
Minimum:					-1.5			
Maximum:			5.0	5.0	500.0			
	324.0786	324.0783	0.3	0.9	9.5	640.5	0.0	C15 H15 N3 O2 Na S

2-(butylthio)-5-[(quinolin-8-yloxy)methyl]-1,3,4-oxadiazole (**4c**)

^{1}H NMR

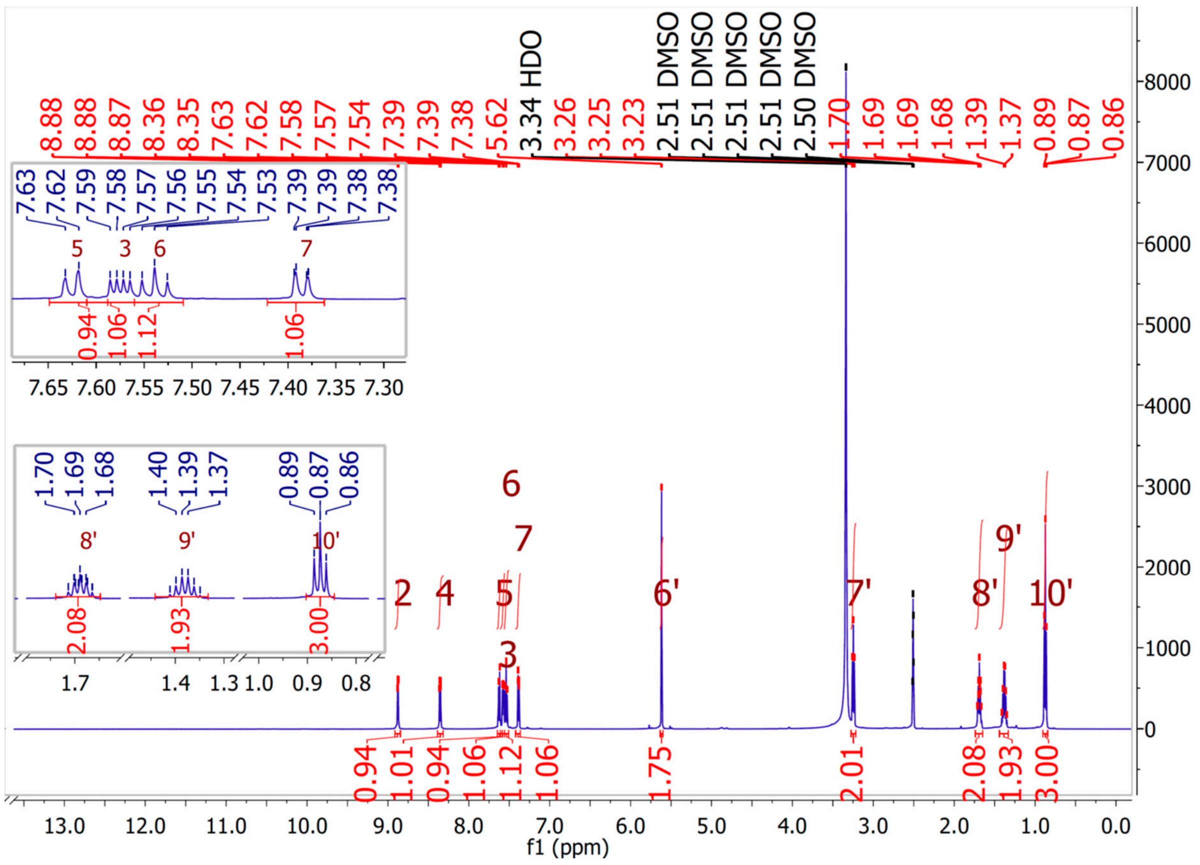

^{13}C NMR

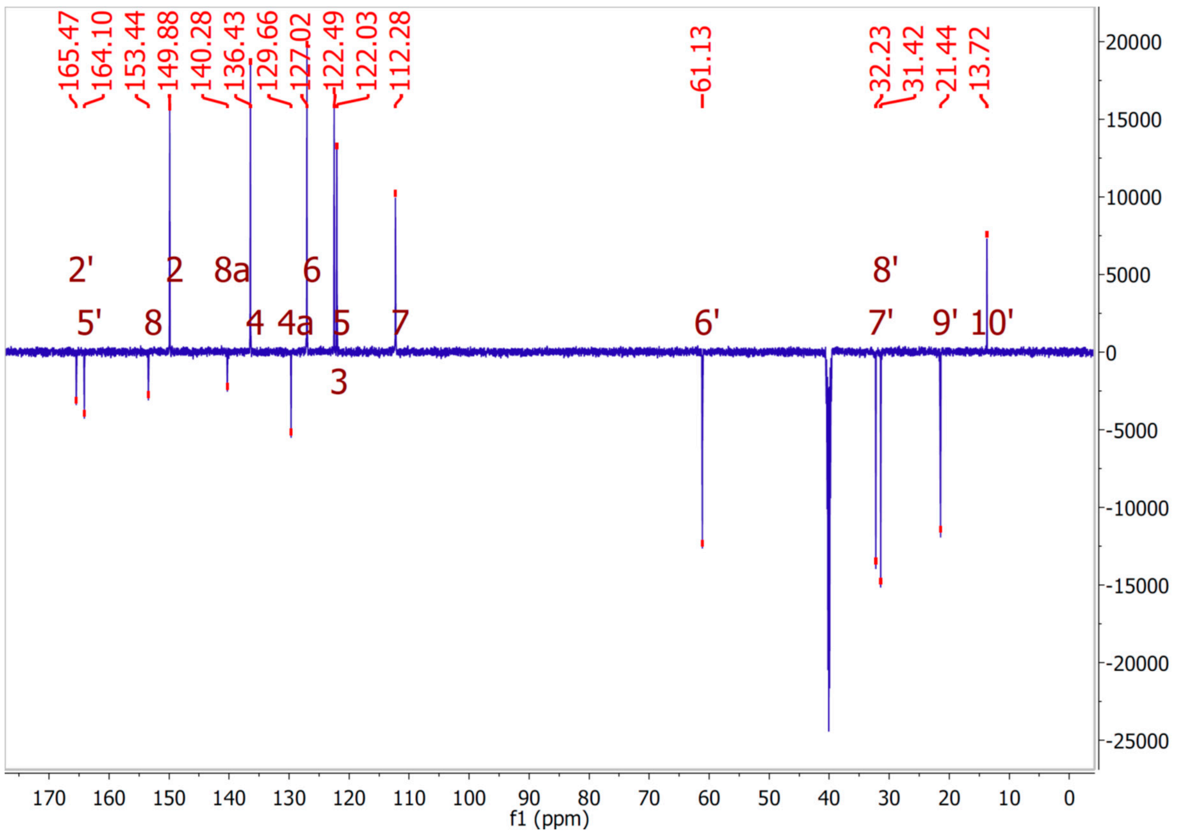

HSQC

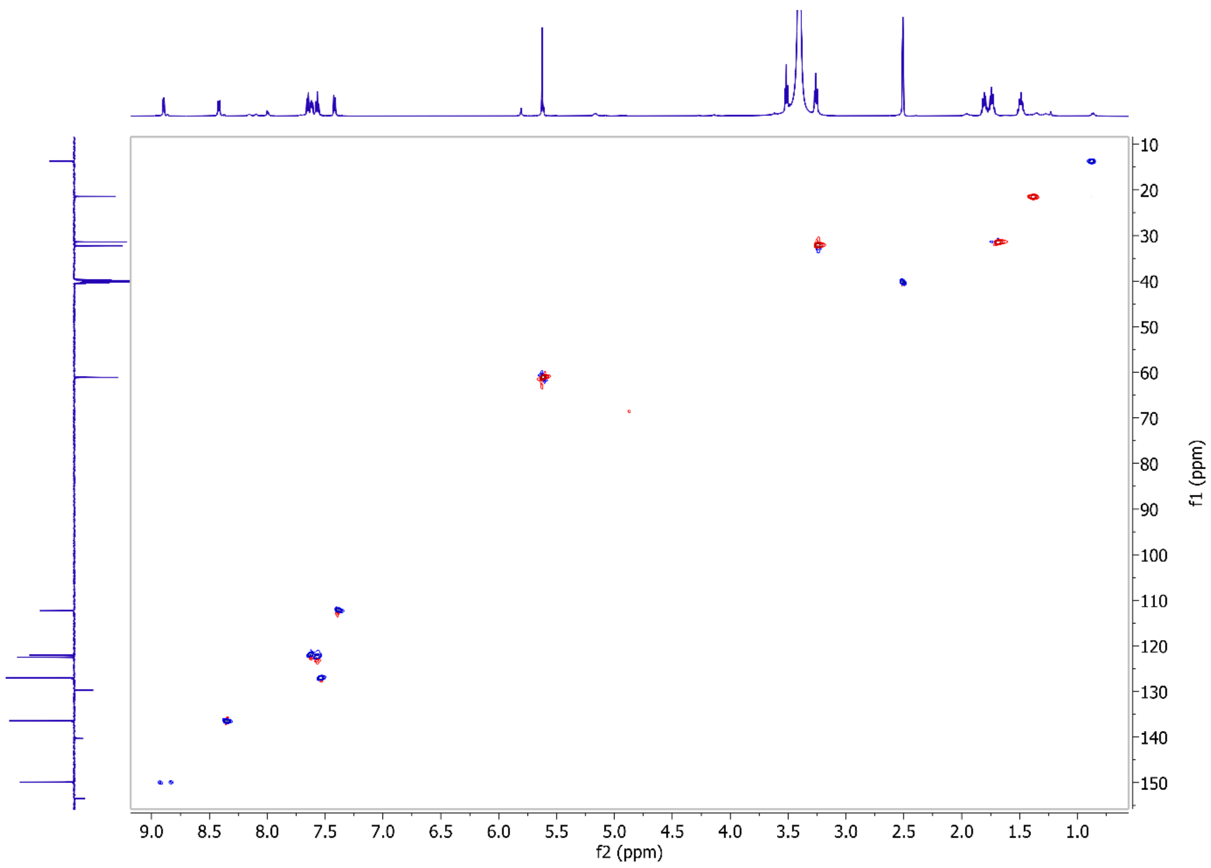

HMBC

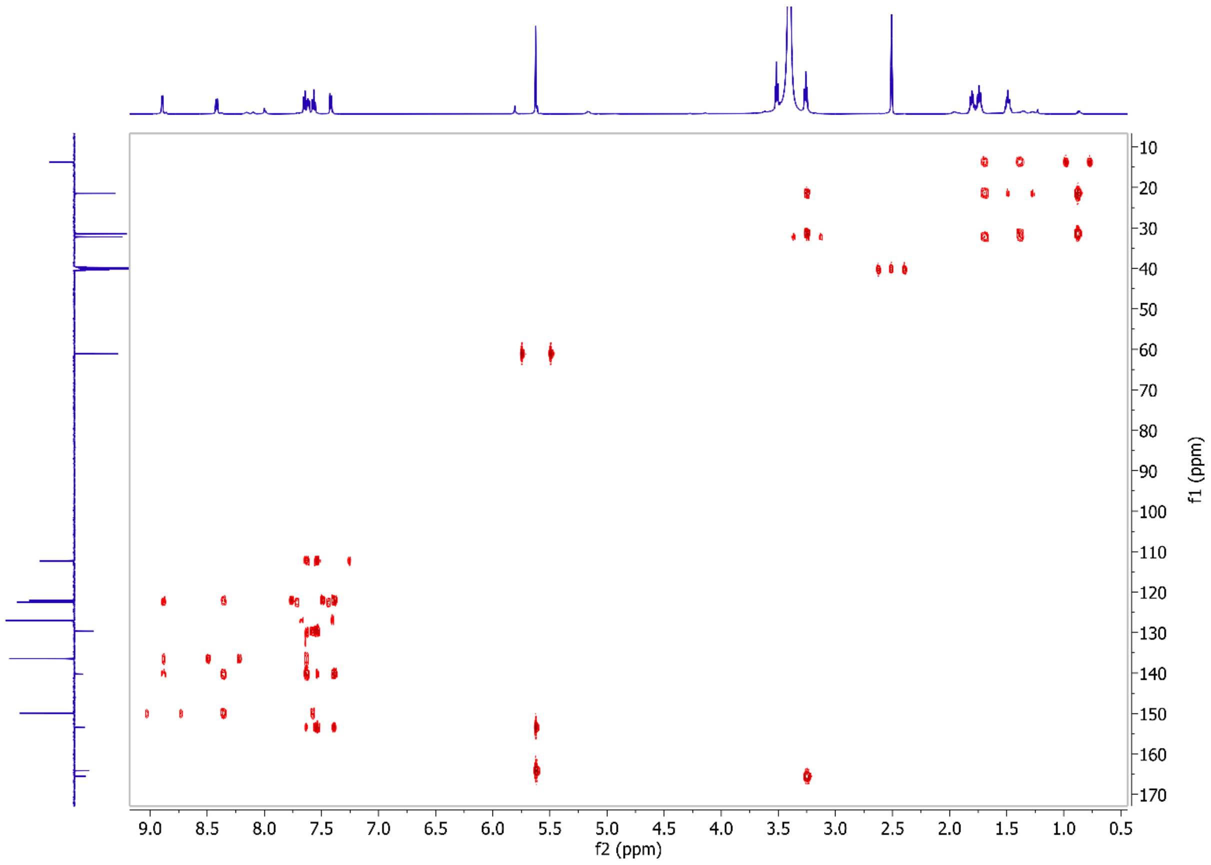

HRMS

Elemental Composition Report_Comound 4c

Single Mass Analysis

Tolerance = 5.0 PPM / DBE: min = -1.5, max = 500.0
Element prediction: Off
Number of isotope peaks used for i-FIT = 3

Monoisotopic Mass, Even Electron Ions
62 formula(e) evaluated with 1 results within limits (all results (up to 1000) for each mass) Elements Used:
C: 15-20 H: 15-20 N: 0-5 O: 0-5 Na: 0-1 S: 0-1

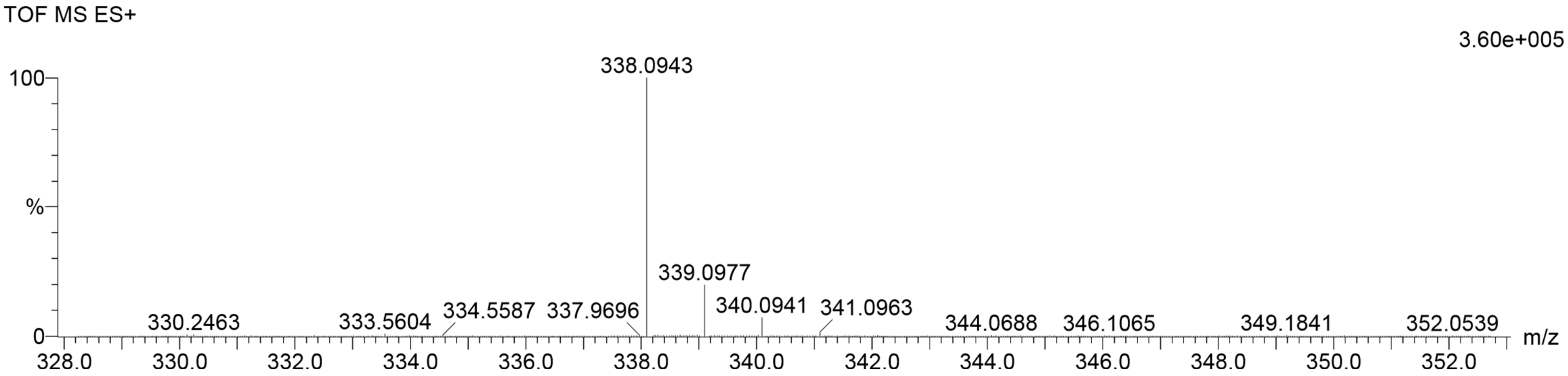

Minimum:				-1.5			
Maximum:		5.0	5.0	500.0			
Mass	Calc. Mass	mDa	PPM	DBE	i-FIT	i-FIT (Norm)	Formula
338.0943	338.0939	0.4	1.2	9.5	662.4	0.0	C16 H17 N3 O2 Na S

2-(pentylthio)-5-[(quinolin-8-yloxy)methyl]-1,3,4-oxadiazole (**4d**)

^{1}H NMR

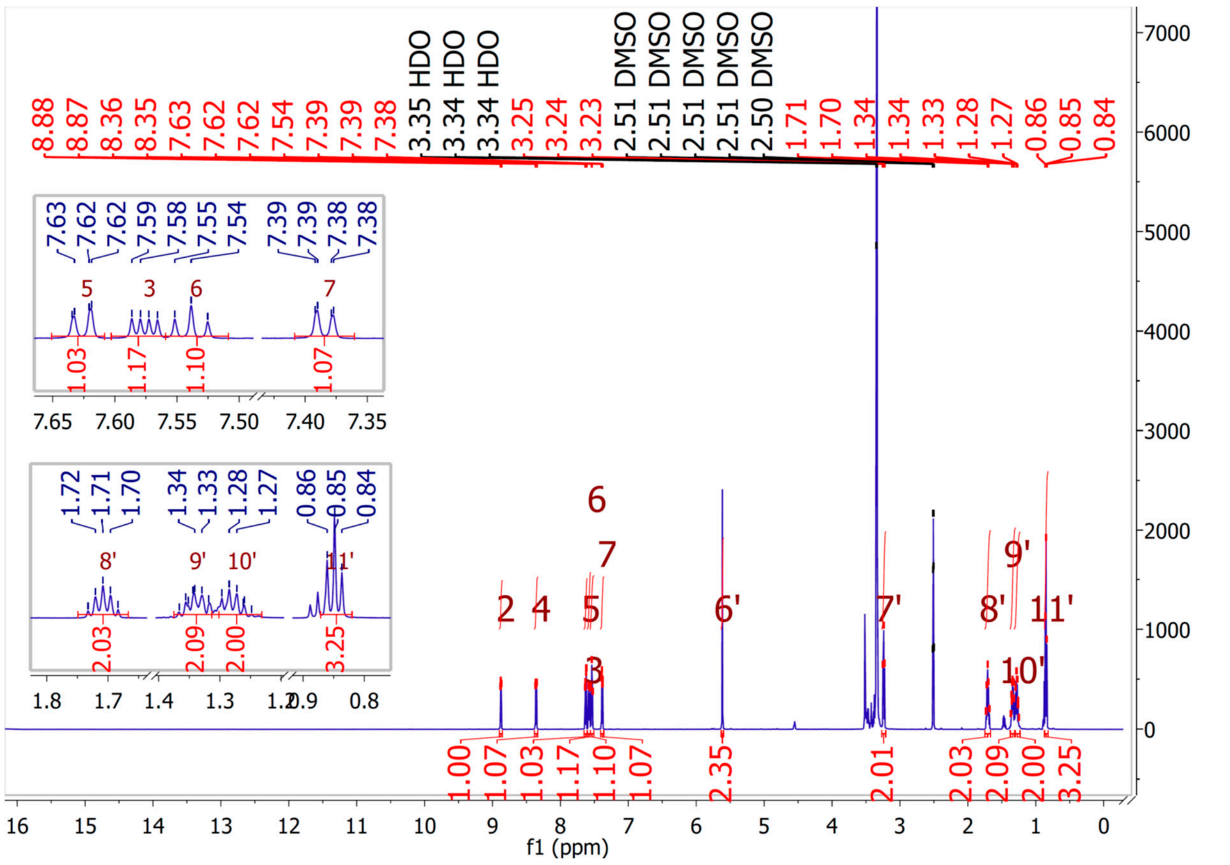

^{13}C NMR

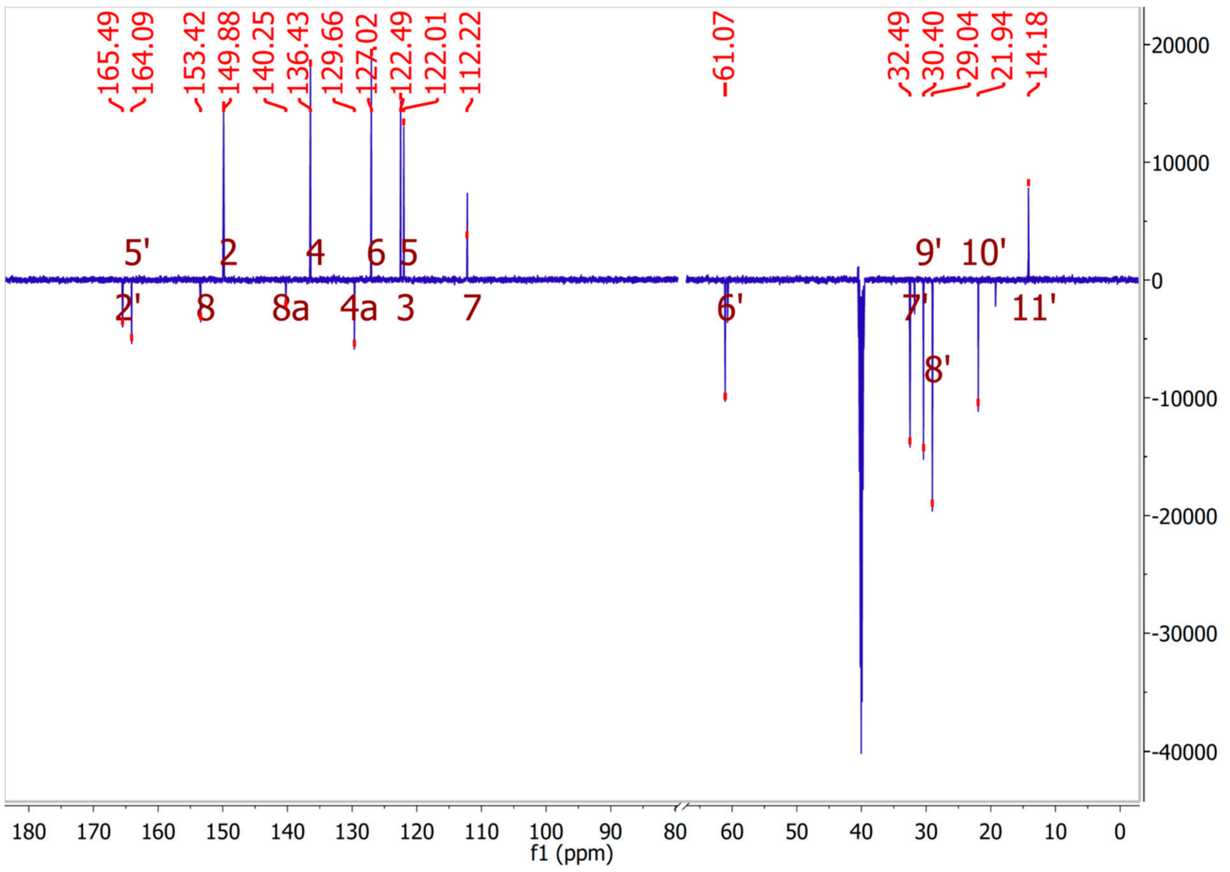

HRMS

Elemental Composition Report_Compound 4d

Single Mass Analysis

Tolerance = 5.0 PPM / DBE: min = -1.5, max = 500.0
Element prediction: Off
Number of isotope peaks used for i-FIT = 3

Monoisotopic Mass, Even Electron Ions
59 formula(e) evaluated with 1 results within limits (all results (up to 1000) for each mass) Elements Used:
C: 15-20 H: 15-20 N: 0-5 O: 0-5 Na: 0-1 S: 0-1

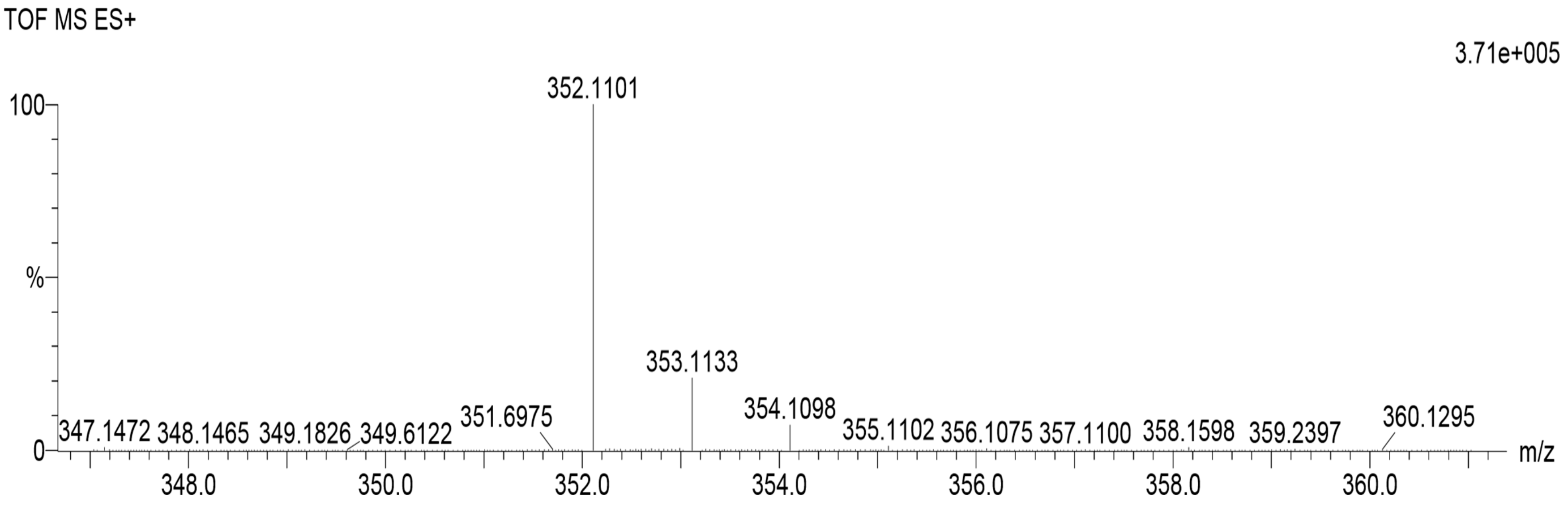

Minimum:				-1.5			
Maximum:		5.0	5.0	500.0			
Mass	Calc. Mass	mDa	PPM	DBE	i-FIT	i-FIT (Norm)	Formula
352.1101	352.1096	0.5	1.4	9.5	667.7	0.0	C17 H19 N3 O2 Na

2-(hexylthio)-5-[(quinolin-8-yloxy)methyl]-1,3,4-oxadiazole (**4e**)

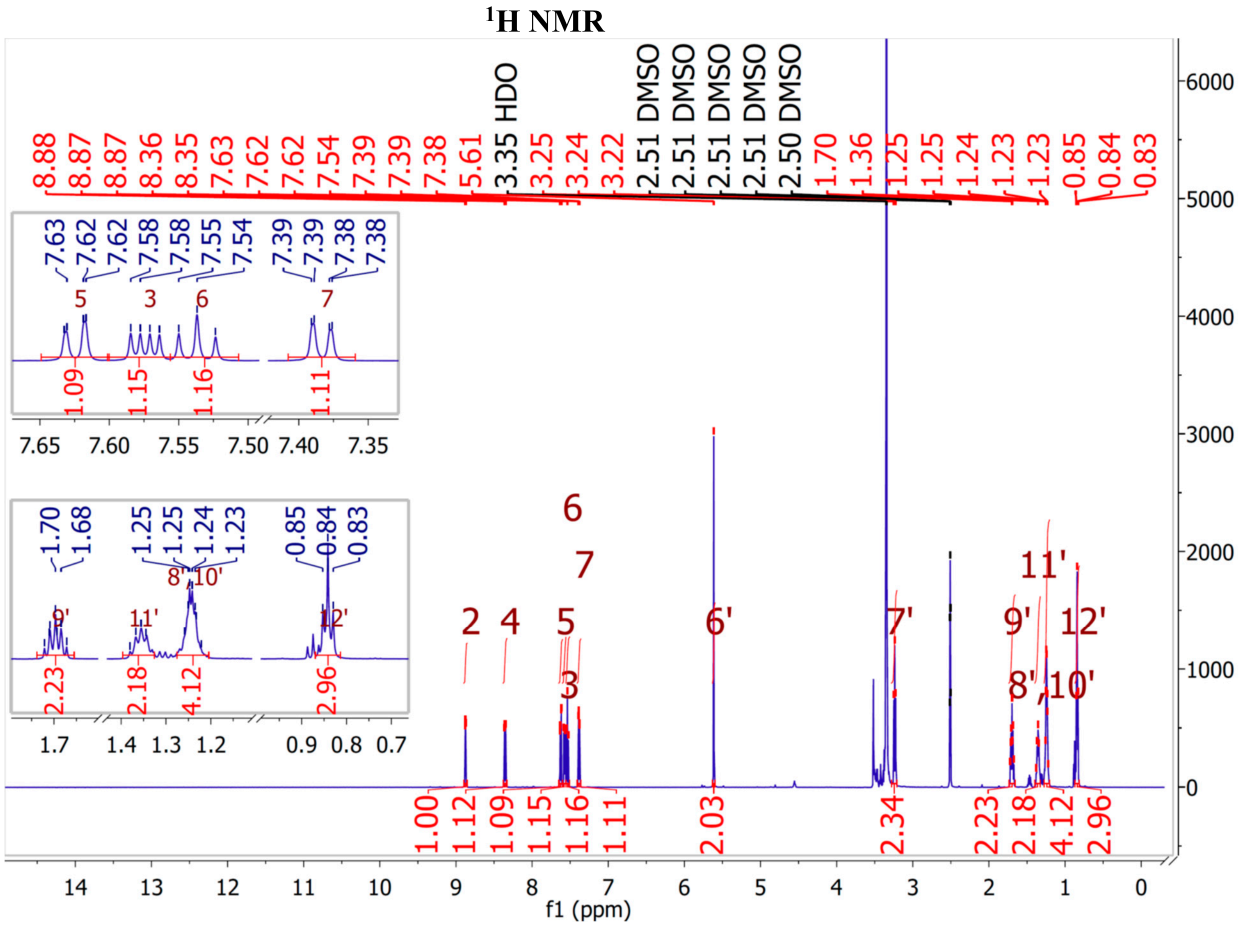

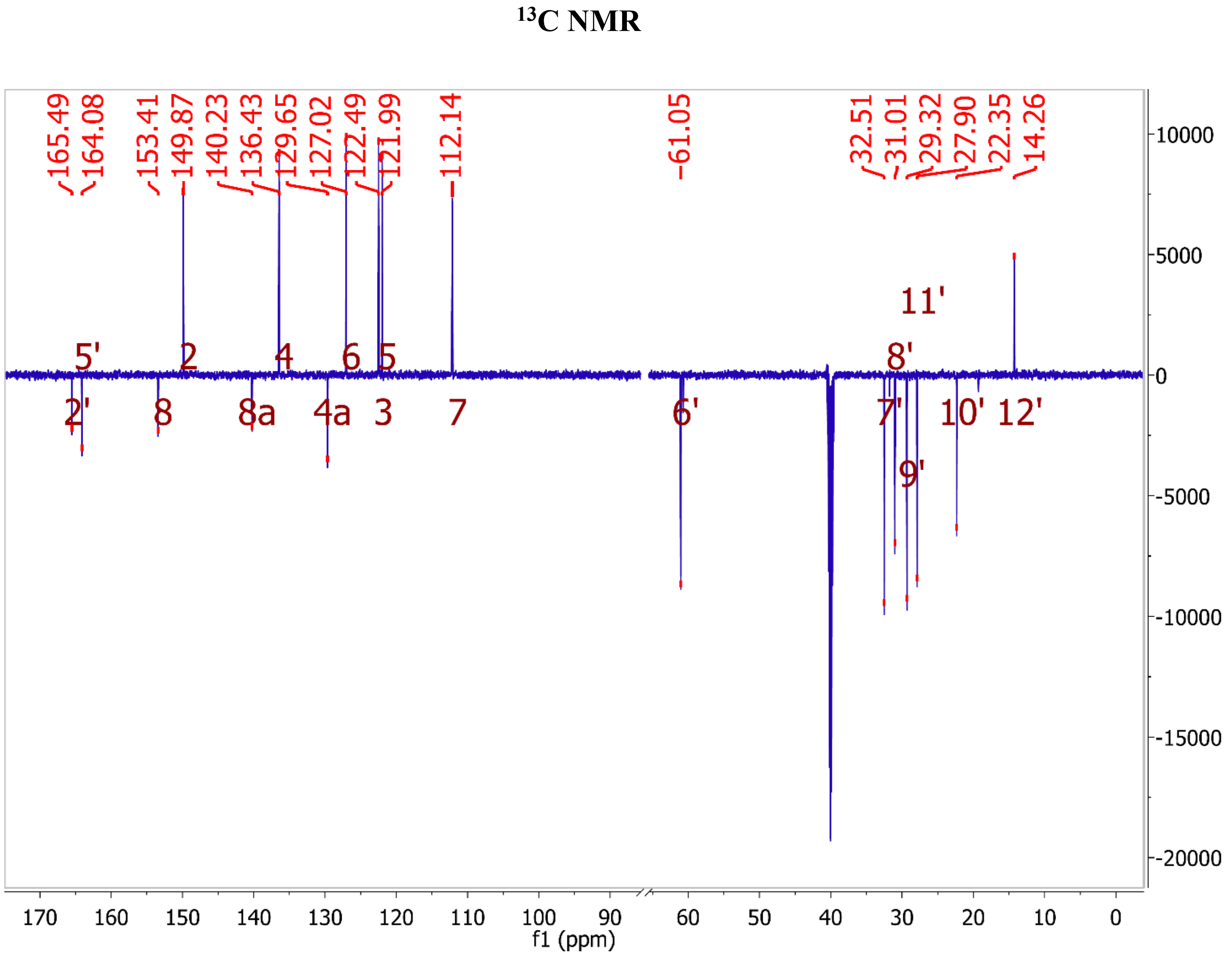

HRMS

Elemental Composition Report

Single Mass Analysis_Compound 4e

Tolerance = 5.0 PPM / DBE: min = -1.5, max = 500.0
Element prediction: Off
Number of isotope peaks used for i-FIT = 3

Monoisotopic Mass, Even Electron Ions
29 formula(e) evaluated with 1 results within limits (all results (up to 1000) for each mass) Elements Used:
C: 15-20 H: 20-25 N: 0-5 O: 0-5 Na: 1-1 S: 0-1

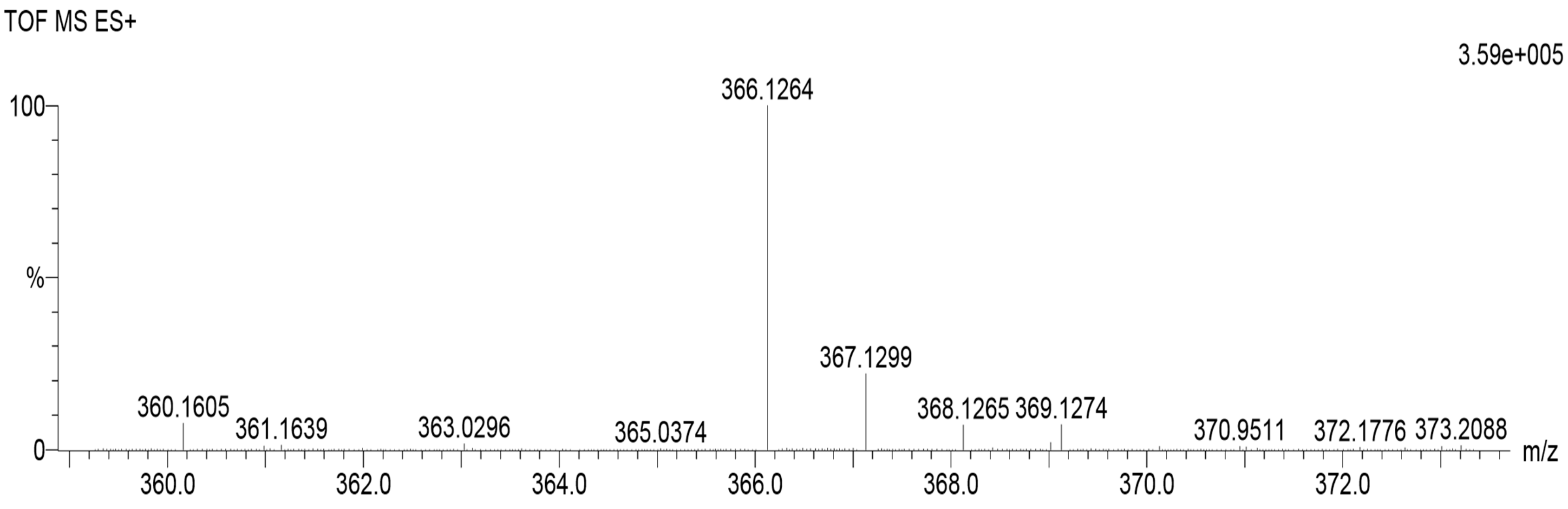

Minimum:				-1.5			
Maximum:		5.0	5.0	500.0			
Mass	Calc. Mass	mDa	PPM	DBE	i-FIT	i-FIT (Norm)	Formula
366.1252	366.1252	1.2	3.3	9.5	644.4	0.0	C18 H21 N3 O2 Na S

2-(allylthio)-5-[(quinolin-8-yloxy)methyl]-1,3,4-oxadiazole (**4f**)

^{1}H NMR

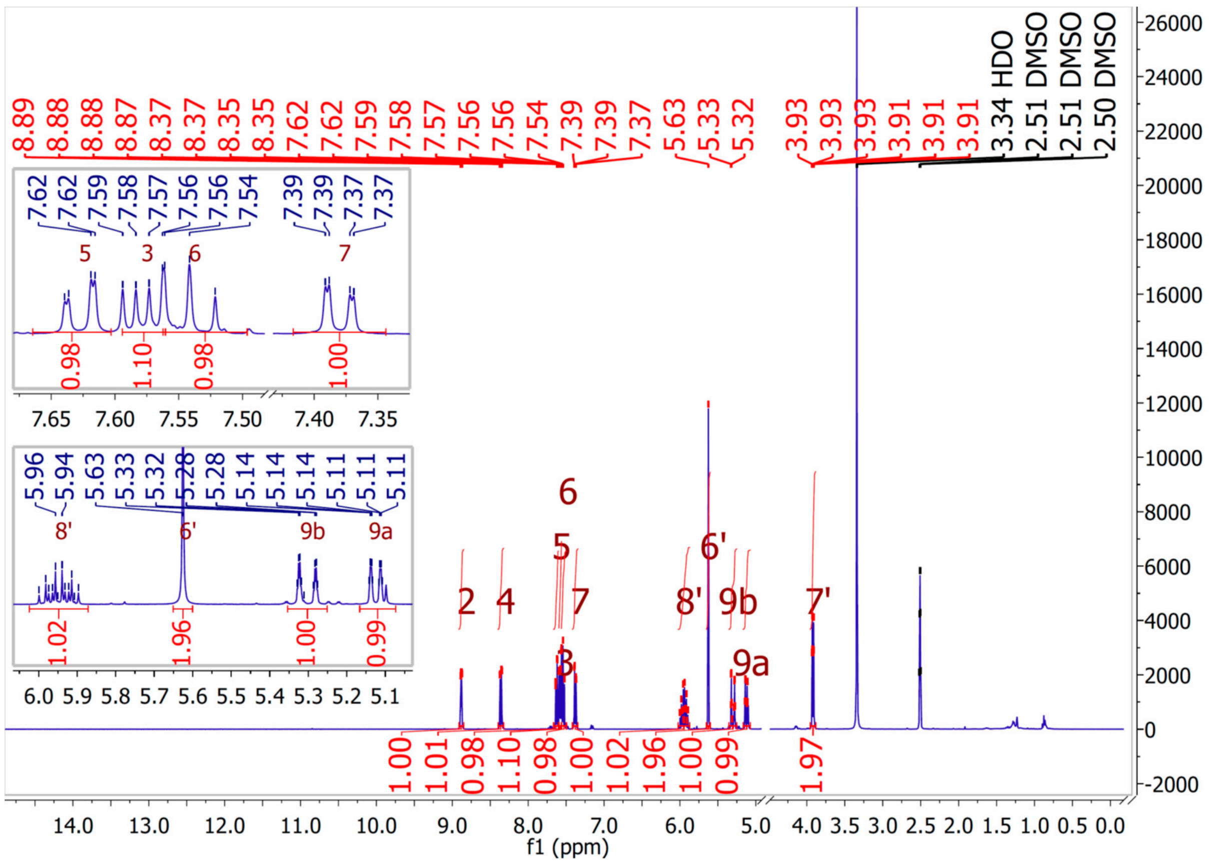

^{13}C NMR

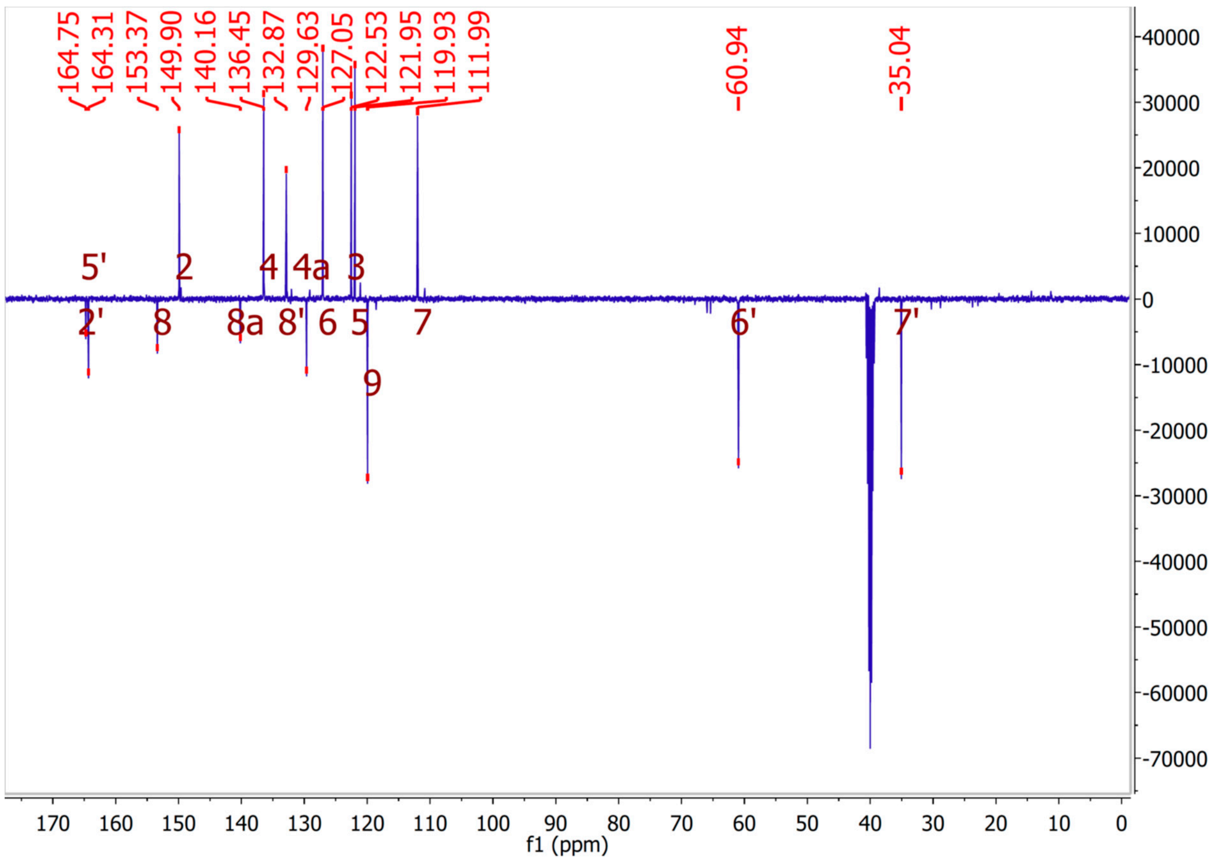

HRMS

Elemental Composition Report_Compound 4f

Single Mass Analysis

Tolerance = 5.0 PPM / DBE: min = -1.5, max = 500.0
Element prediction: Off
Number of isotope peaks used for i-FIT = 3

Monoisotopic Mass, Even Electron Ions
39 formula(e) evaluated with 1 results within limits (all results (up to 1000) for each mass) Elements Used:
C: 15-20 H: 10-15 N: 0-5 O: 0-5 Na: 1-1 S: 0-1

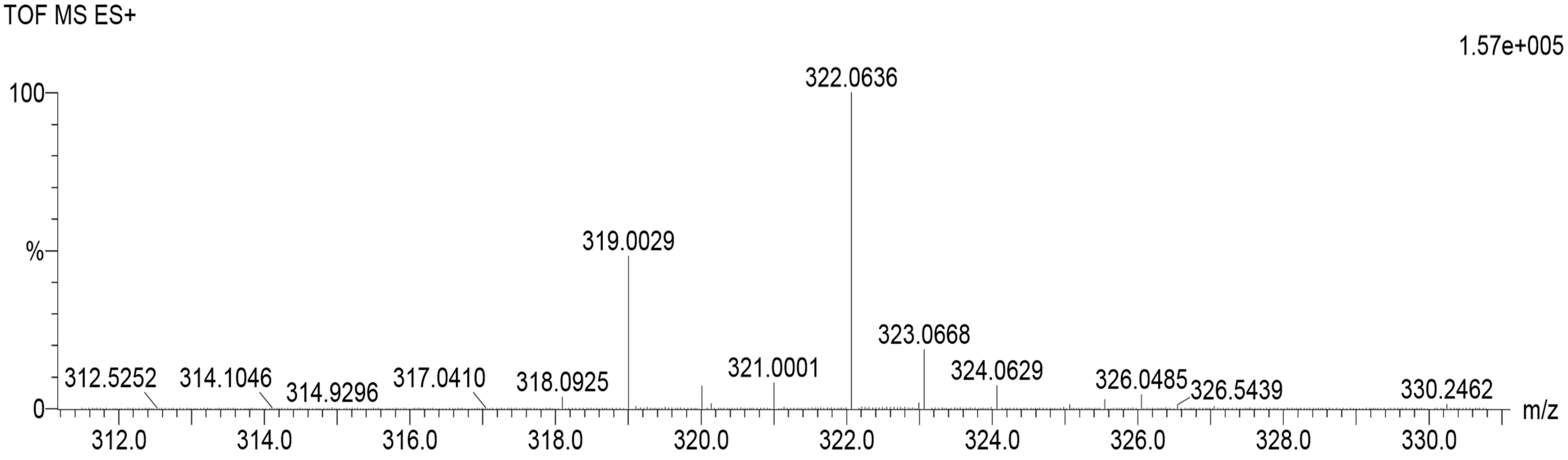

Minimum:				-1.5			
Maximum:		5.0	5.0	500.0			
Mass	Calc. Mass	mDa	PPM	DBE	i-FIT	i-FIT (Norm)	Formula
322.0636	322.0626	1.0	3.1	10.5	619.2	0.0	C15 H13 N3 O2 Na S

2-[(3-methylbut-2-en-1-yl)thio]-5-[(quinolin-8-yloxy)methyl]-1,3,4-oxadiazole (**4g**)

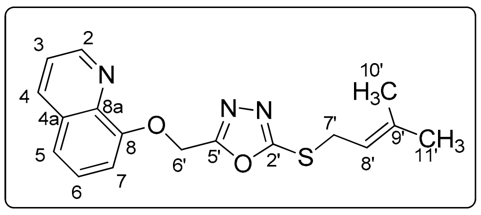

^{1}H NMR

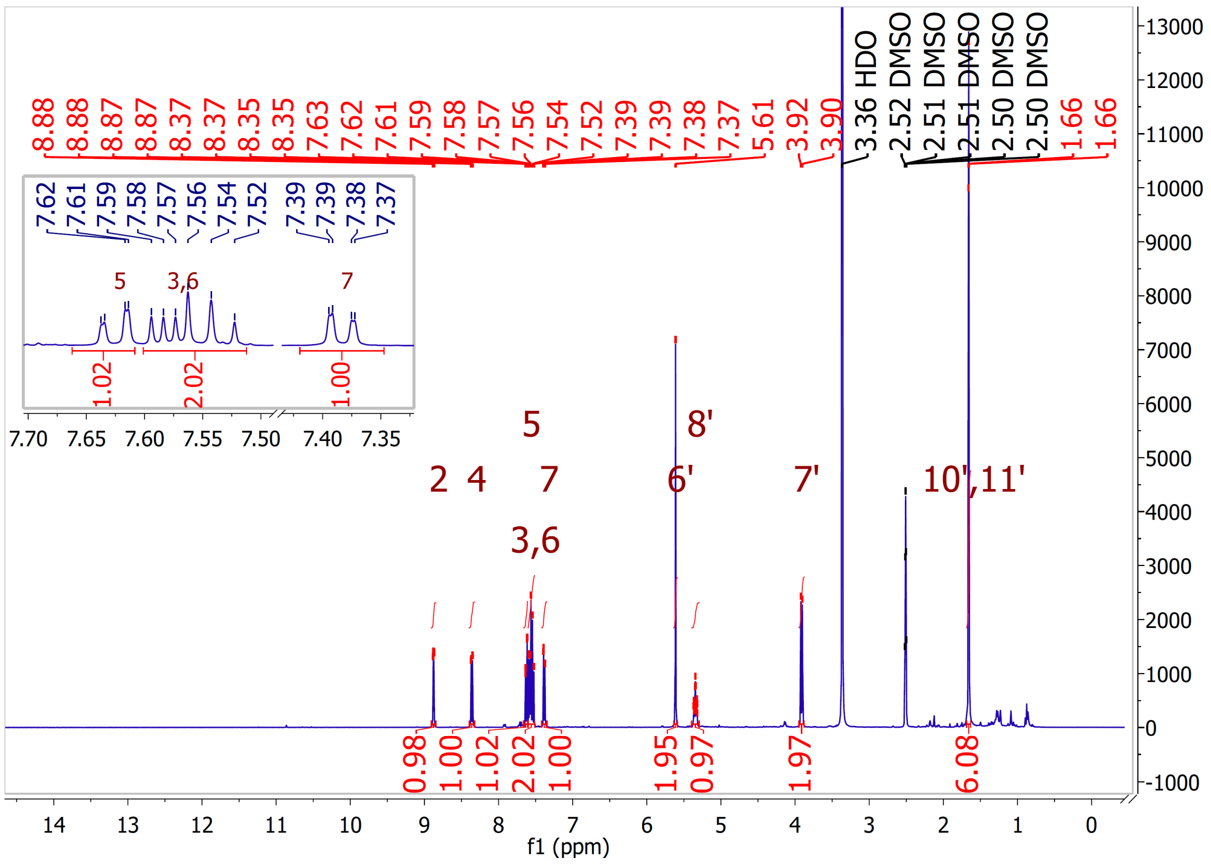

^{13}C NMR

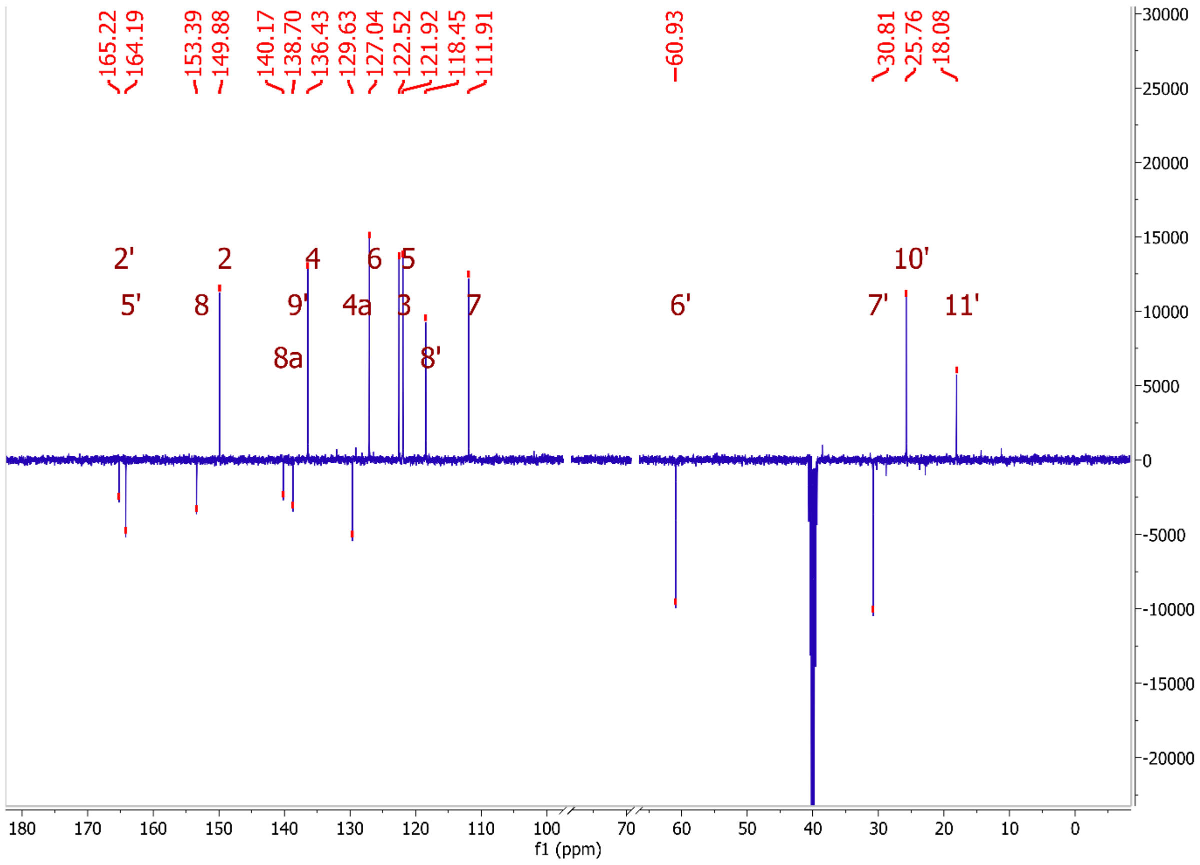

HRMS

Elemental Composition Report_Compound 4g

Single Mass Analysis
Tolerance = 5.0 PPM / DBE: min = -1.5, max = 500.0
Element prediction: Off
Number of isotope peaks used for i-FIT = 3

Monoisotopic Mass, Even Electron Ions
26 formula(e) evaluated with 1 results within limits (all results (up to 1000) for each mass) Elements Used:
C: 15-20 H: 15-20 N: 0-5 O: 0-5 Na: 1-1 S: 0-1

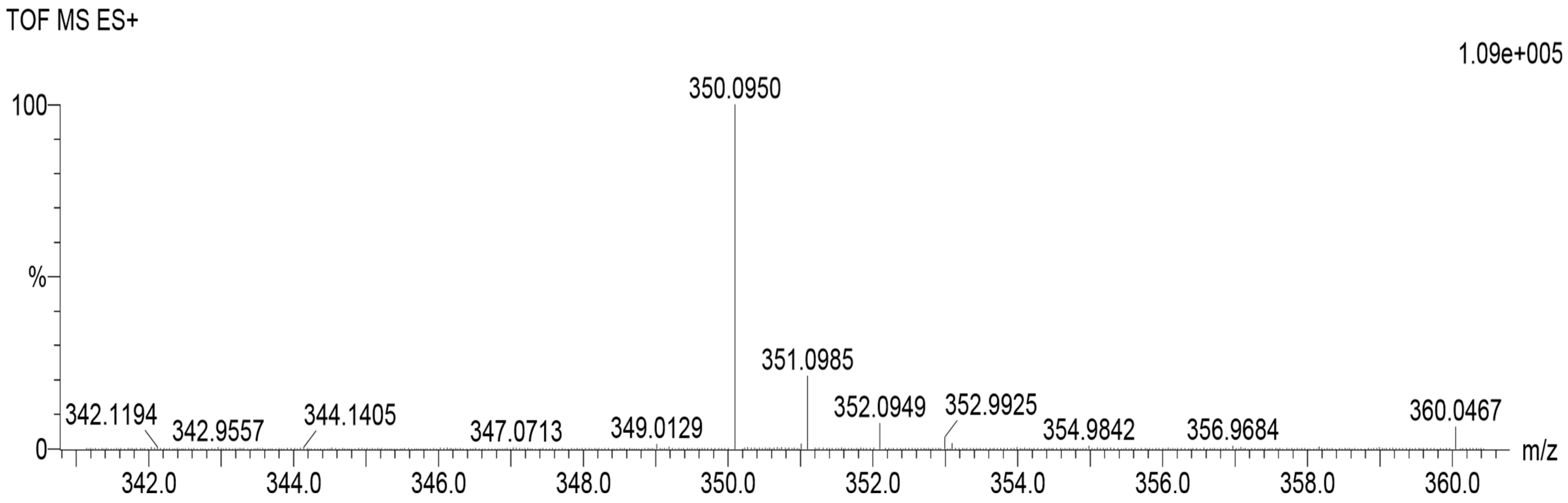

Minimum:				-1.5			
Maximum:		5.0	5.0	500.0			
Mass	Calc. Mass	mDa	PPM	DBE	i-FIT	i-FIT (Norm)	Formula
350.0950	350.0939	1.1	3.1	10.5	558.3	0.0	C17 H17 N3 O2 Na S

2-[(2-bromoethyl)thio]-5-[(quinolin-8-yloxy)methyl]-1,3,4-oxadiazole (**4h**)

^{1}H NMR

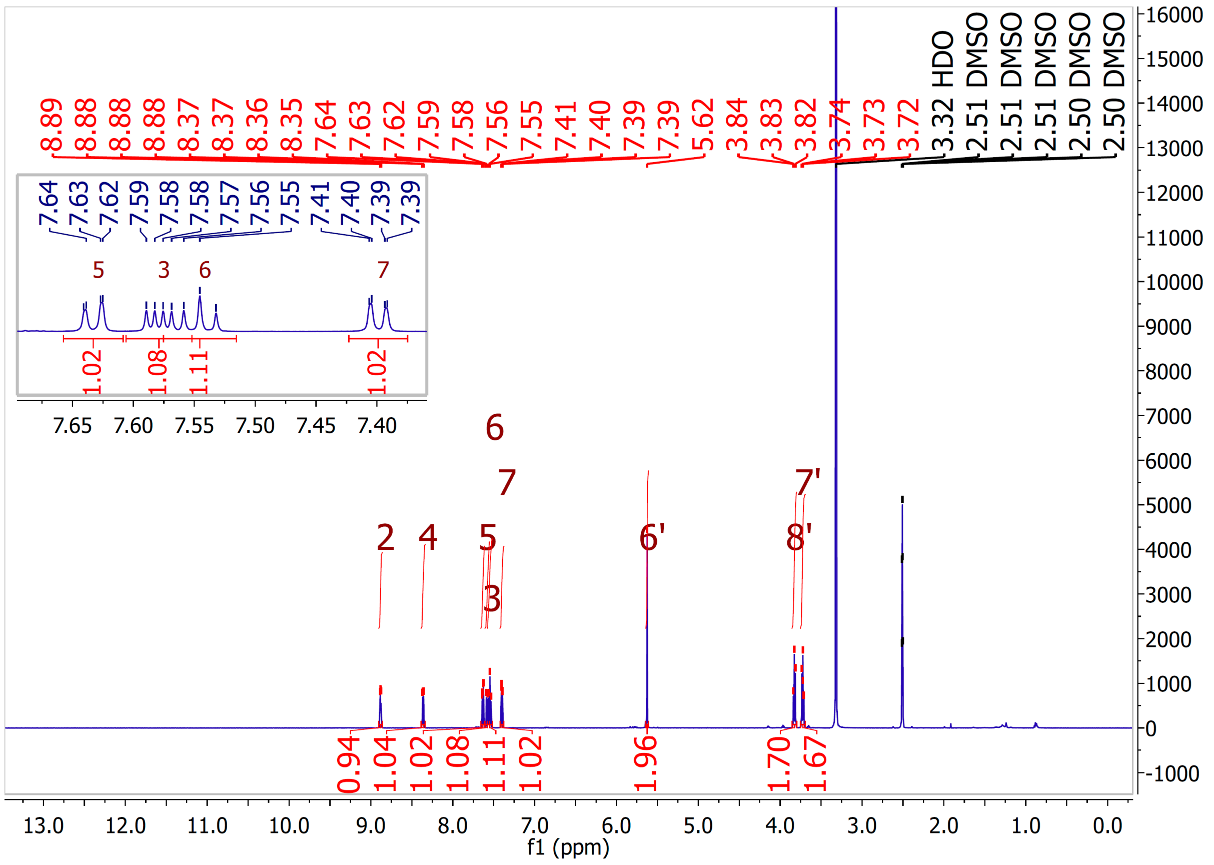

^{13}C NMR

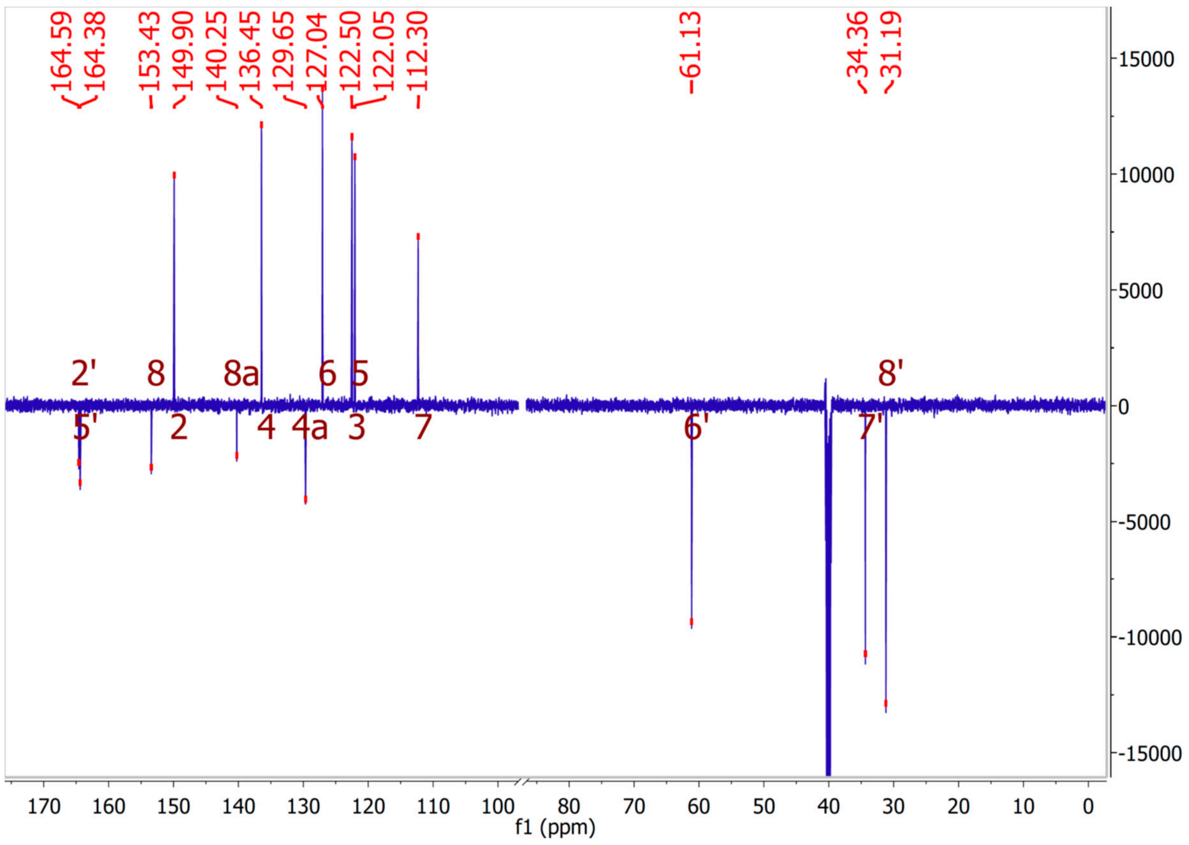

HRMS

Elemental Composition Report_Compound 4h

Single Mass Analysis
Tolerance = 5.0 PPM / DBE: min = -1.5, max = 500.0
Element prediction: Off
Number of isotope peaks used for i-FIT = 3

Monoisotopic Mass, Even Electron Ions
98 formula(e) evaluated with 1 results within limits (all results (up to 1000) for each mass) Elements Used:
C: 10-15 H: 10-15 N: 0-5 O: 0-5 Na: 1-1 S: 0-1 Br: 0-1

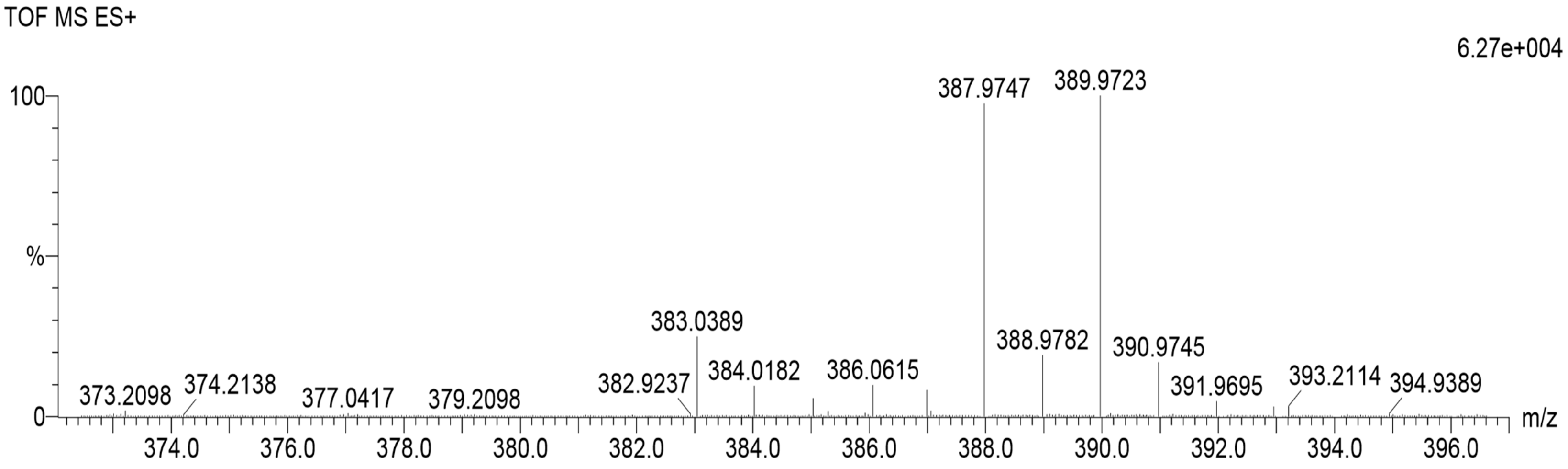

Minimum:				-1.5			
Maximum:		5.0	5.0	500.0			
Mass	Calc. Mass	mDa	PPM	DBE	i-FIT	i-FIT (Norm)	Formula
387.9747	387.9731	1.6	4.1	9.5	553.1	0.0	C14 H12 N3 O2 Na S Br

2-[(5-bromopentyl)thio]-5-[(quinolin-8-yloxy)methyl]-1,3,4-oxadiazole (**4i**)

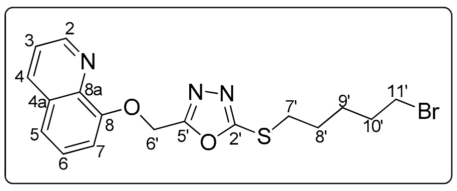

^{1}H NMR

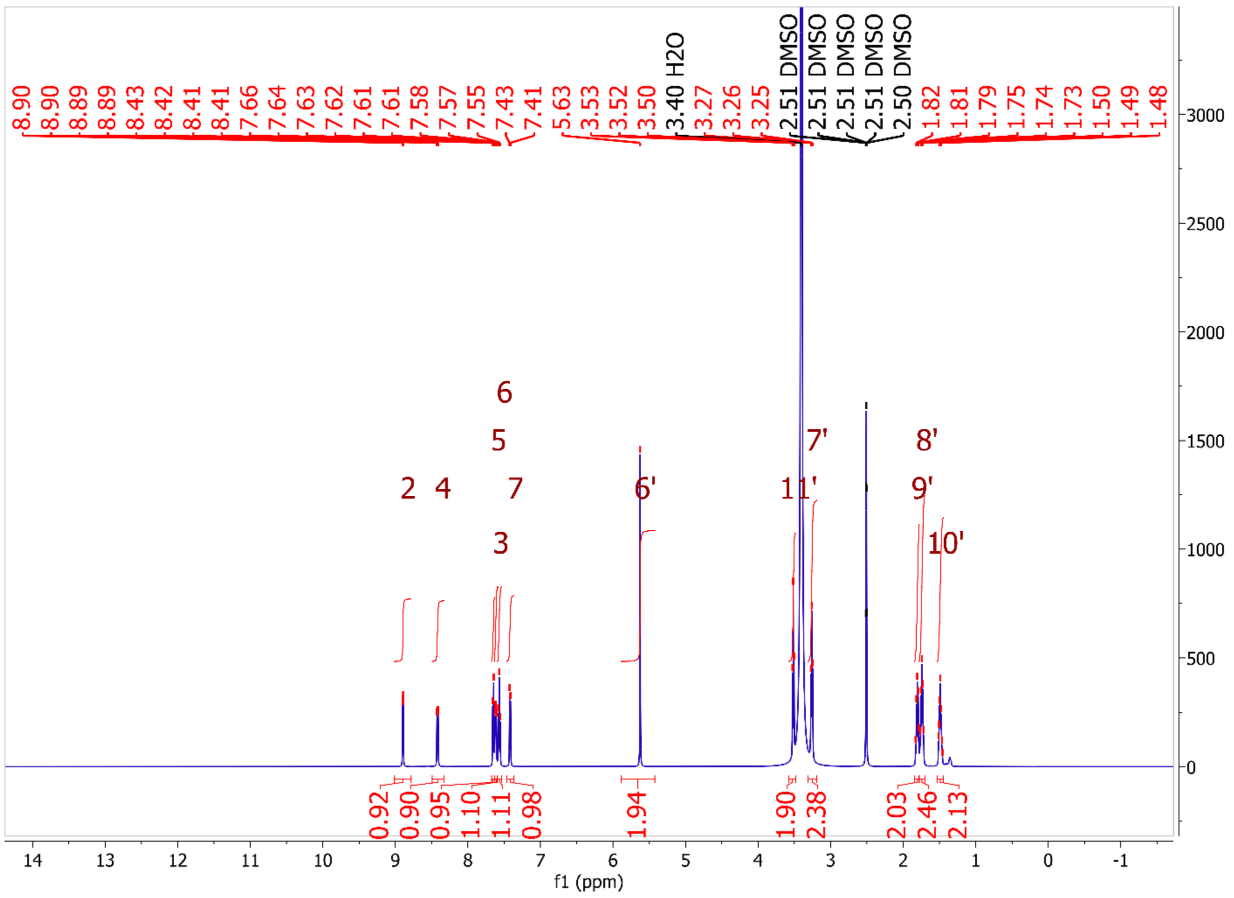

^{13}C NMR

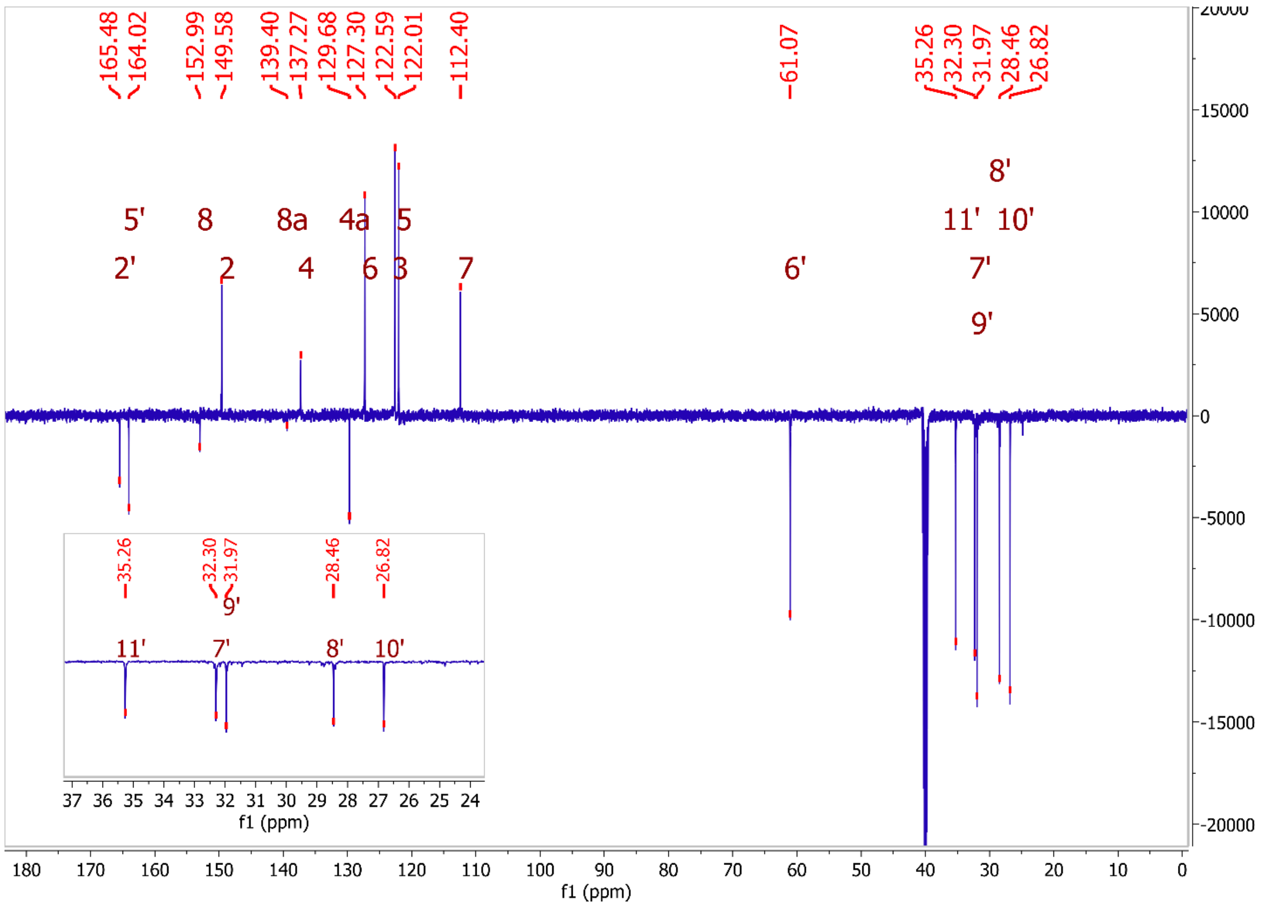

HRMS

Elemental Composition Report_Compound 4i

Single Mass Analysis
Tolerance = 5.0 PPM / DBE: min = -1.5, max = 500.0
Element prediction: Off
Number of isotope peaks used for i-FIT = 3

Monoisotopic Mass, Even Electron Ions
73 formula(e) evaluated with 1 results within limits (all results (up to 1000) for each mass) Elements Used:
C: 15-20 H: 15-20 N: 0-5 O: 0-5 Na: 1-1 S: 0-1 Br: 0-1

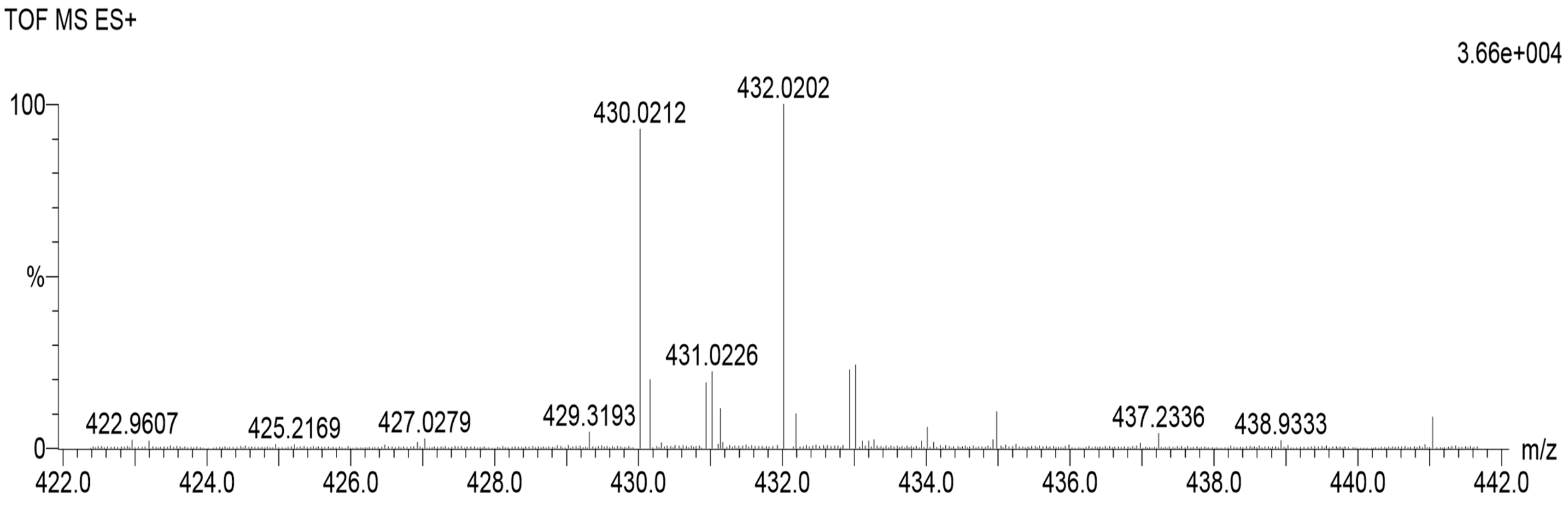

Minimum:				-1.5			
Maximum:		5.0	5.0	500.0			
Mass	Calc. Mass	mDa	PPM	DBE	i-FIT	i-FIT (Norm)	Formula
430.0212	430.0201	1.1	2.6	9.5	560.0	0.0	C17 H18 N3 O2 Na S Br

2-(benzylthio)-5-[(quinolin-8-yloxy)methyl]-1,3,4-oxadiazole (**4j**)

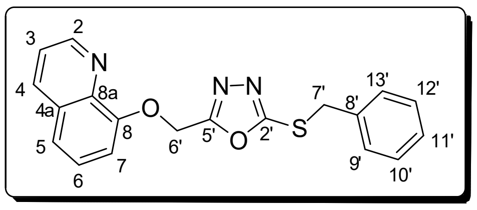

¹H NMR

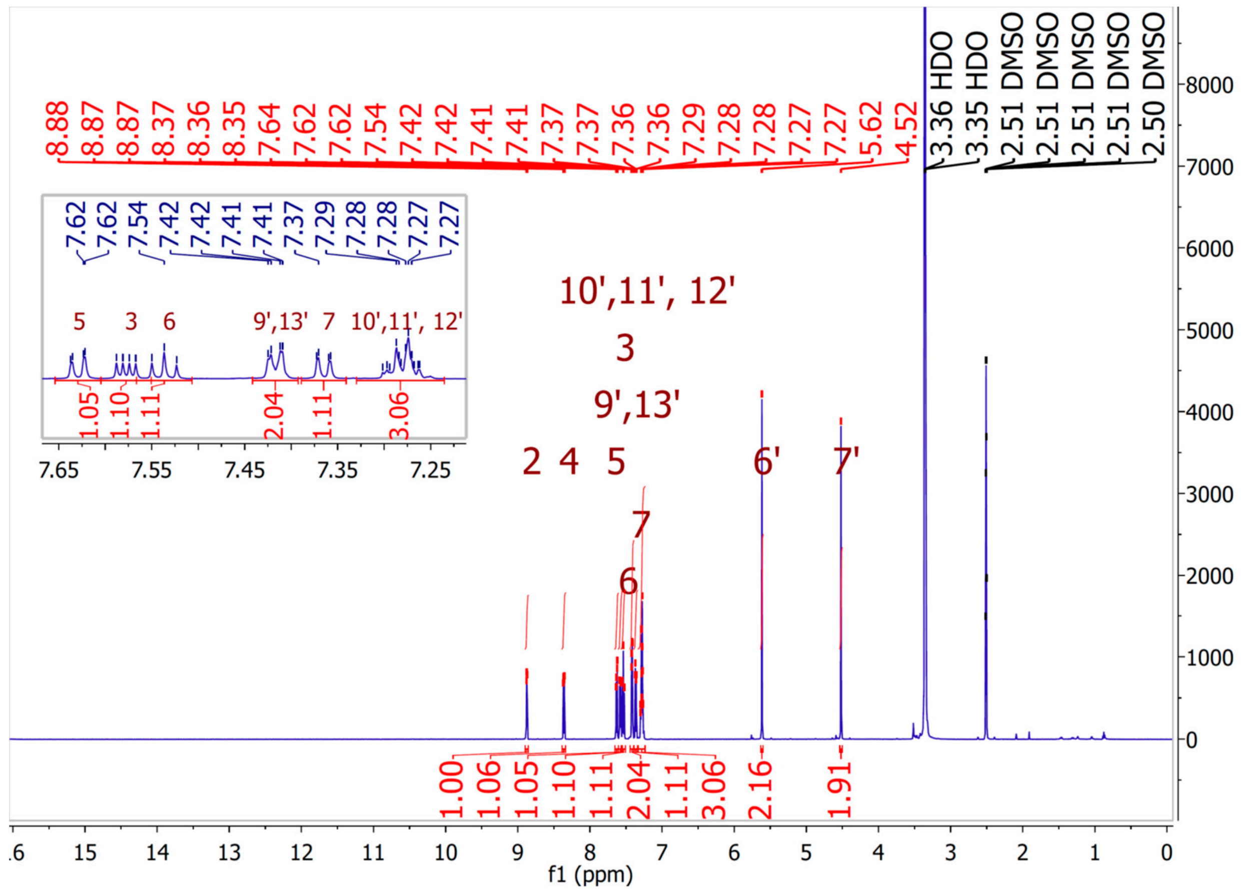

¹³C NMR

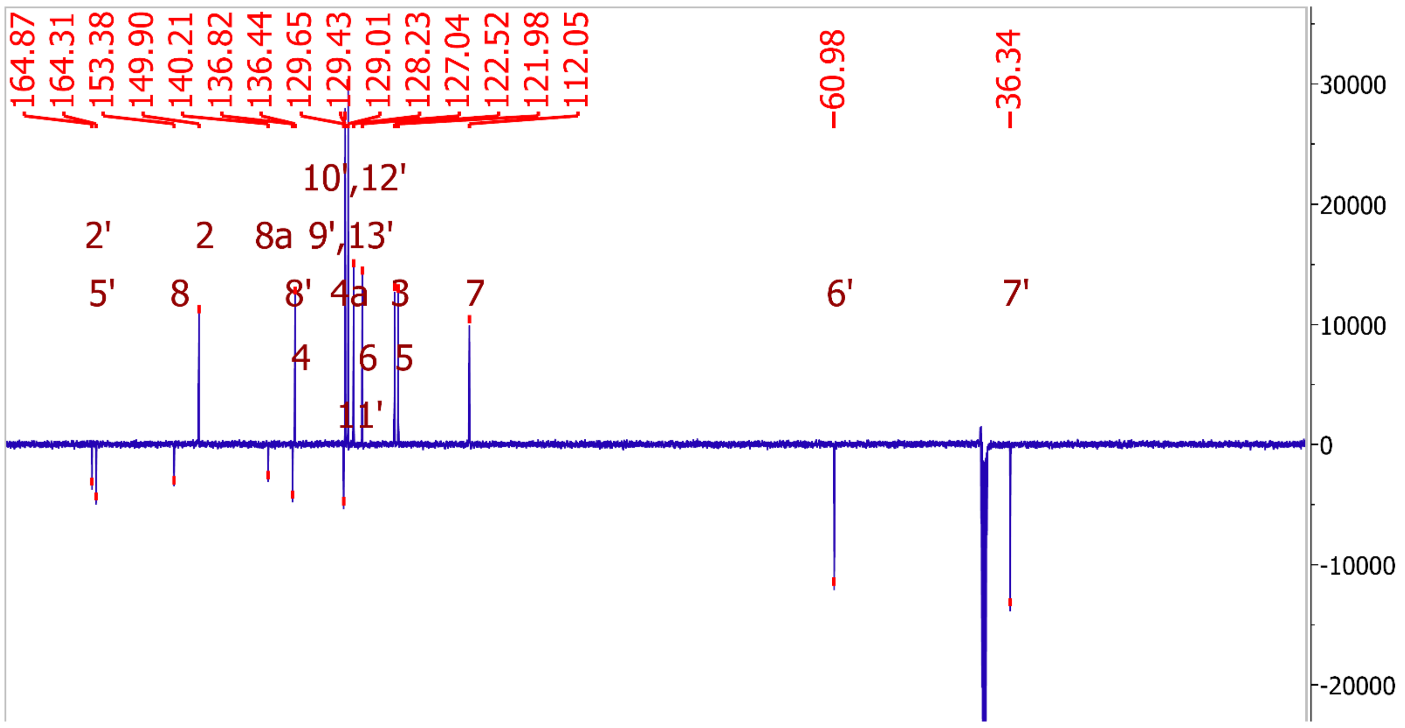

HMBC

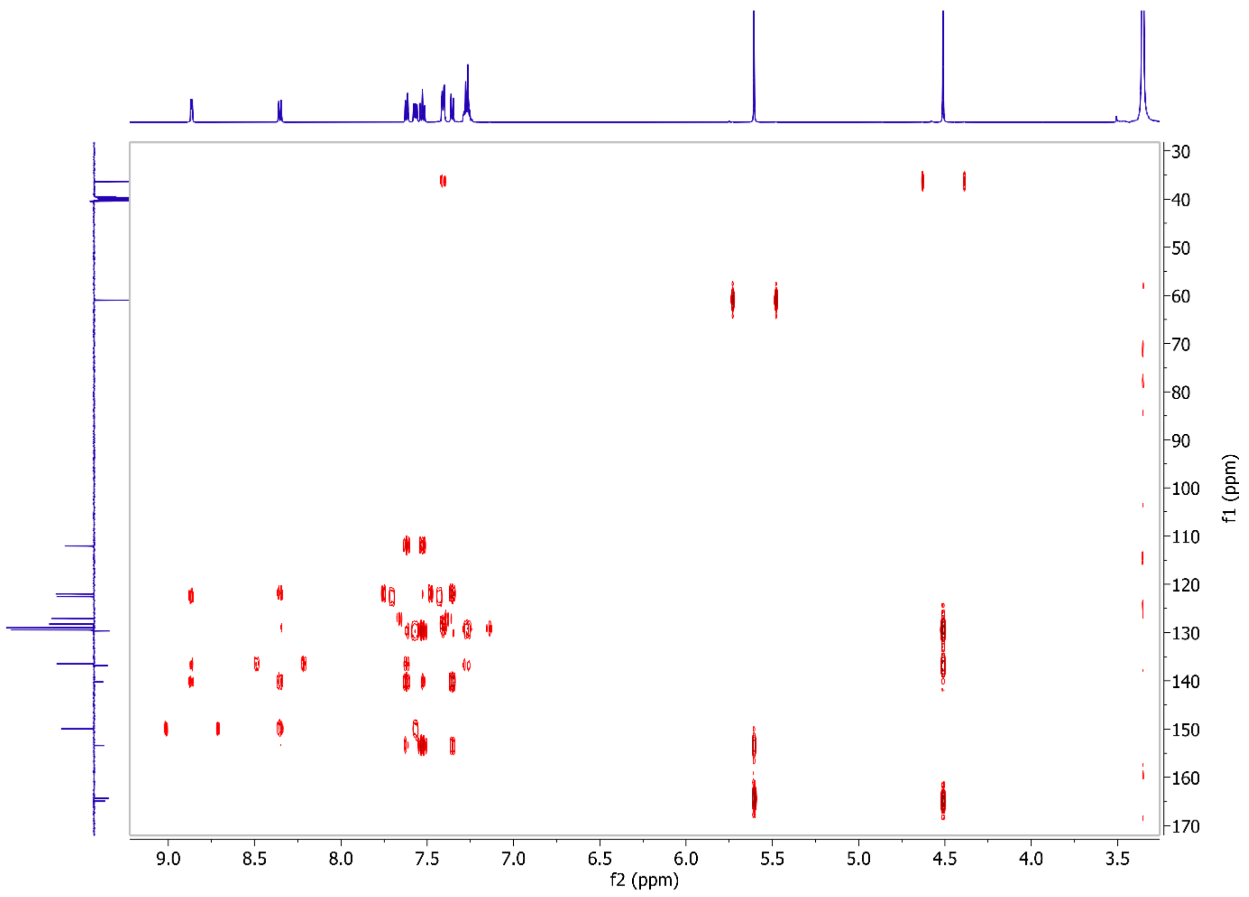

HSQC

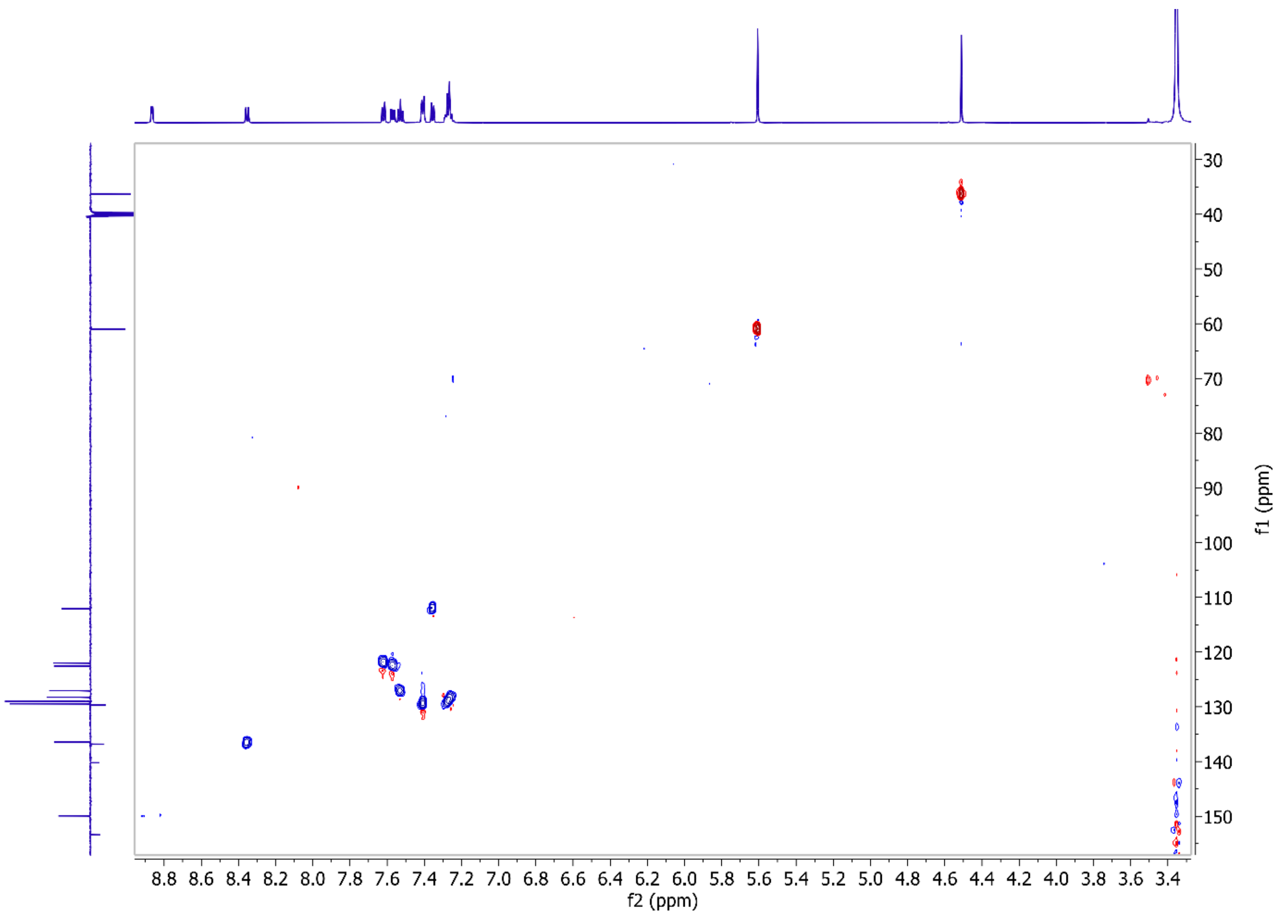

HRMS

Elemental Composition Report_Compound 4j

Single Mass Analysis
Tolerance = 5.0 PPM / DBE: min = -1.5, max = 500.0
Element prediction: Off
Number of isotope peaks used for i-FIT = 3

Monoisotopic Mass, Even Electron Ions
33 formula(e) evaluated with 1 results within limits (all results (up to 1000) for each mass) Elements Used:
C: 15-20 H: 15-20 N: 0-5 O: 0-5 Na: 1-1 S: 0-1

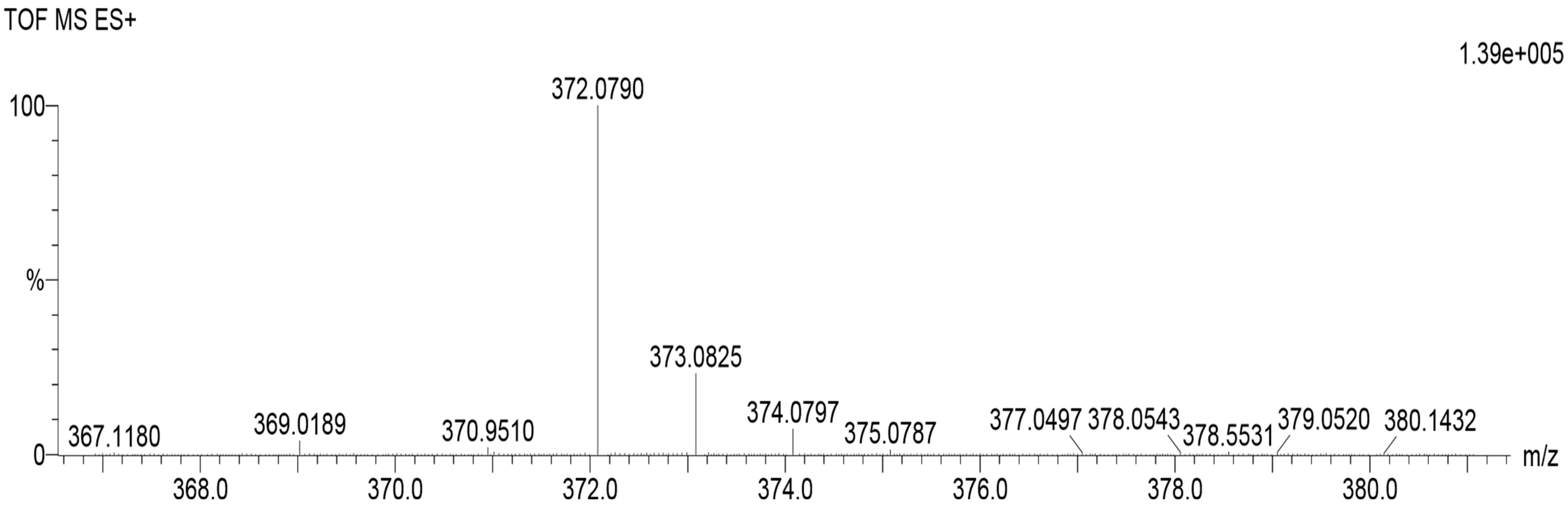

Minimum:				-1.5			
Maximum:		5.0	5.0	500.0			
Mass	Calc. Mass	mDa	PPM	DBE	i-FIT	i-FIT (Norm)	Formula
372.0790	372.0783	0.7	1.9	13.5	585.5	0.0	C19 H15 N3 O2 Na

2-([4-fluorobenzyl)thio]-5-[(quinolin-8-yloxy)methyl]-1,3,4-oxadiazole (**4k**)

^{1}H NMR

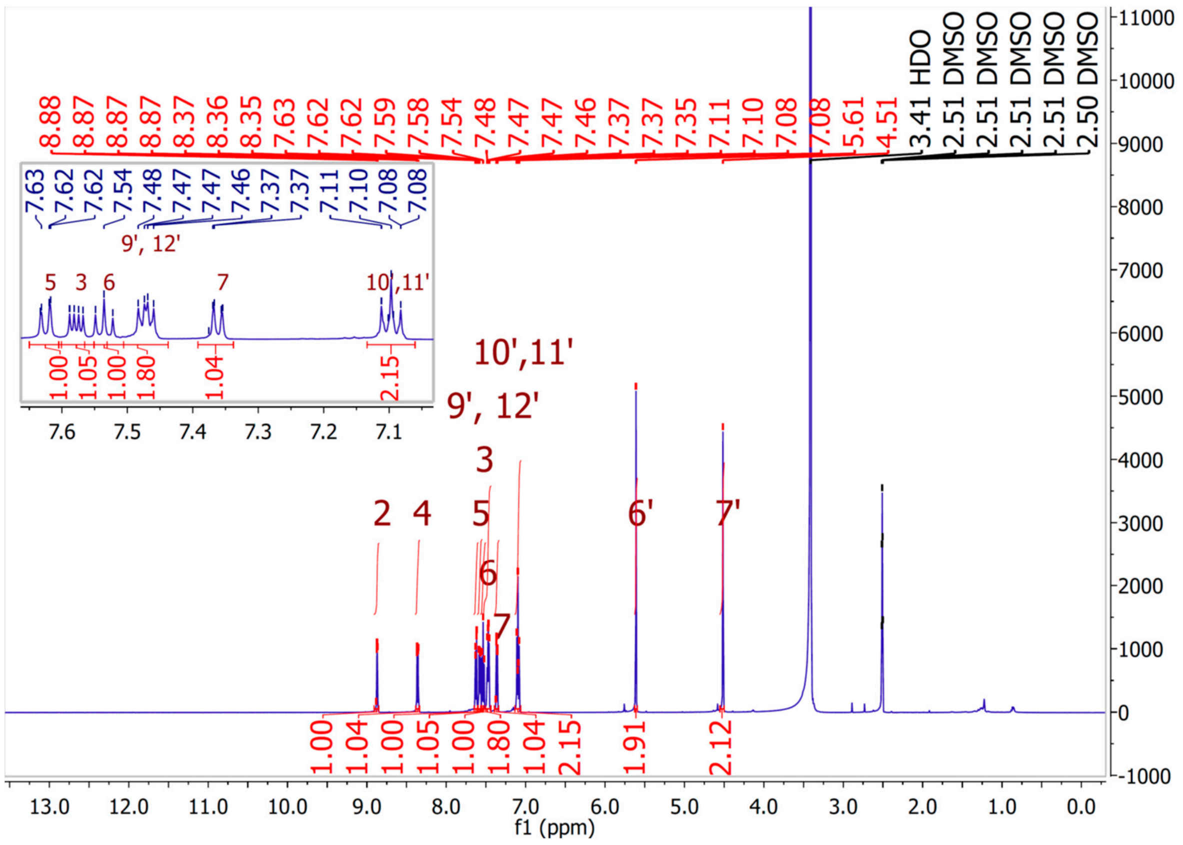

^{13}C NMR

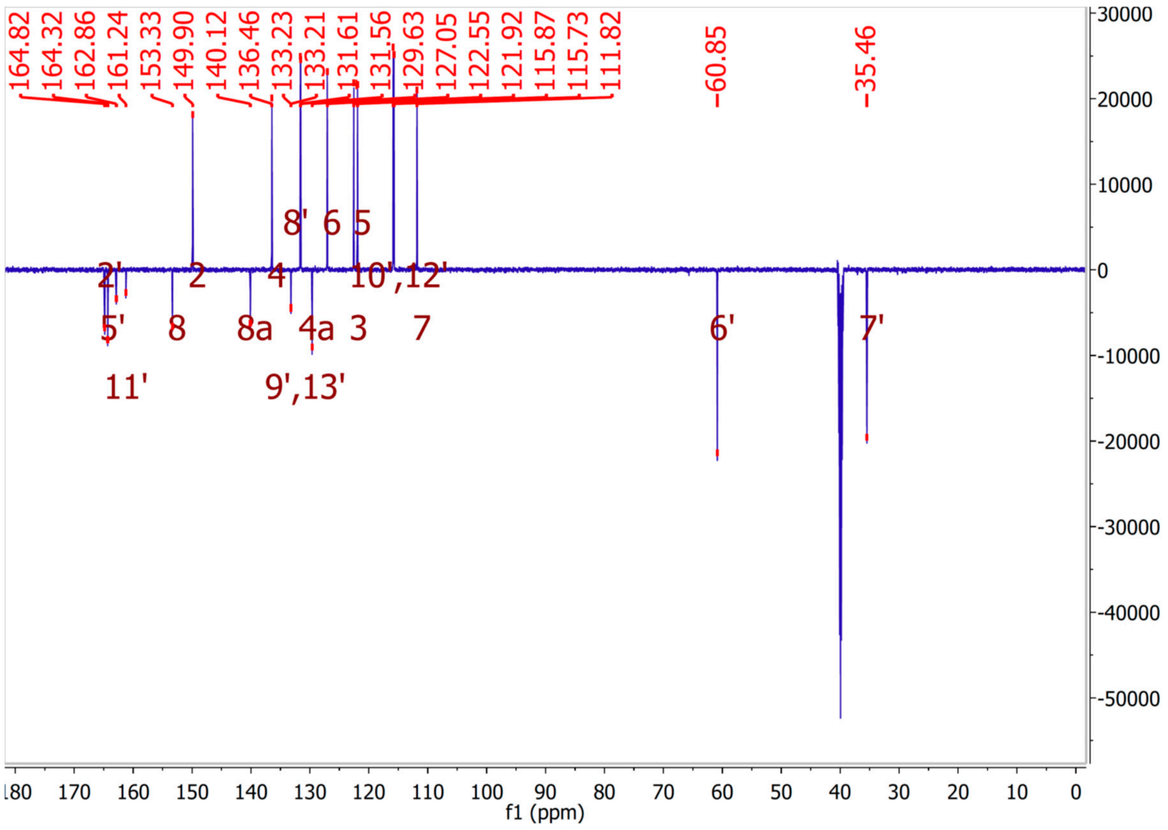

HSQC

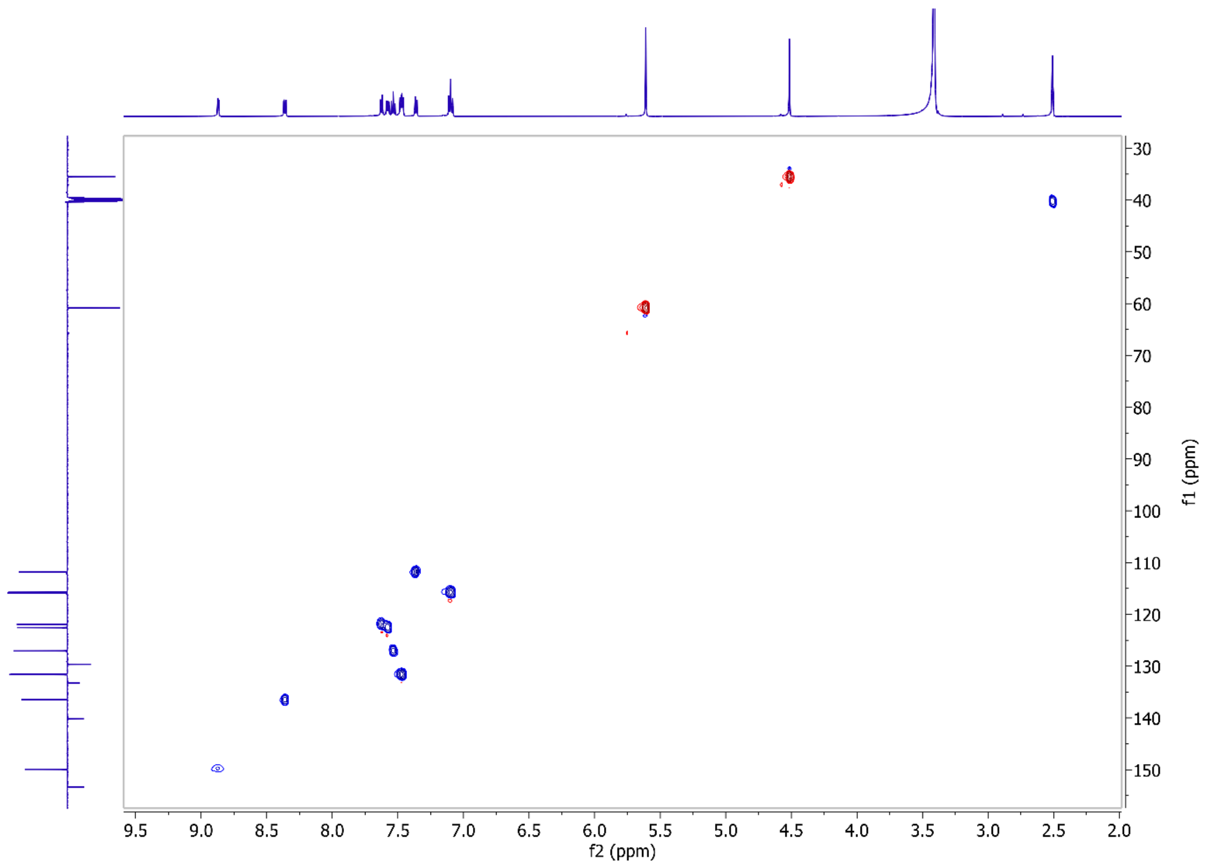

HMBC

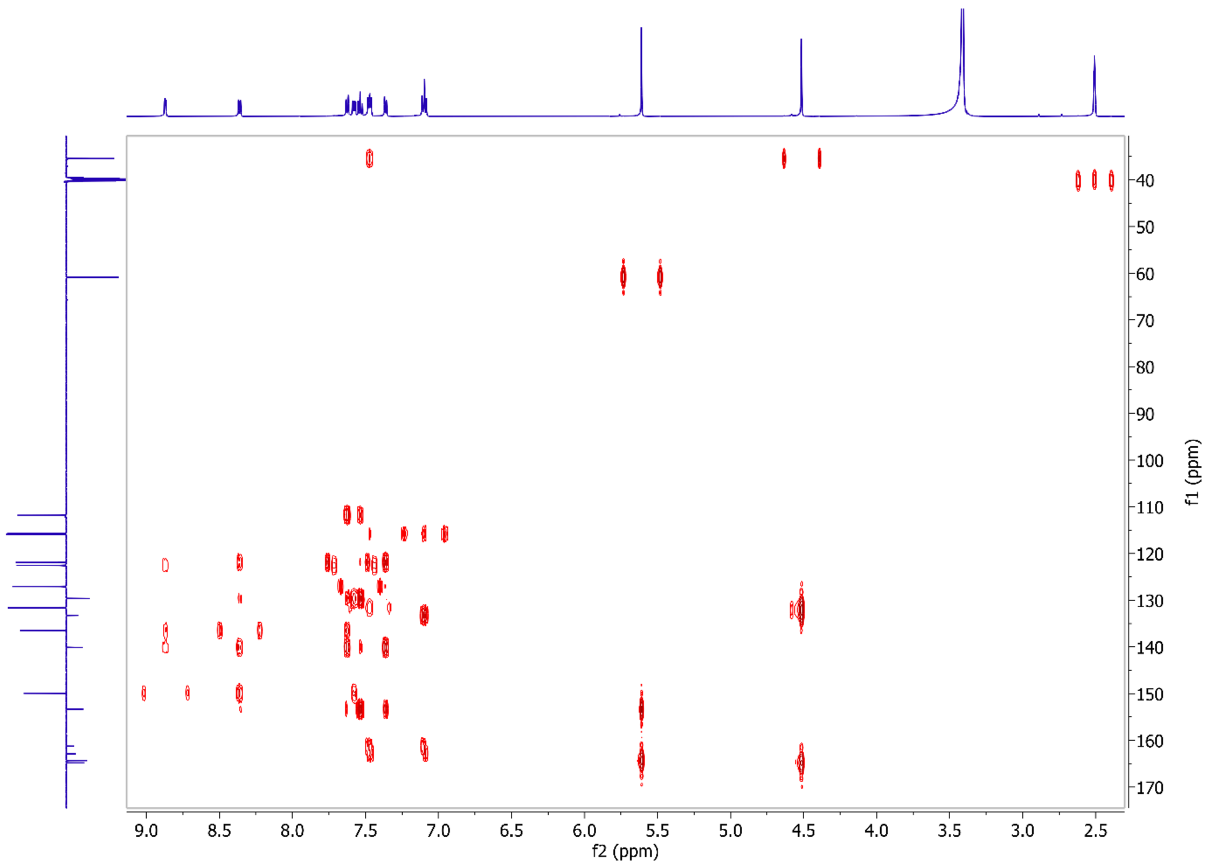

HRMS

Elemental Composition Report_Compound 4k

Single Mass Analysis

Tolerance = 5.0 PPM / DBE: min = -1.5, max = 500.0
Element prediction: Off
Number of isotope peaks used for i-FIT = 3

Monoisotopic Mass, Even Electron Ions
62 formula(e) evaluated with 1 results within limits (all results (up to 1000) for each mass) Elements Used:
C: 15-20 H: 10-15 N: 0-5 O: 0-5 Na: 1-1 S: 0-1 F: 0-1

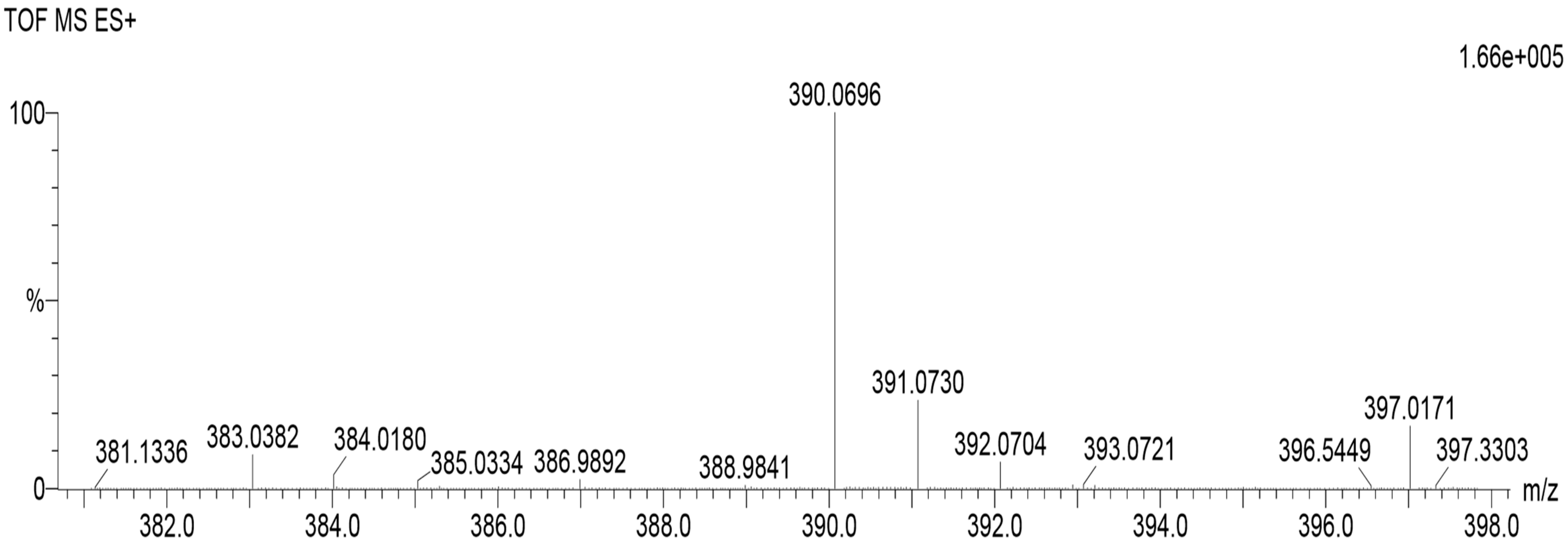

Minimum:				-1.5			
Maximum:		5.0	5.0	500.0			
Mass	Calc. Mass	mDa	PPM	DBE	i-FIT	i-FIT (Norm)	Formula
390.0696	390.0688	0.8	2.1	13.5	582.5	0.0	C19 H14 N3 O2 Na S F

2-[(4-chlorobenzyl)thio]-5-[(quinolin-8-yloxy)methyl]-1,3,4-oxadiazole (**4l**)

^{1}H NMR

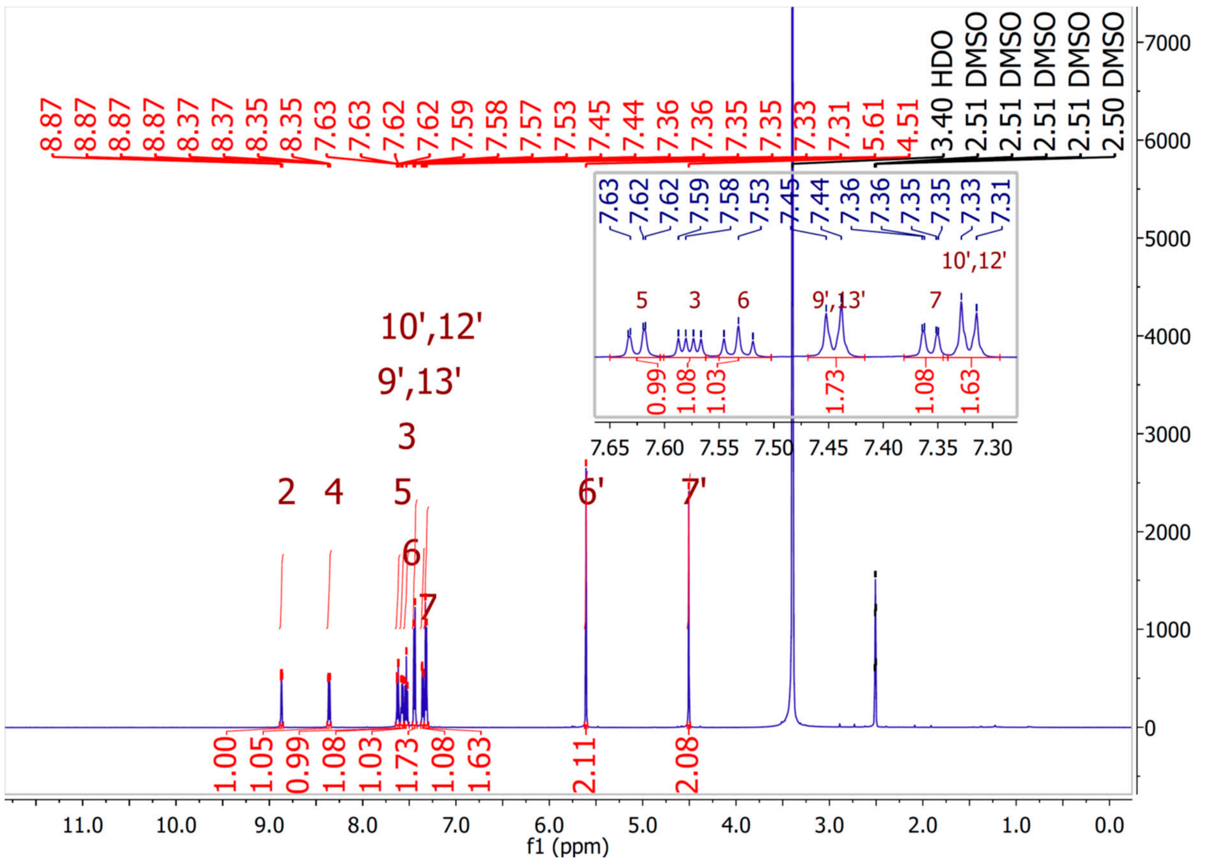

^{13}C NMR

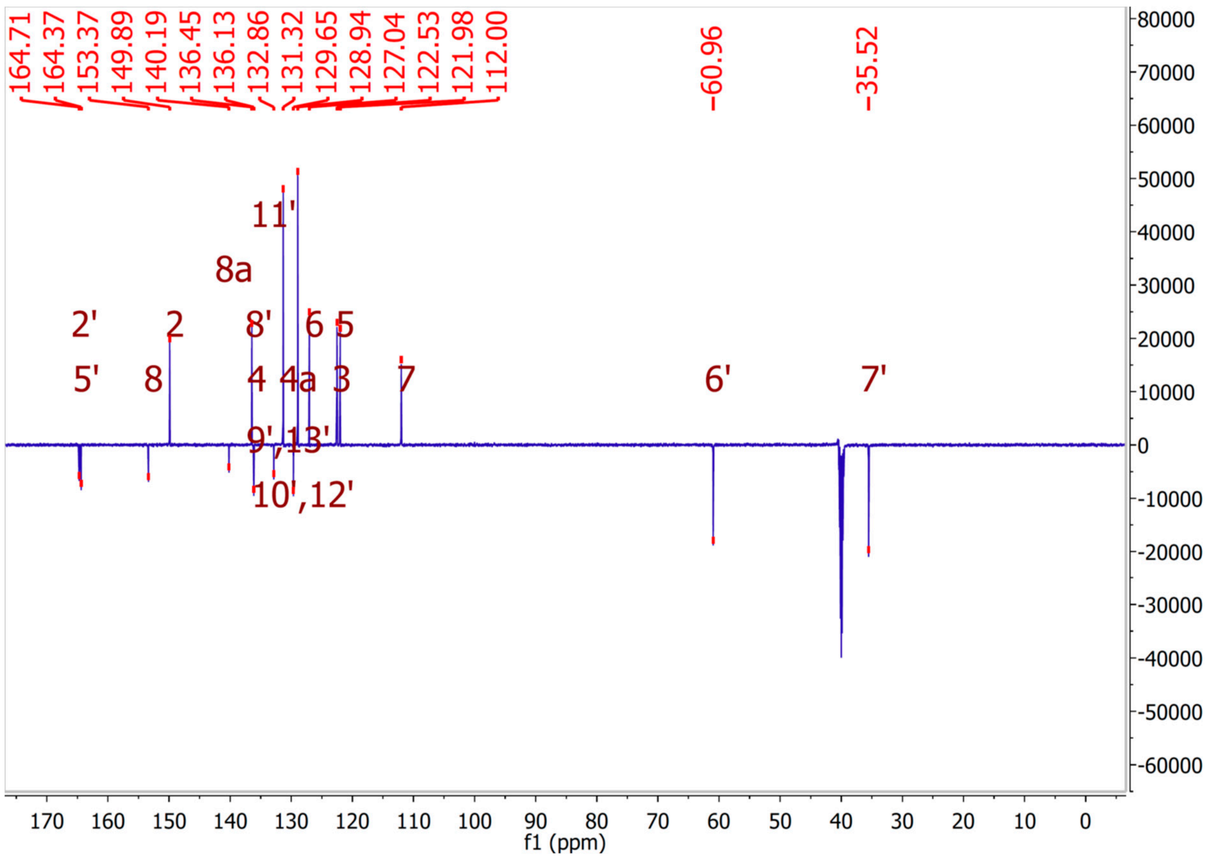

HSQC

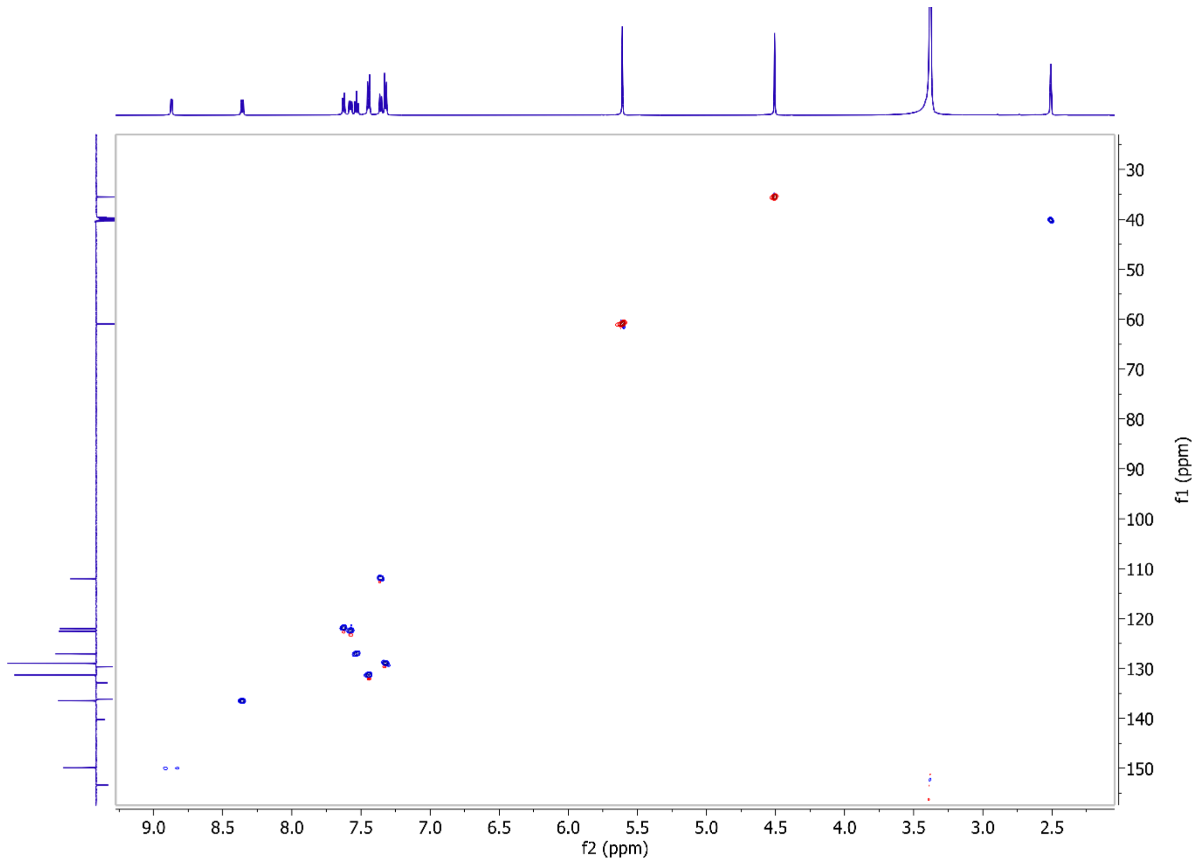

HMBC

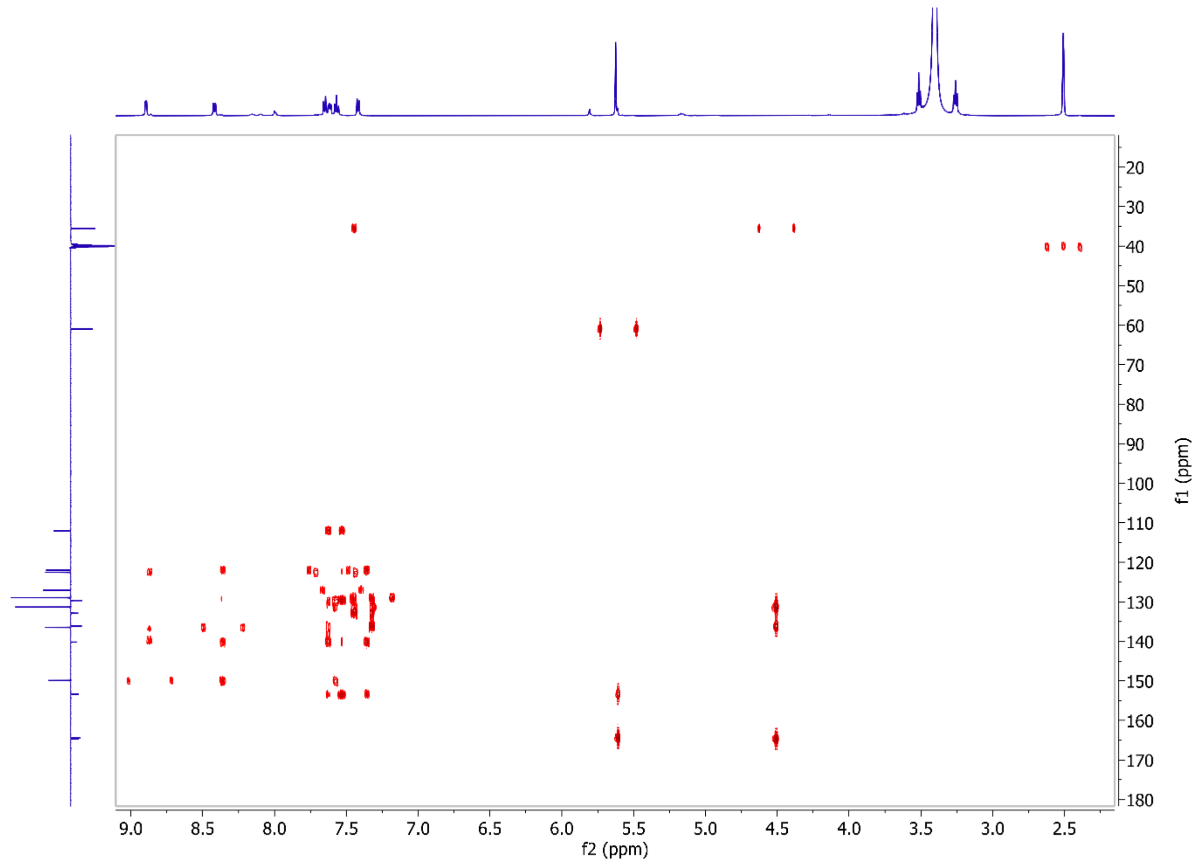

HRMS

Elemental Composition Report_Compound 4l

Single Mass Analysis

Tolerance = 5.0 PPM / DBE: min = -1.5, max = 500.0
Element prediction: Off
Number of isotope peaks used for i-FIT = 3

Monoisotopic Mass, Even Electron Ions
67 formula(e) evaluated with 1 results within limits (all results (up to 1000) for each mass) Elements Used:
C: 15-20 H: 10-15 N: 0-5 O: 0-5 Na: 1-1 S: 0-1 Cl: 0-1

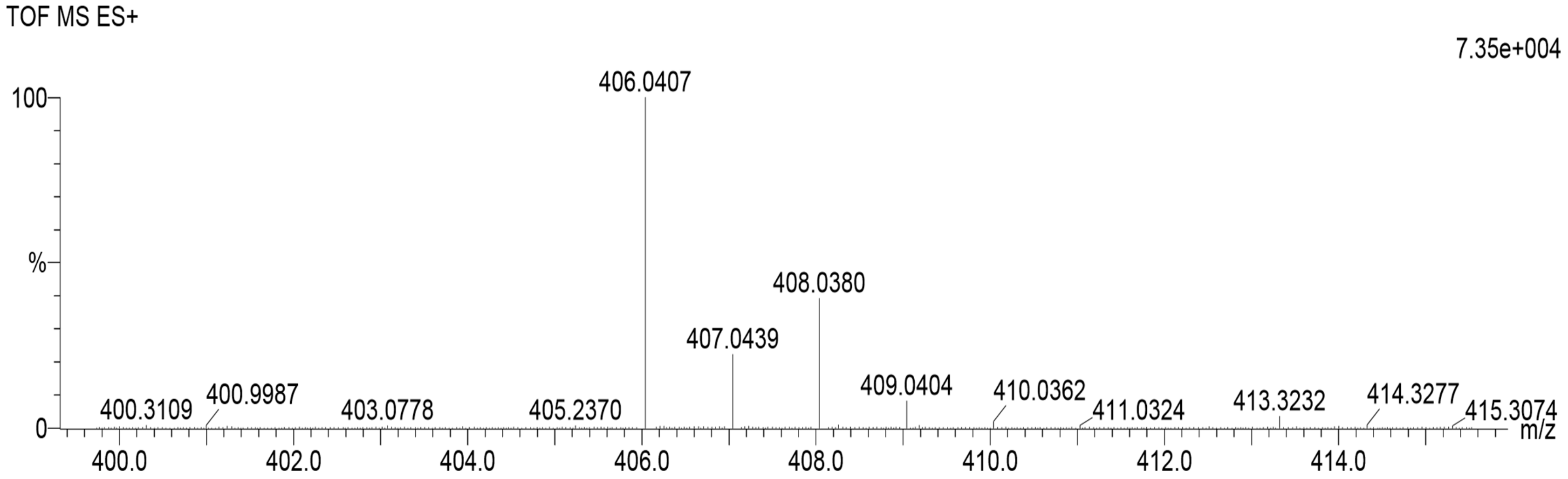

	Mass	Calc. Mass	mDa	PPM	DBE	i-FIT	i-FIT (Norm)	Formula
Minimum:					-1.5			
Maximum:			5.0	5.0	500.0			
	406.0407	406.0393	1.4	3.4	13.5	525.4	0.0	C19 H14 N3 O2 Na S Cl

2-[(4-bromobenzyl)thio)]-5-[(quinolin-8-yloxy)methyl]-1,3,4-oxadiazole (**4m**)

^{1}H NMR

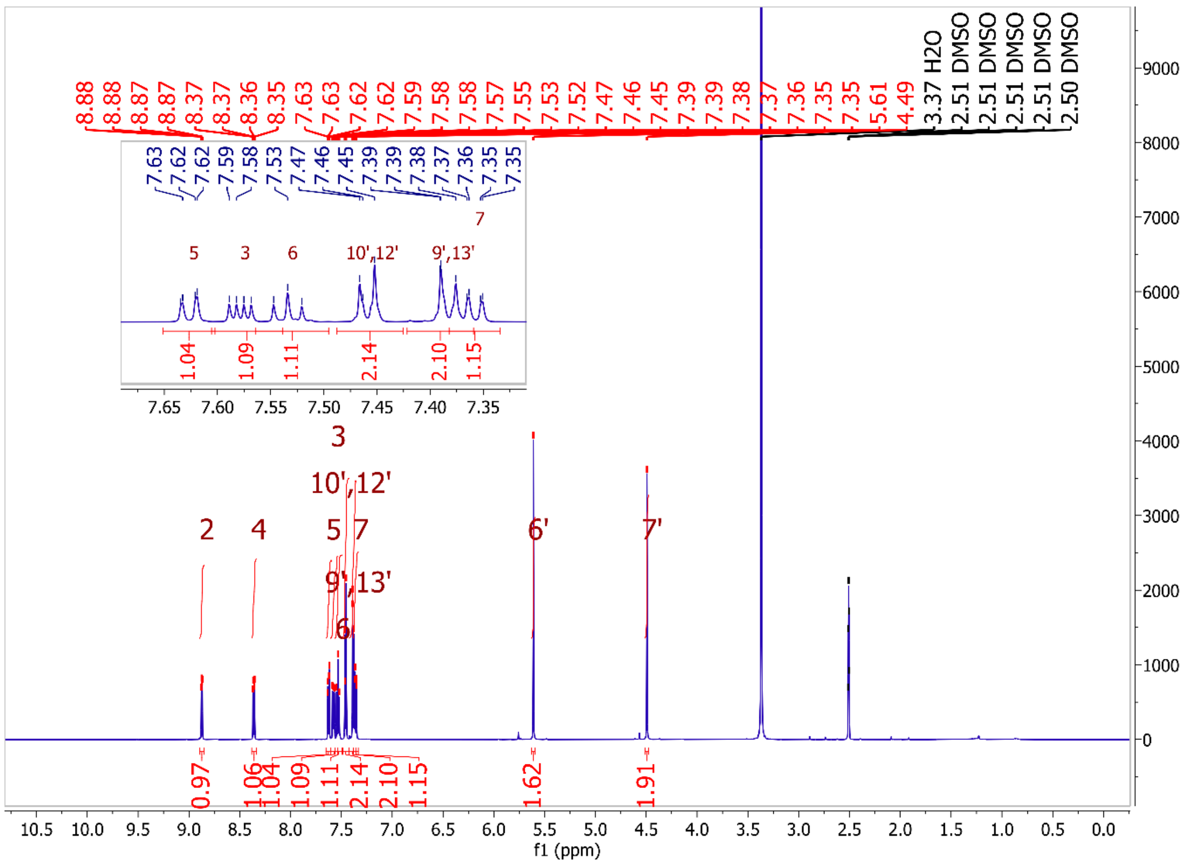

^{13}C NMR

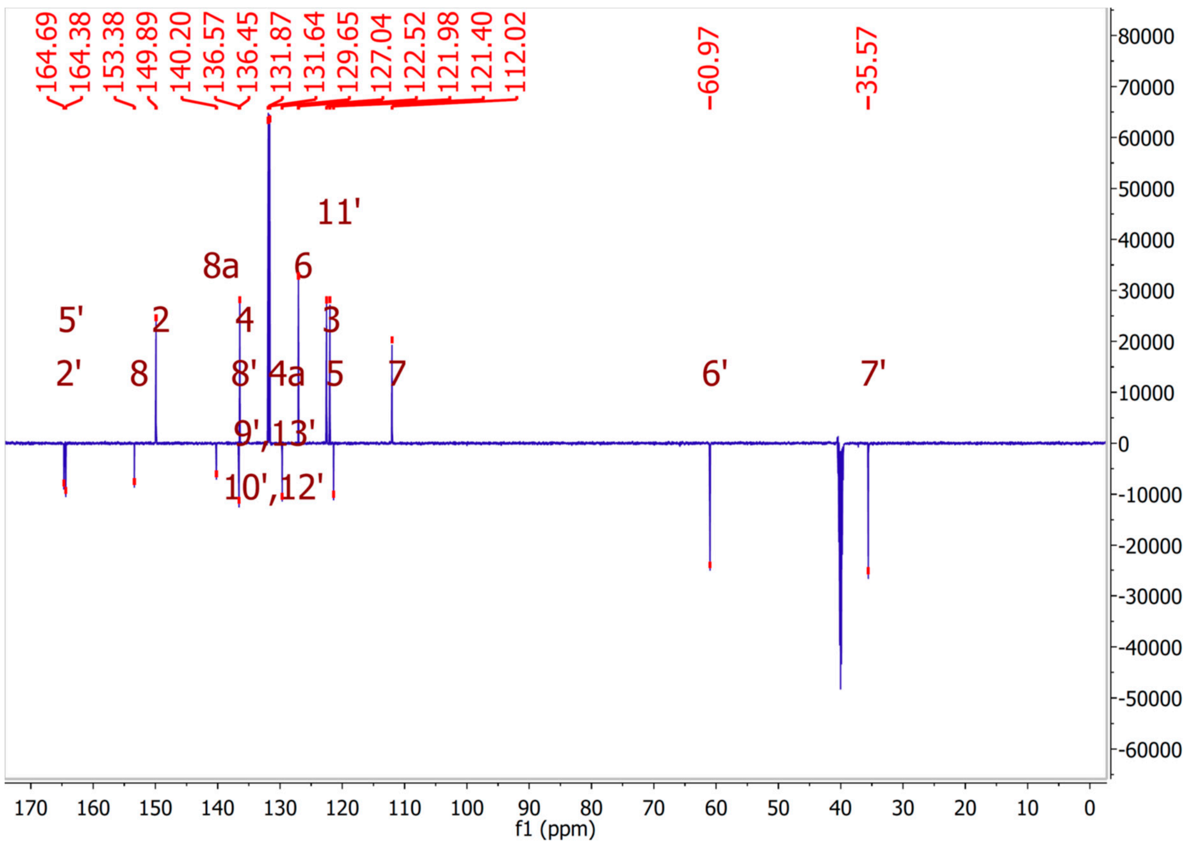

HRMS

Elemental Composition Report_Compound 4m

Single Mass Analysis

Tolerance = 5.0 PPM / DBE: min = -1.5, max = 500.0
Element prediction: Off
Number of isotope peaks used for i-FIT = 3

Monoisotopic Mass, Even Electron Ions
90 formula(e) evaluated with 1 results within limits (all results (up to 1000) for each mass) Elements Used:
C: 15-20 H: 10-15 N: 0-5 O: 0-5 Na: 1-1 S: 0-1 Br: 0-1

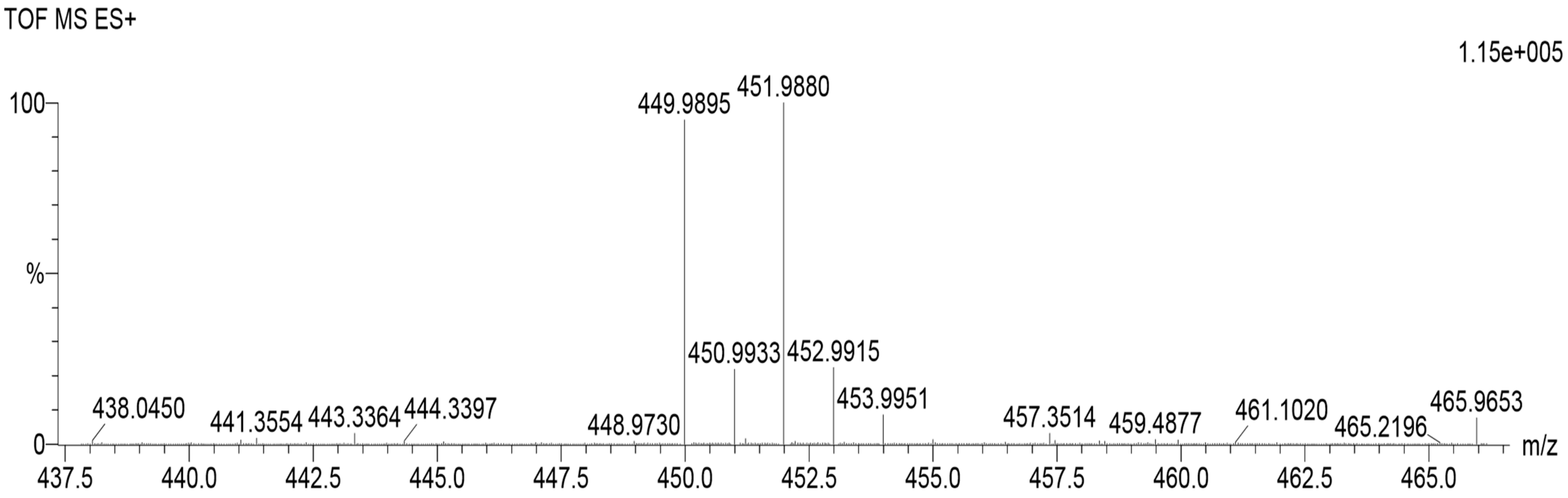

Minimum:				-1.5			
Maximum:		5.0	5.0	500.0			
Mass	Calc. Mass	mDa	PPM	DBE	i-FIT	i-FIT (Norm)	Formula
449.9895	449.9888	0.7	1.6	13.5	541.5	0.0	C19 H14 N3 O2 Na S Br

2-[(4-nitrobenzyl)thio]-5-[(quinolin-8-yloxy)methyl]-1,3,4-oxadiazole (**4n**)

^{1}H NMR

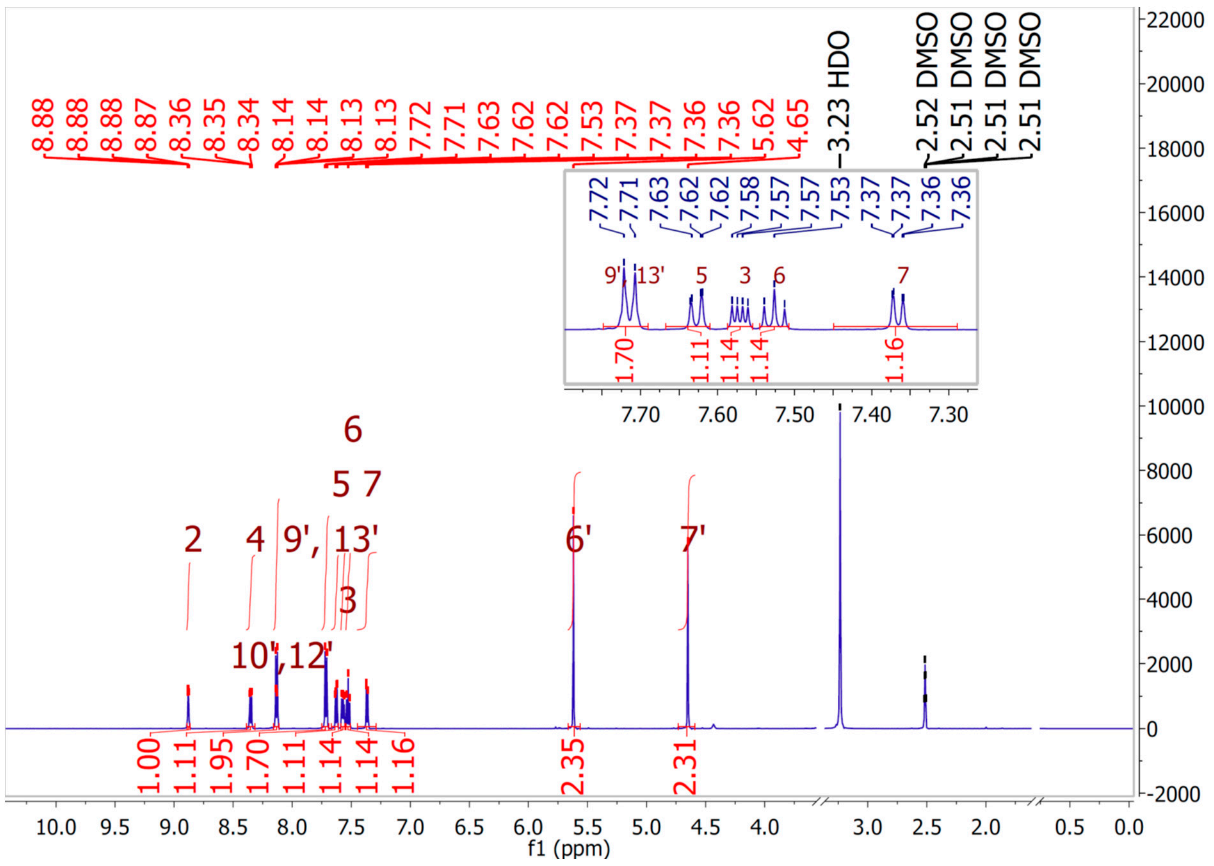

^{13}C NMR

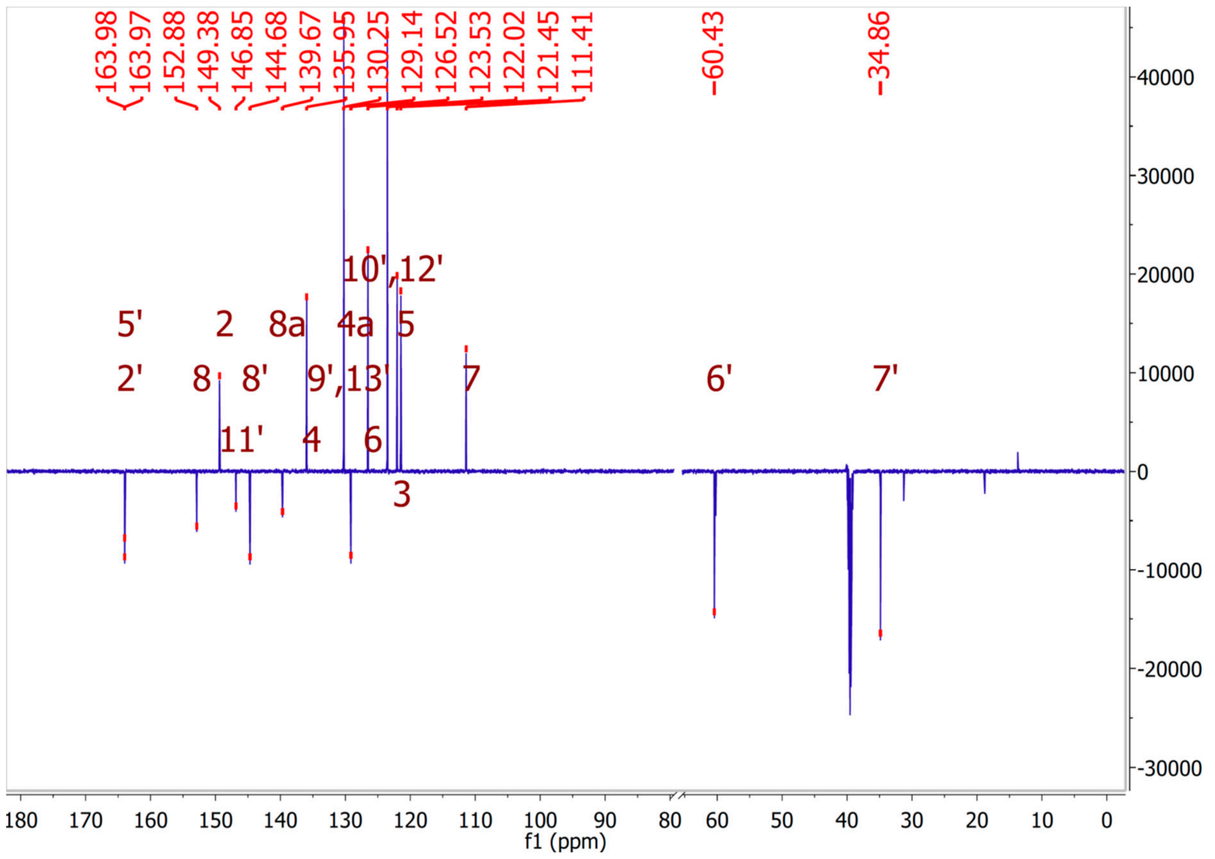

HRMS

Elemental Composition Report_Compound 4n

Single Mass Analysis

Tolerance = 5.0 PPM / DBE: min = -1.5, max = 500.0
Element prediction: Off
Number of isotope peaks used for i-FIT = 3

Monoisotopic Mass, Even Electron Ions
46 formula(e) evaluated with 1 results within limits (all results (up to 1000) for each mass) Elements Used:
C: 15-20 H: 10-15 N: 0-5 O: 0-5 Na: 1-1 S: 0-1

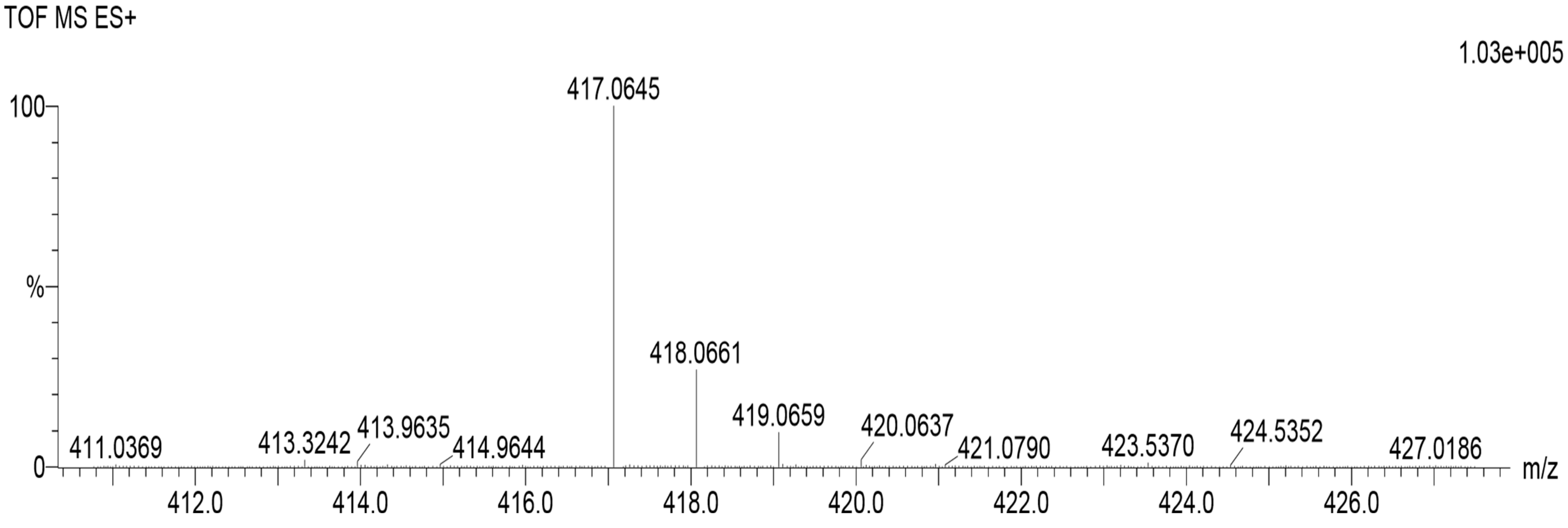

Minimum:				-1.5			
Maximum:		5.0	5.0	500.0			
Mass	Calc. Mass	mDa	PPM	DBE	i-FIT	i-FIT (Norm)	Formula
417.0645	417.0633	1.2	2.9	14.5	557.5	0.0	C19 H14 N4 O4 Na S

2-[(3-fluorobenzyl)thio]-5-[(quinolin-8-yloxy)methyl]-1,3,4-oxadiazole (**4o**)

^{1}H NMR

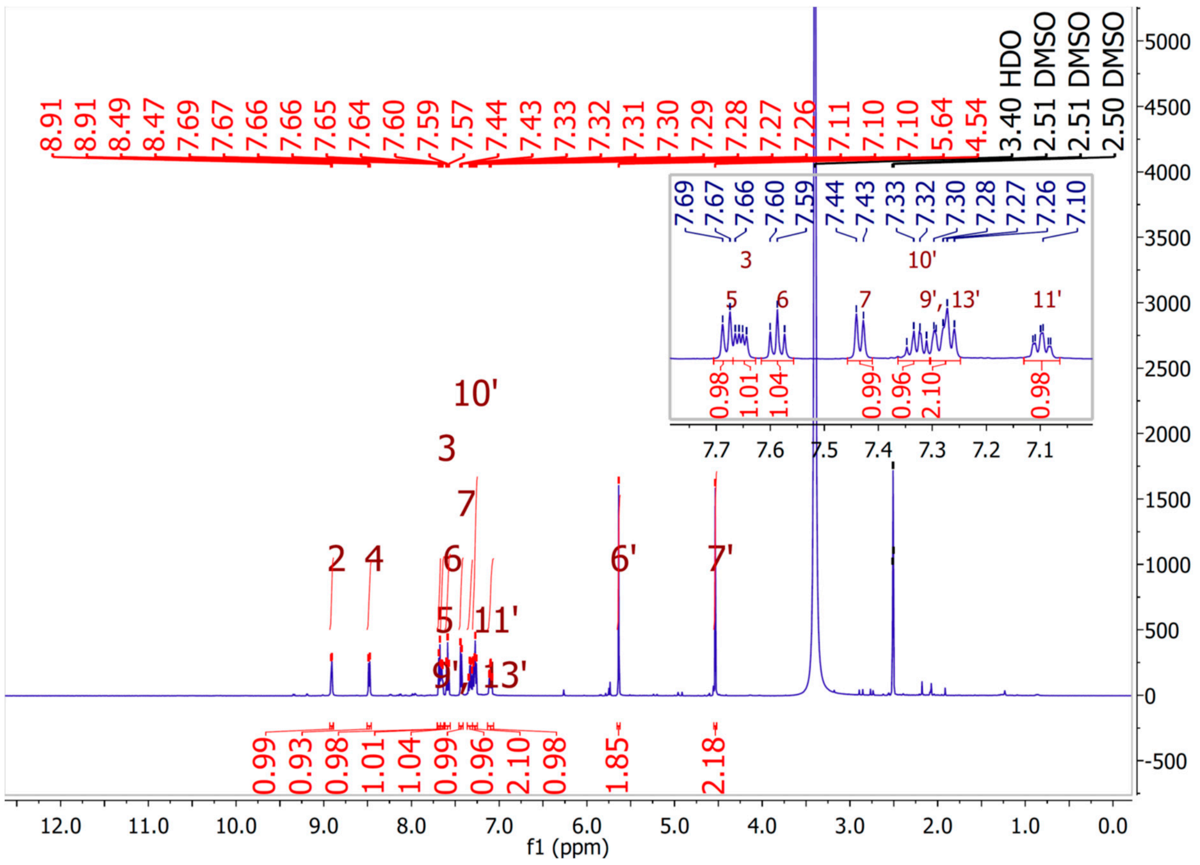

^{13}C NMR

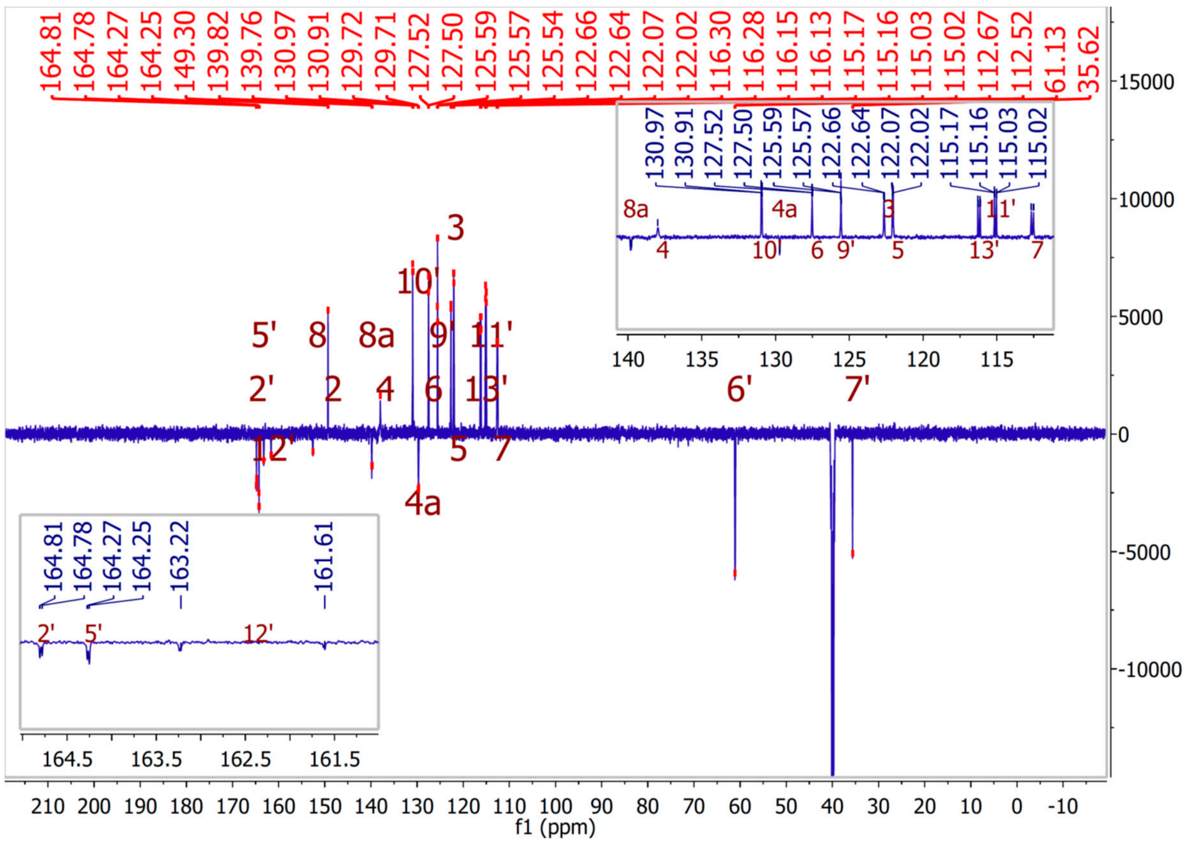

HRMS

Elemental Composition Report_Compound 4o

Single Mass Analysis
Tolerance = 5.0 PPM / DBE: min = -1.5, max = 500.0
Element prediction: Off
Number of isotope peaks used for i-FIT = 3

Monoisotopic Mass, Even Electron Ions
62 formula(e) evaluated with 1 results within limits (all results (up to 1000) for each mass) Elements Used:
C: 15-20 H: 10-15 N: 0-5 O: 0-5 Na: 1-1 S: 0-1 F: 0-1

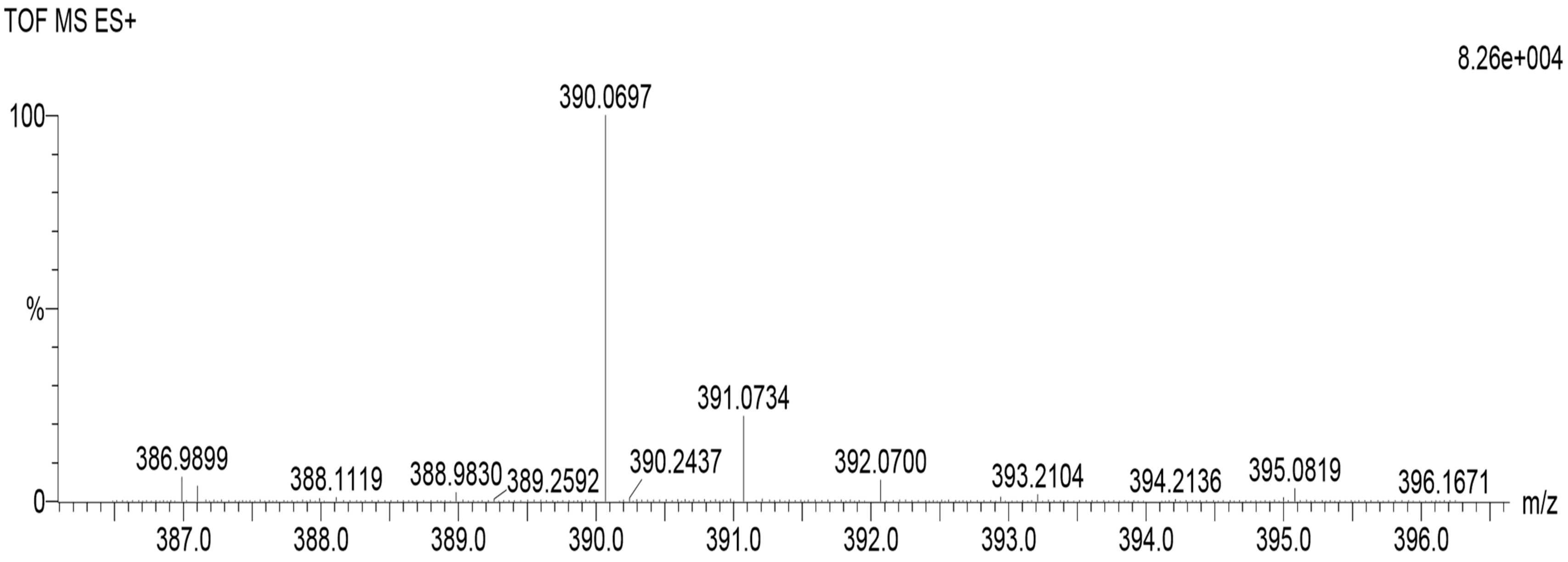

Minimum:				-1.5			
Maximum:		5.0	5.0	500.0			
Mass	Calc. Mass	mDa	PPM	DBE	i-FIT	i-FIT (Norm)	Formula
390.0697	390.0688	0.9	2.3	13.5	550.7	0.0	C19 H14 N3 O2 Na S F

2-[(3-chlorobenzyl)thio]-5-[(quinolin-8-yloxy)methyl]-1,3,4-oxadiazole (**4p**)

^{1}H NMR

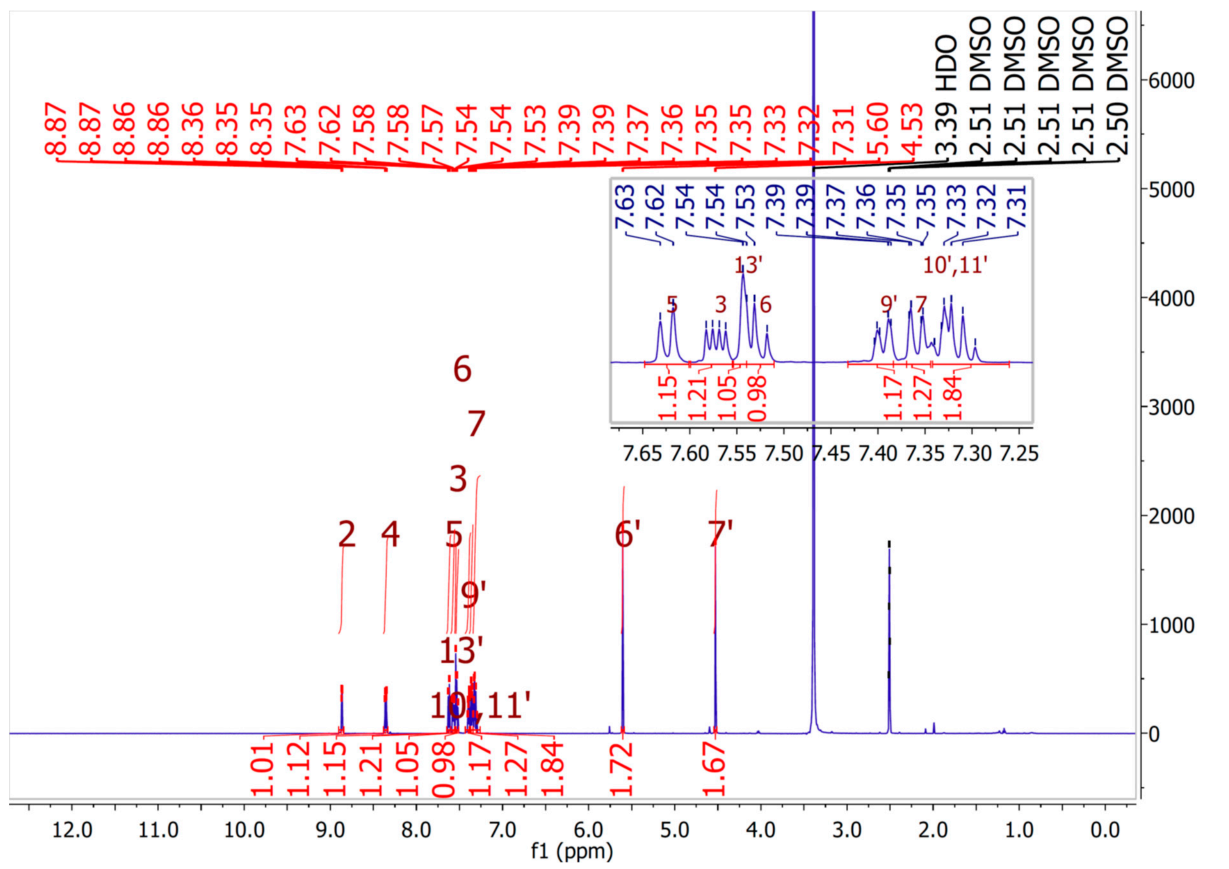

^{13}C NMR

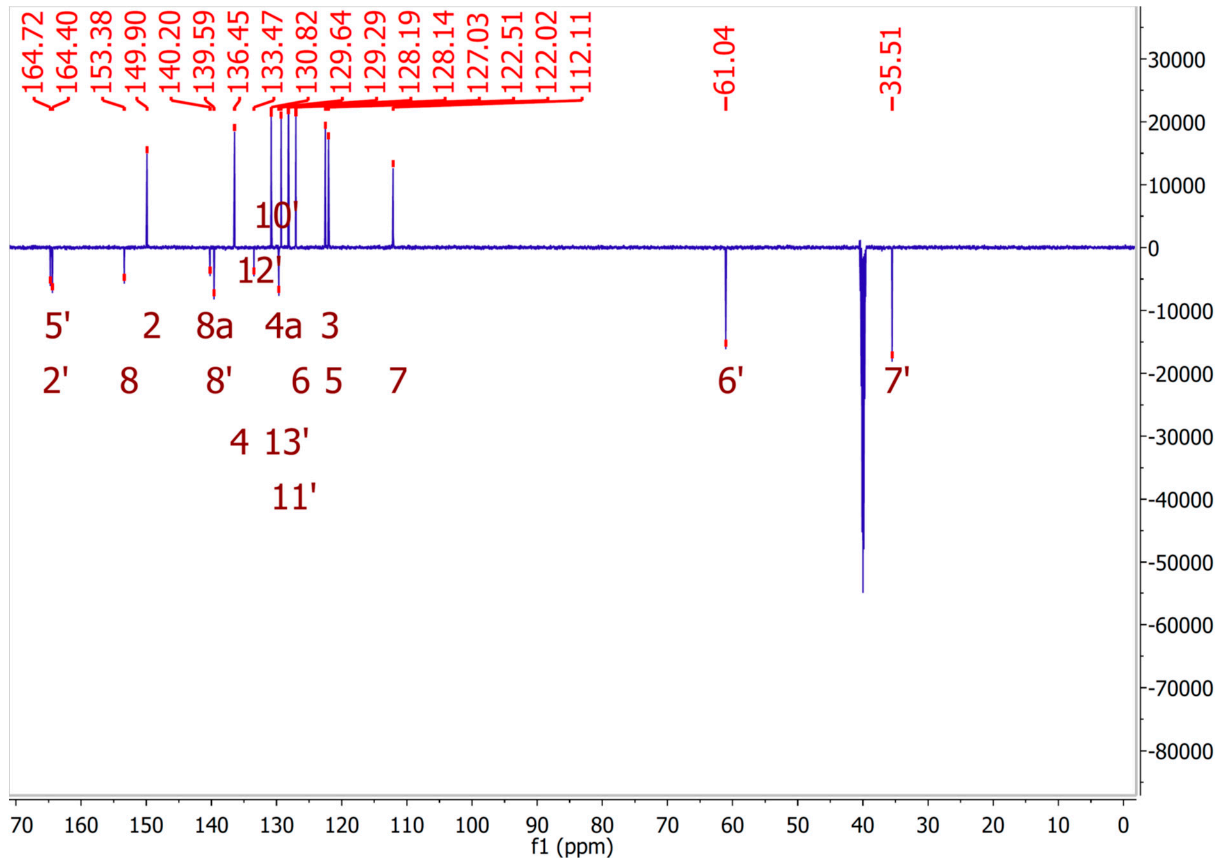

HRMS

Elemental Composition Report_Compound 4p

Single Mass Analysis

Tolerance = 5.0 PPM / DBE: min = -1.5, max = 500.0
Element prediction: Off
Number of isotope peaks used for i-FIT = 3

Monoisotopic Mass, Even Electron Ions
67 formula(e) evaluated with 1 results within limits (all results (up to 1000) for each mass) Elements Used:
C: 15-20 H: 10-15 N: 0-5 O: 0-5 Na: 1-1 S: 0-1 Cl: 0-1

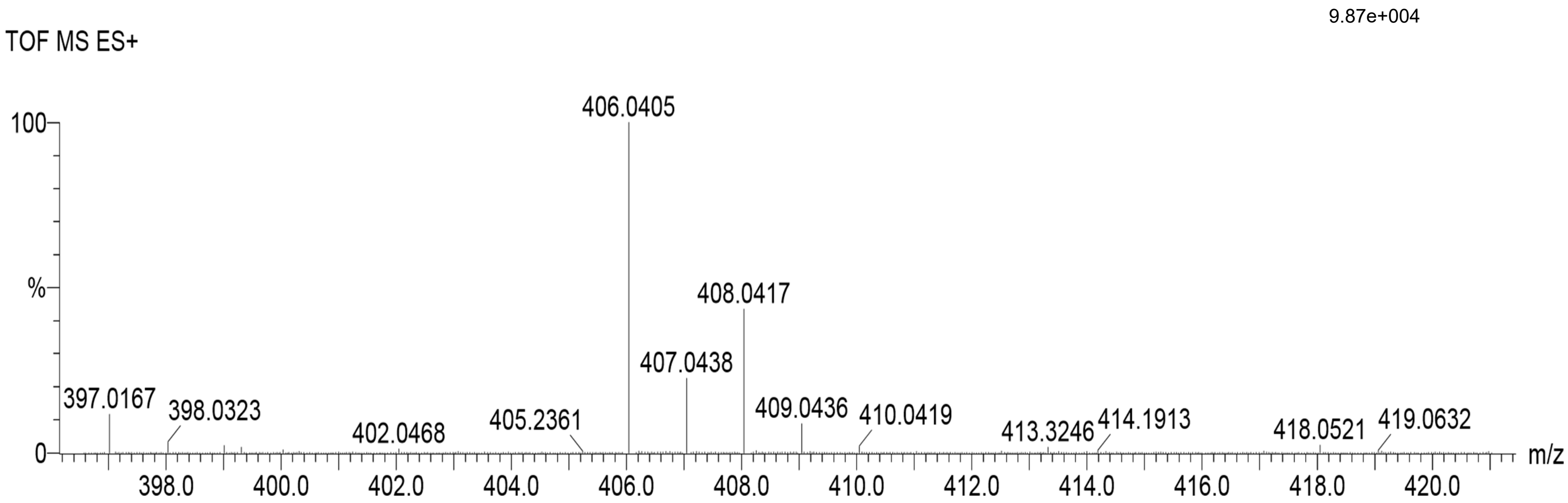

Minimum:				-1.5			
Maximum:		5.0	5.0	500.0			
Mass	Calc. Mass	mDa	PPM	DBE	i-FIT	i-FIT (Norm)	Formula
406.0405	406.0393	1.2	3.0	13.5	538.1	0.0	C19 H14 N3 O2 Na S Cl

2-[(3-bromobenzyl)thio]-5-[(quinolin-8-yloxy)methyl]-1,3,4-oxadiazole (**4q**)

^{1}H NMR

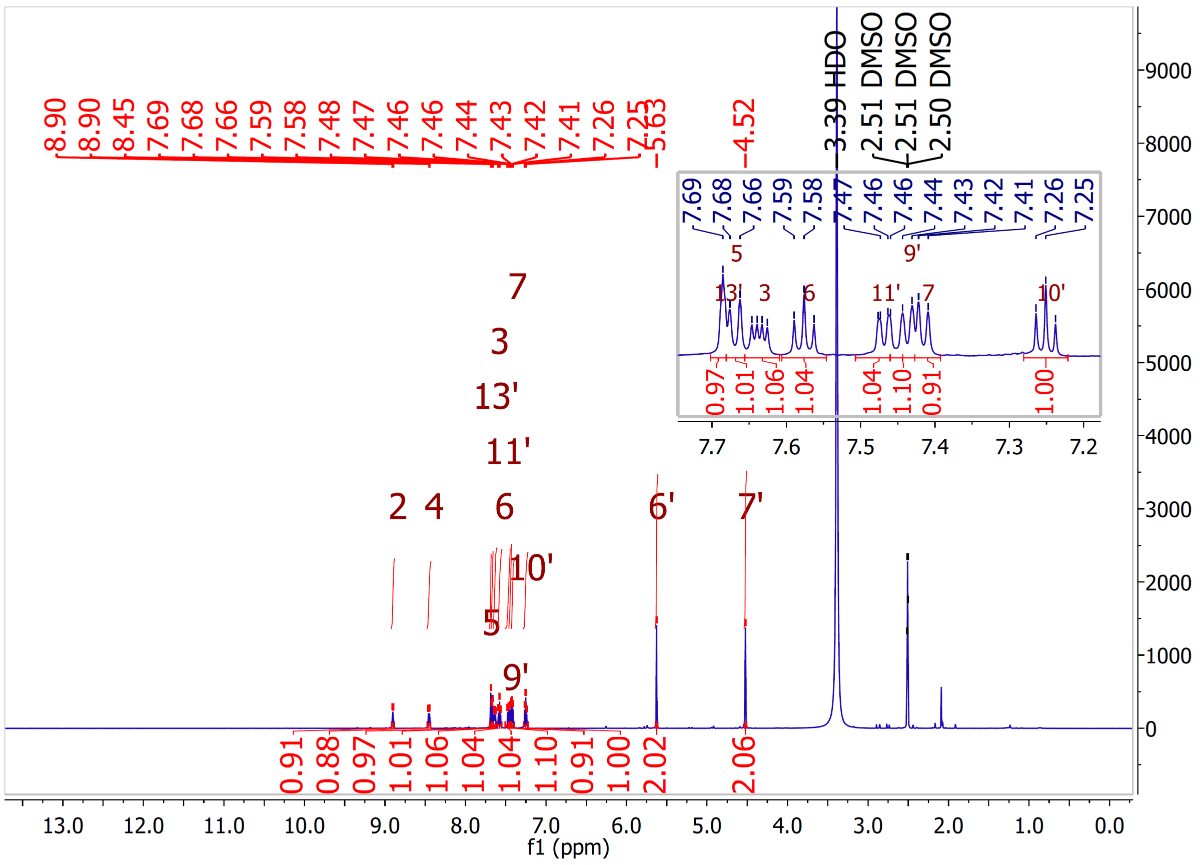

^{13}C NMR

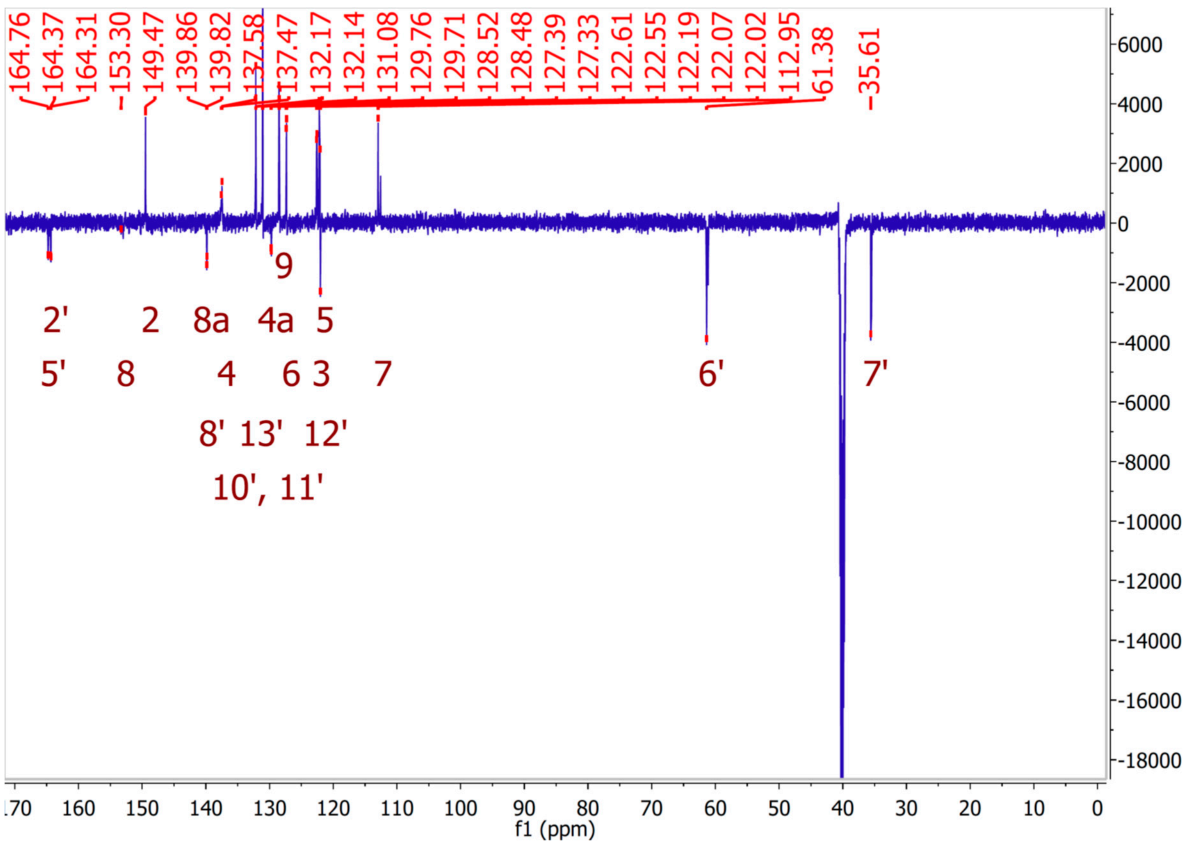

HRMS

Elemental Composition Report_Compound 4q

Single Mass Analysis

Tolerance = 5.0 PPM / DBE: min = -1.5, max = 500.0
Element prediction: Off
Number of isotope peaks used for i-FIT = 3

Monoisotopic Mass, Even Electron Ions
90 formula(e) evaluated with 1 results within limits (all results (up to 1000) for each mass) Elements Used:
C: 15-20 H: 10-15 N: 0-5 O: 0-5 Na: 1-1 S: 0-1 Br: 0-1

TOF MS ES+

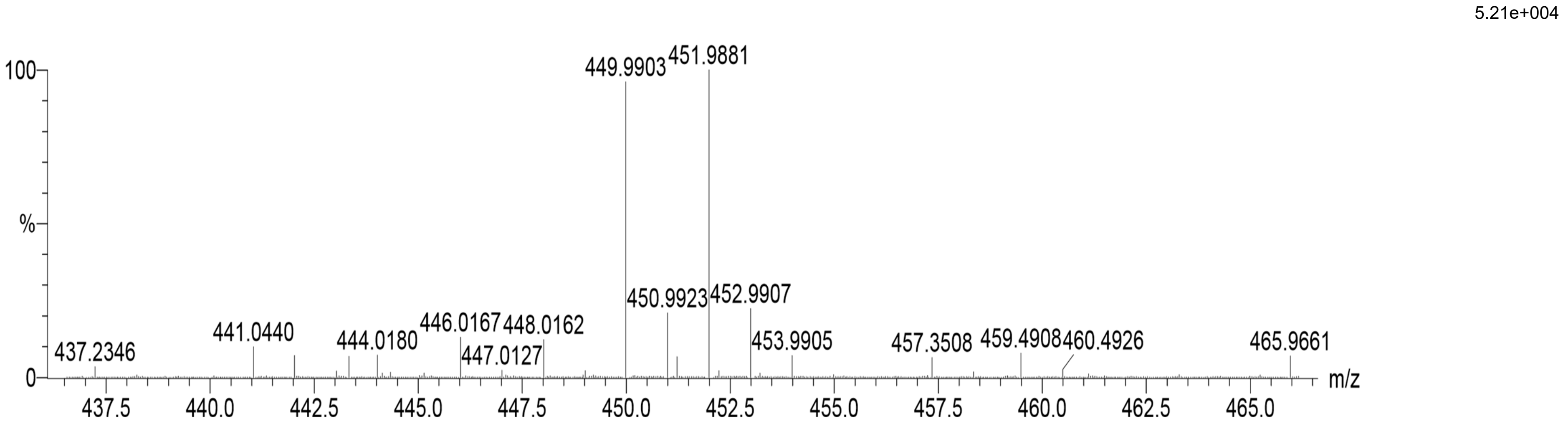

452.5 455.0 457.5 460.0 462.5 465.0

Minimum:				-1.5			
Maximum:		5.0	5.0	500.0			
Mass	Calc. Mass	mDa	PPM	DBE	i-FIT	i-FIT (Norm)	Formula
449.9903	449.9888	1.5	3.3	13.5	492.1	0.0	C19 H14 N3 O2 Na S Br

2-[(2,4-difluorobenzyl)thio)]-5-[(quinolin-8-yloxy)methyl]-1,3,4-oxadiazole (**4r**)

^{1}H NMR

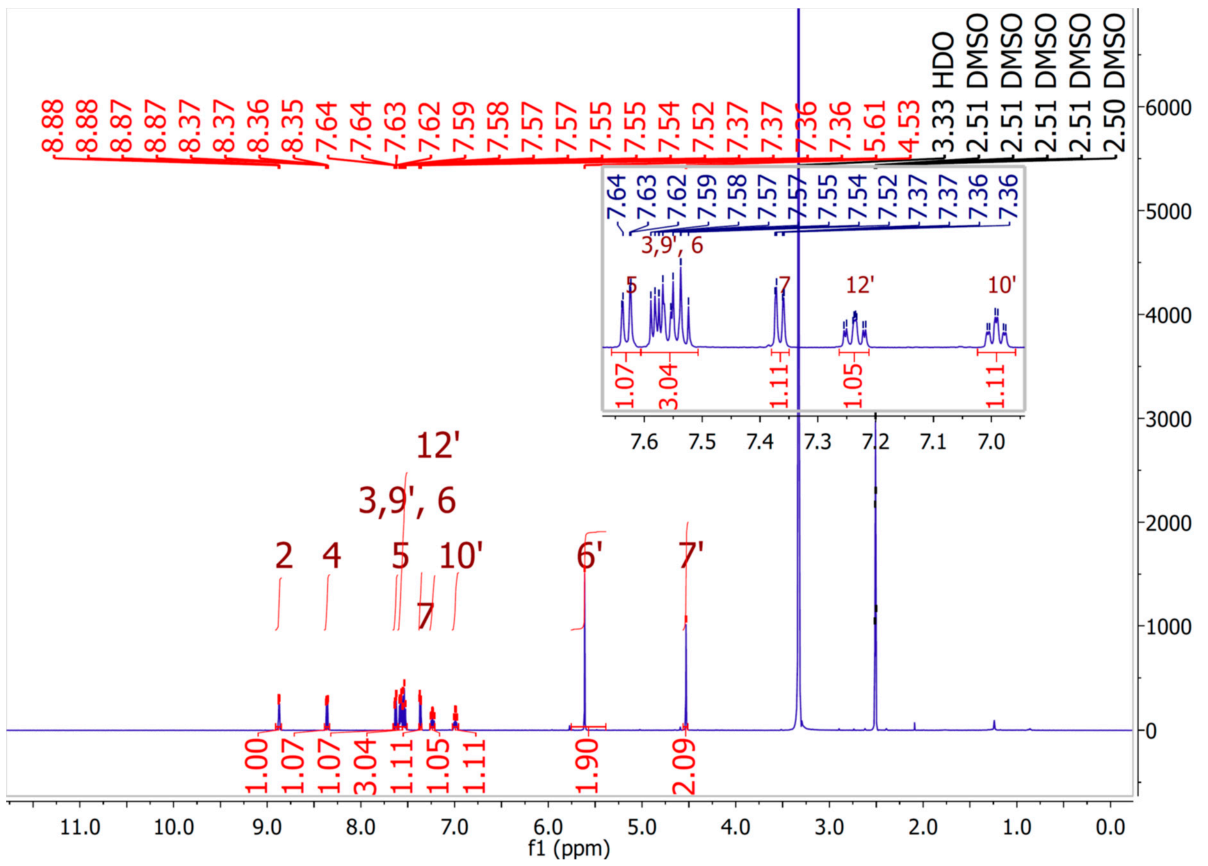

^{13}C NMR

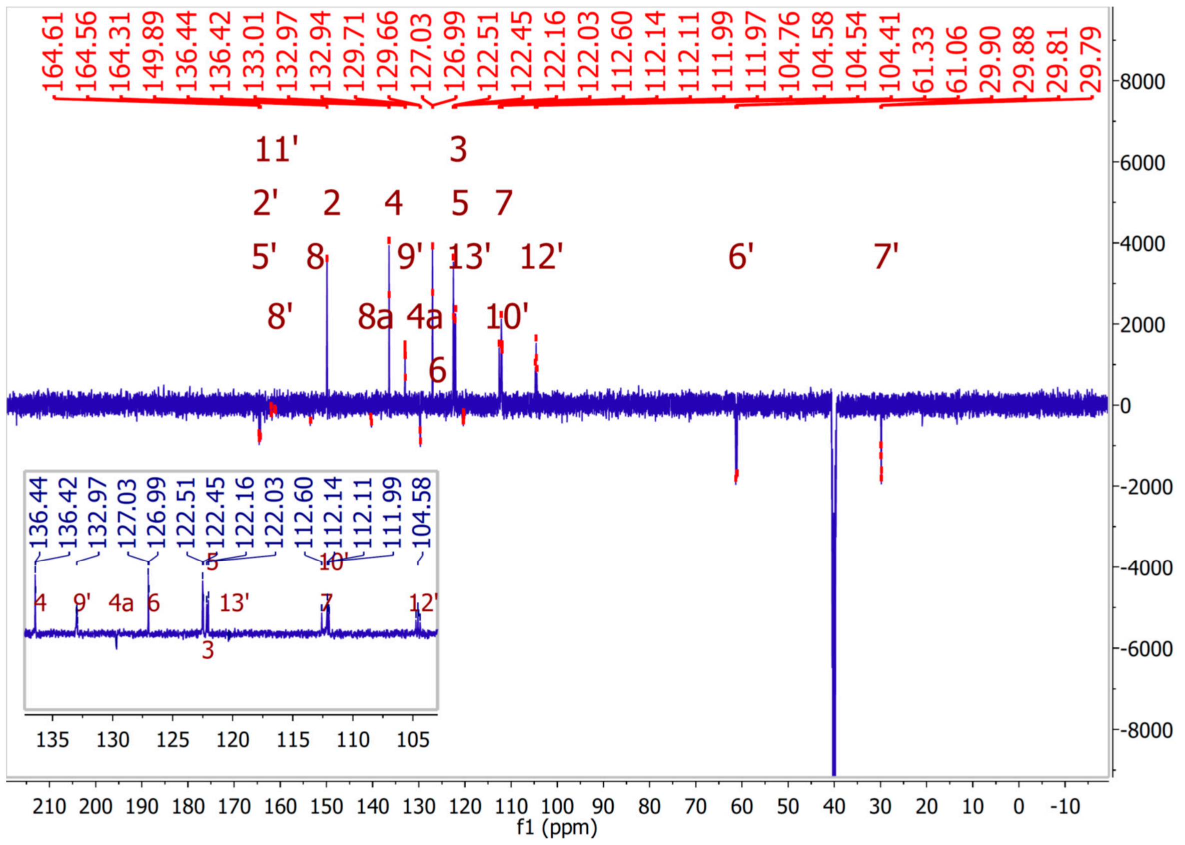

HRMS

Elemental Composition Report_Compound 4r

Single Mass Analysis
Tolerance = 5.0 PPM / DBE: min = -1.5, max = 500.0
Element prediction: Off
Number of isotope peaks used for i-FIT = 3

Monoisotopic Mass, Even Electron Ions
101 formula(e) evaluated with 1 results within limits (all results (up to 1000) for each mass) Elements Used:
C: 15-20 H: 10-15 N: 0-5 O: 0-5 Na: 1-1 S: 0-1 F: 0-2

TOF MS ES+

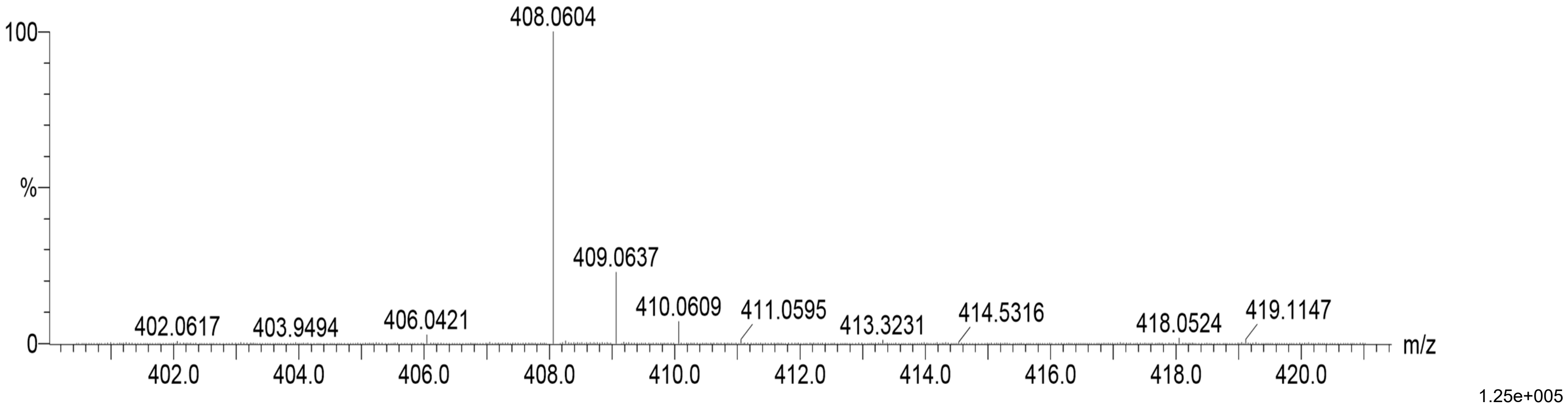

Minimum:				-1.5			
Maximum:		5.0	5.0	500.0			
Mass	Calc. Mass	mDa	PPM	DBE	i-FIT	i-FIT (Norm)	Formula
408.0604	408.0594	1.0	2.5	13.5	569.4	0.0	C19 H13 N3 O2 Na S F2

2-[(3,4-difluorobenzyl)thio]-5-[(quinolin-8-yloxy)methyl]-1,3,4-oxadiazole (**4s**)

^{1}H NMR

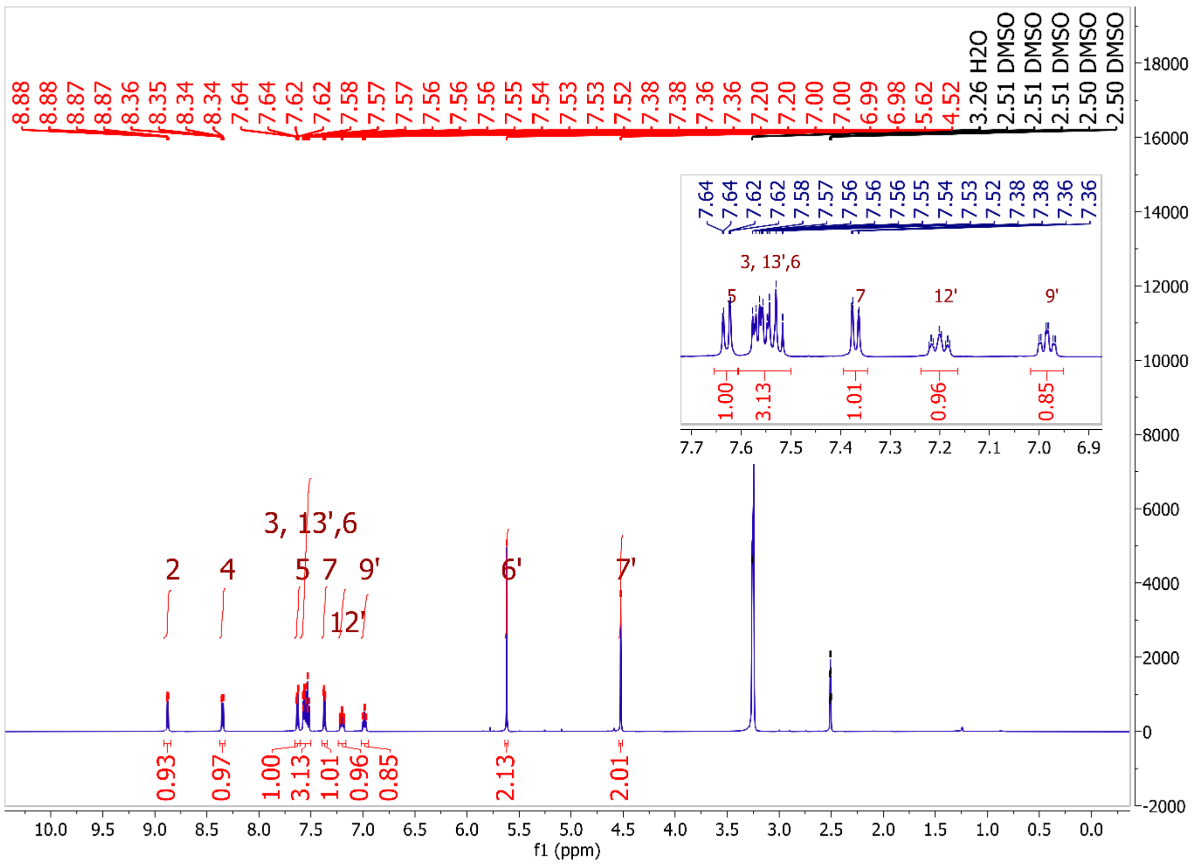

^{13}C NMR

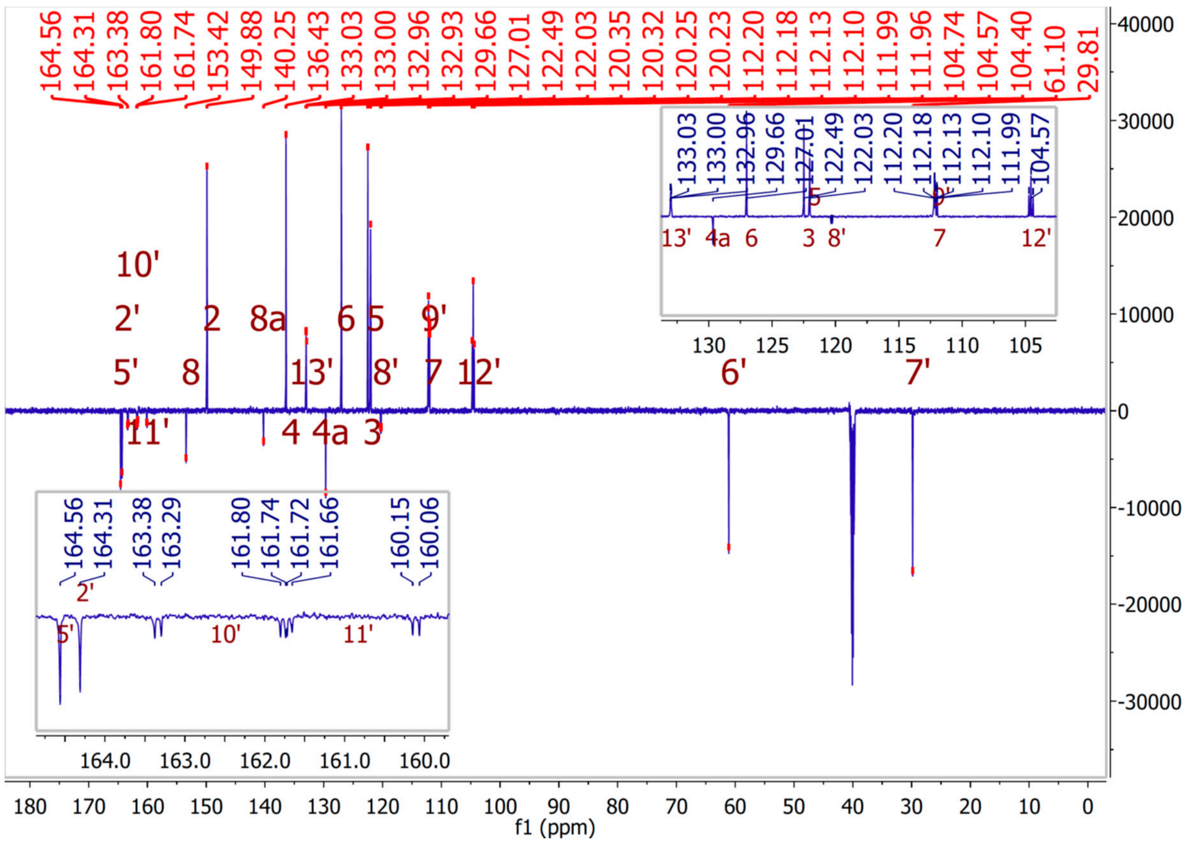

HRMS

Elemental Composition Report_Compound 4s

Single Mass Analysis

Tolerance = 5.0 PPM / DBE: min = -1.5, max = 500.0
Element prediction: Off
Number of isotope peaks used for i-FIT = 3

Monoisotopic Mass, Even Electron Ions
101 formula(e) evaluated with 1 results within limits (all results (up to 1000) for each mass) Elements Used:
C: 15-20 H: 10-15 N: 0-5 O: 0-5 F: 0-2 Na: 1-1 S: 0-1

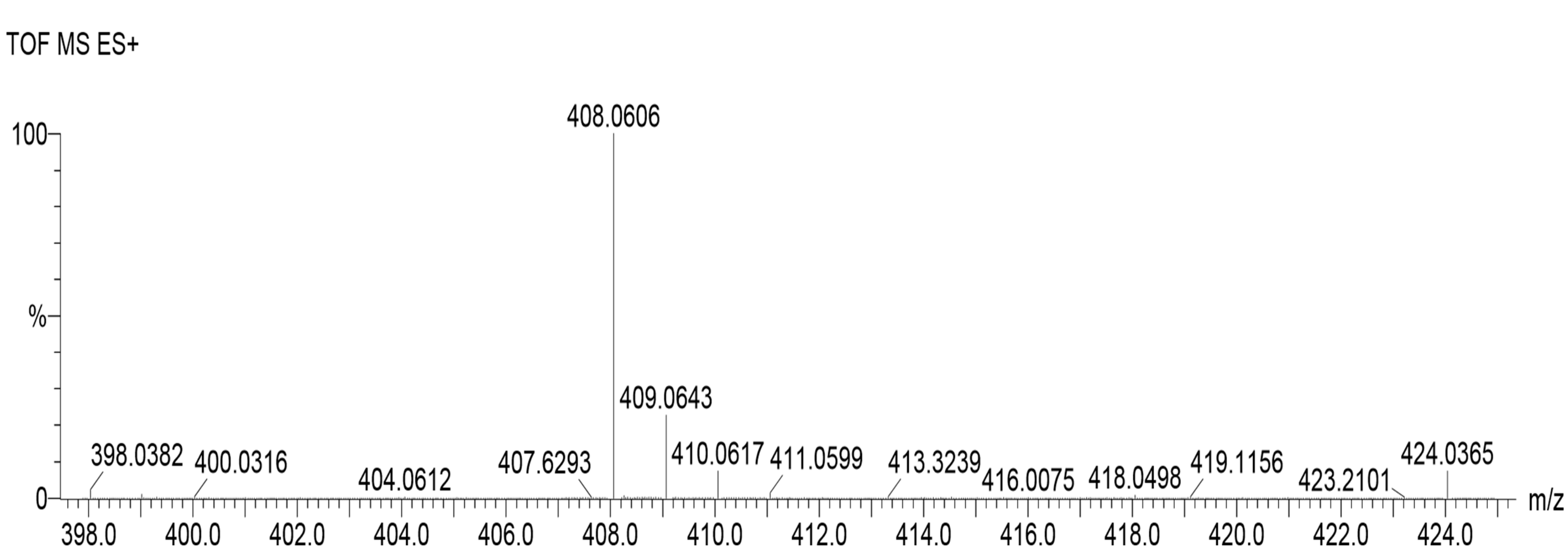

Minimum:				-1.5			
Maximum:		5.0	5.0	500.0			
Mass	Calc. Mass	mDa	PPM	DBE	i-FIT	i-FIT (Norm)	Formula
408.0606	408.0594	1.2	2.9	13.5	597.7	0.0	C19 H13 N3 O2 F2 Na S

2-[(2,4-dichlorobenzyl)thio]-5-[(quinolin-8-yloxy)methyl]-1,3,4-oxadiazole (**4t**)

^{1}H NMR

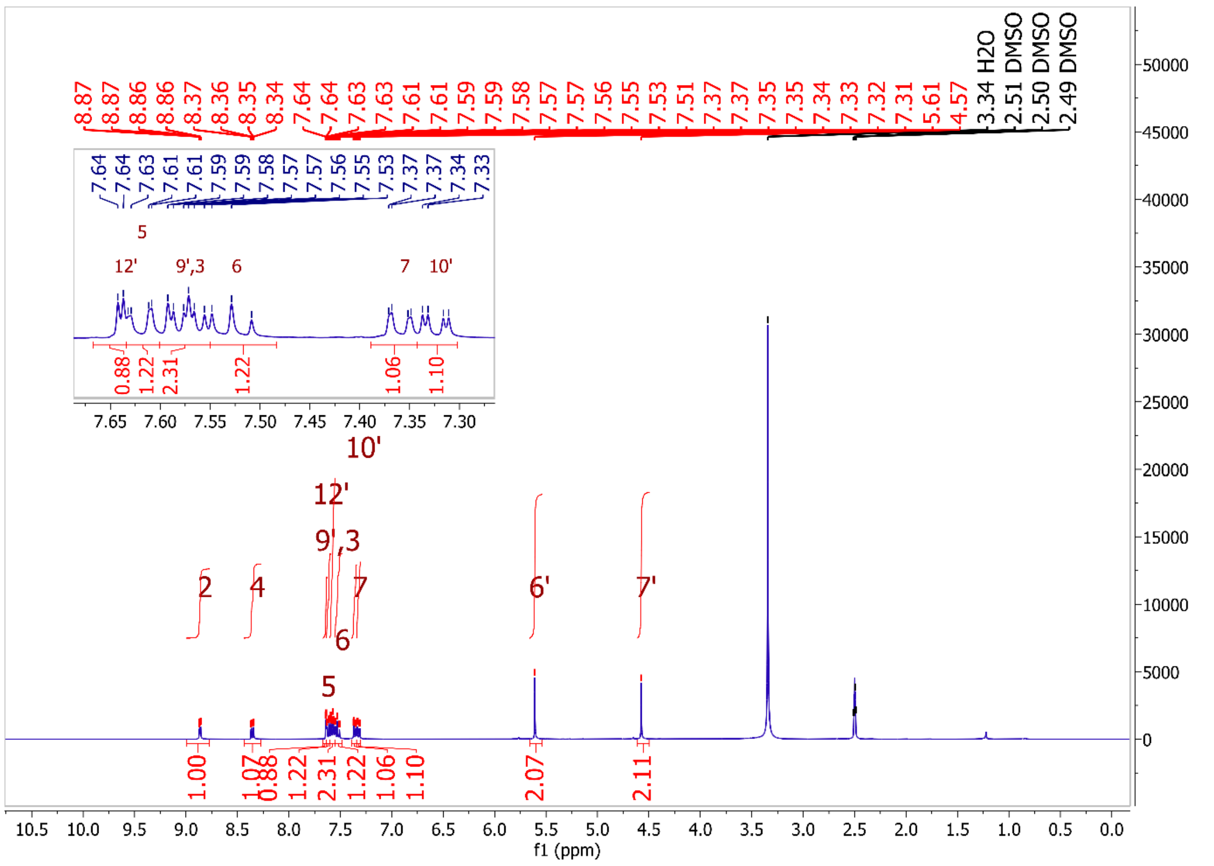

^{13}C NMR

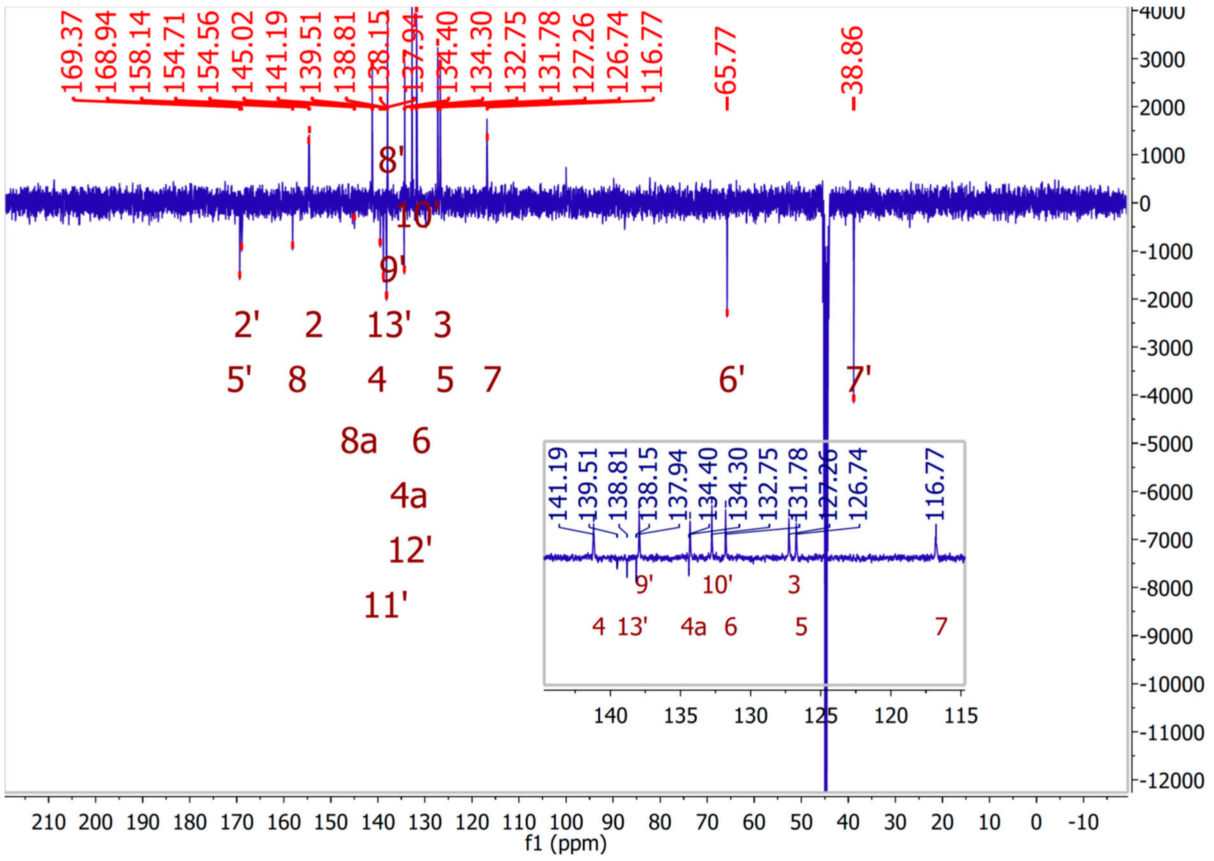

HRMS

Elemental Composition Report_Compound 4t

Single Mass Analysis
Tolerance = 5.0 PPM / DBE: min = -1.5, max = 500.0
Element prediction: Off
Number of isotope peaks used for i-FIT = 3

Monoisotopic Mass, Even Electron Ions
122 formula(e) evaluated with 1 results within limits (all results (up to 1000) for each mass) Elements Used:
C: 15-20 H: 10-15 N: 0-5 O: 0-5 Na: 1-1 S: 0-1 Cl: 0-2

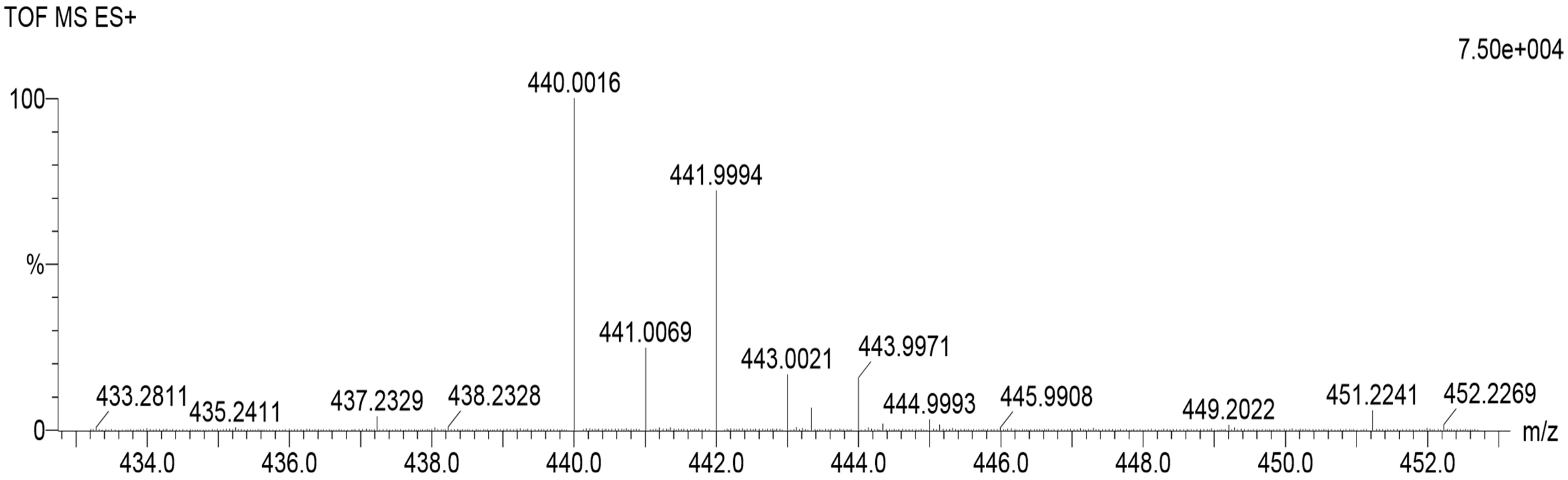

Minimum:				-1.5			
Maximum:		5.0	5.0	500.0			
Mass	Calc. Mass	mDa	PPM	DBE	i-FIT	i-FIT (Norm)	Formula
440.0016	440.0003	1.3	3.0	13.5	531.6	0.0	C19 H13 N3 O2 Na S Cl2

2-{[(1-pentyl-1H-1,2,3-triazol-4-yl)methyl]thio}-5-[(quinolin-8-yloxy)methyl]-1,3,4-oxadiazole (**12a**)

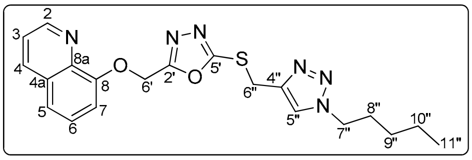

^{1}H NMR

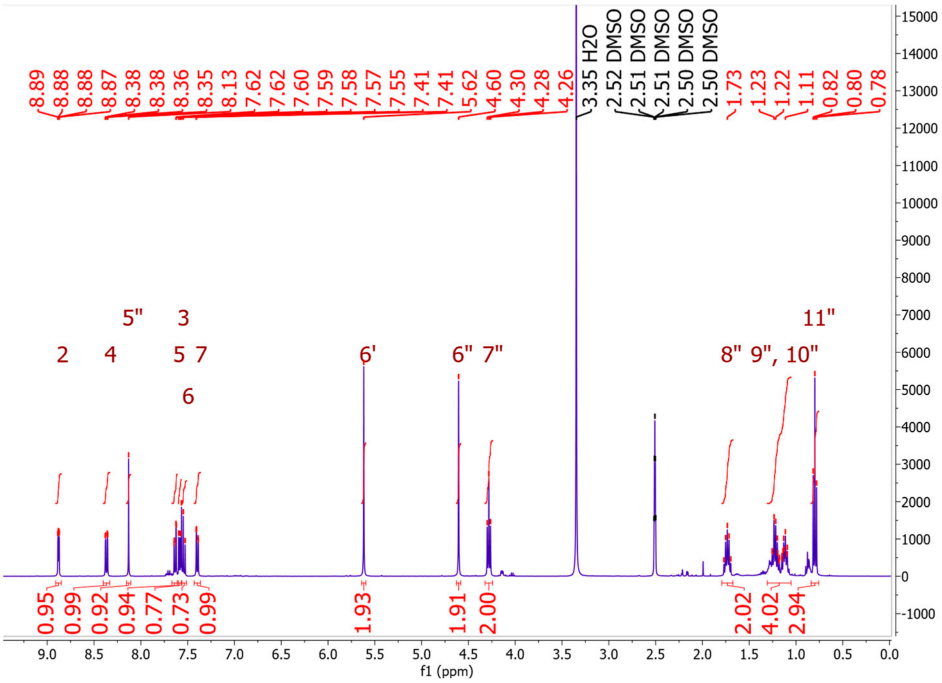

^{13}CNMR

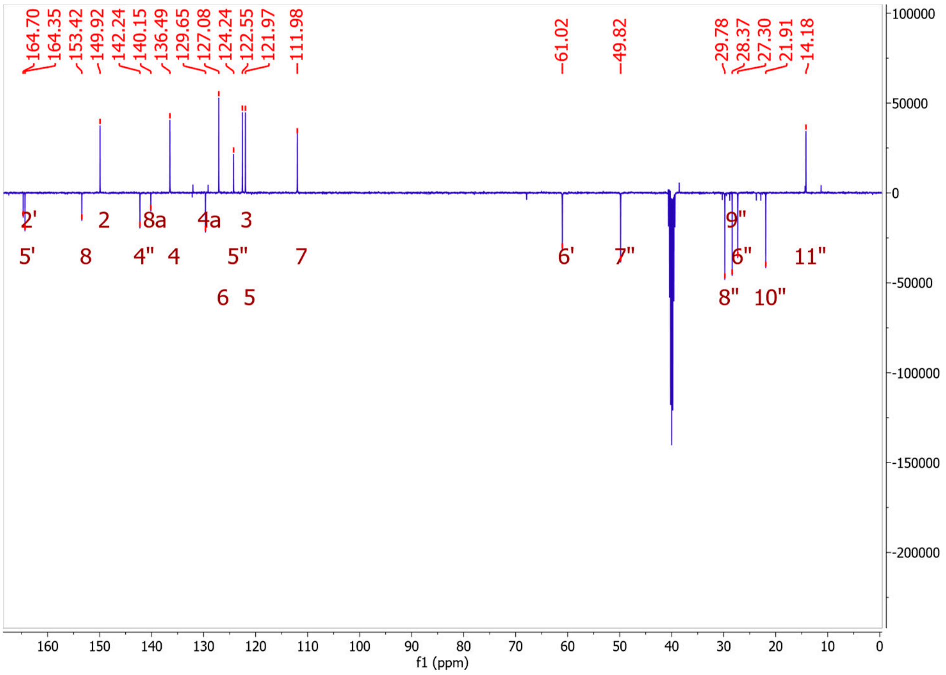

Elemental Composition Report _Compound 12a

Single Mass Analysis

Tolerance = 5.0 PPM / DBE: min = -1.5, max = 500.0
Element prediction: Off
Number of isotope peaks used for i-FIT = 3
Monoisotopic Mass, Even Electron Ions
72 formula(e) evaluated with 1 results within limits (all results (up to 1000) for each mass)
Elements Used:
C: 20-25 H: 20-25 N: 5-10 O: 0-5 S: 0-1 Na: 0-1

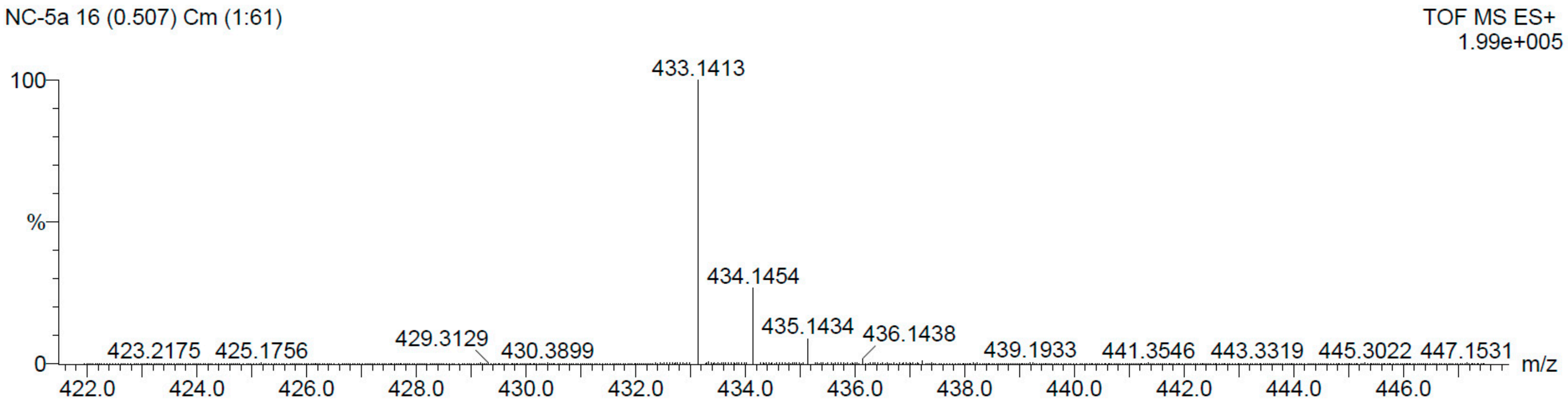

Minimum: -1.5
Maximum: 5.0 5.0 500.0

Mass	Calc. Mass	mDa	PPM	DBE	i-FIT	i-FIT (Norm)	Formula
433.1413	433.1423	-1.0	-2.3	12.5	544.5	0.0	C20 H22 N6 O2 S Na

2-{[(1-hexyl-1H-1,2,3-triazol-4-yl)methyl]thio}-5-[(quinolin-8-yloxy)methyl]-1,3,4-oxadiazole (**12b**)

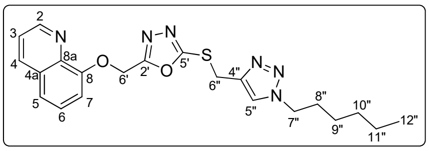

^{1}H NMR

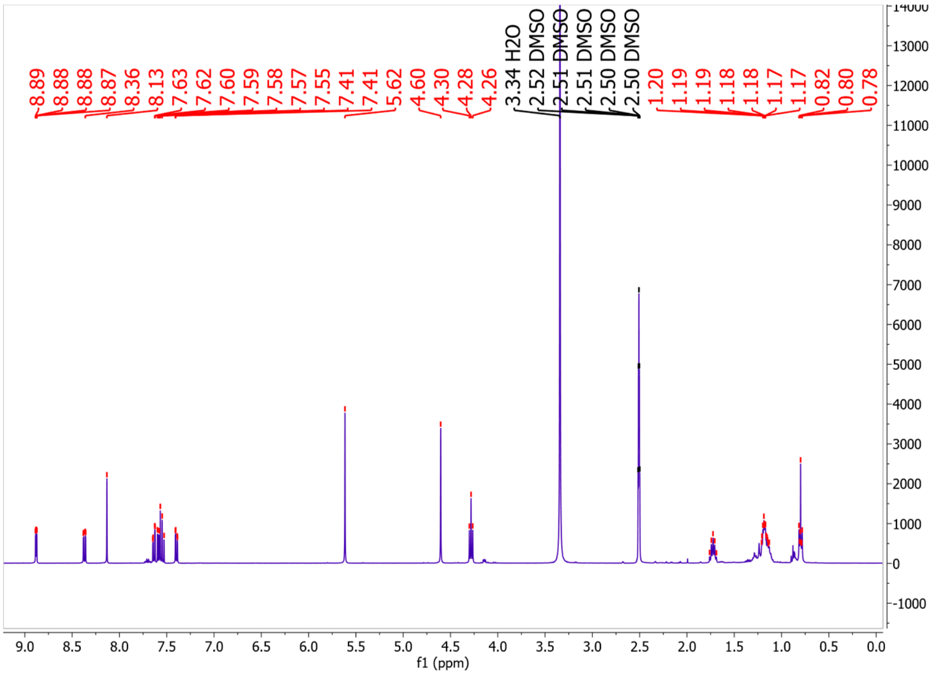

^{13}C NMR

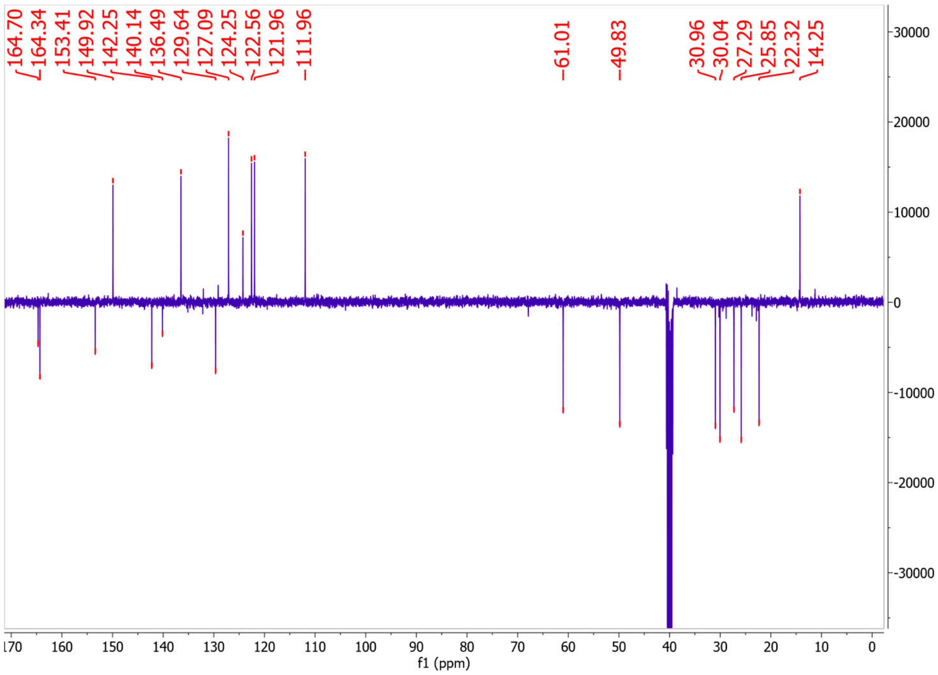

Elemental Composition Report _Compound 12b

Single Mass Analysis

Tolerance = 5.0 PPM / DBE: min = -1.5, max = 500.0
Element prediction: Off
Number of isotope peaks used for i-FIT = 3
Monoisotopic Mass, Even Electron Ions
64 formula(e) evaluated with 1 results within limits (all results (up to 1000) for each mass)
Elements Used:
C: 20-25 H: 20-25 N: 5-10 O: 0-5 Na: 0-1 S: 0-1

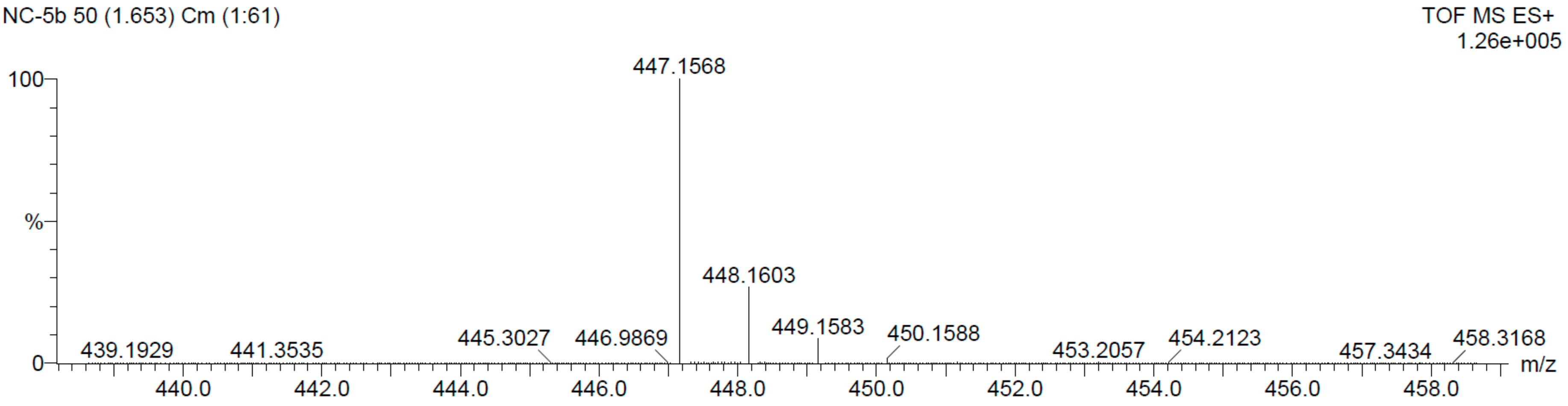


```
Minimum: -1.5
Maximum: 5.0 5.0 500.0
Mass Calc. Mass mDa PPM DBE i-FIT i-FIT (Norm) Formula
447.1568 447.1579 -1.1 -2.5 12.5 472.2 0.0 C21 H24 N6 O2 Na
```

2-{[(1-benzyl-1H-1,2,3-triazol-4-yl)methyl]thio}-5-[(quinolin-8-yloxy)methyl]-1,3,4-oxadiazole (**12c**)

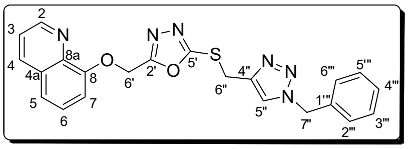

^{1}H NMR

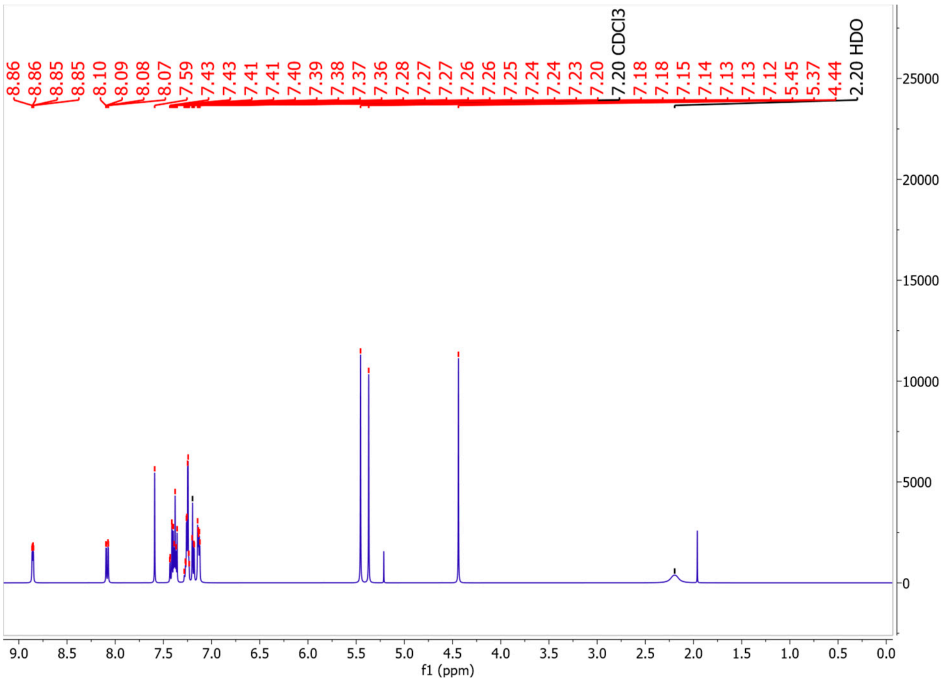

^{13}C NMR

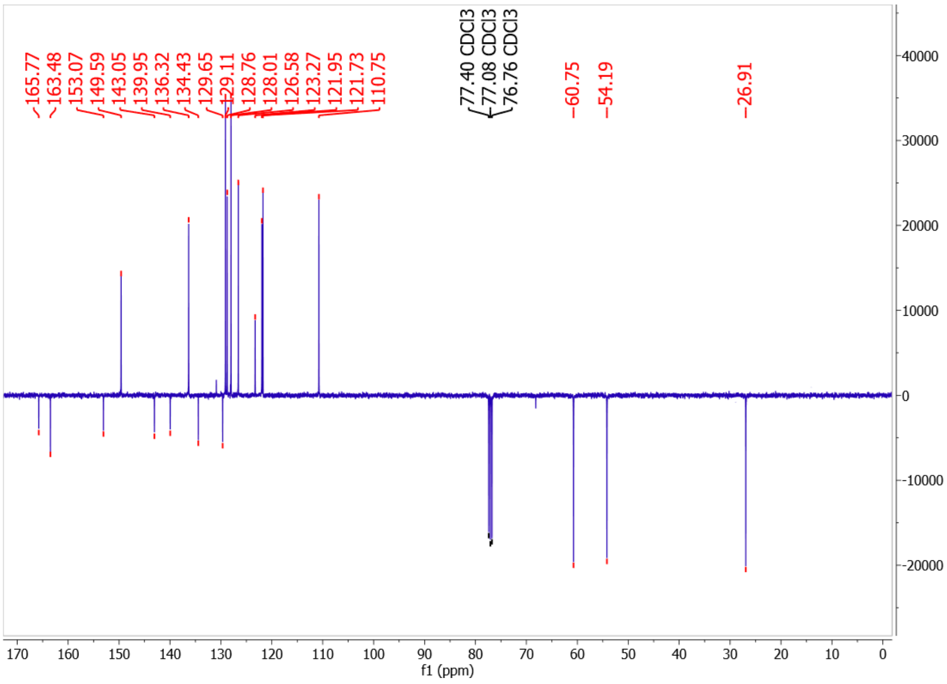

Elemental Composition Report _Compound 12c

Single Mass Analysis

Tolerance = 5.0 PPM / DBE: min = -1.5, max = 500.0
Element prediction: Off
Number of isotope peaks used for i-FIT = 3
Monoisotopic Mass, Even Electron Ions
63 formula(e) evaluated with 1 results within limits (all results (up to 1000) for each mass)
Elements Used:
C: 20-25 H: 15-20 N: 5-10 O: 0-5 Na: 0-1 S: 0-1

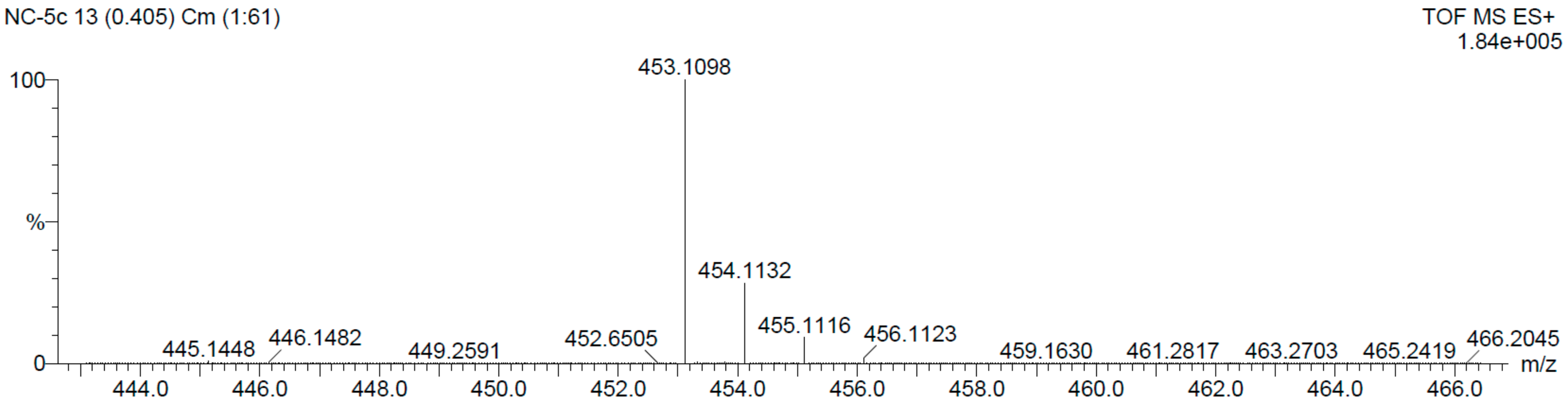

Minimum: -1.5
Maximum: 5.0 5.0 500.0

Mass	Calc. Mass	mDa	PPM	DBE	i-FIT	i-FIT (Norm)	Formula
453.1098	453.1110	-1.2	-2.6	16.5	530.5	0.0	C22 H18 N6 O2 Na S

2-{[(1-(4-chlorobenzyl)-1H-1,2,3-triazol-4-yl)methyl]thio}-5-[(quinolin-8-yloxy)methyl]-1,3,4-oxadiazole (**12d**)

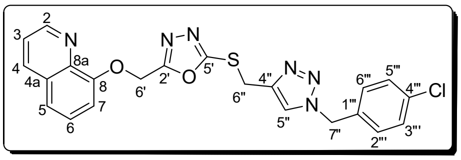

^{1}H NMR

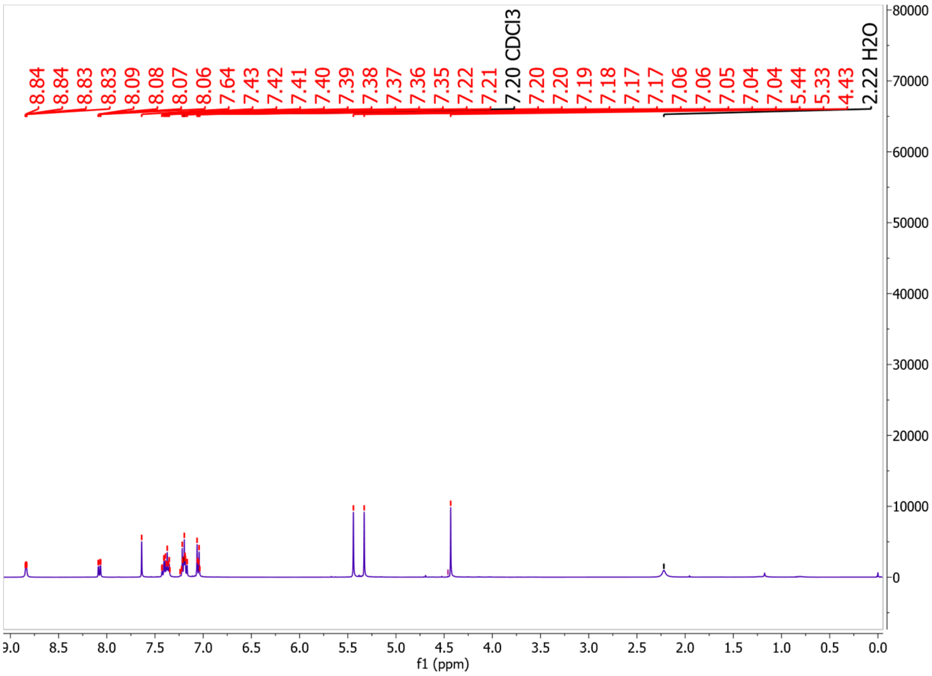

^{13}C NMR

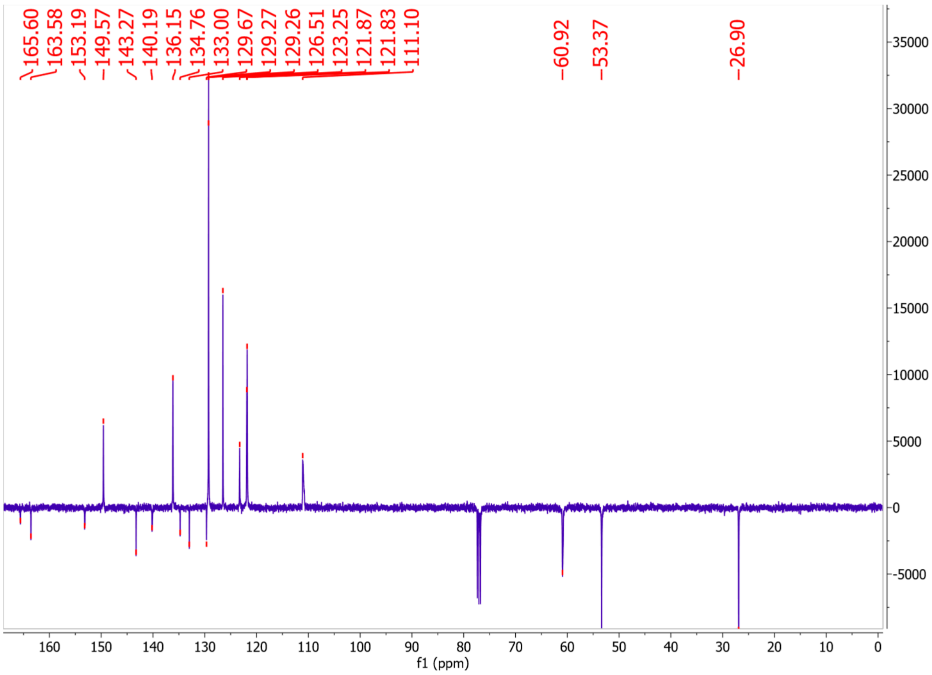

Elemental Composition Report _Compound12d

Single Mass Analysis

Tolerance = 5.0 PPM / DBE: min = -1.5, max = 500.0
Element prediction: Off
Number of isotope peaks used for i-FIT = 3
Monoisotopic Mass, Even Electron Ions
120 formula(e) evaluated with 1 results within limits (all results (up to 1000) for each mass)
Elements Used:
C: 20-25 H: 15-20 N: 5-10 O: 0-5 Na: 0-1 S: 0-1 Cl: 0-1

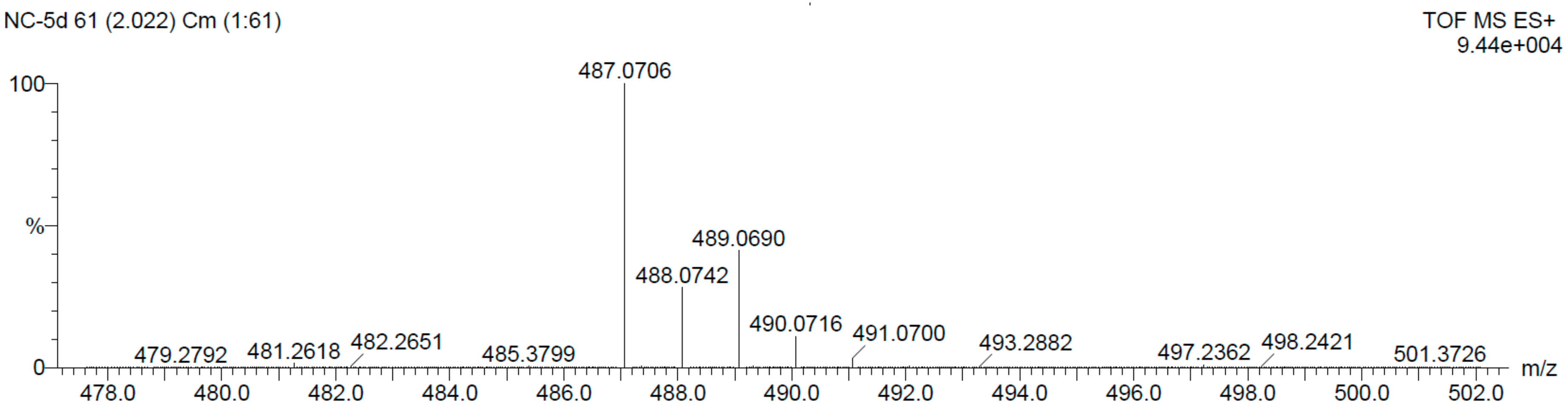

Minimum: -1.5
Maximum: 5.0 5.0 500.0

Mass	Calc. Mass	mDa	PPM	DBE	i-FIT	i-FIT (Norm)	Formula
487.0706	487.0720	-1.4	-2.9	16.5	458.7	0.0	C22 H17 N6 O2 Na Cl

2-{[(1-(4-fluorobenzyl)-1H-1,2,3-triazol-4-yl)methyl]thio}-5-[(quinolin-8-yloxy)methyl]-1,3,4-oxadiazole (**12e**)

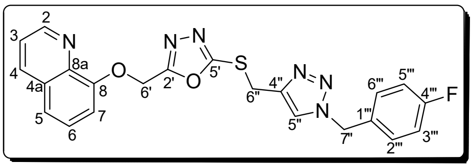

^{1}H NMR

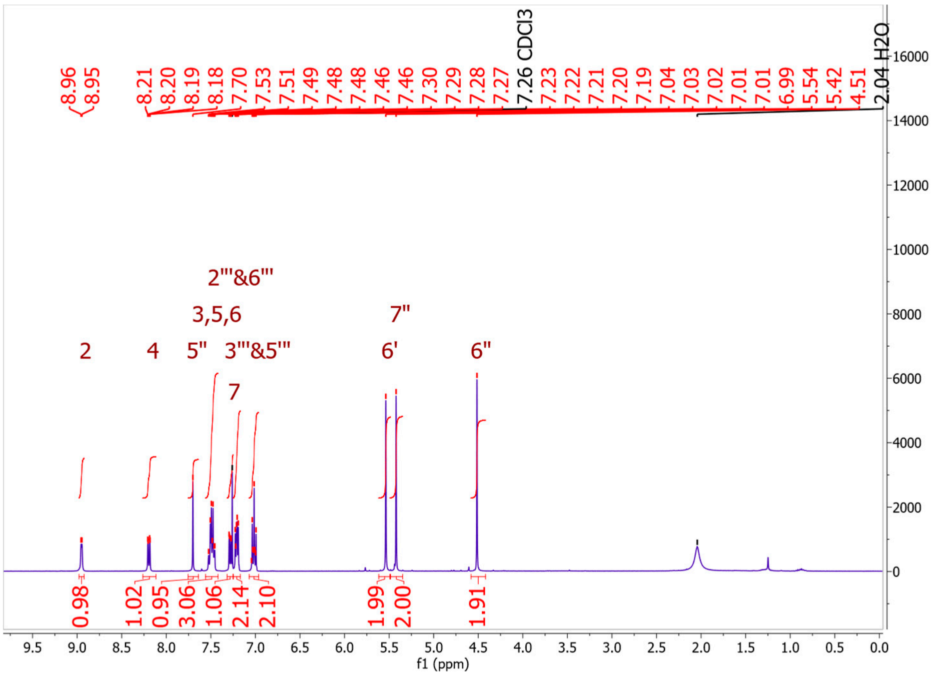

^{13}C NMR

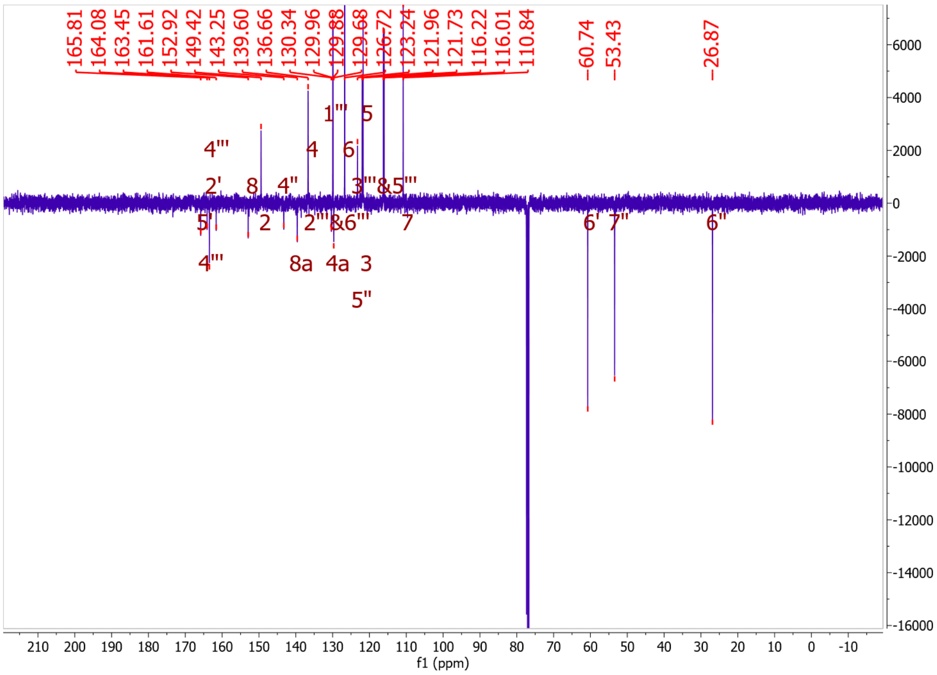

HMBC NMR

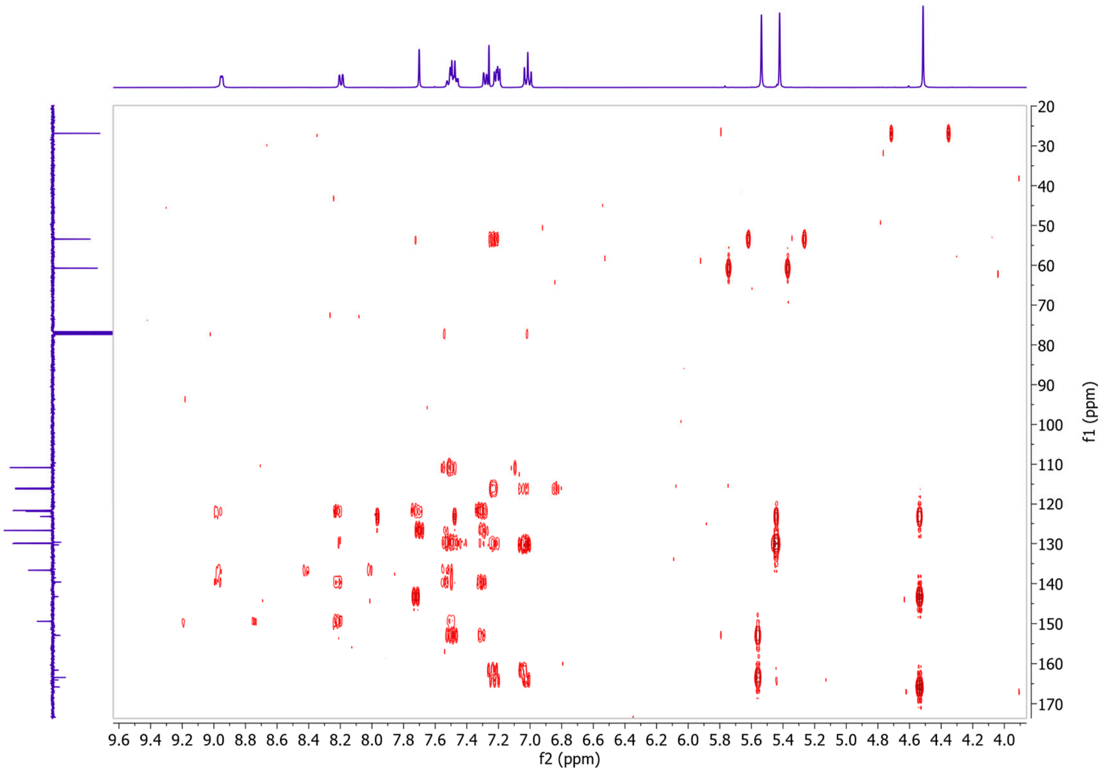

HSQC NMR

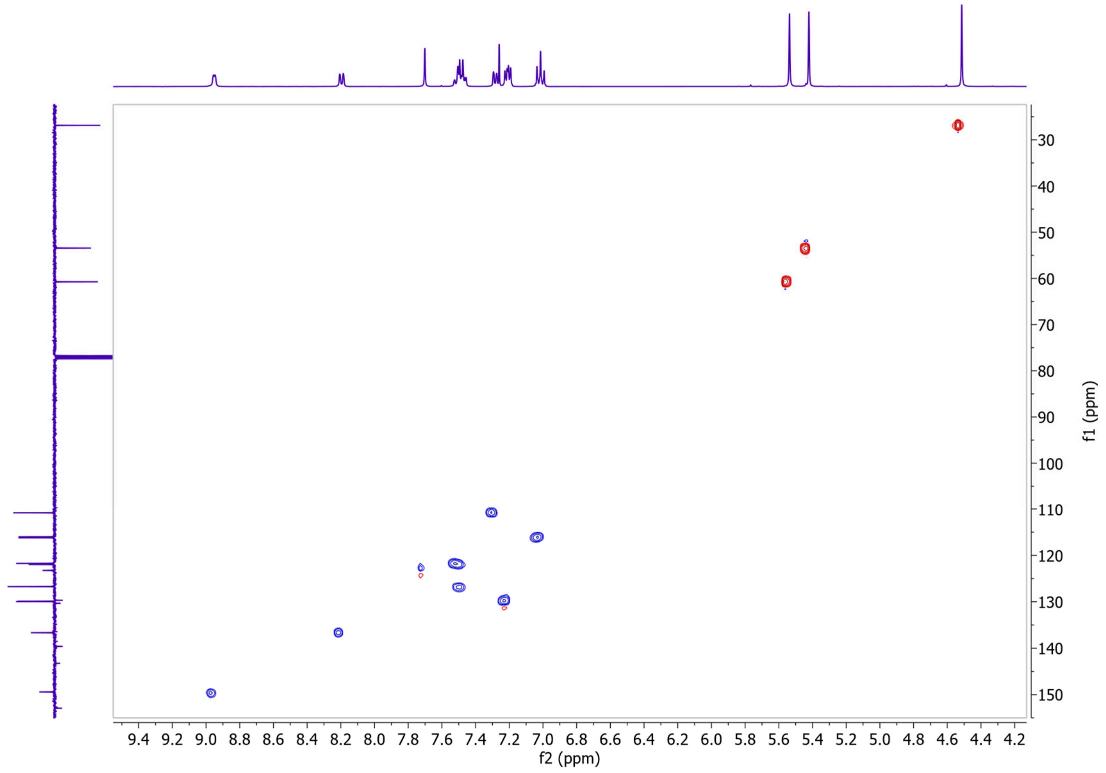

Elemental Composition Report _Compound 12e

Single Mass Analysis
Tolerance = 4.0 PPM / DBE: min = -1.5, max = 500.0
Element prediction: Off
Number of isotope peaks used for i-FIT = 3
Monoisotopic Mass, Even Electron Ions
90 formula(e) evaluated with 1 results within limits (all results (up to 1000) for each mass)
Elements Used:
C: 20-25 H: 15-20 N: 5-10 O: 0-5 Na: 1-1 S: 0-1 Br: 0-1

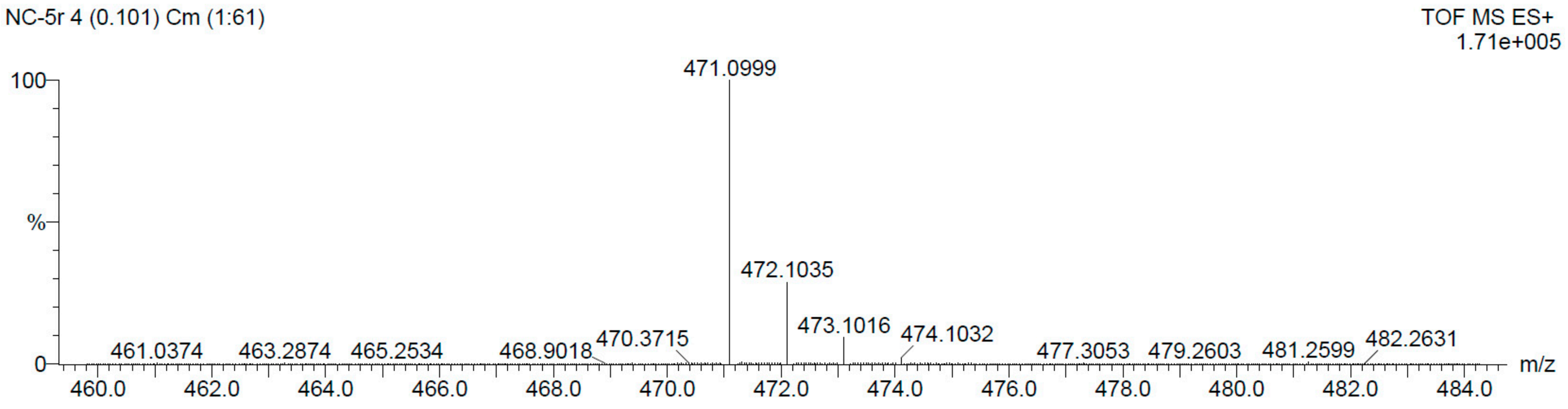

Minimum: -1.5
Maximum: 5.0 4.0 500.0

Mass	Calc. Mass	mDa	PPM	DBE	i-FIT	i-FIT (Norm)	Formula
471.0999	471.1004	-0.5	-1.1	20.5	514.7	0.0	C25 H16 N6 O Na S F

2-{[(1-(4-bromobenzyl)-1H-1,2,3-triazol-4-yl)methyl]thio}-5-[(quinolin-8-yloxy)methyl]-1,3,4-oxadiazole (**12f**)

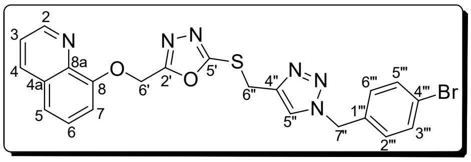

^{1}H NMR

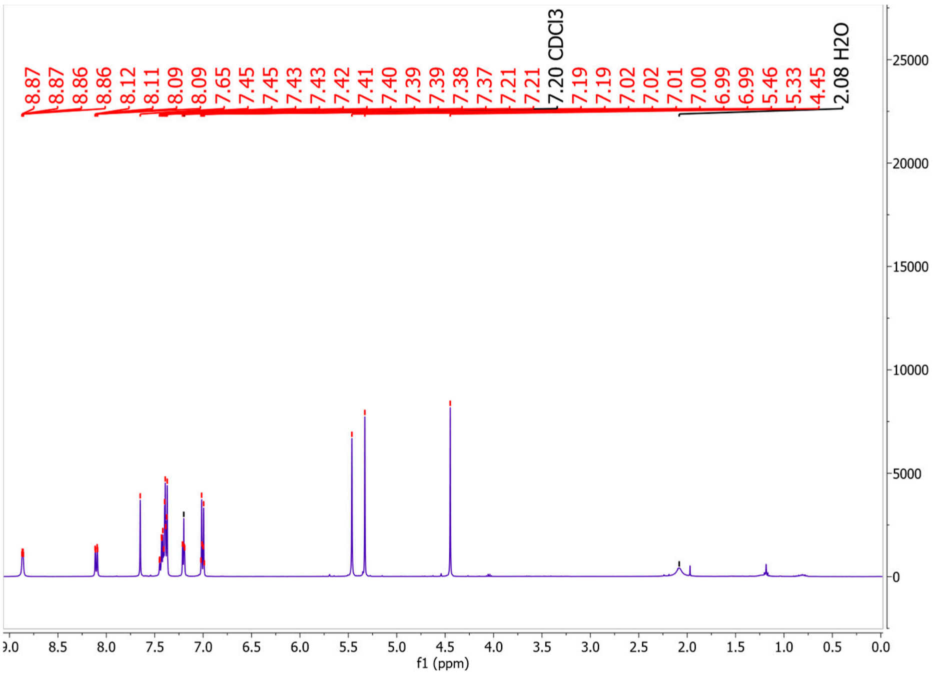

^{13}C NMR

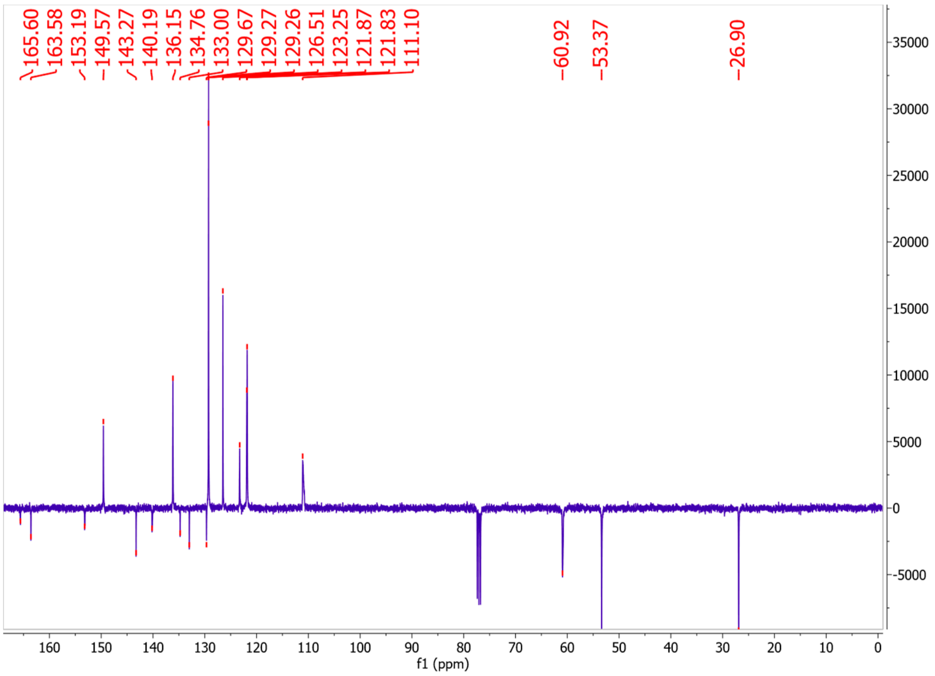

Elemental Composition Report_Compound 12f

Single Mass Analysis

Tolerance = 4.0 PPM / DBE: min = -1.5, max = 500.0
Element prediction: Off
Number of isotope peaks used for i-FIT = 3
Monoisotopic Mass, Even Electron Ions
68 formula(e) evaluated with 1 results within limits (all results (up to 1000) for each mass)
Elements Used:
C: 20-25 H: 15-20 N: 5-10 O: 0-5 Na: 1-1 S: 0-1 Br: 0-1

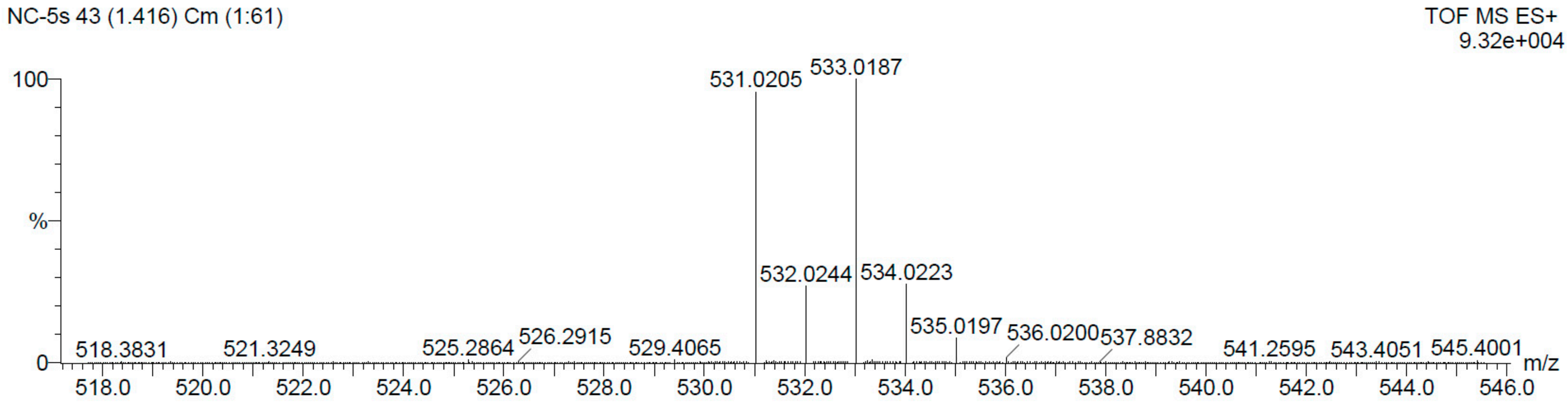

Minimum: -1.5
Maximum: 5.0 4.0 500.0

Mass	Calc. Mass	mDa	PPM	DBE	i-FIT	i-FIT (Norm)	Formula
531.0205	531.0215	-1.0	-1.9	16.5	468.4	0.0	C22 H17 N6 O2 Na S Br

2-{[(1-(4-nitrobenzyl)-1H-1,2,3-triazol-4-yl)methyl]thio}-5-[(quinolin-8-yloxy)methyl]-1,3,4-oxadiazole (**12g**)

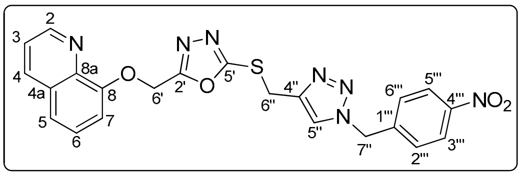

^{1}H NMR

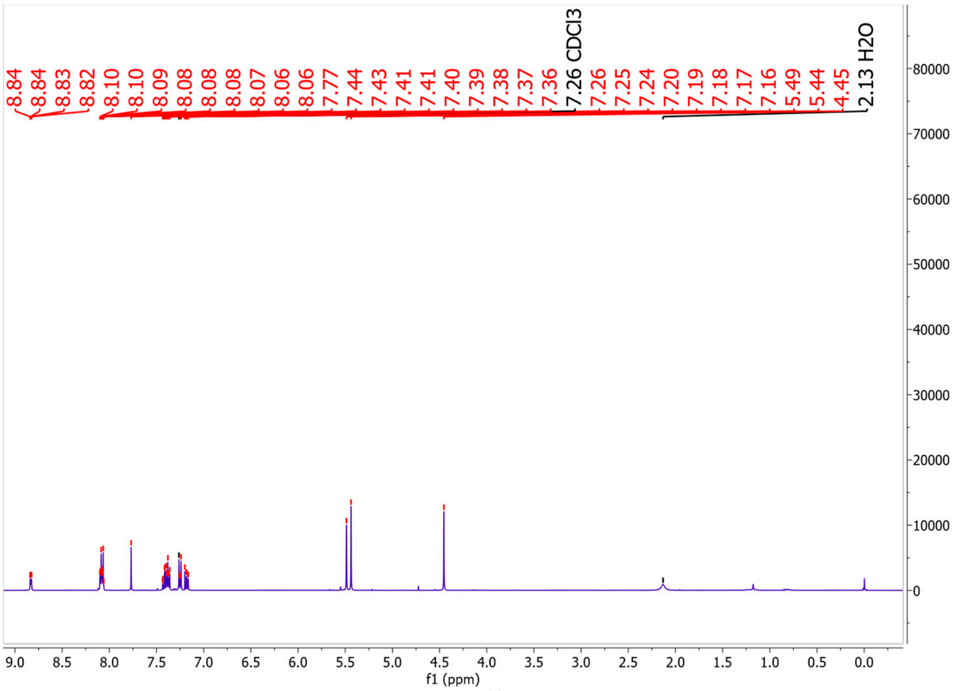

^{13}C NMR

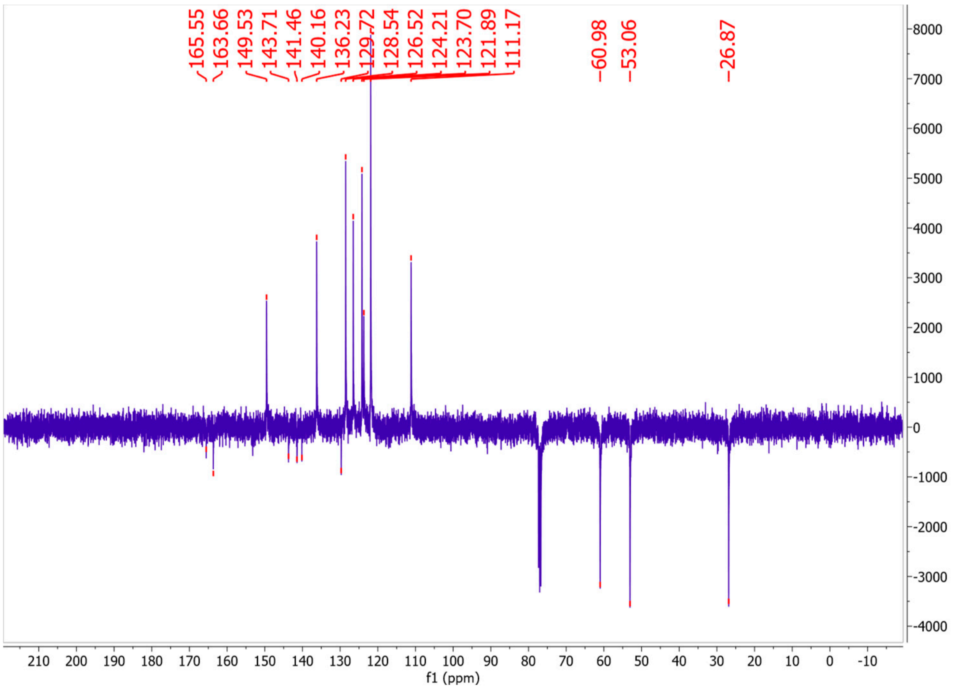

Elemental Composition Report_Compound 12g

Single Mass Analysis

Tolerance = 5.0 PPM / DBE: min = -1.5, max = 500.0
Element prediction: Off
Number of isotope peaks used for i-FIT = 3
Monoisotopic Mass, Even Electron Ions
55 formula(e) evaluated with 1 results within limits (all results (up to 1000) for each mass)
Elements Used:
C: 20-25 H: 15-20 N: 5-10 O: 0-5 Na: 0-1 S: 0-1

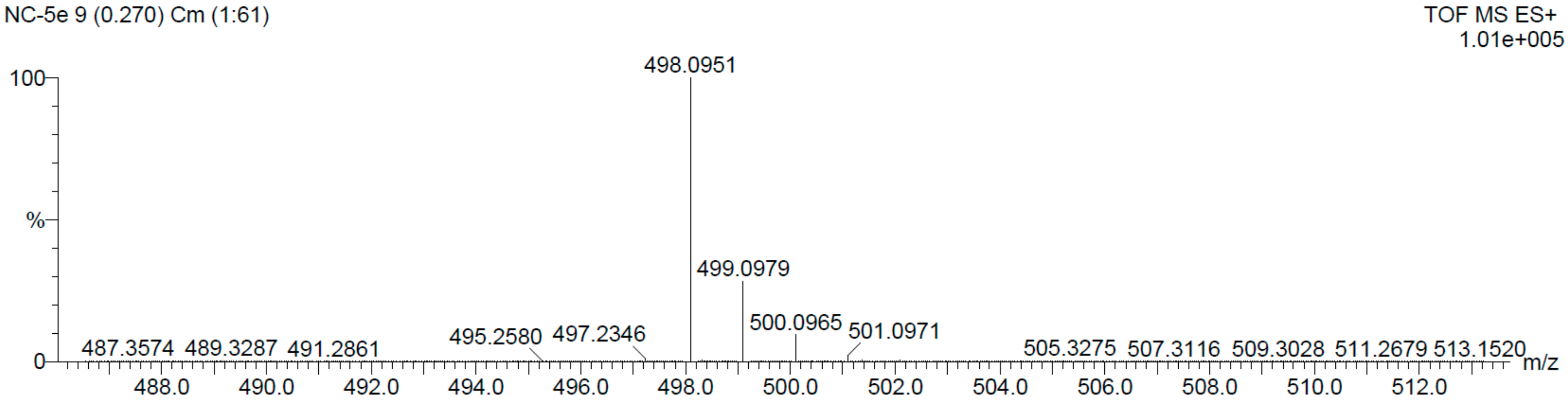

Minimum: -1.5
Maximum: 5.0 5.0 500.0

Mass	Calc. Mass	mDa	PPM	DBE	i-FIT	i-FIT (Norm)	Formula
498.0951	498.0960	-0.9	-1.8	17.5	478.2	0.0	C22 H17 N7 O4 Na S

2-{[(1-(3-chlorobenzyl)-1H-1,2,3-triazol-4-yl)methyl]thio}-5-[(quinolin-8-yloxy)methyl]-1,3,4-oxadiazole (**12h**)

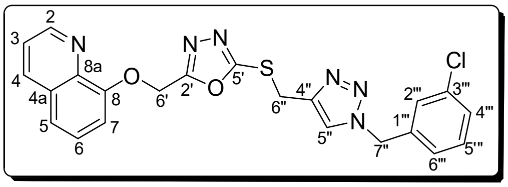

^{1}H NMR

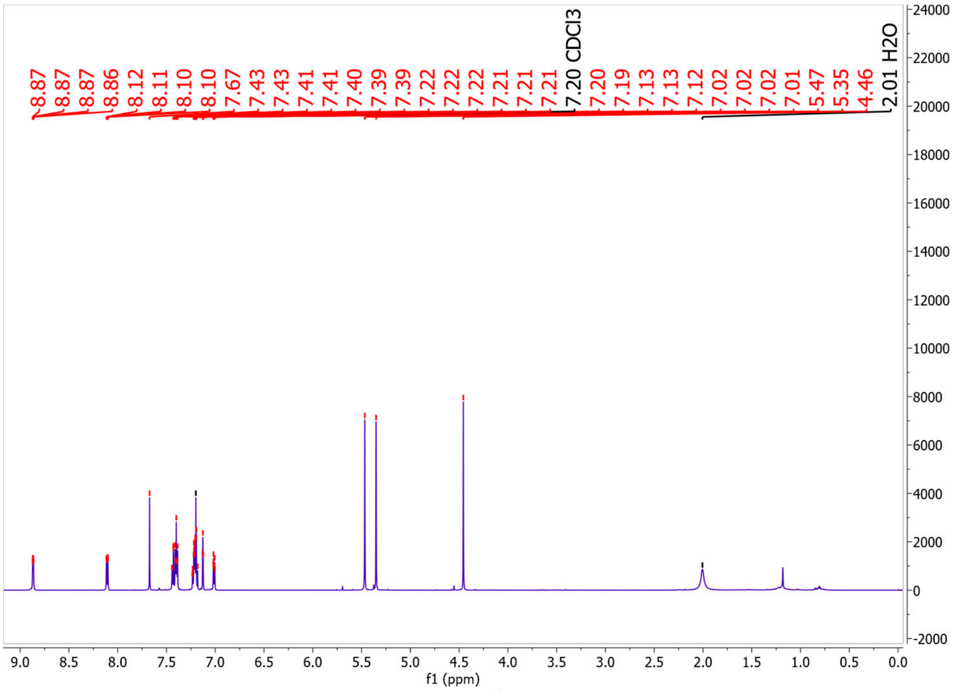

^{13}C NMR

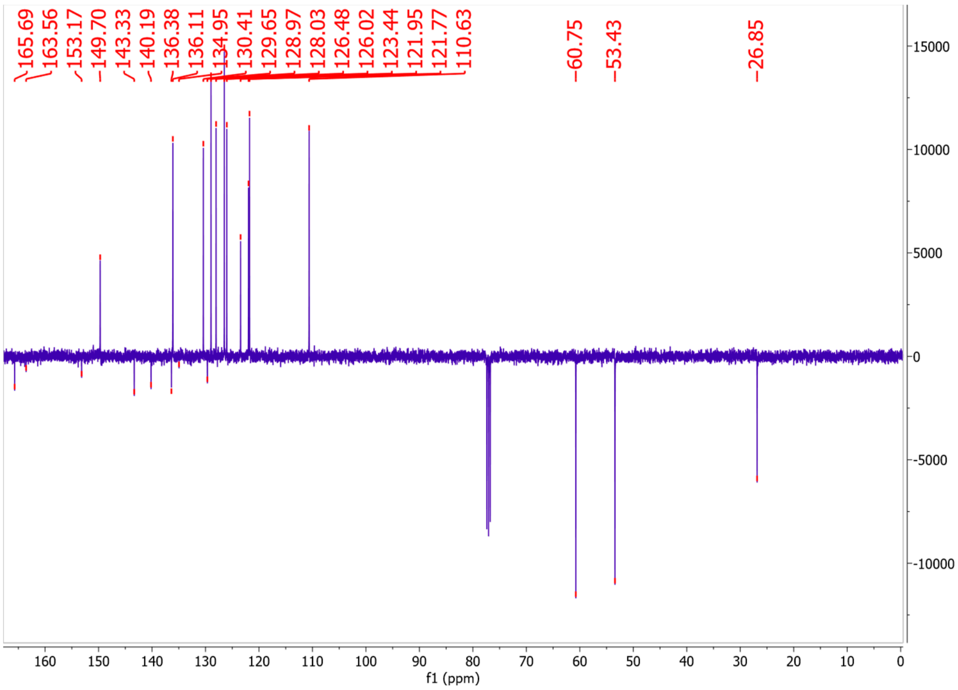

Elemental Composition Report_Compound 12h

Single Mass Analysis
Tolerance = 4.0 PPM / DBE: min = -1.5, max = 500.0
Element prediction: Off
Number of isotope peaks used for i-FIT = 3
Monoisotopic Mass, Even Electron Ions
65 formula(e) evaluated with 1 results within limits (all results (up to 1000) for each mass)
Elements Used:
C: 20-25 H: 15-20 N: 5-10 O: 0-5 Na: 1-1 S: 0-1 Cl: 0-1

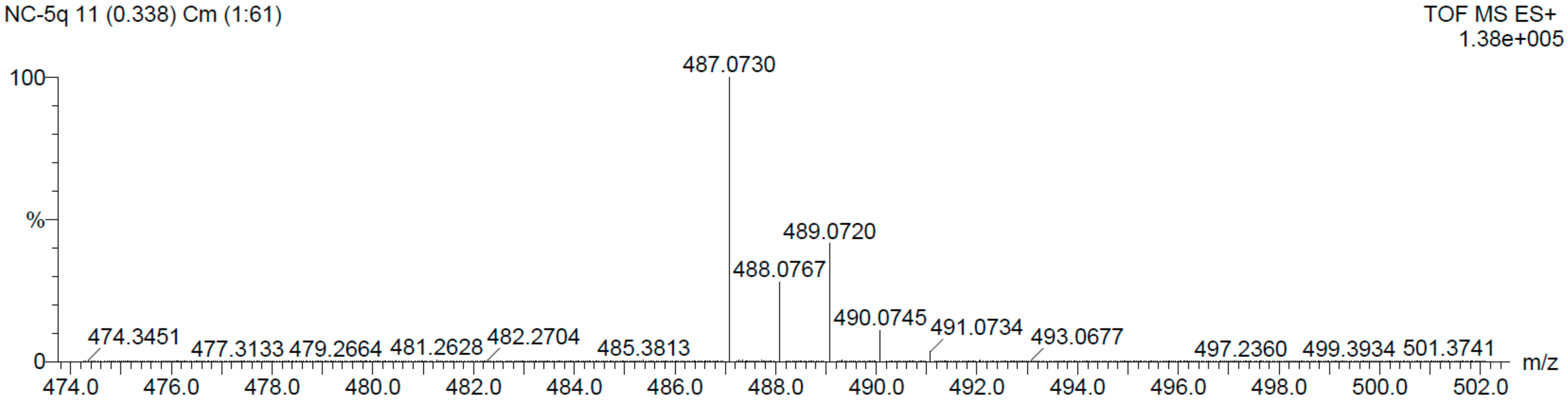

Minimum: -1.5
Maximum: 5.0 4.0 500.0

Mass	Calc. Mass	mDa	PPM	DBE	i-FIT	i-FIT (Norm)	Formula
487.0730	487.0720	1.0	2.1	16.5	508.7	0.0	C22 H17 N6 O2 Na S Cl

2-{[(1-(2,4-dichlorobenzyl)-1H-1,2,3-triazol-4-yl)methyl]thio}-5-[(quinolin-8-yloxy)methyl]-1,3,4-oxadiazole (**12i**)

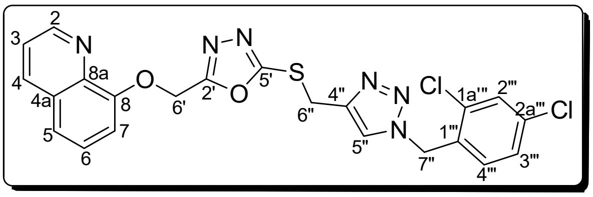

^{1}H NMR

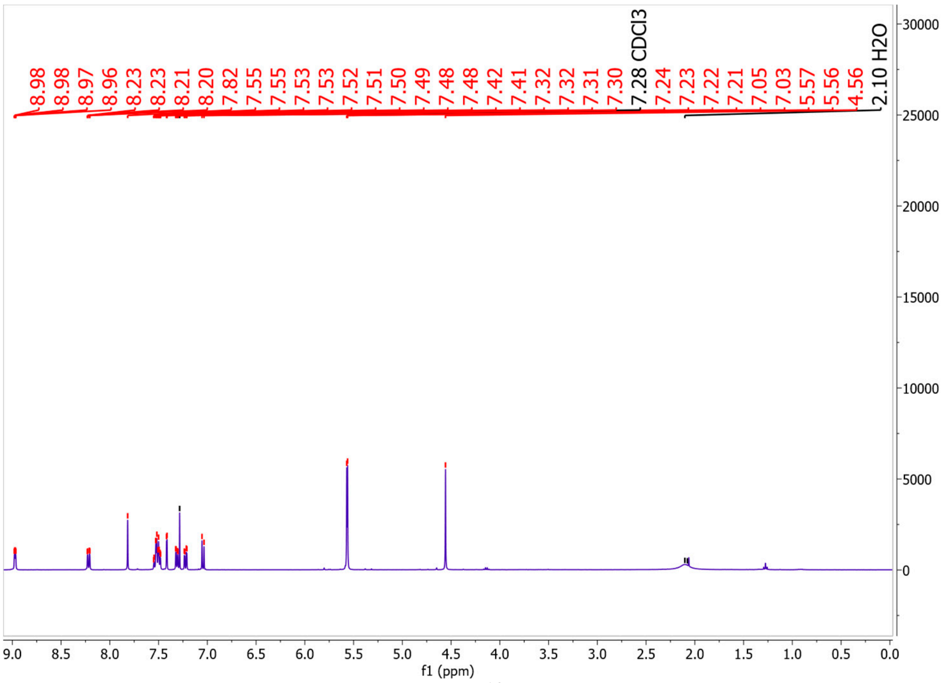

^{13}C NMR

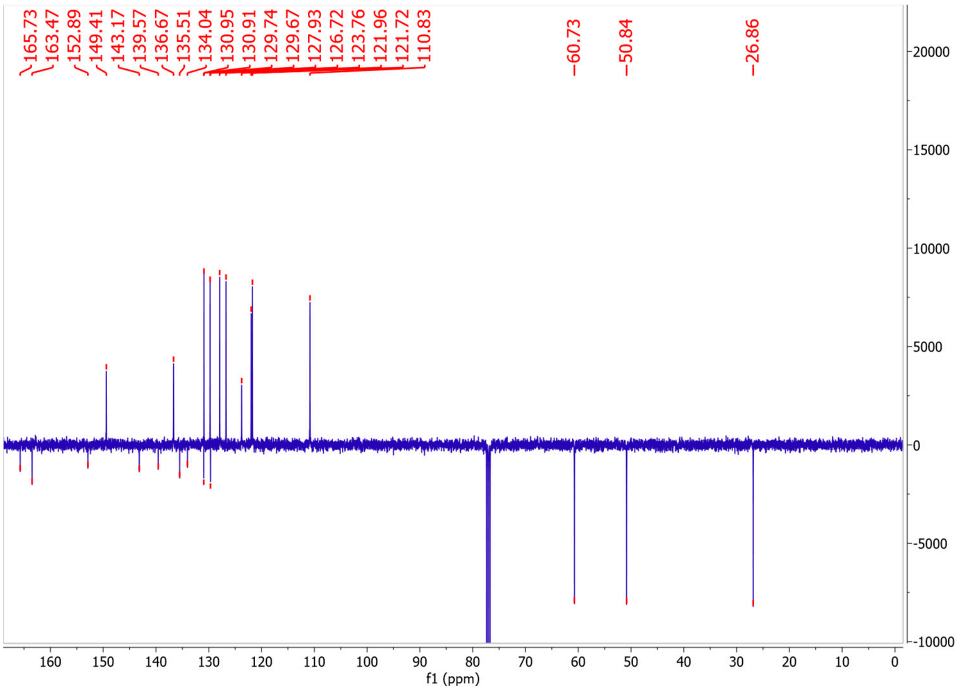

Elemental Composition Report_Compound 12i

Single Mass Analysis

Tolerance = 4.0 PPM / DBE: min = -1.5, max = 500.0
Element prediction: Off
Number of isotope peaks used for i-FIT = 3
Monoisotopic Mass, Even Electron Ions
89 formula(e) evaluated with 1 results within limits (all results (up to 1000) for each mass)
Elements Used:
C: 20-25 H: 15-20 N: 5-10 O: 0-5 Na: 1-1 S: 0-1 Cl: 0-2

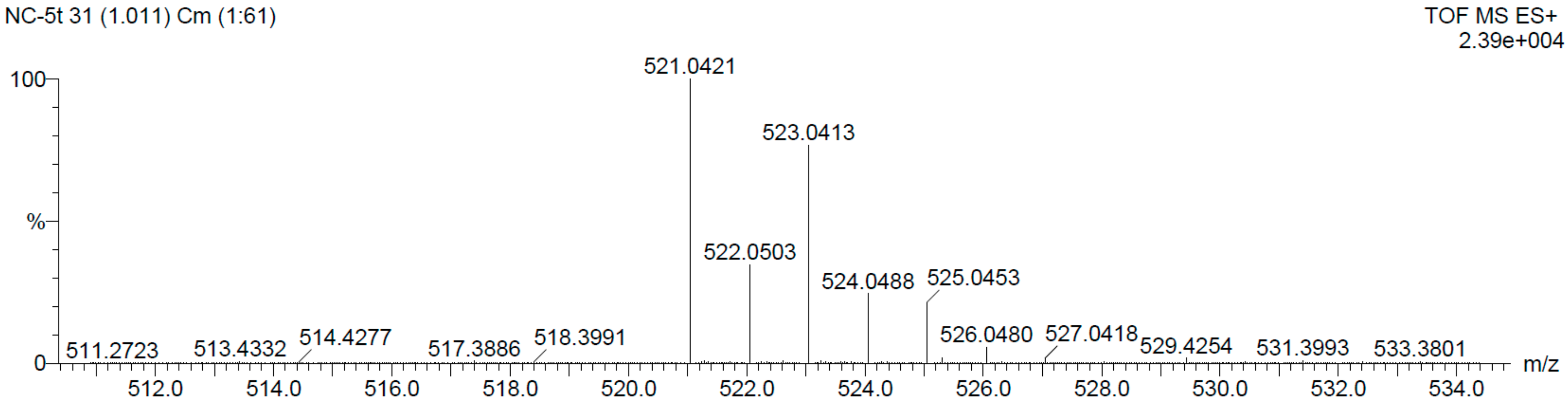

Minimum: -1.5
Maximum: 5.0 4.0 500.0

Mass	Calc. Mass	mDa	PPM	DBE	i-FIT	i-FIT (Norm)	Formula
			-1.5				
		5.0	4.0	500.0			
521.0421	521.0411	1.0	1.9	16.5	392.9	0.0	C21 H15 N6 O5 Na S Cl

2-{[(1-(3,4-difluorobenzyl)-1H-1,2,3-triazol-4-yl)methyl]thio}-5-[(quinolin-8-yloxy)methyl]-1,3,4-oxadiazole (**12j**)

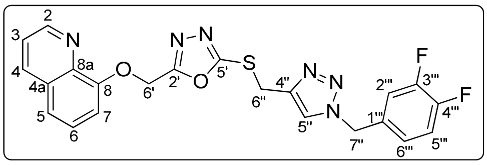

^{1}H NMR

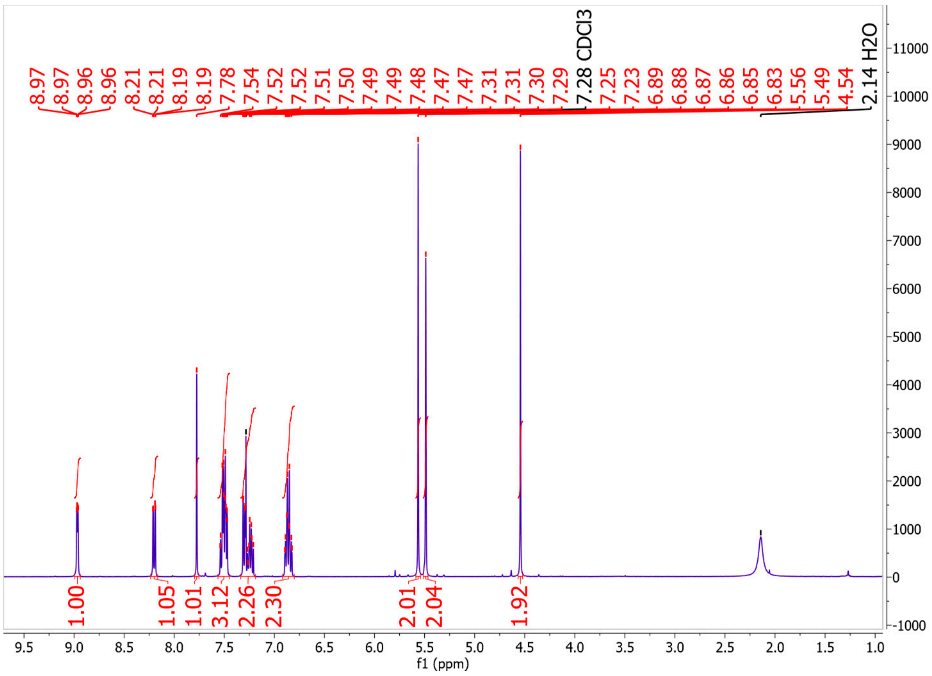

^{13}C NMR

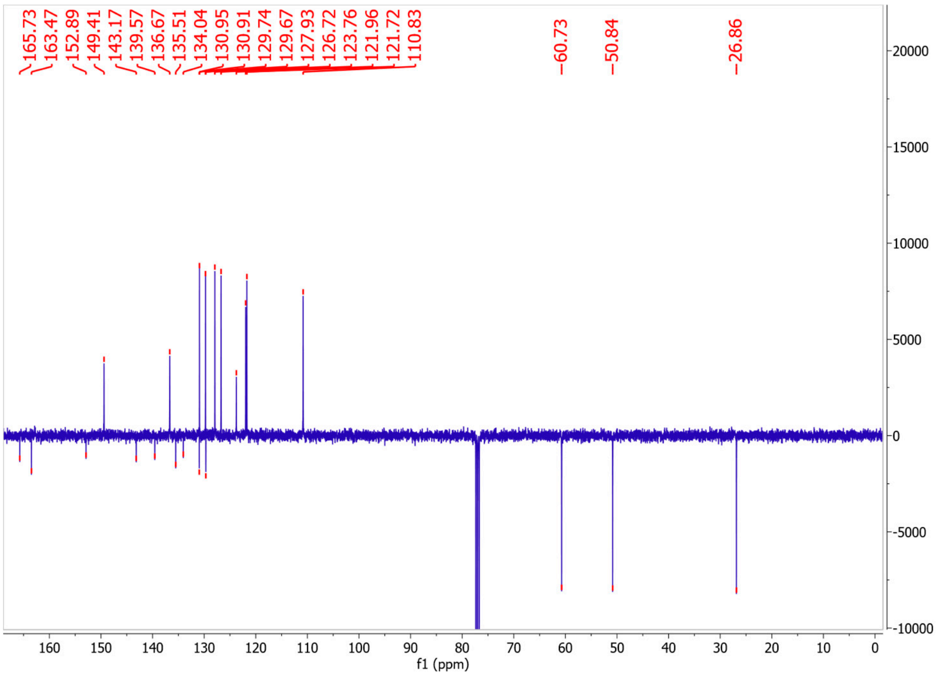

2-{[(1-phenyl-1H-1,2,3-triazol-4-yl)methyl]thio}-5-[(quinolin-8-yloxy)methyl]-1,3,4-oxadiazole (**12k**)

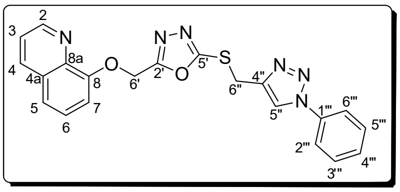

^{1}H NMR

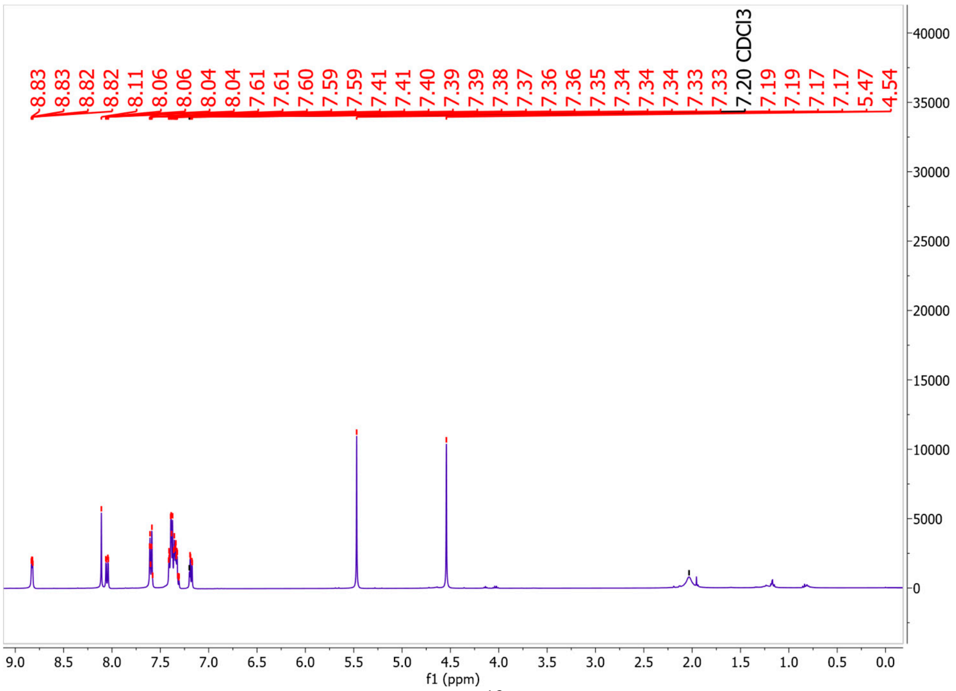

^{13}C NMR

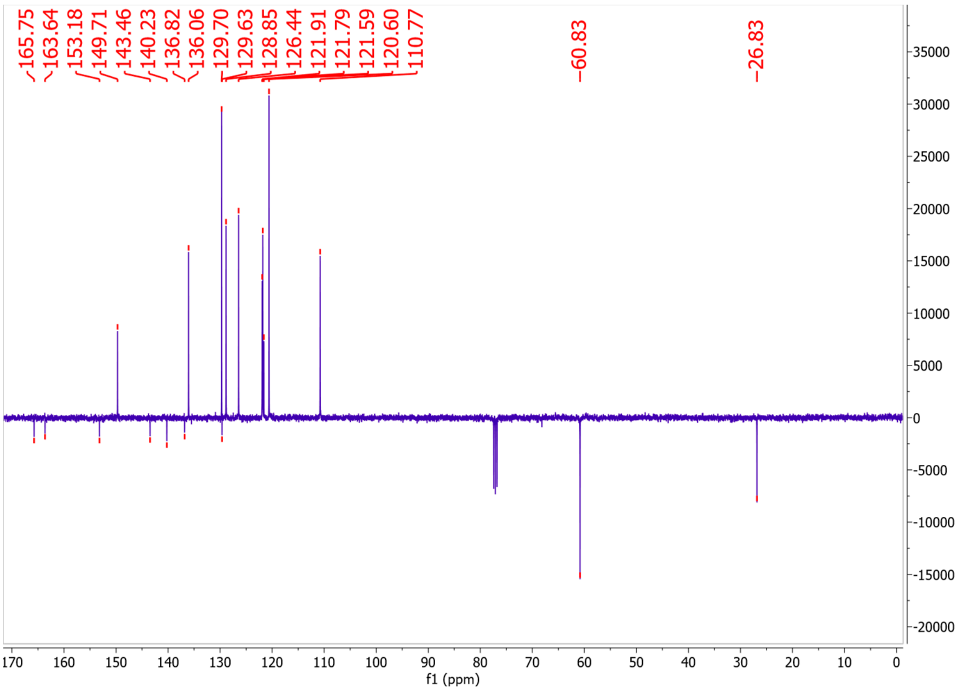

Elemental Composition Report_Compound 12k

Single Mass Analysis

Tolerance = 4.0 PPM / DBE: min = -1.5, max = 500.0
Element prediction: Off
Number of isotope peaks used for i-FIT = 3
Monoisotopic Mass, Even Electron Ions
69 formula(e) evaluated with 1 results within limits (all results (up to 1000) for each mass)
Elements Used:
C: 20-25 H: 15-20 N: 5-10 O: 0-5 Na: 0-1 S: 0-1

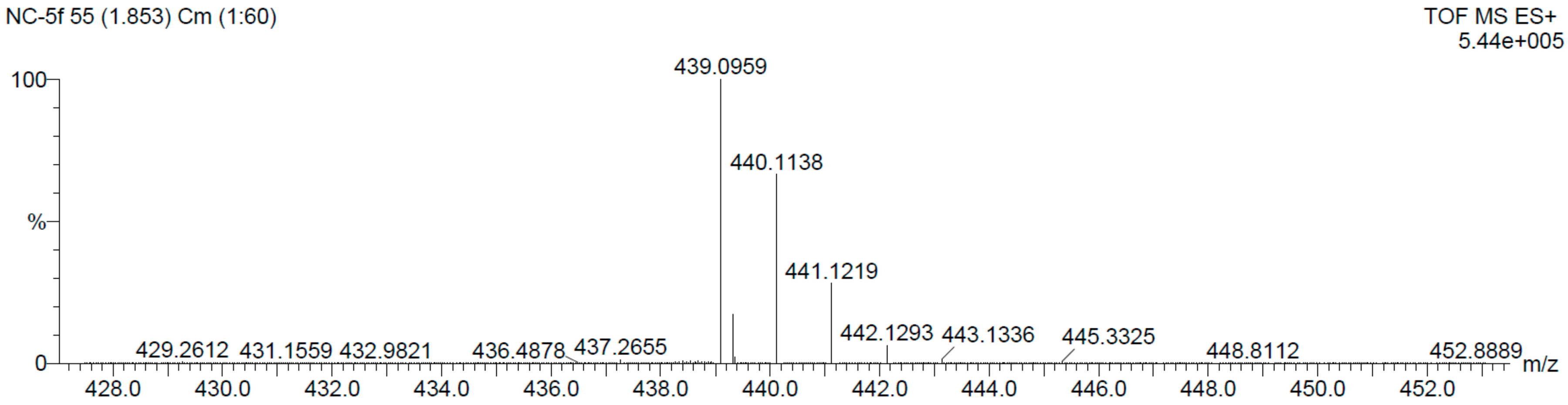


```
Minimum: -1.5
Maximum: 5.0 4.0 500.0
Mass Calc. Mass mDa PPM DBE i-FIT i-FIT (Norm) Formula
439.0959 439.0953 0.6 1.4 16.5 515.7 0.0 C21 H16 N6 O2 Na S
```

2-{[(1-(4-chlorophenyl)-1H-1,2,3-triazol-4-yl)methyl]thio}-5-[(quinolin-8-yloxy)methyl]-1,3,4-oxadiazole (**12*l***)

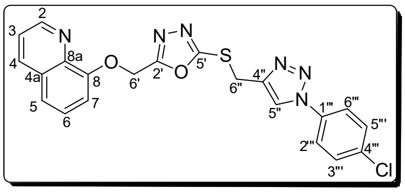

^{1}H NMR

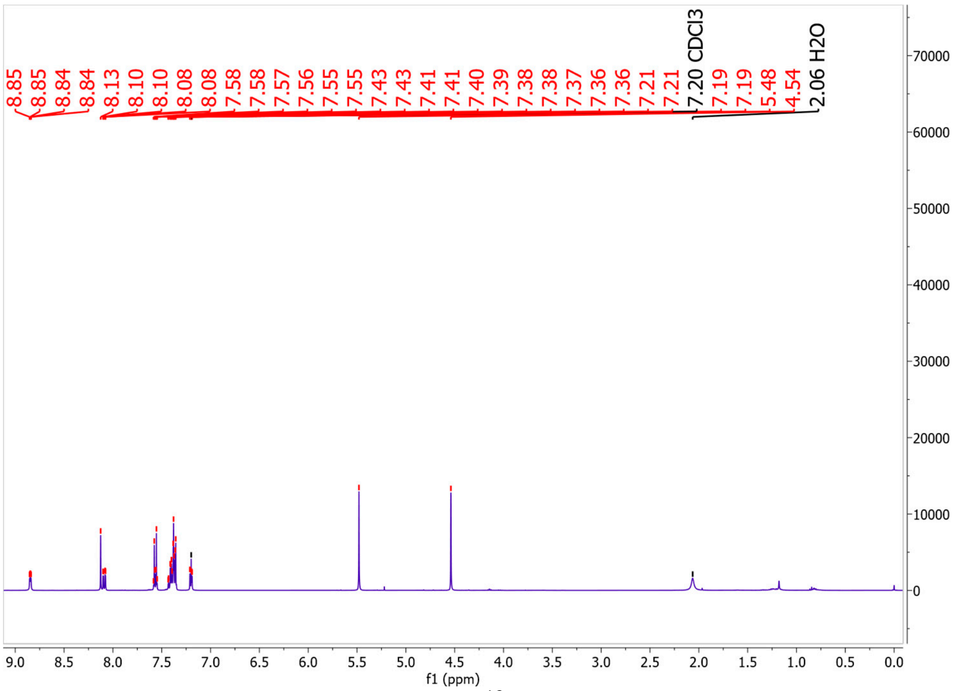

^{13}C NMR

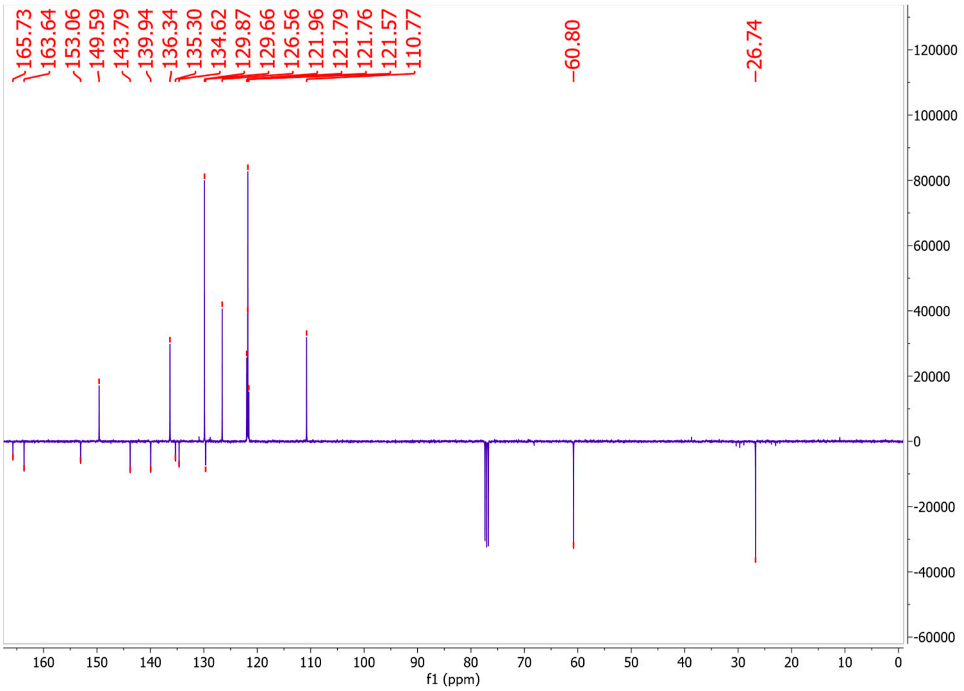

Elemental Composition Report_Compound 12*l*

Single Mass Analysis

Tolerance = 5.0 PPM / DBE: min = -1.5, max = 500.0
Element prediction: Off
Number of isotope peaks used for i-FIT = 3
Monoisotopic Mass, Even Electron Ions
127 formula(e) evaluated with 1 results within limits (all results (up to 1000) for each mass)
Elements Used:
C: 20-25 H: 15-20 N: 5-10 O: 0-5 Na: 0-1 S: 0-1 Cl: 0-1

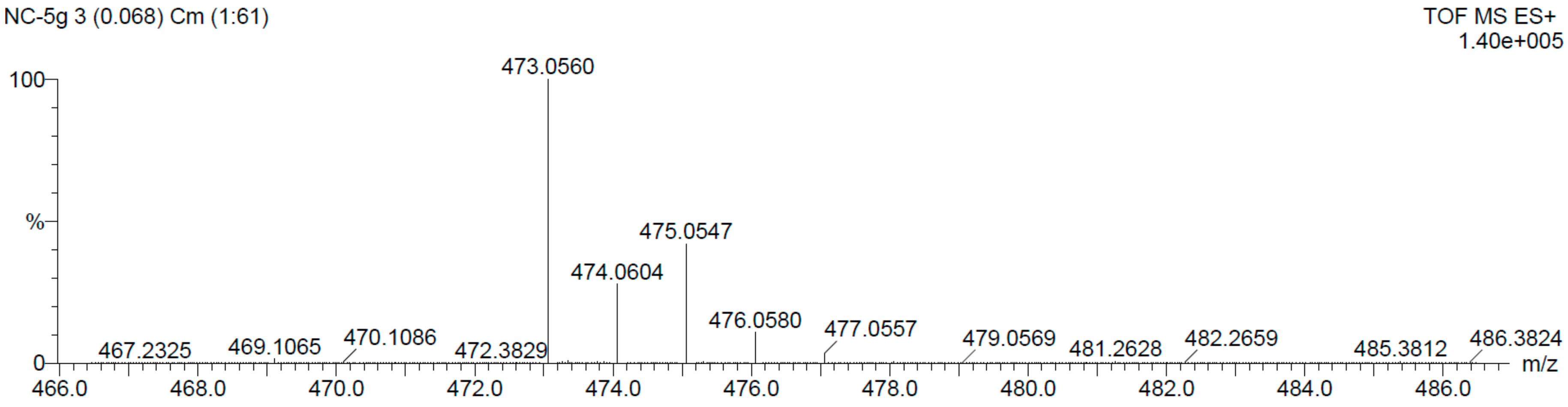


```
Minimum: -1.5
Maximum: 5.0 5.0 500.0
Mass Calc. Mass mDa PPM DBE i-FIT i-FIT (Norm) Formula
473.0560 473.0563 -0.3 -0.6 16.5 504.3 0.0 C21 H15 N6 O2 Na S Cl
```

2-{[(1-(4-fluorophenyl)-1H-1,2,3-triazol-4-yl)methyl]thio}-5-[(quinolin-8-yloxy)methyl]-1,3,4-oxadiazole (**12m**)

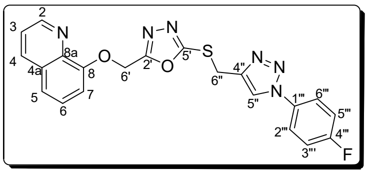

^{1}H NMR

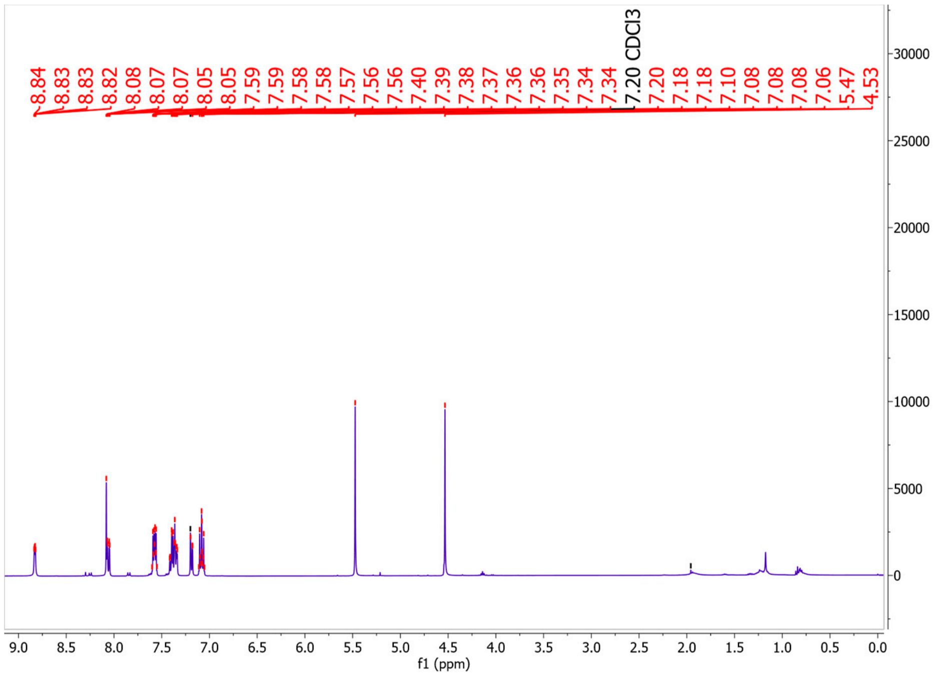

^{13}C NMR

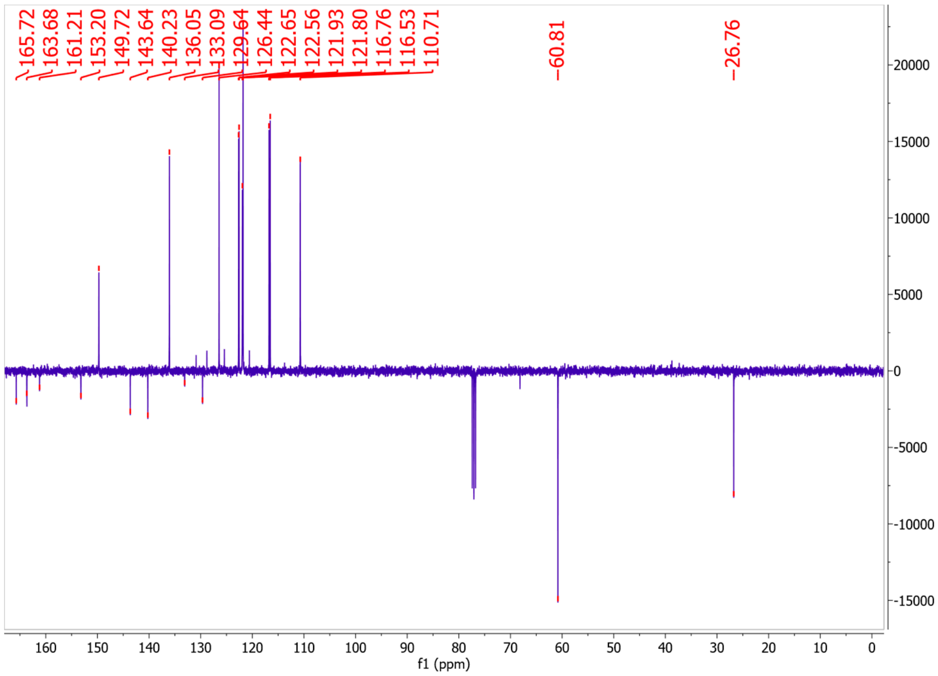

Elemental Composition Report_Compound 12m

Single Mass Analysis

Tolerance = 5.0 PPM / DBE: min = -1.5, max = 500.0
Element prediction: Off
Number of isotope peaks used for i-FIT = 3

Monoisotopic Mass, Even Electron Ions
134 formula(e) evaluated with 1 results within limits (all results (up to 1000) for each mass)
Elements Used:
C: 20-25 H: 15-20 N: 5-10 O: 0-5 Na: 0-1 S: 0-1 F: 0-1

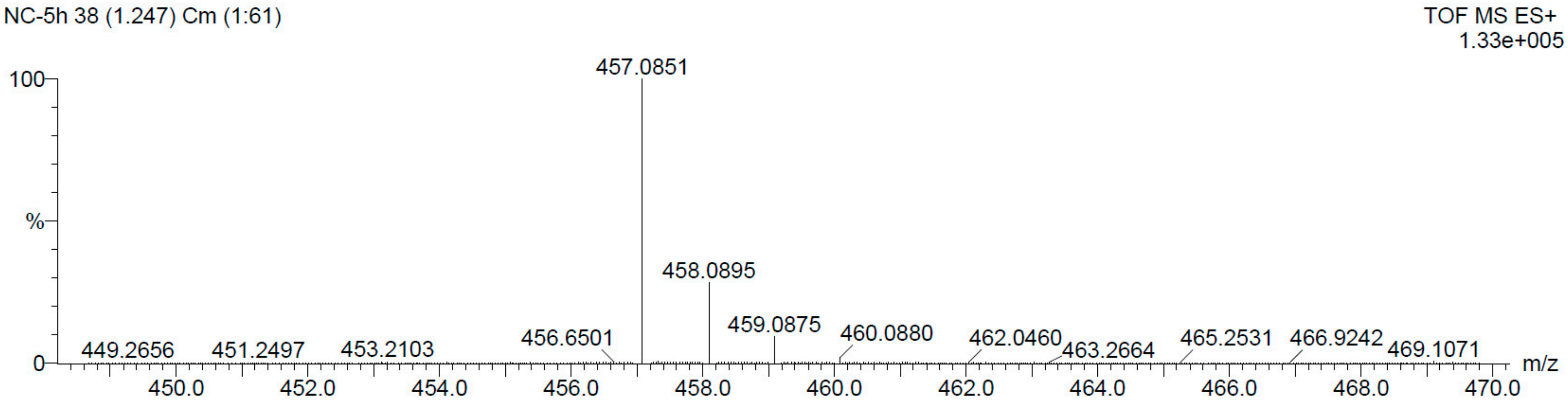

Minimum: -1.5
Maximum: 5.0 5.0 500.0

Mass	Calc. Mass	mDa	PPM	DBE	i-FIT	i-FIT (Norm)	Formula
457.0851	457.0859	-0.8	-1.8	16.5	521.4	0.0	C21 H15 N6 O2 Na S F

2-{[(1-(4-bromophenyl)-1H-1,2,3-triazol-4-yl)methyl]thio}-5-[(quinolin-8-yloxy)methyl]-1,3,4-oxadiazole (**12n**)

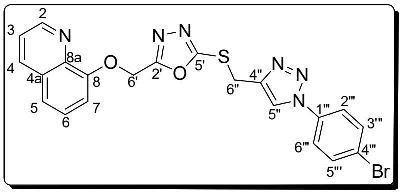

^{1}H NMR

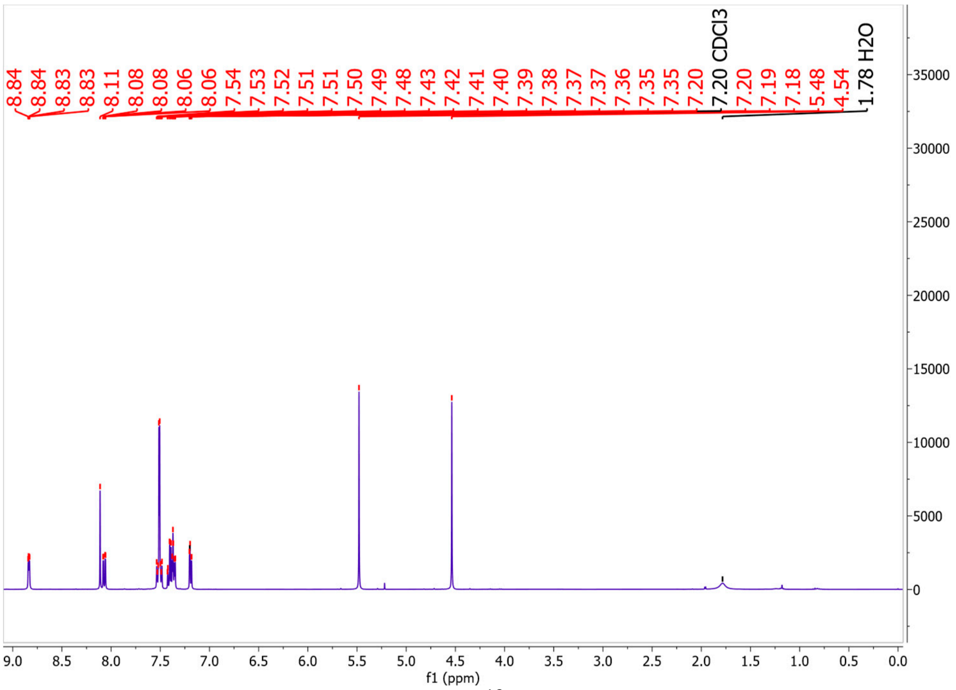

^{13}C NMR

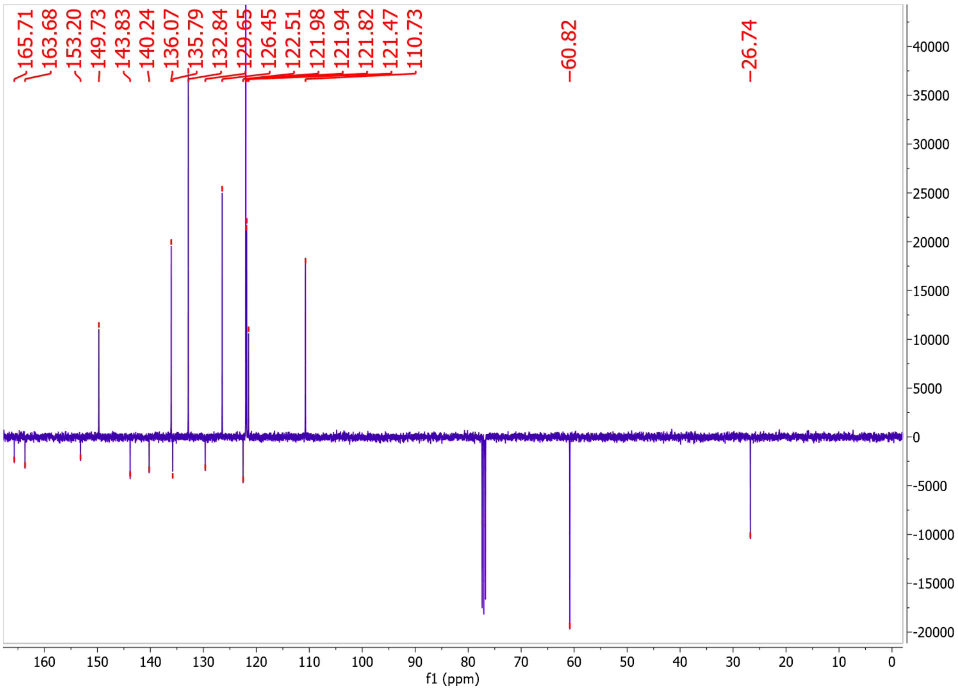

Single Mass Analysis

Tolerance = 5.0 PPM / DBE: min = -1.5, max = 500.0
Element prediction: Off
Number of isotope peaks used for i-FIT = 3
Monoisotopic Mass, Even Electron Ions
130 formula(e) evaluated with 1 results within limits (all results (up to 1000) for each mass)
Elements Used:
C: 20-25 H: 15-20 N: 5-10 O: 0-5 Na: 0-1 S: 0-1 Br: 0-1

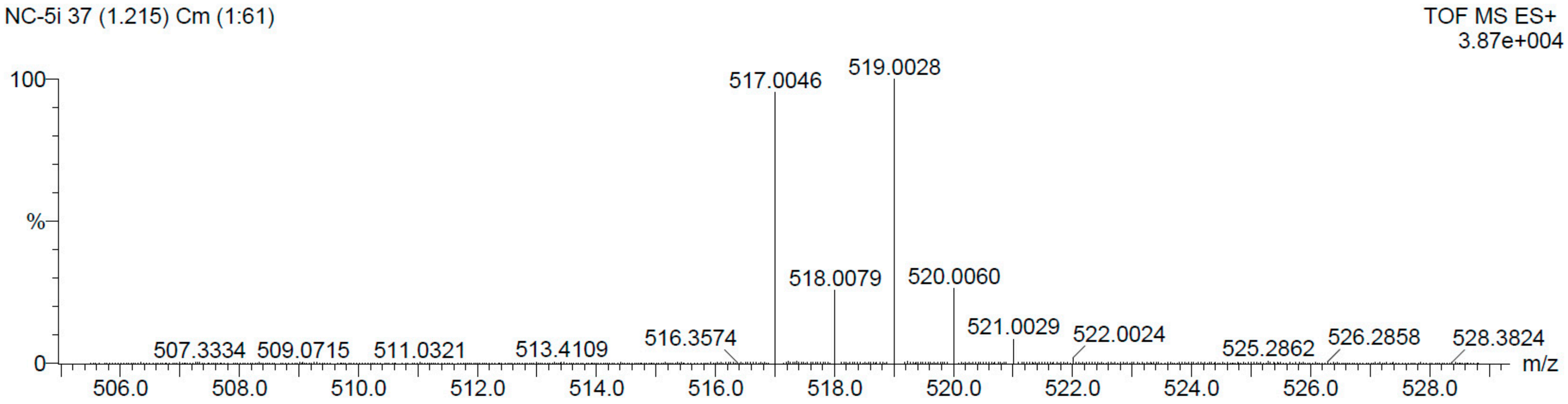


```
Minimum: -1.5
Maximum: 5.0 5.0 500.0
Mass Calc. Mass mDa PPM DBE i-FIT i-FIT (Norm) Formula
517.0046 517.0058 -1.2 -2.3 16.5 404.1 0.0 C21 H15 N6 O2 Na S Br
```

2-{[(1-(4-nitrophenyl)-1H-1,2,3-triazol-4-yl)methyl]thio}-5-[(quinolin-8-yloxy)methyl]-1,3,4-oxadiazole (**12o**)

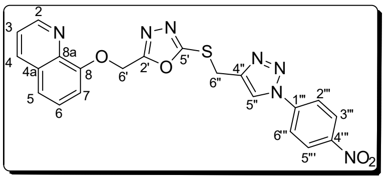

^{1}H NMR

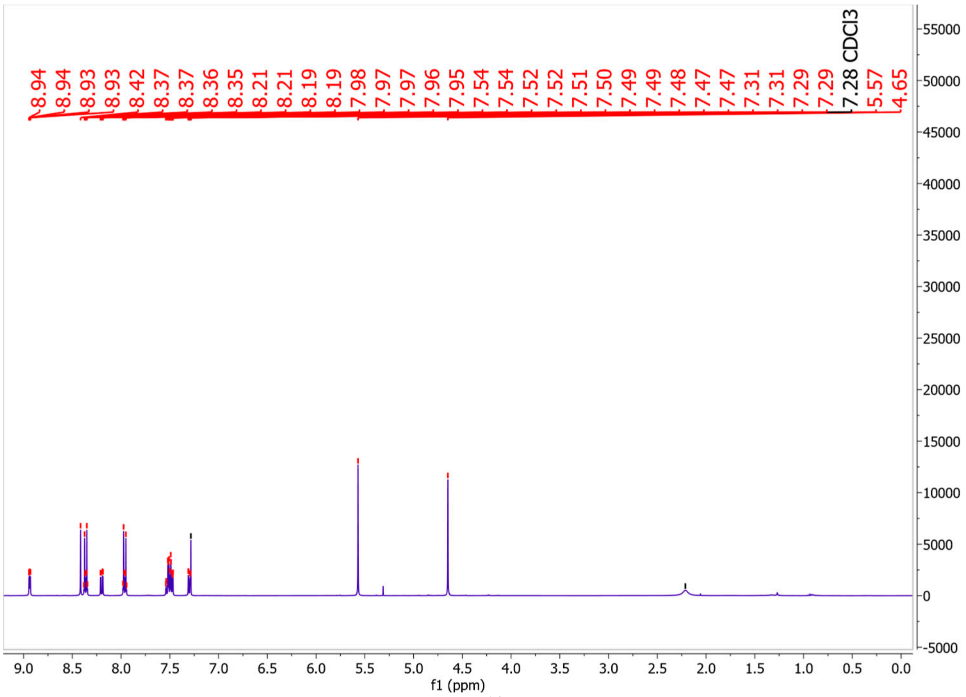

^{13}C NMR

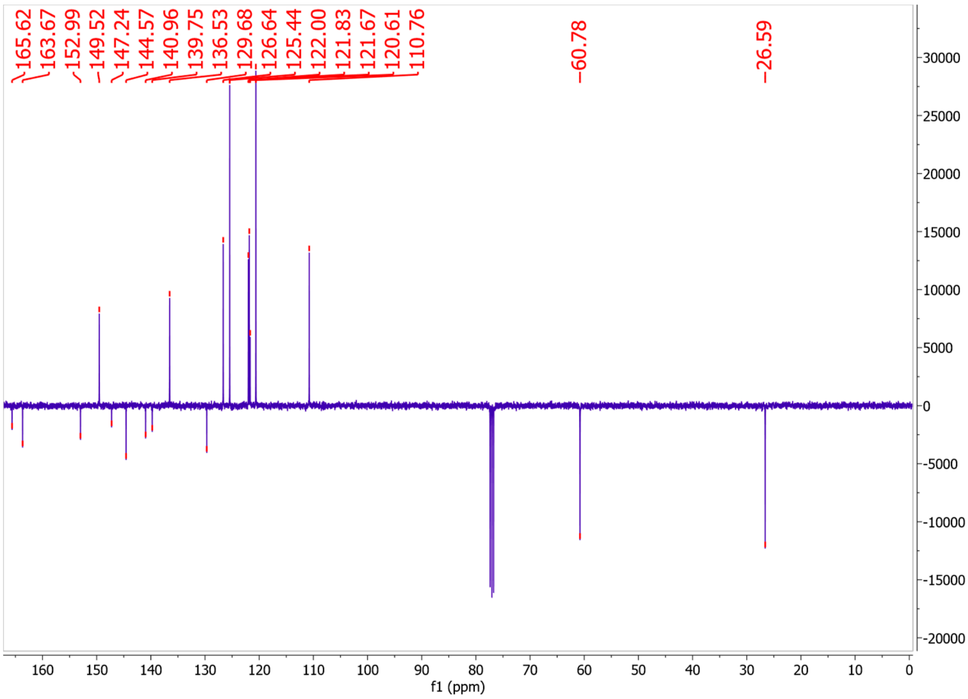

Single Mass Analysis

Tolerance = 5.0 PPM / DBE: min = -1.5, max = 500.0
Element prediction: Off
Number of isotope peaks used for i-FIT = 3

Monoisotopic Mass, Even Electron Ions
53 formula(e) evaluated with 1 results within limits (all results (up to 1000) for each mass)
Elements Used:
C: 20-25 H: 15-20 N: 5-10 O: 0-5 Na: 0-1 S: 0-1

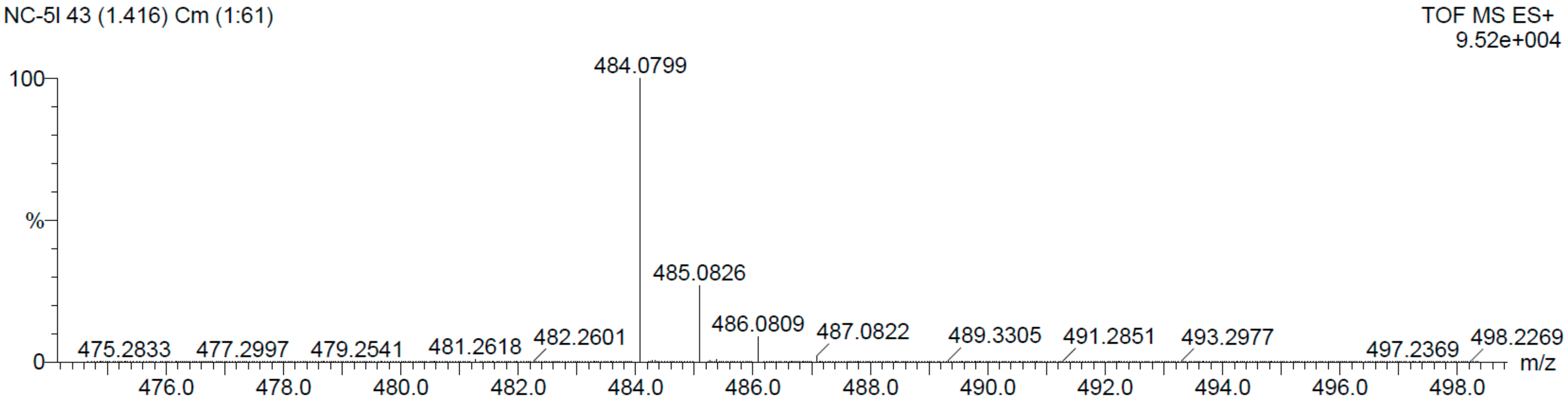

Minimum: -1.5
Maximum: 5.0 5.0 500.0

Mass	Calc. Mass	mDa	PPM	DBE	i-FIT	i-FIT (Norm)	Formula
484.0799	484.0804	-0.5	-1.0	17.5	465.6	0.0	C21 H15 N7 O4 Na S

2-[(quinolin-8-yloxy)methyl]-5-{[(1-(p-tolyl)-1H-1,2,3-triazol-4-yl)methyl]thio}-1,3,4-oxadiazole (**12p**)

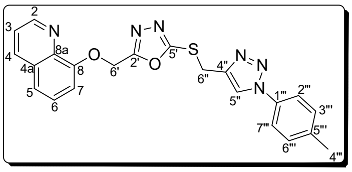

^{1}H NMR

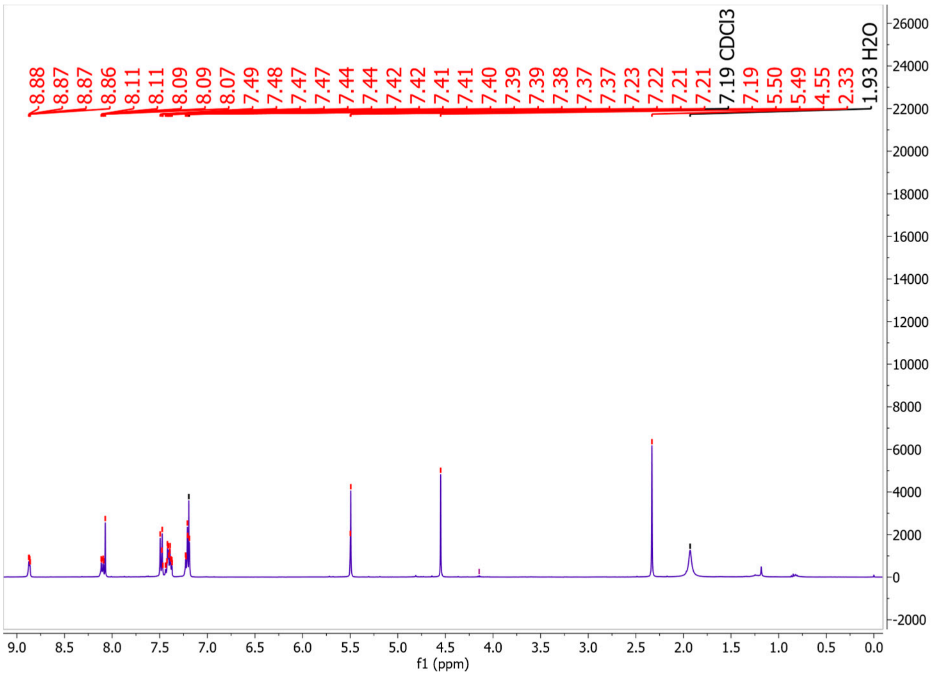

^{13}C NMR

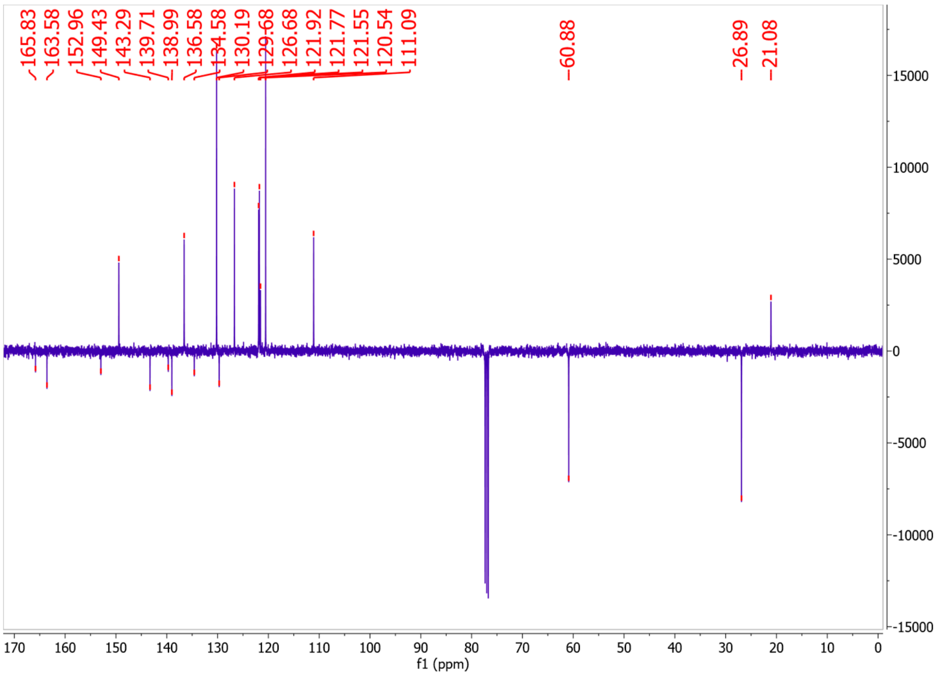

Elemental Composition Report_Compound 12p

Single Mass Analysis

Tolerance = 5.0 PPM / DBE: min = -1.5, max = 500.0
Element prediction: Off
Number of isotope peaks used for i-FIT = 3

Monoisotopic Mass, Even Electron Ions
63 formula(e) evaluated with 1 results within limits (all results (up to 1000) for each mass)
Elements Used:
C: 20-25 H: 15-20 N: 5-10 O: 0-5 Na: 0-1 S: 0-1

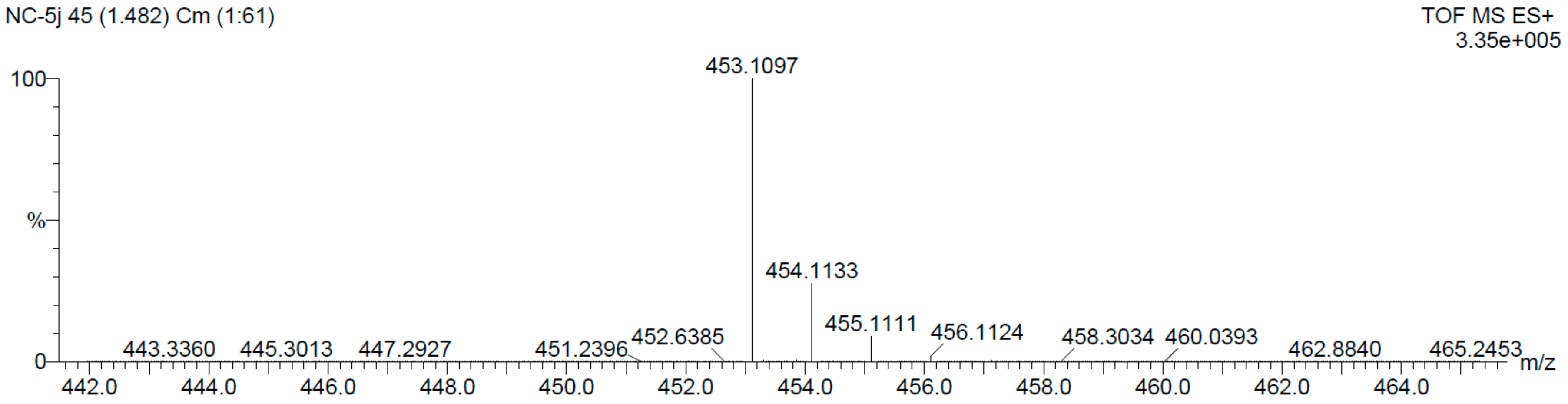

Minimum: -1.5
Maximum: 5.0 5.0 500.0

Mass	Calc. Mass	mDa	PPM	DBE	i-FIT	i-FIT (Norm)	Formula
453.1097	453.1110	-1.3	-2.9	16.5	588.2	0.0	C22 H18 N6 O2 Na S

2-{[(1-(4-methoxyphenyl)-1H-1,2,3-triazol-4-yl)methyl]thio}-5-[(quinolin-8-yloxy)methyl]-1,3,4-oxadiazole (**12q**)

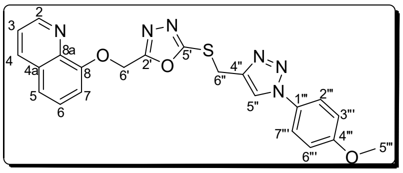

^{1}H NMR

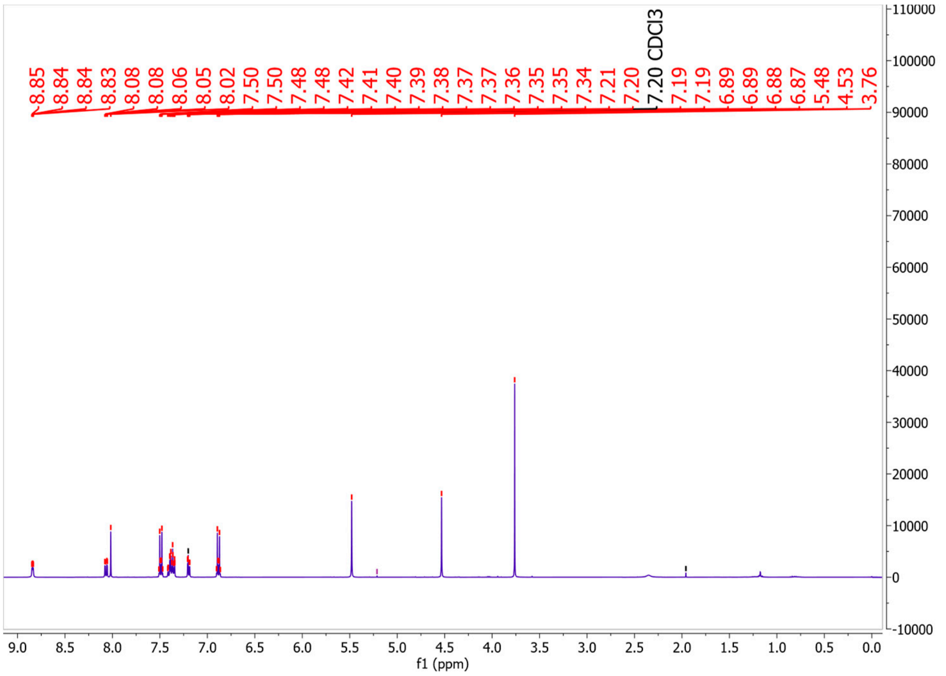

^{13}C NMR

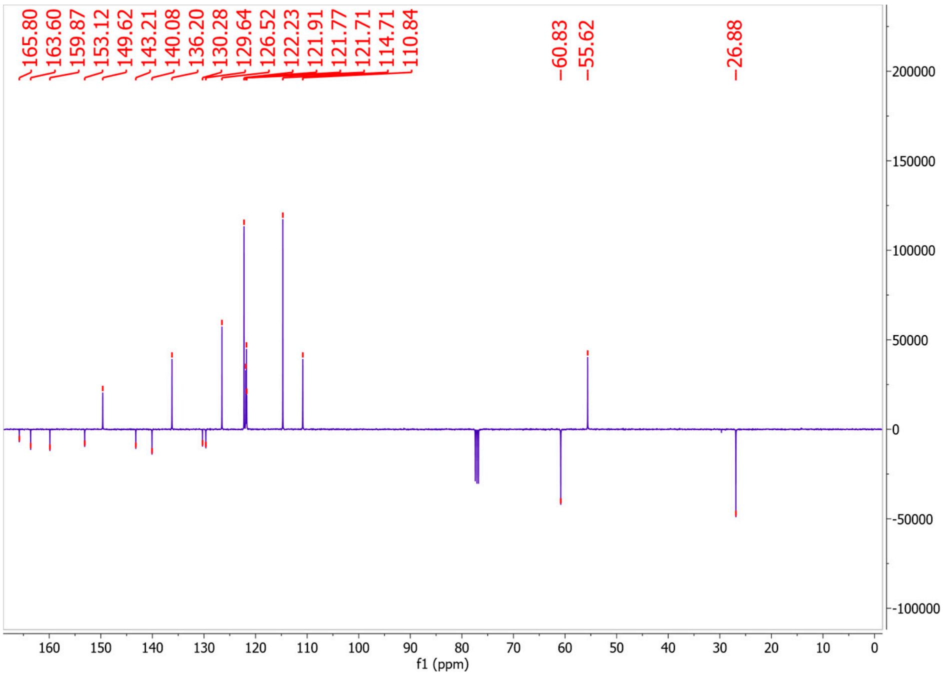

Elemental Composition Report_12q

Single Mass Analysis

Tolerance = 5.0 PPM / DBE: min = -1.5, max = 500.0
Element prediction: Off
Number of isotope peaks used for i-FIT = 3
Monoisotopic Mass, Even Electron Ions
55 formula(e) evaluated with 1 results within limits (all results (up to 1000) for each mass)
Elements Used:
C: 20-25 H: 15-20 N: 5-10 O: 0-5 Na: 0-1 S: 0-1

NC-5k 25 (0.809) Cm (1:61)

TOF MS ES+
3.67e+005

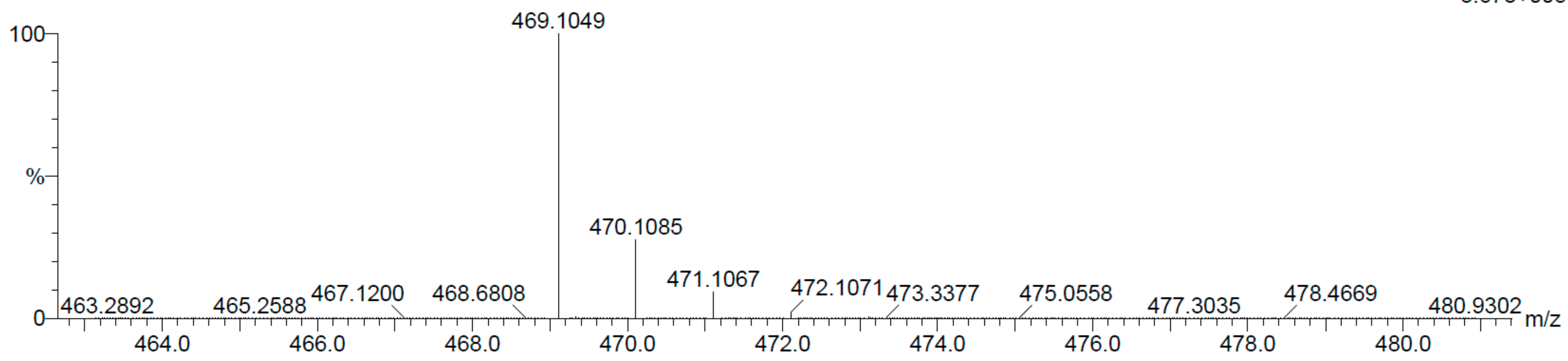

Minimum: -1.5
Maximum: 5.0 5.0 500.0

Mass	Calc. Mass	mDa	PPM	DBE	i-FIT	i-FIT (Norm)	Formula
469.1049	469.1059	-1.0	-2.1	16.5	587.7	0.0	C22 H18 N6 O3 Na S

2-{[(1-(2,4-dichlorophenyl)-1H-1,2,3-triazol-4-yl)methyl]thio}-5-[(quinolin-8-yloxy)methyl]-1,3,4-oxadiazole (**12r**)

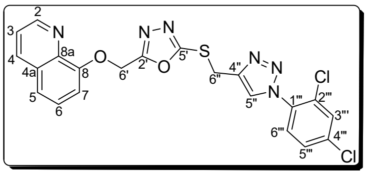

^{1}H NMR

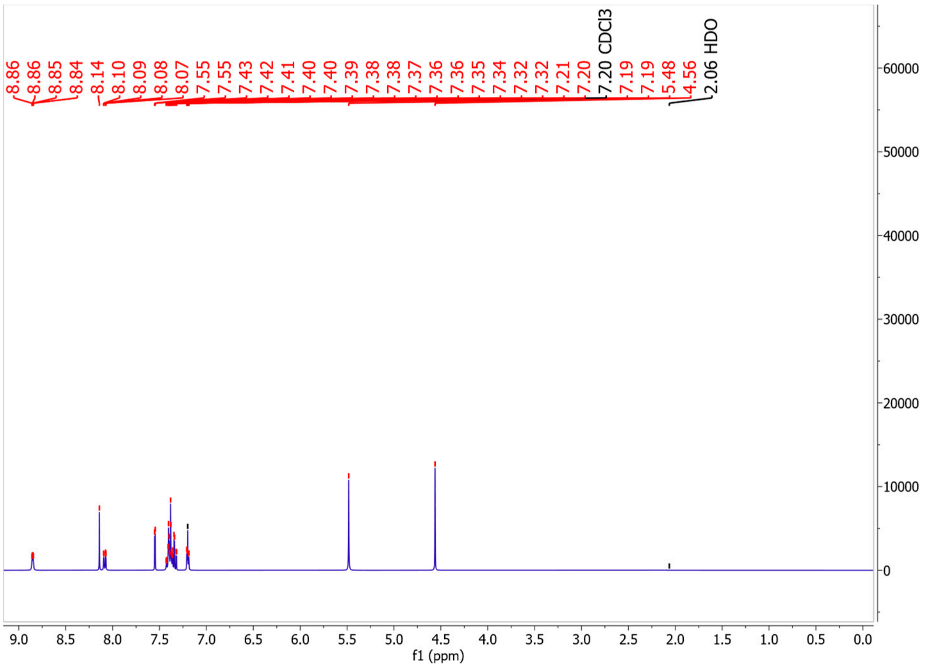

^{13}C NMR

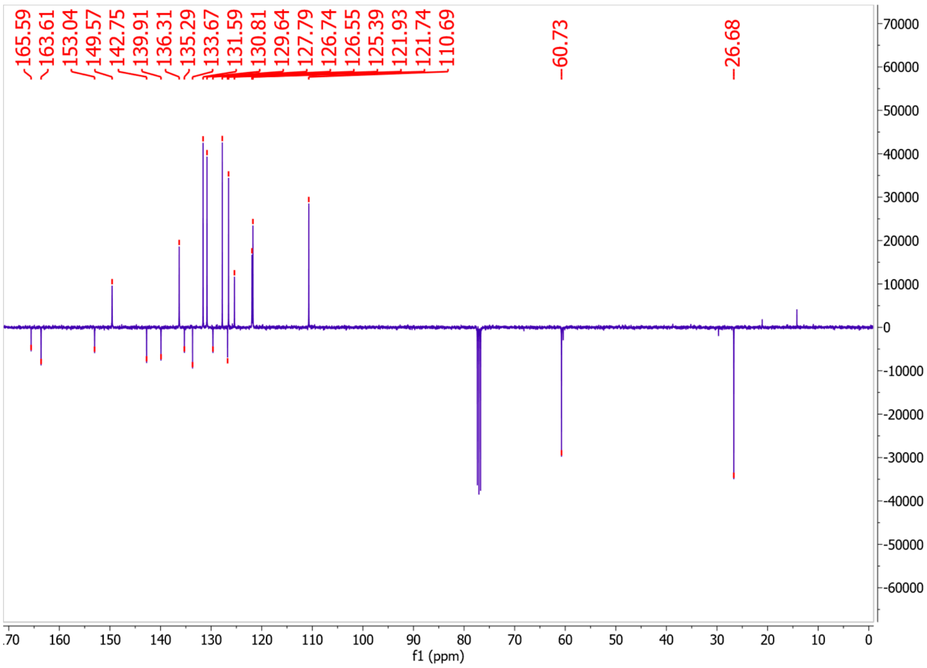

Elemental Composition Report_Compound 12r

Single Mass Analysis

Tolerance = 4.0 PPM / DBE: min = -1.5, max = 500.0
Element prediction: Off
Number of isotope peaks used for i-FIT = 3
Monoisotopic Mass, Even Electron Ions
191 formula(e) evaluated with 1 results within limits (all results (up to 1000) for each mass)
Elements Used:
C: 20-25 H: 10-15 N: 5-10 O: 0-5 Na: 0-1 S: 0-1 Cl: 0-2

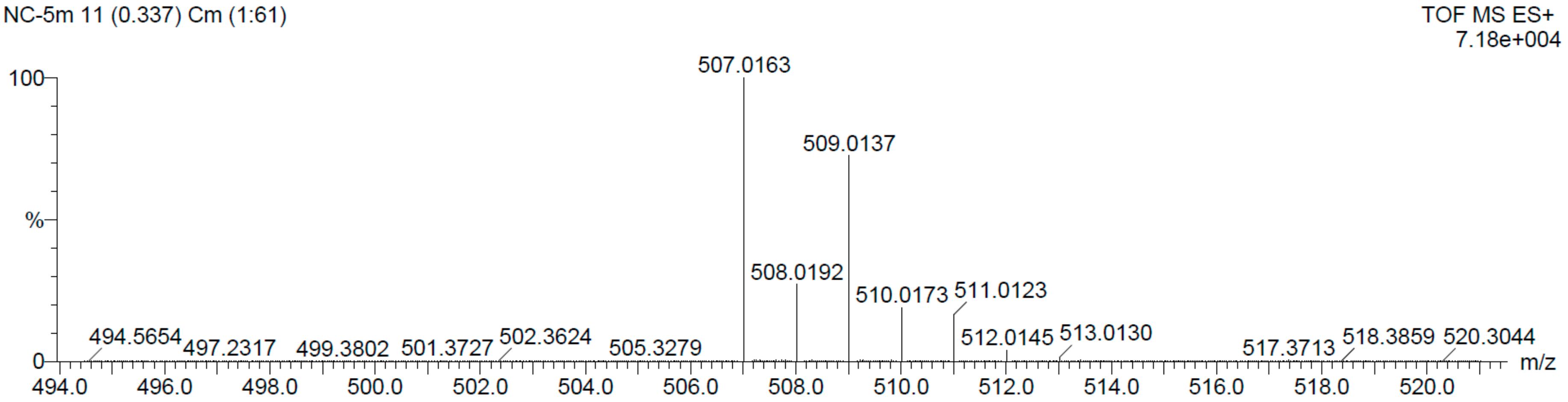

Minimum: -1.5
Maximum: 5.0 4.0 500.0

Mass	Calc. Mass	mDa	PPM	DBE	i-FIT	i-FIT (Norm)	Formula
507.0163	507.0174	-1.1	-2.2	16.5	470.1	0.0	C21 H14 N6 O2 Na S Cl2

2-{[(1-(2,6-dichlorophenyl)-1H-1,2,3-triazol-4-yl)methyl]thio}-5-[(quinolin-8-yloxy)methyl]-1,3,4-oxadiazole (**12s**)

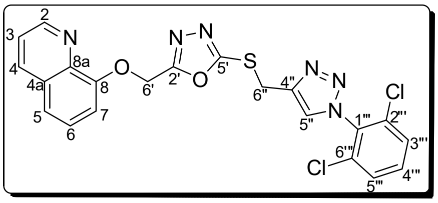

^{1}H NMR

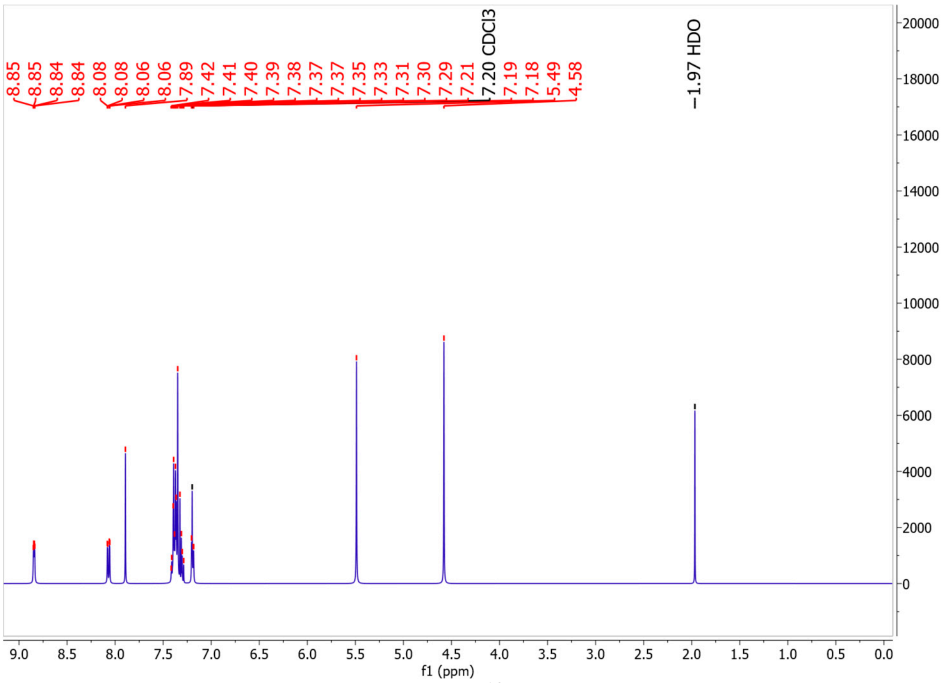

^{13}C NMR

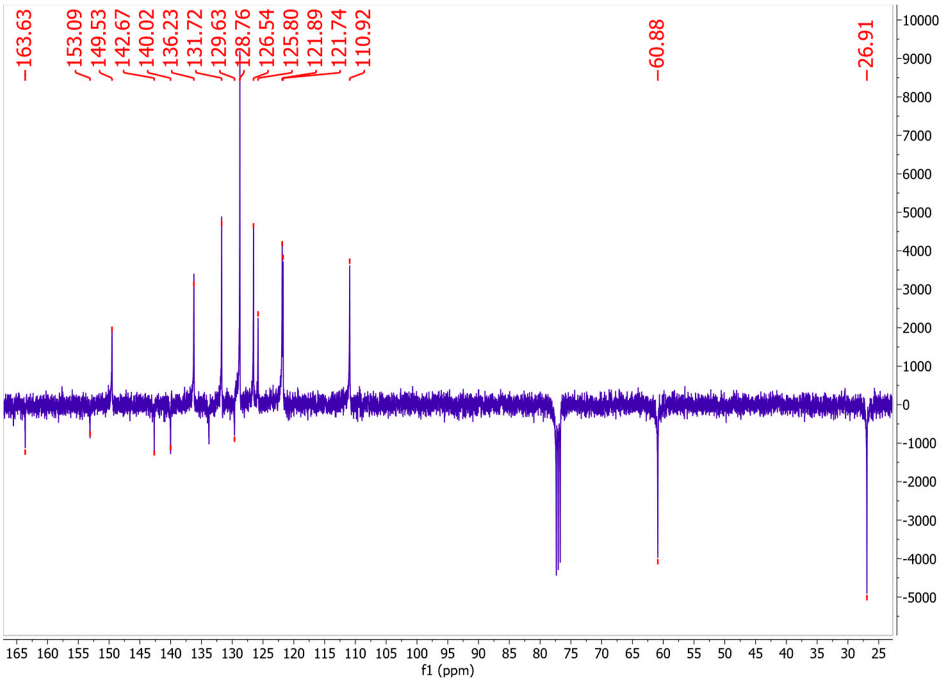

Elemental Composition Report_Compound 12s

Single Mass Analysis

Tolerance = 4.0 PPM / DBE: min = -1.5, max = 500.0
Element prediction: Off
Number of isotope peaks used for i-FIT = 3
Monoisotopic Mass, Even Electron Ions
191 formula(e) evaluated with 1 results within limits (all results (up to 1000) for each mass)
Elements Used:
C: 20-25 H: 10-15 N: 5-10 O: 0-5 Na: 0-1 S: 0-1 Cl: 0-2

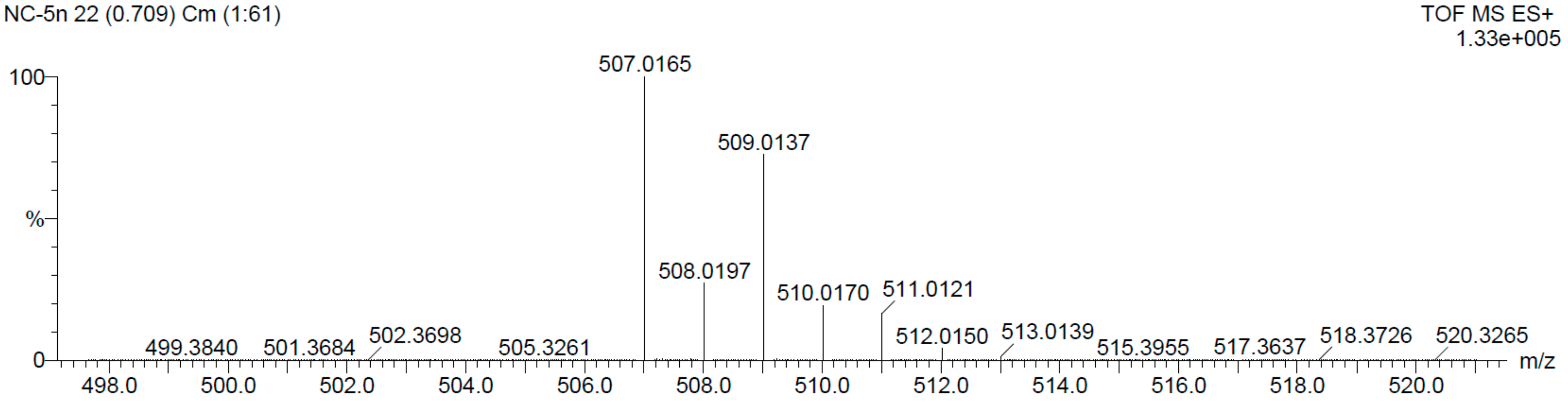


```
Minimum: -1.5
Maximum: 5.0 4.0 500.0
Mass Calc. Mass mDa PPM DBE i-FIT i-FIT (Norm) Formula
507.0165 507.0174 -0.9 -1.8 16.5 476.7 0.0 C21 H14 N6 O2 Na S Cl2
```

2-{[(1-(2,6-dimethylphenyl)-1H-1,2,3-triazol-4-yl)methyl]thio}-5-[(quinolin-8-yloxy)methyl]-1,3,4-oxadiazole (**12t**)

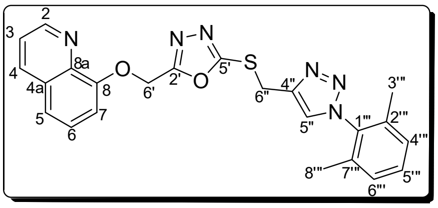

^{1}H NMR

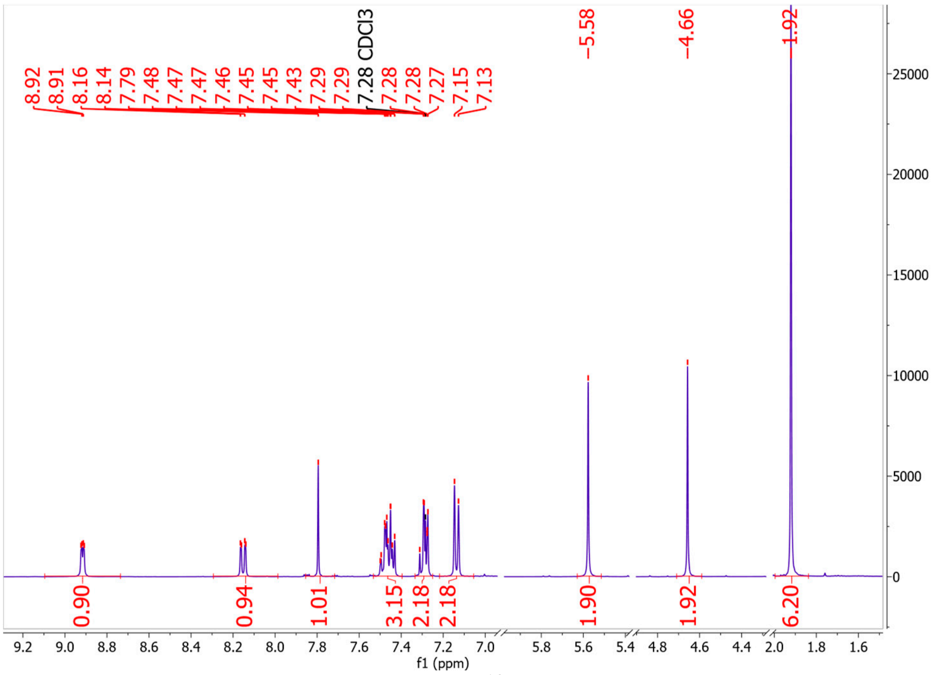

^{13}C NMR

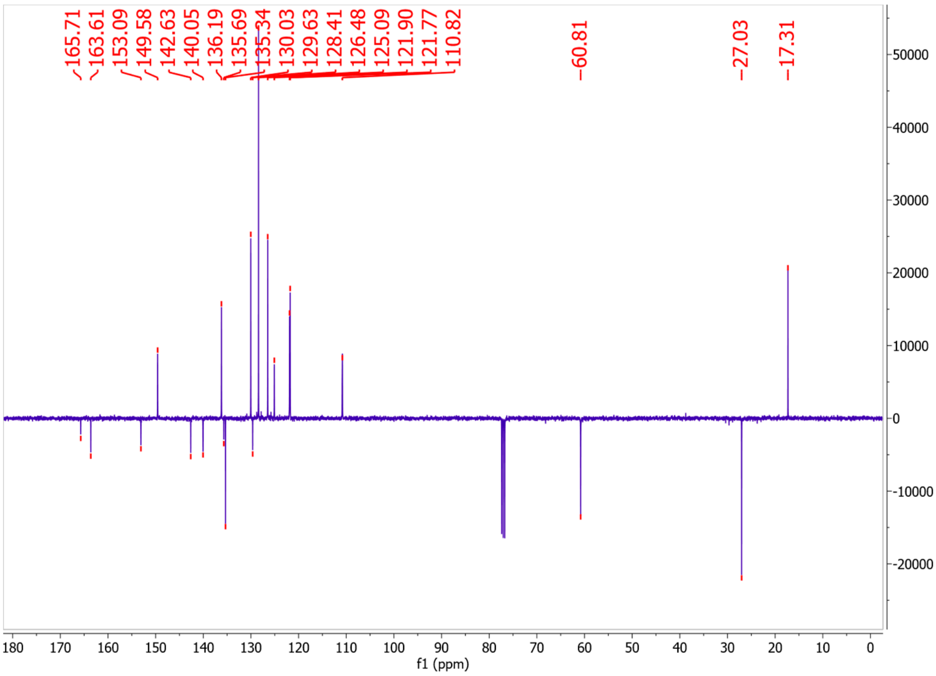

Elemental Composition Report_Compound 12t

Single Mass Analysis

Tolerance = 4.0 PPM / DBE: min = -1.5, max = 500.0
Element prediction: Off
Number of isotope peaks used for i-FIT = 3

Monoisotopic Mass, Even Electron Ions
29 formula(e) evaluated with 1 results within limits (all results (up to 1000) for each mass)
Elements Used:
C: 20-25 H: 15-20 N: 5-10 O: 0-5 Na: 1-1 S: 0-1

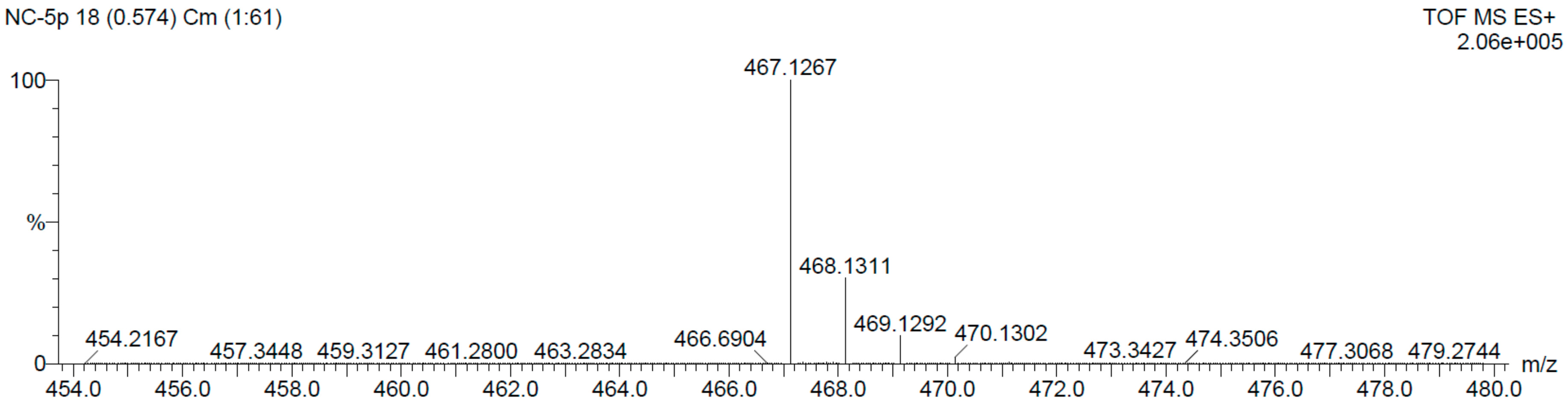

Minimum: -1.5
Maximum: 5.0 4.0 500.0

Mass	Calc. Mass	mDa	PPM	DBE	i-FIT	i-FIT (Norm)	Formula
467.1267	467.1266	0.1	0.2	16.5	537.3	0.0	C23 H20 N6 O2 Na S
